# Funnel metadynamics and behavioral studies reveal complex effect of D2AAK1 ligand on anxiety-like processes

**DOI:** 10.1038/s41598-022-25478-7

**Published:** 2022-12-07

**Authors:** Damian Bartuzi, Ewa Kędzierska, Katarzyna M. Targowska-Duda, Oliwia Koszła, Tomasz M. Wróbel, Simon Jademyr, Tadeusz Karcz, Katarzyna Szczepańska, Piotr Stępnicki, Olga Wronikowska-Denysiuk, Grażyna Biała, Jadwiga Handzlik, Jesper L. Kristensen, Antti Poso, Agnieszka A. Kaczor

**Affiliations:** 1 Department of Synthesis and Chemical Technology of Pharmaceutical Substances with Computer Modeling Laboratory, Faculty of Pharmacy, Medical University of Lublin, 4A Chodźki St., 20093 Lublin, Poland; 2 Science for Life Laboratory, Department of Cell and Molecular Biology, Uppsala University, 75124 Uppsala, Sweden; 3 Department of Pharmacology and Pharmacodynamics, Faculty of Pharmacy, Medical University of Lublin, 4A Chodźki St., 20093 Lublin, Poland; 4 Department of Biopharmacy, Faculty of Pharmacy, Medical University of Lublin, 4A Chodźki St., 20093 Lublin, Poland; 5 Department of Drug Design and Pharmacology, Faculty of Health and Medical Sciences, University of Copenhagen, Universitetsparken 2, 2100 Copenhagen, Denmark; 6 Department of Technology and Biotechnology of Drugs, Faculty of Pharmacy, Jagiellonian University, Medical College, Medyczna 9, 30-688 Kraków, Poland; 7 Independent Laboratory of Behavioral Studies, Chair of Biomedical Sciences, Faculty of Biomedicine, Medical University of Lublin, 4A Chodźki St., 20093 Lublin, Poland; 8 School of Pharmacy, University of Eastern Finland, Yliopistonranta 1, P.O. Box 1627, 70211 Kuopio, Finland; 9 Department of Internal Medicine VIII, University Hospital Tübingen, Otfried-Müller-Strasse 14, 72076 Tübingen, Germany; 10 Department of Pharmaceutical and Medicinal Chemistry, Institute of Pharmaceutical Sciences, Eberhard-Karls-Universität, Auf der Morgenstelle 8, 72076 Tübingen, Germany; 11 Cluster of Excellence iFIT (EXC 2180) “Image-Guided and Functionally Instructed Tumor Therapies”, University of Tübingen, 72076 Tübingen, Germany; 12 Tübingen Center for Academic Drug Discovery & Development (TüCAD2), 72076, Tübingen, Germany

**Keywords:** Computational biology and bioinformatics, Drug discovery, Anxiety

## Abstract

Anxiety is a troublesome symptom for many patients, especially those suffering from schizophrenia. Its regulation involves serotonin receptors, targeted e.g. by antipsychotics or psychedelics such as LSD. 5-HT_2A_ receptors are known for an extremely long LSD residence time, enabling minute doses to exert a long-lasting effect. In this work, we explore the changes in anxiety-like processes induced by the previously reported antipsychotic, D2AAK1. In vivo studies revealed that the effect of D2AAK1 on the anxiety is mediated through serotonin 5-HT_1A_ and 5-HT_2A_ receptors, and that it is time-dependent (anxiogenic after 30 min, anxiolytic after 60 min) and dose-dependent. The funnel metadynamics simulations suggest complicated ligand-5HT_2A_R interactions, involving an allosteric site located under the third extracellular loop, which is a possible explanation of the time-dependency. The binding of D2AAK1 at the allosteric site results in a broader opening of the extracellular receptor entry, possibly altering the binding kinetics of orthosteric ligands.

## Introduction

Anxiety belongs to the most prevalent psychiatric disorders^1,2^. It becomes even more common in the time of unrest, with factors such as the global COVID-19 pandemic or climate change taking its toll^3^. For instance, in the study of Hyland et al. during a nationwide quarantine in Ireland caused by the COVID-19-outbreak, 20% of respondents have shown symptoms of generalized anxiety disorder^4^. According to several studies, anxiety disorders may affect as much as 13.6% of the population of Europe, and comparable fraction of the USA society^5^. Anxiety can affect patients of any age, including children and elders, greatly affecting their quality of life and prevalence of comorbidities^6,7^. Moreover, anxiety symptoms have been reported in up to 65% of patients with schizophrenia, and may reach the diagnostic threshold in other disorders, such as depression^8^. The effect of antipsychotics on the anxiety processes is important to their pharmacological activity. Data from randomized and open trials have demonstrated that aripiprazole and risperidone could be efficient for diminishing obsessive–compulsive and social anxiety symptoms, while quetiapine and olanzapine can be helpful in generalized anxiety^9,10^.

Numerous neurotransmitter systems are involved in mediating anxiety processes. Among them, serotonin plays a key role. It is well-known that the stimulation of the pre-synaptic 5-HT_1A_ receptors leads to an anxiolytic effect due to suppression of the serotonin release^11^. The 5-HT_1A_ autoreceptor is thus a target for anxiolytic drugs, such as buspirone and fluoxetine^11^. The anxiolytic properties of antipsychotics, in particular second generation drugs, results partially from stimulation of 5-HT_1A_. This is exemplified by the atypical antipsychotics like clozapine, quetiapine and ziprasidone, which combine D_2_ receptor antagonism and 5-HT_1A_ agonism^12^. Furthermore, the 5-HT_2A_ receptor is also engaged in the development of anxiety, and its antagonists, including atypical antipsychotics, are useful in the anxiety treatment.

**Figure 1:**
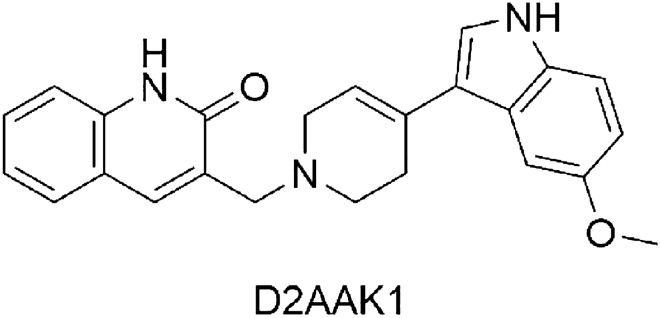
Structure of D2AAK1.

Previously, we identified a multi-target ligand of aminergic G protein-coupled receptors (GPCRs), D2AAK1 (see Fig. 1)^13^. This compound has nanomolar affinity to dopamine D_2_ receptor (K_i_ of 58 nM), serotonin 5-HT_1A_ receptor (K_i_ of 125 nM) and serotonin 5-HT_2A_ receptor (K_i_ of 358 nM)^13^. It is D_2_ and 5-HT_2A_ receptor antagonist and 5-HT_1A_ receptor partial agonist^14^. The compound displays antipsychotic, anxiolytic and procognitive activity in respective mice models^14^. Here we report a detailed investigation of the complex effect of D2AAK1 on anxiety processes and present the molecular-level explanation of the pharmacological data provided by the funnel metadynamics approach. The elevated plus-maze test (EPM) was used for measuring anxiety. This test is based on the hypothesis that exposure to an elevated and open maze alley leads to an approach–avoidance conflict that is considerably stronger that evoked by exposure to a closed maze alley^15^. Fear-induced inhibition of exploratory activity affects entries into open arms in this task. As a significant increase in the percentage of time spent on the open arms and the number of entries into open arms is observed only with drugs that are clinically effective anxiolytics^16^, this model has been used to assess anxiolytic-like activity of new putative anxiolytic.

## Results

### ADMET parameters

To evaluate the suitability of D2AAK1 for animal studies, its selected ADMET parameters were determined. The details are described in Supplementary Information. It was found that D2AAK1 displays moderate passive transport through biological membranes (Table S1). D2AAK1 presented beneficial active transport properties with no P-gp ATPase substrate properties, which is a critical factor that may affect the drug accumulation into brain (Fig. S1). D2AAK1 moderately inhibited CYP3A4 activity, however less than reference CYP3A4 inhibitor—ketoconazole (Fig. S2). Furthermore, D2AAK1 displayed no neurotoxic activity at neuroblastoma SH-SY5Y cells (Fig. S3).

### DOI synthesis

DOI, a necessary pharmacological tool for animal studies, was synthesized by a newly developed protocol (Scheme S1). The synthesis involves an effective way to reduce nitroalkenes to phenethylamines in a CuCl_2_–NaBH_4_ one-pot reaction. Details are presented in Supplementary Information.

### Behavioral studies

**Figure 2:**
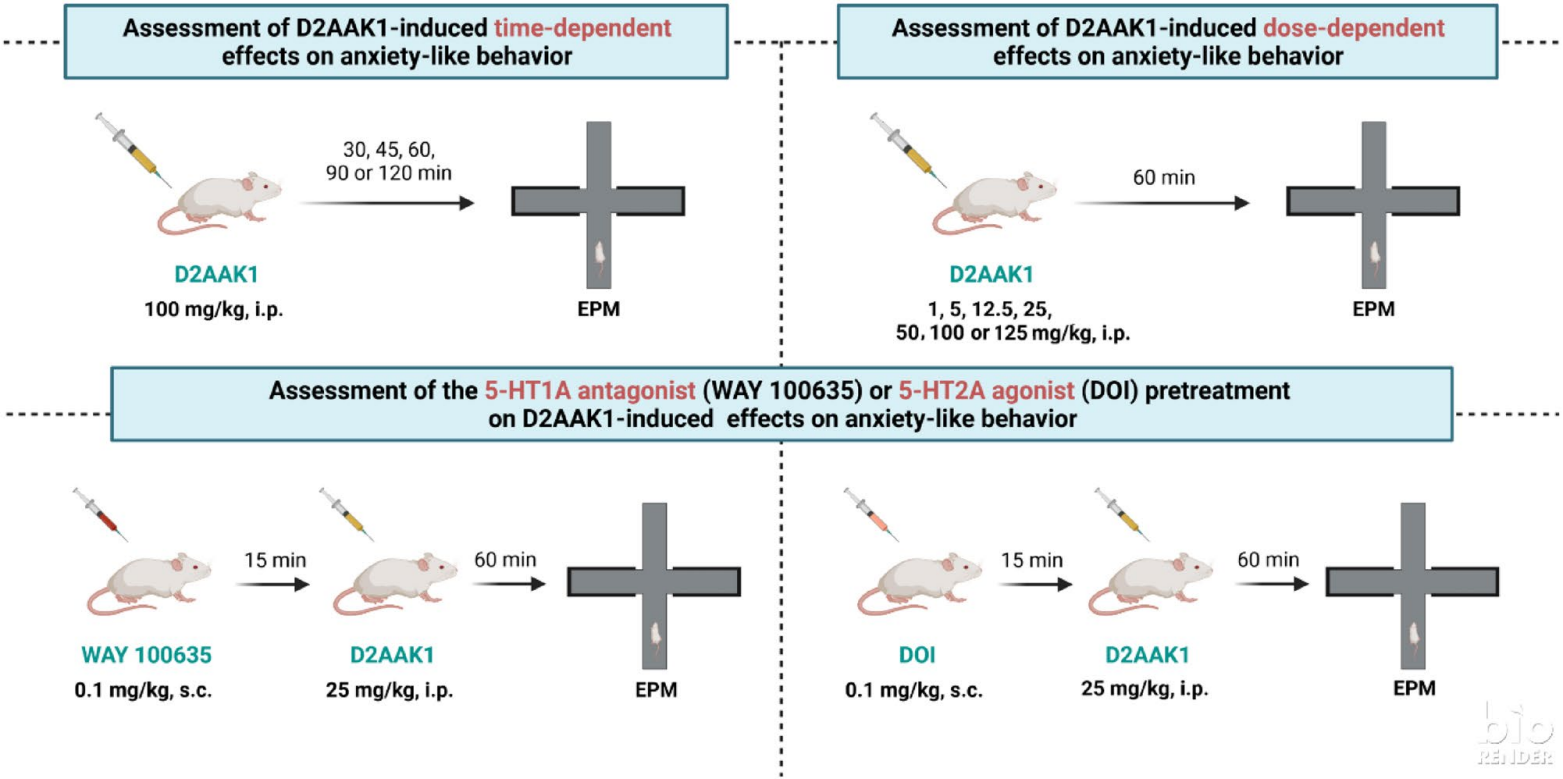
The scheme of behavioral experiments.

The scheme of behavioral experiments is shown in Fig. 2.

#### Effect on the anxiety-like behavior: time dependence

**Figure 3:**
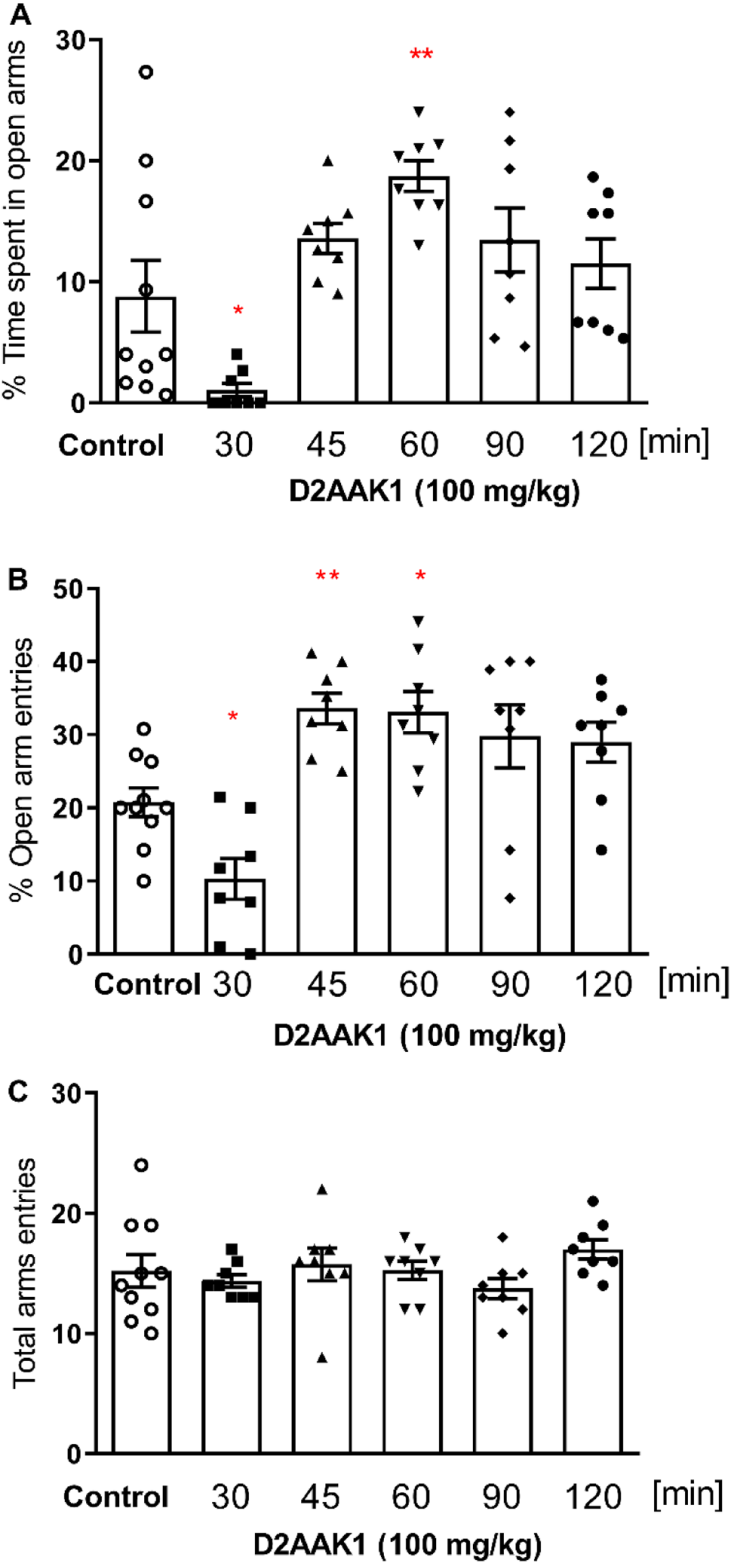
Time-dependence of D2AAK1 effect on the anxiety-like behavior. D2AAK1 (100 mg/kg) was tested on mice using elevated plus maze. The compound was injected i.p. 30, 45, 60, 90 and 120 min before the test. The values represent the mean ± SEM of percentage time spent on the open arms (**A**), percentage of entries into the open arms (**B**), and the locomotor activity of the animals, presented as the sum of entries into open and closed arms (**C**). **p < 0.01; *p < 0.05 versus control group (Bonferroni’s test).

D2AAK1 (100 mg/kg) effect on the anxiety-like behavior in mice was studied using elevated plus maze (EPM) test. One-way ANOVA showed significant changes in percentage of open arms time [F(5, 44) = 7.840, *p* < 0.001, Fig. 3A] and in the percentage of open arm entries, [F(5, 44) = 9.877, *p* < 0.001, Fig. 3B]. In addition, we determined mouse activity by counting the total number of entries to both arms (i.e. the tested compound did not show statistically significant changes when compared to the control group [F(5, 44) = 1.125, *p* = 0.3615, Fig. 3C). The results showed that the influence of D2AAK1 on anxiety-like behavior is time-dependent. Bonferroni’s post-hoc test confirmed that D2AAK1 significantly increased percentage of time spent in open arms 60 min (*p* < 0.01) after its administration and percentage of open arms entries 45 min and 60 min (at least *p* < 0.05) after its administration. Interestingly, D2AAK1 decreased both percentage of time spent in open arms as well as percentage of open arms entries (*p* < 0.05 for both) after 30 min, suggesting anxiogenic activity.

#### The influence on the elevated plus maze performance: dose dependence

**Figure 4:**
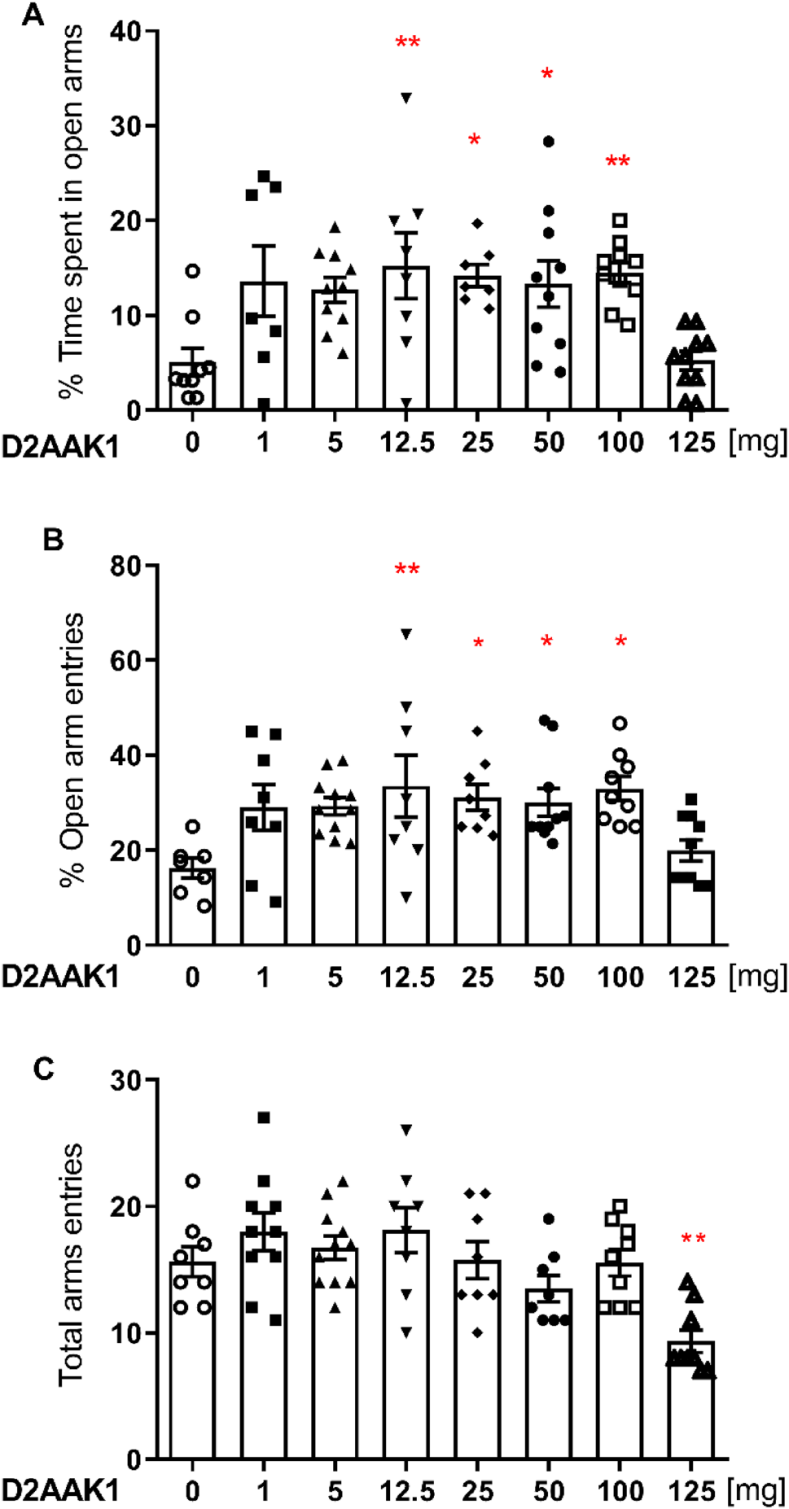
D2AAK1 (1–125 mg/kg) effect on anxiety-like behavior using elevated plus maze performance in mice. D2AAK1 was injected i.p. 60 min before the test. The values represent the mean ± SEM of percentage of time spent on the open arms (**A**), percentage of entries into the open arms (**B**), and the locomotor activity of the animals, presented as the sum of entries into open and closed arms (**C**). **p < 0.01; *p < 0.05 versus control group (Bonferroni’s post hoc test).

One-way ANOVA showed significant changes in percentage of time spent in open arms [F(7,63) = 4.185, *p* = 0.008, (Fig. 4A)], in the percentage of open arm entries [F(7,63) = 3.186, *p* = 0.0060, (Fig. 4B)] and in the total arm entries [F(7,63) = 5.228, *p* < 0.0001, (Fig. 4C)]. Bonferroni’s post hoc test confirmed that compound D2AAK1 significantly increased percentage of time spent in open arms and percentage of open arms entries at the doses of 12.5, 25, 50 and 100 mg/kg (at least *p* < 0.05), whereas only at the dose of 125 mg/kg it decreased the total number of entries to both arm types when compared to the control group (*p* < 0.01).

#### Effect of the pretreatment with WAY-100635, 5-HT_1A_ antagonist, on the anxiolytic activity in the EPM

**Figure 5:**
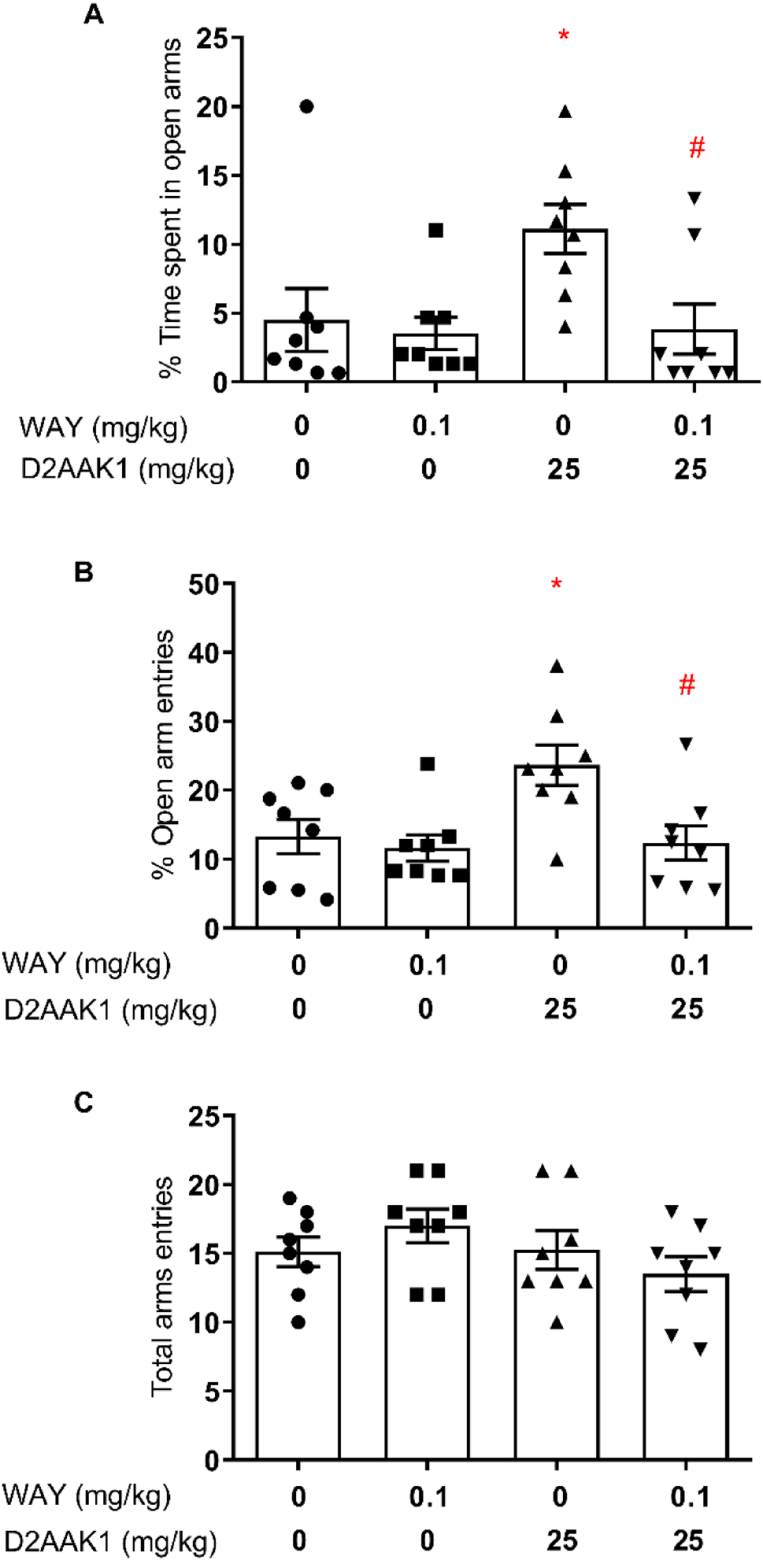
Anxiolytic activity of D2AAK1 is blocked by treatment with WAY in EPM test. Mice were pretreated with WAY 100635 (0.1 mg/kg s.c.), selective 5-HT_1A_ receptor antagonist 15 min before treatment with D2AAK1 (25 mg/kg, i.p.), which was injected 60 min before the test. The values represent the mean ± SEM of percentage of time spent on the open arms (**A**), percentage of entries into the open arms (**B**), and the locomotor activity of the animals, presented as the sum of entries into open and closed arms (**C**). *p < 0.05 versus control group, ^#^*p* < 0.05 versus D2AAK1 group (Bonferroni’s post hoc test).

A two-way ANOVA with repeated measures showed the statistically significant pretreatment effect (WAY): [F(1,28) = 5.235, *p* < 0.05] on anxiolytic activity of 25 mg/kg D2AAK1 (Fig. 5A). Bonferroni’s post hoc test revealed a significant increase in percentage of time spent in the open arms after administration of D2AAK1 (*p* < 0.05). Moreover, this effect was reduced by pretreatment with 0.1 mg/kg WAY in the group treated with D2AAK1 (*p* < 0.05). Two-way ANOVA with repeated measures showed statistically significant changes in the percentage of entries into the open arms {pretreatment effect (WAY): [F(1,28) = 6.709, *p* < 0.05] and treatment effect (D2AAK1): [F(1,28) = 5.004, *p* < 0.05], (Fig. 5B)}. Bonferroni’s post hoc test indicated statistically significant increase in percentage of entries into the open arms (*p* < 0.05) for D2AAK1-treated group. Moreover, this effect was diminished when mice were pretreated with WAY (*p* < 0.05). All tested compounds did not show statistically significant changes in the total number of entries to the both arms of EPM {two-way ANOVA, pretreatment effect (WAY): [F(1,28) = 0.002512, *p* = 0.9604]; treatment effect (D2AAK1): [F(1,28) = 1.831, *p* = 0.1868] and ANOVA interaction effect between pretreatment and treatment: [F1,28) = 2.112, *p* = 0.1572]}, Fig. 5C.

#### Effect of pretreatment with DOI on the anxiolytic activity in the EPM

**Figure 6:**
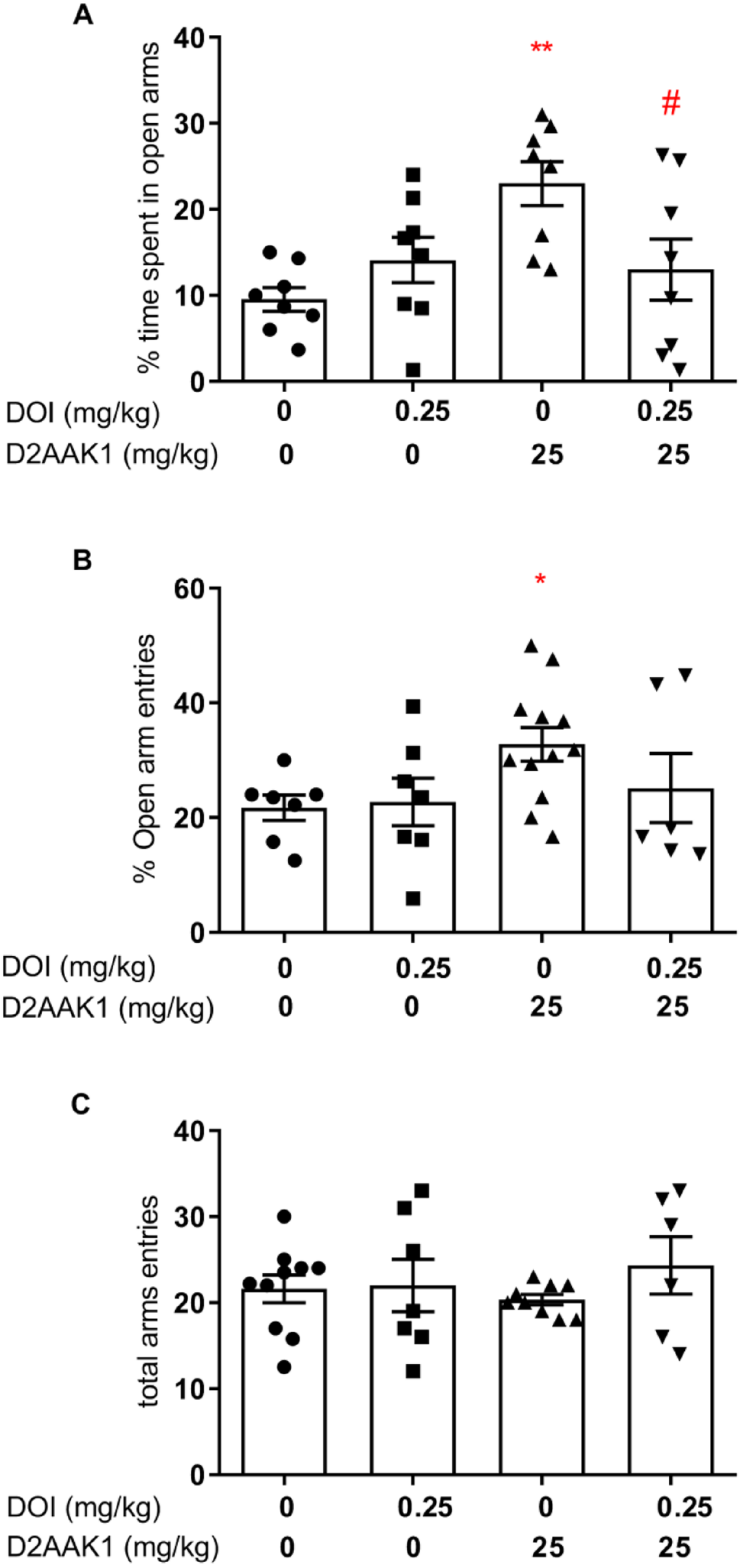
Effect of DOI, a 5-HT_2A_, 5-HT_2B_ and 5-HT_2C_ receptor agonist, on the anxiolytic-like activity of D2AAK1 in EPM test. Mice were pretreated with DOI (0.25 mg/kg s.c.), 15 min before administration of D2AAK1 (25 mg/kg, i.p.), that was injected 60 min before the test. The values represent the mean ± SEM of percentage time spent in the open arms (**A**), percentage of entries into the open arms (**B**), and the locomotor activity of the animals, presented as the sum of entries into open and closed arms (**C**). *p < 0.05, **p < 0.01 versus control group, ^#^*p* < 0.05 versus D2AAK1 group (Bonferroni’s post hoc test).

Two-way ANOVA showed the statistically significant treatment effect (D2AAK1): [F(1,29) = 4.774, *p* = 0.0371] and ANOVA interactions between pretreatment and treatment [F(1,29) = 5.715, *p* = 0.0235] on anxiolytic activity of 25 mg/kg D2AAK1 (Fig. 6A). Bonferroni’s post hoc test revealed a significant increase in percentage of time spent in the open arms of EPM after administration of D2AAK1 (*p* = 0.0123). Moreover, the D2AAK1 effect was reduced by pretreatment with DOI (*p* = 0.0363). Two-way ANOVA showed statistically significant changes in the percentage of open arms entries {treatment effect (D2AAK1): F(1,29) = 4.421, *p* = 0.0443], (Fig. 6B)}. Bonferroni’s post hoc test indicated lack of statistically significant increase in percentage of open arms entries (*p* > 0.05). The tested compound did not show statistically significant changes in the total number of entries to both arms when compared to the control group {two-way ANOVA: pretreatment (DOI): [F(1,29) = 2.638, *p* = 0.1152]; treatment (D2AAK1): [F(1,29) = 0.0468, *p* = 0.8303] and ANOVA interaction effect between pretreatment x treatment: [F(1,29) = 1.651, *p* = 0.2090]}, Fig. 6C.

### Funnel metadynamics simulations

**Figure 7:**
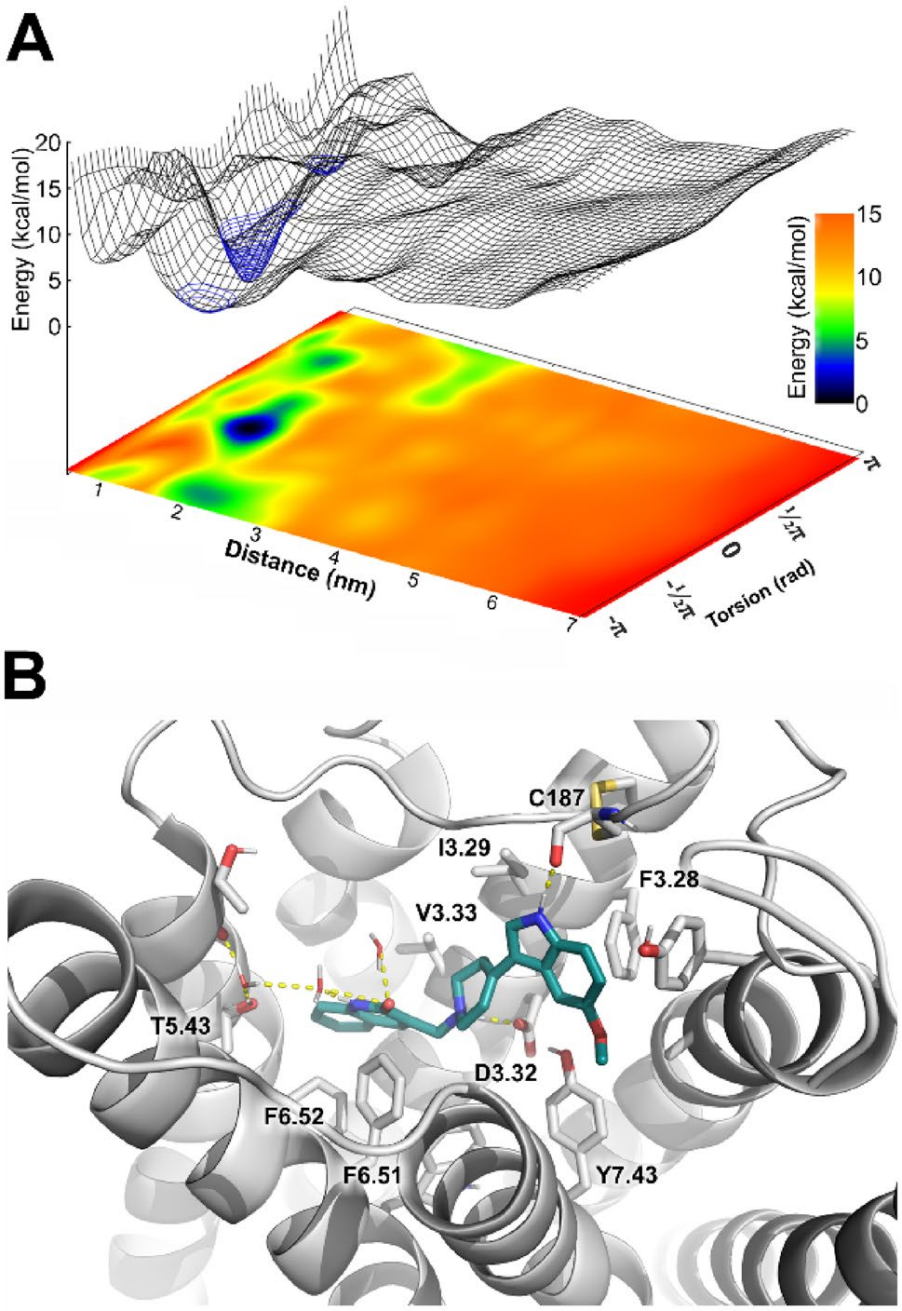
Results of the FM of D2AAK1 binding to the 5-HT_1A_ receptor. (**A**) A FES revealed one well-defined binding site. The apparent free energy basin, additionally marked with a blue mesh, corresponds to the orthosteric pocket of 5-HT_1A_ receptor. (**B**) The binding pose coresponding to the free energy minimum.

**Figure 8:**
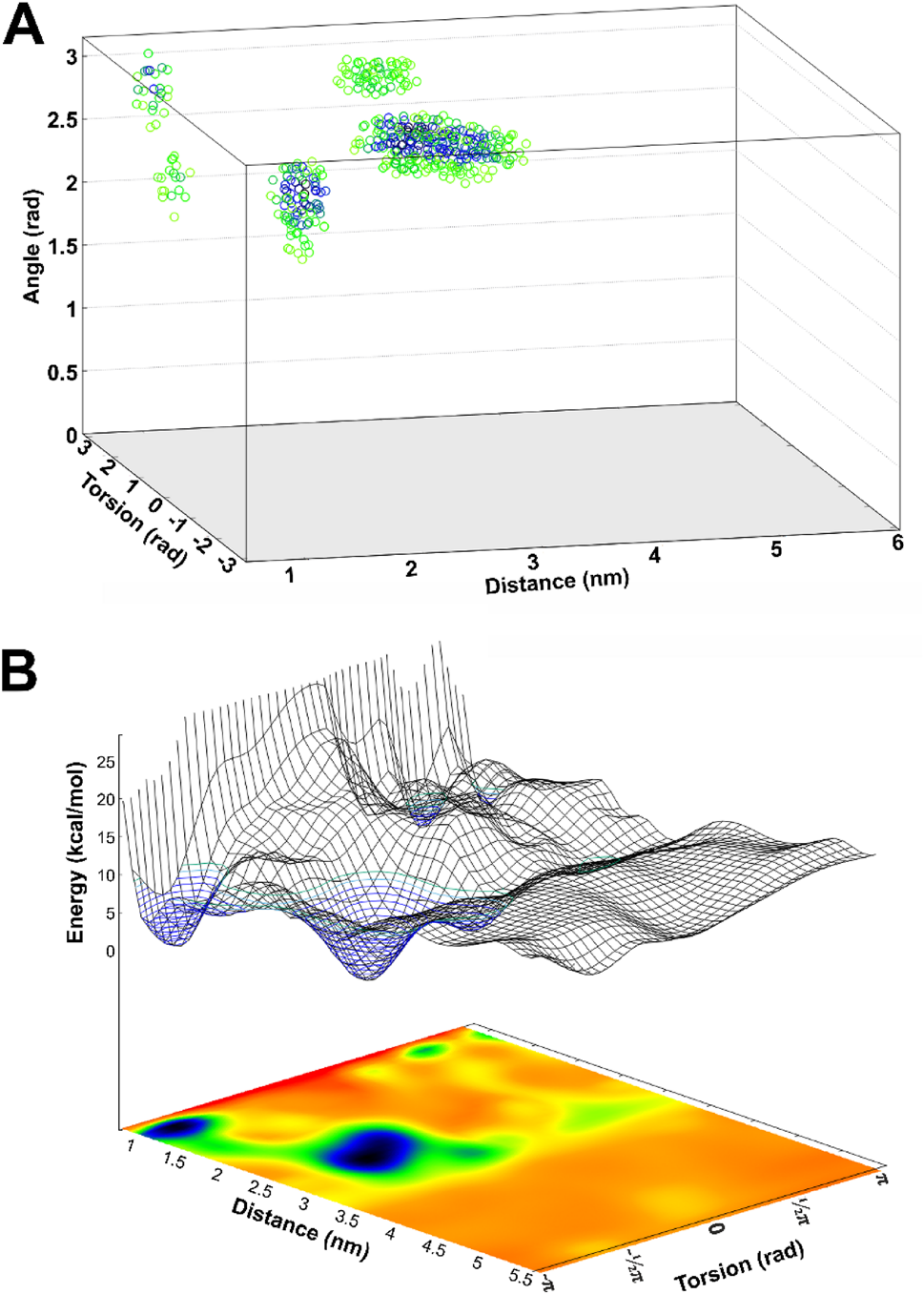
Free energy landscapes in FM simulations of D2AAK1 binding to the 5-HT_2A_ receptor. (**A**) A four-dimensional representation shows that all the relevant free energy basins correspond to angle values above 2.5 rad, enabling efficient FES representation by projections on a surface. (**B**) The three-dimensional representation reveals two deep basins. The free energy minima are additionally marked with a blue mesh.

The kinetics of D2AAK1 interactions with 5-HT_1A_ and 5-HT_2A_ receptors were investigated with funnel metadynamics (FM)^17^, following the protocol suggested by Raniolo and Limongelli. Notably, in all the relevant free energy basins in both receptors the angle CV remains close to π rad, enabling plausible representation of the free energy surfaces (FES’s) in two-dimensional projections on distance and torsion CVs (Figs. 7 and 8). The ligand binding with 5-HT_1A_ receptor resulted in a binding FES bearing no surprises, with a binding mode comparable to that obtained with a regular docking (Fig. 7B).

The indole moiety, which could be seen as analogous to that of serotonin, interacts with ECL2, engaging in a hydrogen bond with C187 and T-stacking π–π interaction with F3.28, while the quinolinone moiety dives deeper into the receptor interior, involving edge-to-face interaction with F6.52 and water-mediated polar interactions with T5.43, with possibility of employing S5.42 to similar interactions (Fig. 7). The binding free energy was calculated to be − 8.986 ± 0.713 kcal/mol.

**Figure 9:**
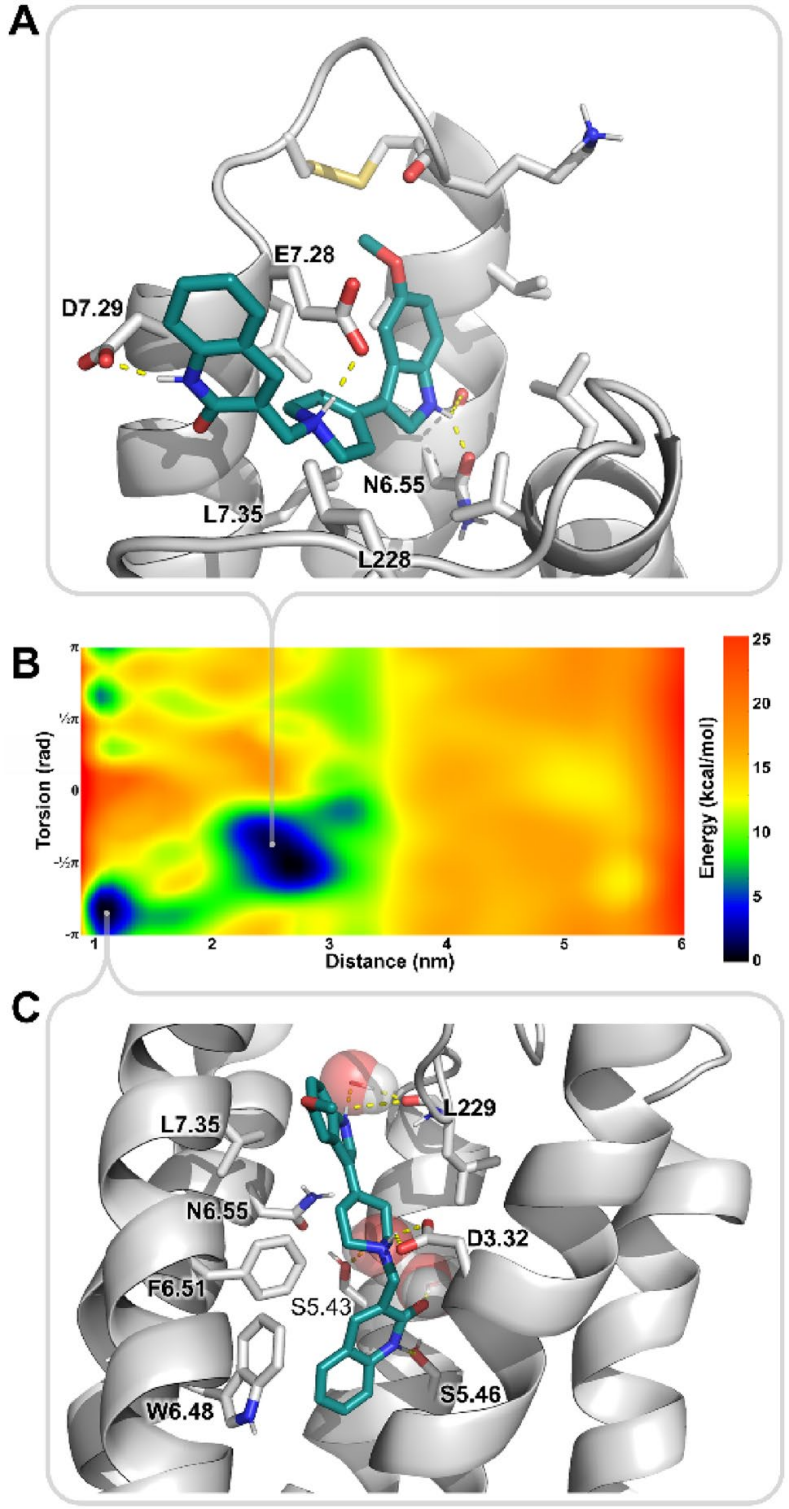
D2AAK1 binding sites identified in 5-HT_2A_ receptor. (**A**) The allosteric site is located at the top of TM6 and TM7 and involves several polar interactions with aspartate and glutamate residues, as well as non-polar interactions and notable shape complementarity of the methoxyindole moiety and a small pocket under ECL3. (**B**) FES of D2AAK1 binding to 5HT_2A_R with its two apparent basins: one at the distance of ca. 2.5 nm, corresponding to the pocket shown in the panel (**A**), and the one at the distance of ca. 1.1 nm, corresponding to the orthosteric binding as shown in the panel (**C**). (**C**) D2AAK1 binds in the orthosteric pocket of 5HT_2A_R in a manner resembling methiothepin, as seen in the 6WH4 PDB structure, preventing W6.48 motility. Simulations in explicit solvent enabled identification of water-mediated interactions with S5.43.

A possible explanation of at least a part of complicated anxiogenic/anxiolytic properties of the compound was provided by the simulations of 5-HT_2A_ receptor. FM-derived FES clearly shows two separate, deep free energy basins corresponding to the orthosteric site and an additional, allosteric site located at the distance corresponding to ECL2–ECL3 level (Figs. 8 and 9). Importantly, the simulation convergence plots, provided in Fig. S4, show that the allosteric basin is stable.

The orthosteric binding mode is similar to that of methiothepin as seen in the X-ray structure (PDB ID: 6WH4), in that D2AAK1 establishes an edge-to-face π–π interaction with the conserved W6.48, locking it in the conformation previously described as proper to inactive-state receptor^18^. The position of the deeply bound quinolinone moiety is further stabilized by H-boding with S5.46 and water-mediated interaction with S5.43. Meanwhile, an allosteric binding site was found at the top of TM6 and TM7, where shape complementarity makes the methoxyindole moiety fit into pocket formed by ECL3 and its cysteine bridge, and its position is further stabilized by the indole H-bonding with N6.55 (Fig. 9, Fig. S5). The molecule is held in the site also by polar interactions between its protonated nitrogen and E7.28, between its lactam nitrogen atom and D7.29, as well as non-polar interactions with several residues, including L7.35 and L228 (ECL2). Simulation frames with ligand bound in this site are characterized by an increased average distance between ECL3 and ECL2 residues forming the ‘lid’^19^, located on the N-side of the C227 residue, as compared to the same distance measured in frames with D2AAK1 bound to the orthosteric site, 1.681 ± 0.156 nm versus 1.499 ± 0.151 nm, respectively. Binding free energies calculated for both orthosteric and allosteric sites were comparable, with values of − 13.38 ± 1.55 kcal/mol and − 14.63 ± 0.690 kcal/mol, respectively.

To verify the calculated free energy values, additional unbiased MD simulations were performed, followed by end-state free energy calculations with gmx_MMPBSA^20,21^. These calculations evaluate the binding free energy to the allosteric site at 5-HT_2A_ receptor to be of − 28.78 ± 3.84 kcal/mol, and at its orthosteric site to − 45.59 ± 3.79 kcal/mol. The same method applied to the binding mode at the orthosteric site of 5-HT_1A_ receptor yielded a value of − 35.56 ± 3.17 kcal/mol (Table 1). The differences between value ranges obtained by the two methods result from the principles behind those methods—results of the FM method are usually closer to the experimental values^22^, while MM-PBSA and MM-GBSA results are often overestimated due to used approximations^23,24^. Nevertheless, the end-state calculations confirm stable binding of D2AAK1 in the investigated pockets. The in silico investigation therefore indicates complexity of the D2AAK1 binding kinetics at 5-HT_2A_R, originating in the presence of an allosteric/metastable binding site.

**Table 1:** Summary of the calculated binding free energies of D2AAK1, compared to the experimentally obtained Ki values.

Binding site	Binding free energy (kcal/mol)		Experimental Ki (nm)^13^
	FM	MM-GBSA	
5-HT_1A_R	− 8.986 ± 0.71	− 35.56 ± 3.17	125 ± 8
5-HT_2A_R orthosteric	− 13.38 ± 1.55	− 45.59 ± 3.79	358 ± 213
5-HT_2A_R allosteric	− 14.63 ± 0.69	− 28.78 ± 3.84	–

## Discussion

Treatment of anxiety, due to its prevalence in the modern society, is an urgent problem. It is also a key component of schizophrenia symptoms management. Atypical antipsychotics display anxiolytic activity based on 5-HT_2A_ receptor antagonism and 5-HT_1A_ receptor agonism or partial agonism. D2AAK1^13,14^ has a complex effect on the anxiety processes, similarly as a previously reported compound D2AAK3^25^. Administration of D2AAK3 results in anxiogenic properties after 30 min and lack of the influence on anxiety-like behavior after 60 min. Detailed investigations of the effects of potential antipsychotics on the anxiety processes are necessary, as favorable multi-receptor profile may display anxiogenic properties, as seen in another compound reported by our group, D2AAK4^26^.

Behavioral studies indicated time-dependent and dose-dependent effect of D2AAK1 on the anxiety-like processes. The D2AAK1 effect was investigated 30, 45, 60, 90 and 120 min after drug administration. The compound induced anxiogenic activity 30 min after administration, while anxiolytic properties were found 60 min after treatment, creating an inverted U-shaped dose–effect curve (IUSDEC). The IUSDEC effect is a frequently observed phenomenon in behavioral-pharmacological studies^27^ and several possible explanations of this outcome may be suggested. Firstly, as an anxiolytic effect of D2AAK1 develops in time, we may hypothesize that in the shortest treatment-testing interval (30 min), some non-specific changes in activation of various receptors might have occurred, leading to an anxiogenic effect. Although the serotoninergic receptors are the key candidates suspected of D2AAK1-related anxiety-like behaviors, this compound is a novel multi-target ligand and existence of off-targets cannot be excluded. Secondly, the results indicate that the tolerance to D2AAK1-induced anxiogenic effect develops over time and this effect is dose-dependently reversed into anxiolytic outturn. Finally, the observed IUSDEC effect may be related to receptors desensitization theory^28^, which suggest that over time some receptors may display decreased sensitivity to the administered compound. In the light of our findings, we may hypothesize that for D2AAK1-induced anxiolytic effects part of the receptors (most possibly 5-HT_1A_ receptors) should be desensitized over time. Importantly, similar IUSDEC anxiety-related have been previously reported in mice treated with Carica papaya pulp extract^29^. Furthermore, an anxiolytic IUSDEC effect was also observed in humans where subjective anxiety measures were reduced with the middle, but not the lowest and highest of the administered cannabidiol doses^30^.

Considering all investigated doses of D2AAK1, the compound produced an anxiolytic effect (perceived as an increase in percentage of time spent in open arms and percentage of open arms entries) at the doses of 12.5, 25, 50 and 100 mg/kg.

Importantly, the anxiolytic activity of D2AAK1 was blocked by the pretreatment with WAY-100635 (selective 5-HT_1A_ receptor antagonist) and DOI (a 5-HT_2A_, 5-HT_2B_ and 5-HT_2C_ receptor agonist). The observed effect suggests that the anxiolytic effect of D2AAK1 is mediated through both, 5-HT_2A_ and 5-HT_1A,_ subtypes of serotonergic receptors. This is in agreement with literature data about atypical antipsychotics, including that at clinically effective doses induced extensive blockade of 5-HT_2A_ receptors as well as potentiation of 5-HT_1A_ receptors^31^.

Altogether, the observed D2AAK1 behavioral effects suggest promising profile in terms of alleviating anxiety-like symptoms frequently observed in schizophrenic patients.

FM simulations were applied to seek for possible explanations of the time- and dose-dependence of the D2AAK1 anxiolytic effects in its interactions with its main anxiety-related targets, i.e. 5-HT_1A_R and 5-HT_2A_R. The method was previously shown to properly identify primary and alternative binding modes, and provide accurate estimates of ligand binding free energy values^17,22^. The results strongly suggest that time and dose dependence may be related to binding kinetics at 5-HT_2A_R. Simultaneous excitation of 5-HT_1A_R and blockade of 5-HT_2A_R, related to the anxiolytic properties, seems to be delayed by the late onset of the latter. The 5-HT_2A_ receptor is known for its long-lasting binding interactions with LSD, with the ligand residence time reaching tens of minutes, which can be attributed to the presence of an extracellular ‘lid’, formed by the ECL2 residues, closing the ligand in the orthosteric site^19^. Mutation of L229 ECL2 residue greatly affects motility of the ‘lid’, significantly shortening the residence time of LSD and effect of the incubation time on its efficacy^19^. The ‘lid’ in the wild-type protein tightly closes the entrance to the orthosteric site of 5-HT_2A_ receptor from the extracellular milieu for significant part of the time, limiting ligand entrance episodes, which is altered by L229A mutation^19^. D2AAK1, bound in its allosteric site with significant binding free energy, interacts with L228—a residue immediately preceding L229 in the receptor sequence—and loosens ECL2-ECL3 interactions (average distance between loops increased from 1.499 ± 0.151 nm in orthosteric D2AAK1 binding to 1.681 ± 0.156 nm in allosteric binding), leaving the orthosteric site more accessible to the extracellular ligands. Therefore, the initial anxiogenic response may be a result of an initial increase of endogenous serotonin binding by the receptor, before D2AAK1 leaves the allosteric site and enters deeper to the orthosteric site. The presence of additional binding site and slow binding kinetics would also explain large standard error of the mean of experimental K_i_ values, as well as K_i_ being decreased at 5-HT_1A_ receptor despite the less favorable binding free energy values^13^. Both sites seem to be connected by a curved saddle, indicating that the initial binding at the allosteric site may be an event preceding the ligand entrance to the receptor interior.

The presence of an allosteric site, encompassing small, indole-binding pocket in a serotonin receptor raises questions about generality of the observed results. Notably, in the seminal work of Dror et al.^32^ the authors investigate ligand binding pathways in β_1_ and β_2_ adrenergic receptors, the ligands tend to remain for a significant time in a metastable binding pocket, located in the nearness of ECL3, engaging salt bridges of their protonated amines with aspartate residue in ECL3. Also in our simulations of D2AAK1 binding to the 5-HT_1A_ receptor there is a shallow basin corresponding to similar location of the ligand, although describing a much less defined set of ligand conformations and much shallower well of potential. Therefore, it can be concluded that the D2AAK1 binding occurs along the more general pathway appropriate for aminergic GPCRs, initially binding at the metastable sites found across the subfamily, and it happens to find particularly comfortable fit at the 5-HT_2A_R, possibly due to abundance of negatively charged aspartate and glutamate residues in the area together with the number of positively or partially positively charged moieties in the ligand, developing strong interactions and interacting allosterically from there.

## Conclusions

The effect of D2AAK1 on the anxiety-like processes in mice is dose-dependent and time-dependent. Furthermore, it was shown that this effect is mediated through serotonin 5-HT_1A_ and 5-HT_2A_ receptors. The funnel metadynamics simulations suggest that one possible explanation of the observed phenomena is the complex way of D2AAK1-5-HT_2A_R interactions, with one regular orthosteric site and one additional allosteric site located at the top of TMVI and TMVII, under ECL3, analogically to metastable binding sites found by Dror et al. in β_1_ and β_2_ adrenergic receptors. In particular, the binding of D2AAK1 at the allosteric site of 5-HT_2A_R with relatively favorable binding free energy involves residue neighboring L229, known to affect motility of ECL2, and results in increased distance between ECL2 and ECL3, possibly altering binding kinetics of orthosteric ligands.

## Experimental section

### Behavioral studies

#### Animals

The experiments were carried out, in the Experimental Medicine Center of Medical University of Lublin, on six week old naive male Swiss mice, weighing 24–30 g. The mice were housed in cages, 4 individuals per cage in an environmentally controlled rooms (ambient temperature 22 ± 1 °C; relative humidity 50–60%; 12:12 light:dark cycle, lights on at 8:00). Standard laboratory food (LSM, Agropol-Motycz, Poland) and filtered water were available ad libitum except for the short time that they were removed from their cages for testing. All the experimental procedures were carried out in the light phase, between 09.00 a.m. and 3.00 p.m, mice were randomly assigned to different treatment groups, and the experimenter was blinded to the allocation when conducting the EPM experiments. Rights and permission of the Local Ethical Committee on Animal Testing (permission number 147/2018) were obtained and all procedures were compliant with the ARRIVE guidelines (https://arriveguidelines.org).

#### Drugs

After a short time period of animal adaptation to the room in which the experiment was conducted, the compound D2AAK1 (synthesized in house according to previously reported procedure^33^) was administered. It was dissolved in dimethyl sulfoxide DMSO (to a final concentration of 0.1%) with a drop of 0.2% Tween 80 and then diluted by aqueous solution of 0.5% methylcellulose and injected intraperitoneally (i.p.) respective time before the tests. The following tool substances were used as follows: WAY 100635, and DOI (synthesized in house, (±)-2,5-dimethoxy-4-iodoamphetamine hydrochloride). All were dissolved or diluted in 0.9% tylose containing 0.2% Tween 80 prior to use. To establish the involvement of the serotonergic-mediated mechanism in the anti-anxiety effect D2AAK1 (at the dose of 25 mg/kg, i.p.) in EPM, the animals were pretreated with WAY 100635 (a selective 5-HT_1A_ receptor antagonist) 0.1 mg/kg subcutaneously (s.c.), or DOI (a 5-HT_2A_, 5-HT_2B_ and 5-HT_2C_ receptor agonist) 0.25 mg/kg, i.p., 15 min before the compound. Doses and pretreatment times were selected on the basis of literature data and previous experiments in our laboratory^34^. All compounds were administered in a manner generally accepted in experimental pharmacology, in an amount of 10 mL/kg body weight. The animals were weighed immediately before injection. Each study group consisted of 8–10 individuals. The control group received an equivalent volume of tylose in an adequate time before testing. Between the injections, mice were provided with stable living conditions and unrestricted access to food and water.

#### Elevated plus maze (EPM) procedure

The EPM studies were carried out on mice according to the method of Lister^35^. The EPM apparatus was made of plexiglas and consisted of four crossed arms forming a plus sign, raised 38.5 cm above the floor, and illuminated by a weak red light. Two arms were open (30 × 5 cm) and two closed (30 × 5 × 15 cm). The arms extended from a central platform of 5 × 5 cm. The mice were individually placed at the central square of the plus-maze apparatus, facing the open arm, and their behavior was observed for 5 min (using a stopwatch totalizer). The test arena was wiped with a damp cloth after each trial. The number of entries and the time spent in the open and closed arms were measured by an observer blind to drug treatment. The entry into one arm was defined as the stage when the animal placed all its four paws past the line that divided the central square from the open arms. Anxiolytic activity was defined as an increase of the time spent in the open arms or/and in the number of entries into the open arms and was calculated as percentage of time spent and percentage of entries into the open arms. Additionally, the number of open and closed arm entries was recorded as the indicator of motor activity of tested animals.

Different groups of mice were used to evaluate both the time-dependent as well as dose-dependent effects on anxiety-like behavior in the EPM test. In case of control group for time dependent testing the measurement was done on time point 60 min only due to 3R principle. Similarly, experiments with WAY 100635 and DOI were carried out on different groups of animals.

#### Statistical analysis

Statistical analysis was carried out using GraphPad Prism ver. 5.0. All results are presented as means ± standard errors of means (SEM) for in vivo research. Differences between control and tested D2AAK1 were assessed with one-way analysis of variance (ANOVA) with Bonferroni’s post-hoc test. To evaluate the effects of treatment (D2AAK1), pretreatment (WAY or DOI) or interaction between these two factors, the two-way ANOVA was used followed by Bonferroni’s post hoc test to compare each group to the other. The confidence limit of *p* < 0.05 was considered statistically significant.

### In silico studies

#### Molecular modeling and simulation setup

The structure of D2AAK1 was built with Spartan 10.1^36^ and optimized using DFT B3LYP, 6-31G* basis set. The restrained electrostatic potential atomic partial charges were derived using the RESP ESP Server^37^ and used to prepare ligand topology with ACPYPE^38^. The 5-HT_2A_ receptor structure was obtained from the Protein Data Bank (PDB ID: 6a94)^39^. Since the study began before the cryo-EM structures of 5-HT_1A_ receptor were published, the structure was obtained through homology modeling with MODELLER^40^, using 5-HT_1B_ X-ray structure as a template (PDB ID: 4iar)^41^. 500 models were prepared and assessed with the DOPE scoring function and the Molprobity server^42^. Subsequently, 1 μs molecular dynamics simulation (MD) of the model with ergotamine docked to the orthosteric site were conducted. Amber03 force field was used for protein, Slipids force field^43^ for the membrane. Simulation boxes were built with CHARMM-GUI server^44^. Receptor orientations in membranes were gathered from the OPM database^45^.

#### Funnel metadynamics and MM-GBSA calculations

The multiple walker, well-tempered FM^22^ simulations were performed with Gromacs^46^ patched with Plumed 2.7^47^. Metadynamics runs were carried out in the NVT ensemble in the temperature of 309.75 K. Twenty walkers were used in each simulation. Three CVs were used: a distance between the Ser3.38 residue in 5-HT_1A_ receptor or the Ser3.39 residue in the 5-HT_2A_ receptor and the protonated nitrogen of the ligand, a torsion, and internal ligand angle measured between arms defined by the distal aromatic rings and the central aliphatic ring used as a vertex. Although simpler setups were reported to be successful in enhanced sampling simulations of ligand binding to GPCRs^48,49^, these studies concerned mostly smaller and more rigid, purely orthosteric ligands, which facilitated sampling of conformational space. In our hands, free energy landscapes produced in vanguard simulations of D2AAK1 using only distance and dihedral CV were not reproducible in independent simulations. Internal ligand angle turned out to be a hidden variable^50^, as additional potentials frequently enforced the bent conformation of the ligand involving internal π–π stacking upon its entrance to the receptor, and its relaxation within the binding site was a slow process of the system. Introducing the ligand angle CV resulted in reproducibility of the obtained FESs. Gaussian widths used were 0.3 nm, 0.4 rad and 0.4 rad, respectively. Initial Gaussian height was set at 0.3 kJ/mol, with a deposition timestep of one picosecond. The Gaussian height gradually decreased due to application of the adaptive bias with a ΔT = 36,771 K in 5-HT_2A_ receptor simulations and 46,041 K in 5-HT_1A_ simulations. 20 walker simulations were run for ca. 400 ns, resulting in total of ca. 8 µs of simulations per receptor. Simulation frames corresponding to the free energy minima were extracted from all walkers and clustered by single linkage method with cutoff value = 0.05 nm. Central structure of the largest cluster was selected for each of the basins, minimized and used in unbiased 200 ns MD in the NPT ensemble to check stability of the pose and provide trajectories for MM-GBSA calculations. End-state free energy calculations were performed with gmx_MMPBSA v1.4.3^20,21^. The FM-derived free energy values were calculated as in original paper of Limongelli et al.^17^ using equations:

$$\Delta {G}_{b}^{0}=-\frac{1}{\beta }ln\left({C}^{0}{K}_{b}\right),$$

and:

$${K}_{b}=\pi {R}_{cyl}^{2}\underset{{z}_{min}}{\overset{{z}_{max}}{\int }}dz {e}^{-\beta W\left(z\right),}$$

where $\beta =\frac{1}{{k}_{B}T}$, *k*_*B*_ is the Boltzmann constant, *C*^*0*^ is the standard concentration of 1/1660 Å^−3^, *K*_*b*_ is the binding constant, *πR*^*2*^_*cyl*_ is the surface of the cylinder at the top of the restraining funnel potential, and *W* is the potential of mean force (PMF). Free energy surfaces were plotted with Gnuplot^51^. Protein–ligand complexes were visualized using PyMol^52^.

Selected raw molecular modeling data is included in Supporting Information.

## Supplementary Information

Supplementary Information.

## Data Availability

All data generated or analyzed during this study are included in this published article and its supplementary information file.

## Abbreviations

5-HT_1A_R: Serotonin 5-HT_1A_ receptor
5-HT_2A_R: Serotonin 5-HT_2A_ receptor
CV: Collective variable
D_2_ receptor: Dopamine D_2_ receptor
DOI: 1-(4-Iodo-2,5-dimethoxyphenyl)propan-2-amine
EPM: Elevated plus-maze
FES: Free energy surface
FM: Funnel metadynamics
GPCRs: G protein-coupled receptors
IUSDEC: Inverted U-shaped dose–effect curve
MM-GBSA: Molecular mechanics with generalized Born and surface area solvation
PMF: Potential of mean force
WAY 100635: (*N*-[2-[4-(2-Methoxyphenyl)-1-piperazinyl]ethyl]-*N*-2-pyridinylcyclohexanecarboxamide)
