# Supplementary material for: Funnel metadynamics and behavioral studies reveal complex effect of D2AAK1 ligand on anxiety-like processes
Source: Sci Rep. 2022 Dec 7;12:21192. doi: 10.1038/s41598-022-25478-7 (PMC9729218; doi:10.1038/s41598-022-25478-7)
Supplement: Supplementary file 1 — Supplementary Information. [file 41598_2022_25478_MOESM1_ESM.docx]

Supplementary Information

**Funnel metadynamics and behavioral studies reveal complex effect of D2AAK1 ligand on anxiety-like processes**

Damian Bartuzi^1,2^, Ewa Kędzierska^3^, Katarzyna M. Targowska-Duda^4^, Oliwia Koszła^1^, Tomasz M. Wróbel^1^, Simon Jademyr^5^, Tadeusz Karcz^6^, Katarzyna Szczepańska^6^, Piotr Stępnicki^1^, Olga Wronikowska-Denysiuk^7^, Grażyna Biała^3^, Jadwiga Handzlik^6^, Jesper L. Kristensen^5^, Antti Poso^8,9,10,11,12^, Agnieszka A. Kaczor^1,8*^

^1^Department of Synthesis and Chemical Technology of Pharmaceutical Substances with Computer Modeling Laboratory, Faculty of Pharmacy, Medical University of Lublin, 4A Chodźki St., PL-20093 Lublin, Poland

^2^Science for Life Laboratory, Department of Cell and Molecular Biology, Uppsala University, SE-75124 Uppsala, Sweden

^3^Department of Pharmacology and Pharmacodynamics, Faculty of Pharmacy, Medical University of Lublin, 4A Chodźki St., PL-20093 Lublin, Poland

^4^Department of Biopharmacy, Faculty of Pharmacy, Medical University of Lublin, 4A Chodźki St., PL-20093 Lublin, Poland

^5^Department of Drug Design and Pharmacology, Faculty of Health and Medical Sciences, University of Copenhagen, Universitetsparken 2, 2100 Copenhagen, Denmark

^6^Department of Technology and Biotechnology of Drugs, Faculty of Pharmacy, Jagiellonian University, Medical College, Medyczna 9, PL-30-688 Kraków, Poland

^7^Independent Laboratory of Behavioral Studies, Chair of Biomedical Sciences, Faculty of Biomedicine, Medical University of Lublin, 4A Chodźki St., PL-20093 Lublin, Poland

^8^School of Pharmacy, University of Eastern Finland, Yliopistonranta 1, P.O. Box 1627, FI-70211 Kuopio, Finland

^9^University Hospital Tübingen Department of Internal Medicine VIII, Otfried-Müller-Strasse 14, DE-72076 Tübingen, Germany

^10^Department of Pharmaceutical and Medicinal Chemistry, Institute of Pharmaceutical Sciences, Eberhard-Karls-Universität, Auf der Morgenstelle 8, DE-72076 Tübingen, Germany

^11^Cluster of Excellence iFIT (EXC 2180) “Image-Guided and Functionally Instructed Tumor Therapies”, University of Tübingen, DE-72076 Tübingen, Germany

^12^Tübingen Center for Academic Drug Discovery & Development (TüCAD2), DE-72076 Tübingen, Germany

E-mail: agnieszka.kaczor@umlub.pl

**ADMET parameters**

**Permeability profile.** The ability of compound D2AAK1 to penetrate across lipid membranes was estimated by parallel artificial membrane permeability assay (PAMPA). In medicinal chemistry, PAMPA is a method that determines the passive diffusion of substances from a donor into acceptor compartment, through a lipid-infused artificial membrane; it is the predominant absorption mechanism for passive diffusion of most commercial drugs.^1^ By using the UPLC-MS spectrometry with an internal standard the exact quantity of molecules that penetrated from donor to acceptor wells through phospholipid membrane was estimated. The results were expressed as permeability coefficient *Pe* calculated according to the formulas described in the literature.^2^ Considering gathered data and comparing it to two standards: well-permeable Caffeine (*Pe* = 9.13×10^−6^ cm/s) and low-permeable Sulpiride (*Pe* = 0.00623×10^−6^ cm/s) it seems reasonable to acknowledge D2AAK1 as showing moderate passive transport through biological membranes (see Table S1).

**Table S1.** PAMPA results (permeability coefficient *Pe*) for tested compounds.

| Compound | *Pe* (10^−6^ cm/s) |
| --- | --- |
| D2AAK1 | 1.36 |
| Caffeine | 9.13 |
| Sulpiride | 0.00623 |

**Interaction with P-glycoprotein.** Efflux transporters such as P-glycoprotein (P-gp) play an important role in drug transport in many organs, including brain, where it is expressed naturally on the plasmatic membranes of endothelial cells at the blood-brain barrier. Interaction with the P-gp is a critical factor that may limit the drug penetration into brain. Therefore, D2AAK1 was tested for its influence on the recombinant Pgp ATPase activity, which represents compound ability to serve as a substrate of P-glycoprotein. Tested at 20 µM concentration, D2AAK1 did not influence the Pgp ATPase activity suggesting lack of P-gp substrate properties (see Fig. S1).





**Fig. S1.** Effect of D2AAK1 and reference P-gp substrates – Vincristine (VIN), Doxorubicin (DOX) and Verapamil (VER) on ATPase activity of P-glycoprotein. D2AAK1, VIN and DOX were tested at final concentration of 20 µM, whereas verapamil was used at 200 µM concentration. Data shown represent mean values ± SEM from two experiments conducted at least in duplicates.

**Influence on cytochrome P450 3A4 activity.** In order to assess the potential risk of drug-drug interactions D2AAK1 was tested for its influence on CYP3A4 activity using luminescent P450-Glo assay. The evaluated compound moderately inhibited CYP3A4 activity, however showed less pronounced effect than reference CYP3A4 inhibitor – ketoconazole (see Fig. S2).





**Fig. S2**. The effect of D2AAK1 and the reference inhibitor ketoconazole (KET) on CYP3A4 activity.

**Evaluation of neurotoxicity.** Moreover, D2AAK1 was tested for its potential undesirable neurotoxic activity at neuroblastoma SH‑SY5Y cells. The impact on cell viability was evaluated using MTS assay and compared with clinical cytotoxic drugs: Doxorubicin and Paclitaxel. The considered compound showed good safety profile inducing only 52% cell death at the concentration as high as 30 µM, which is in 100-fold less toxic effect than that of doxorubicin (see Fig. S3).





**Fig. S3.** SH-SY5Y cell viability after 72 h of incubation with the indicated compounds, evaluated using the CellTiter 96 AQueous nonradioactive cell proliferation assay. DOX, Doxorubicin; PTX, Paclitaxel.

**Synthesis of DOI**

DOI was needed as a reference compound used in this study. Due to difficulties in procuring the compound it was decided to synthesize in-house. DOI synthesis (Scheme S1) started with commercially available 2,5-dimethoxybenzaldehyde which was reacted with nitroethane via nitroaldol reaction to give desired nitroalkene.^3^ Commonly used ammonium acetate gave inferior yields in our hands compared to the reaction catalyzed by an amine. Therefore, a combination of butylamine together with acetic acid in isopropanol was tested and found to give satisfactory yields. Reduction of nitrostyrenes to their corresponding amines is usually carried out by lithium aluminum hydride (LAH) in anhydrous etheral solvents such as diethyl ether or tetrahydrofuran.^4^ We looked for an alternative route that did not involve LAH which is sensitive to moisture, fairly expensive and has some considerable safety issues when used in larger scale and often involves cumbersome workups. Catalytic reduction described by Kohno et al.^5^ seemed especially attractive. Unfortunately, the procedure failed to produce any product that could be extracted from the basic aqueous during a workup. Our attention turned to another method that has been used to perform the reduction in two separate steps, saturation of a double bond with sodium borohydride followed by nitro group reduction with nickel chloride.^6^ Observation made by Yoo et al where copper sulfate combined with sodium borohydride can reduce aliphatic nitro group^7^ added to a hypothesis that a potential one-pot method could be developed. It would utilize easier to handle reagents, and reaction could be performed conveniently in one pot. Ultimately, obtained 2,5-dimethoxyamphetamine was conveniently iodinated according to the method described by Wing-Wah et al.^8^ without the need to protect the amino group.





**Scheme S1.** Synthesis of DOI. Reagents and conditions: a) EtNO_2_, AcOH, BuNH_2_, 60°C, 77%; b) NaBH_4_, CuCl_2_, 44%; c) I_2_, Ag_2_SO_4_, 82%.


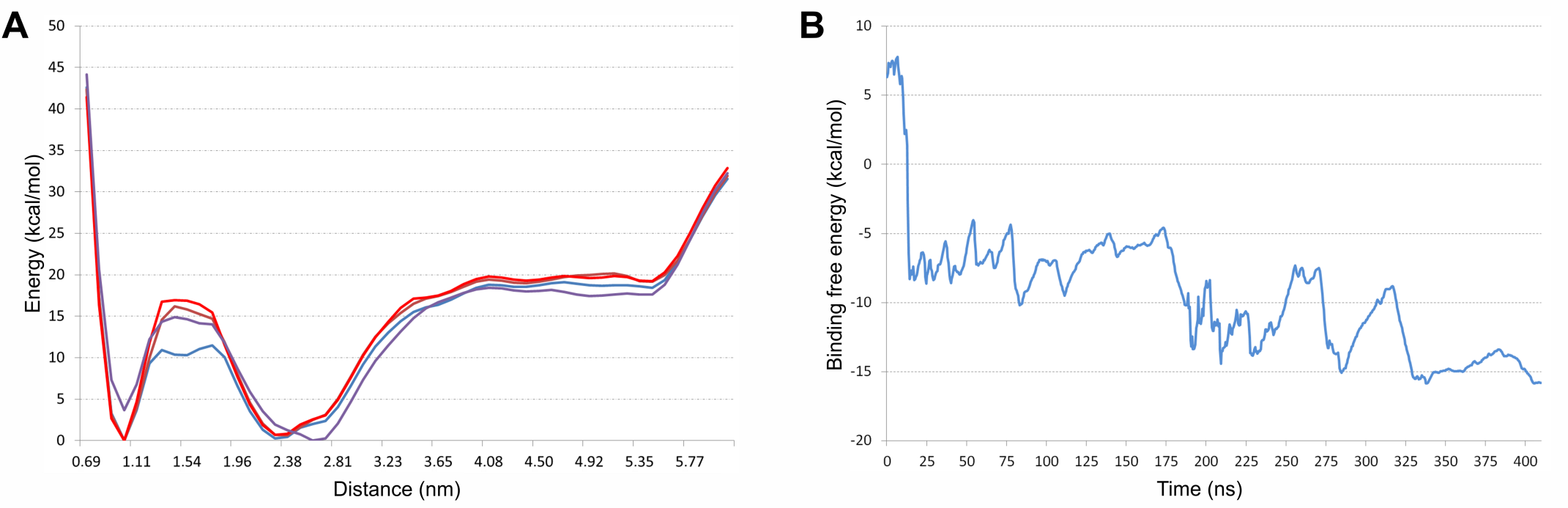


**Fig. S4.** Convergence of the Funnel Metadynamics simulations of the simulation box containing 5-HT2A receptor and D2AAK1 as a ligand. (A) 2D projection of the binding free energy landscapes in 10 ns intervals at the end of the simulation show two stable basins corresponding to two identified binding sites. (B) Evolution of calculated D2AAK1 binding free energy at the allosteric site during the FM simulation. During the last 70 ns of the simulation the binding free energy values are stabilized around -15 kcal/mol.


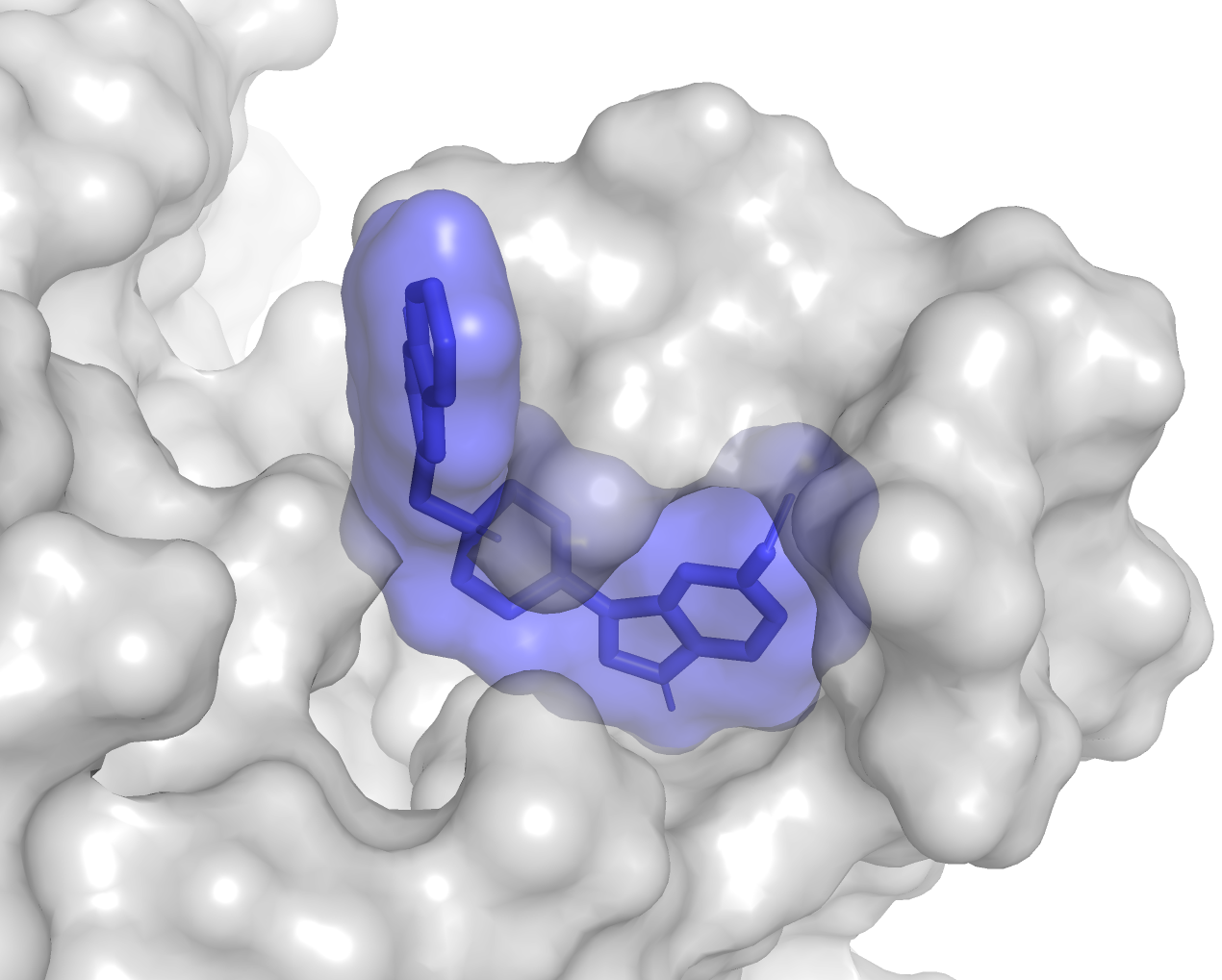


**Fig. S5.** Shape complementarity of D2AAK1 at the allosteric site in 5-HT2A receptor, shown with transparent surfaces.

**Supplementary Methods**

**ADMET parameters**

**Permeability profile.** Pre-coated PAMPA Plate System Gentest™ was obtained from Corning, (Tewksbury, MA, USA). It consists of 96-well receiver filter plate that has been pre-coated with structured layers of phospholipids and a matched donor microplate. The stock solutions of tested compounds and reference drugs were diluted in the PBS buffer (pH 7.4) to the final concentration of 100 µM. The compounds were applied into the donor wells (200 µl) and incubated (5 h) at RT. By using the UPLC-MS spectrometry (Waters ACQUITY™ TQD system with the TQ Detector, Waters, Milford, USA) with an internal standard the exact quantity of molecules that penetrated from donor to acceptor wells through phospholipid membrane was estimated. The permeability coefficients (Pe, cm/s) were calculated using the formula provided by the PAMPA Plate System manufacturer.^2^

**Interaction with P-glycoprotein.** Ppg-Glo Assay Kit (Promega) was used to investigate the direct effect of tested substances on P-gp ATPase activity. The assay was conducted in accordance with the manufacturer’s instructions. Verapamil was used as a reference substrate of P-gp, stimulating ATPase activity, whereas Na_3_VO_4_ was used as a reference blocker of ATPase activity. The assay was conducted in 96-well white microtiter plates. In the first step recombinant, human P-gp membranes (25 µg/well) were incubated for 5 min at 37°C with evaluated compounds. Following agents were tested in the assay: Na_3_VO_4_ (100 µM), Verapamil (200 µM), D2AAK1, Doxorubicin and Vincristine (20 µM). Assay buffer containing dilution solvent - 1% DMSO was used as the negative control. Following the preincubation period with tested compounds, P-gp ATPase reaction was initiated by the addition of enzyme substrate - 5 mM MgATP. The reaction was terminated after 40 minutes of incubation at 37°C by the addition of ATP Detection Reagent, which enables the detection of ATP remaining after the reaction catalyzed by the ATPase domain of P-gp protein. Assay plates were left at room temperature for additional 20 minutes in order to allow the luminescence signal to develop and were then analyzed using EnSpire Multiplate Reader (PerkinElmer). Basal activity of P-glycoprotein was calculated as the difference between average luminescent signals measured for samples treated with Na_3_VO_4_ (RLU_Na3VO4_) and average luminescent signals for untreated samples (RLU_NT_). P-glycoprotein ATPase activity in the presence of a test compound (RLU_TC_) was determined by subtracting the average luminescent signal detected for compound-treated samples RLU_TC_ from average luminescent signals from samples treated with Na_3_VO_4_ according to the equations below:

ΔRLU_basal_ = RLU_Na3VO4_ - RLU_NT_

ΔRLU_TC_ = RLU_Na3VO4_ - RLU_TC_

To determine the fold change of basal ATPase activity ΔRLU_TC_ was divided by ΔRLU_basal_. P-gp ATPase assay was performed in two separate experiments, each conducted at least in duplicates.

**Influence on cytochrome P450 3A4 activity.** Luminescent CYP3A4 P450-Glo kit was obtained from Promega® (Madison, WI, USA). The assay was performed according to the protocol provided by the manufacturer and as described before.^9^ Tests were performed twice in triplicates. The luminescent signal was measured using a microplate reader EnSpire PerkinElmer (Waltham, MA, USA).

**Evaluation of neurotoxicity.** Human neurobalstoma SH-SY5Y cell viability after 72 h of incubation with D2AAK1 and the reference cytostatic drugs: doxorubicin and paclitaxel was determined by performing the CellTiter 96 AQueous nonradioactive cell proliferation assay (Promega GmbH, Mannheim, Germany). Cells were seeded in a 96-well plate at 25 cells/μl in a total volume of 200 μl of medium 24 h prior to the experiment. On the next day, the cell culture medium was removed, and fresh medium was placed in the microplate wells. The compounds were then added to the final concentrations ranging from 0.3 to 30 μM. After the incubation period, the medium containing the tested compounds was removed, and 120 μl of fresh medium containing 3-(4,5-dimethylthiazol-2-yl)-5-(3-carboxymethoxyphenyl)-2-(4-sulfophenyl)-2H-tetrazolium (MTS) dye–phenazine methosulfate solution was added to each well. After 4 h of further incubation, the absorbance at 490 nm was measured directly from 96-well assay plates. Wells containing no cells were used as blank samples, whereas wells containing 1% DMSO were taken as nontreated controls (fully viable cells).

**Synthesis of DOI**

(**E)-1,4-dimethoxy-2-(2-nitroprop-1-en-1-yl)benzene.** 2,5-dimethoxybenzaldehyde (16.62g, 0.1mol) was dissolved in isopropanol (100mL). Nitroethane (15.01g, 0.2mol), acetic acid (2.86mL, 0.05mol) and butylamine (0.99mL, 0.01mol) were added in succession. Reaction was stirred at 60°C for 4 hours after which volatiles were removed *in vacuo*. Remaining amber oil was crystallized from methanol to obtain the title compound as a yellow solid (17.13g, 77%).

^1^H NMR (600 MHz, CDCl3) δ 8.24 (s, 1H), 6.96 (dd, J = 9.0, 3.4 Hz, 1H), 6.89 (d, J = 9.0 Hz, 1H), 6.86 (d, J = 3.1 Hz, 1H), 3.85 (s, 3H), 3.81 (s, 3H), 2.40 (d, J = 1.1 Hz, 3H).

^13^C NMR (151 MHz, CDCl3) δ 153.2, 152.5, 147.8, 129.6, 122.1, 116.1, 115.8, 111.8, 56.0, 55.9, 14.2.

**1-(2,5-dimethoxyphenyl)propan-2-amine.** Obtained nirostyrene (2.23g, 10mmol) was added portionwise (frothing) to a well stirred mixture of sodium borohydride (2.84g, 75mmol) in isopropanol (32mL) and water (16mL). An exothermic reaction emerged and yellow color eventually dissipated. Aqueous solution of copper chloride (0.5mL, 1mmol, 2M) was added dropwise which caused further frothing and appearance of black particles. After addition was complete, the reaction was heated at gentle reflux for 30 minutes. Reaction was allowed to cool to room temperature and it was acidified with conc. HCl (ca. 9mL, 37%). It was then diluted with water (100mL) and washed with chloroform (2 × 30mL). Subsequently it was made alkaline with conc. ammonia (ca. 20mL, 32%) and extracted with chloroform (2 × 30mL). Organics were pooled and dried with anhydrous magnesium sulfate. After solvent removal there was the title compound as a pale amber oil (0.86g, 44%). This can be distilled at 96-104°C/0.1Tr to obtain clear oil.

^1^H NMR (600 MHz, CDCl3) δ 6.78 – 6.76 (m, 1H), 6.73 – 6.70 (m, 2H), 3.76 (s, 3H), 3.75 (s, 3H), 3.23 – 3.16 (m, 1H), 2.71 (dd, J = 13.0, 5.4 Hz, 1H), 2.52 (dd, J = 13.0, 7.9 Hz, 1H), 1.11 (d, J = 6.4 Hz, 3H).

^13^C NMR (151 MHz, CDCl3) δ 153.3, 152.0, 129.3, 117.3, 111.4, 111.3, 55.8, 55.6, 47.1, 41.2, 23.5.

**1-(4-iodo-2,5-dimethoxyphenyl)propan-2-amine (DOI).** Ethanol (40mL, 96%) was added to silver sulfate (1.60g, 5mmol) and iodine (1.30g, 5mmol) and the mixture was stirred for 10 minutes at room temperature. 2,5-dimethoxyamphetamine (0.50g, 2.5mmol) was added as a solution in ethanol (10mL, 96%). The reaction was stirred at room temperature for 18 hours, after which time it was filtered and stripped of solvent. Remaining residue was taken up in chloroform (30mL) and washed subsequently with sodium thiosulfate (15mL, 1M), sodium hydroxide (15mL, 15%) and water (15mL). Drying with anhydrous magnesium sulfate and removing solvent gave amber oil which solidified on standing. Crude product was purified by flash column chromatography on silica eluting with 7M ammonia/MeOH in CHCl3 (4:96) obtaining the title compound as an off-white oil (671mg, 82%). The product was converted into hydrochloride salt by means of dissolving free base in ether and bubbling anhydrous HCl gas and characterized as such.

^1^H NMR (600 MHz, D2O) δ 7.37 (s, 1H), 6.81 (s, 1H), 3.76 (s, 3H), 3.73 (s, 3H), 3.58 (h, J = 6.8 Hz, 1H), 2.84 – 2.81 (m, 2H), 1.22 (d, J = 6.6 Hz, 3H).

^13^C NMR (151 MHz, D2O) δ 152.4, 152.1, 125.7, 122.4, 115.2, 83.8, 57.2, 56.2, 48.0, 35.0, 17.6.

**References:**

1. Di, L., Kerns, E.H. Drug-Like Properties: Concepts, Structure Design and Methods from ADME to Toxicity Optimization. Academic Press, Amsterdam **2016**.

2. Chen, X., Murawski, A., Patel, K., Crespi, C. L. & Balimane, P. V. A novel design of artificial membrane for improving the PAMPA model. *Pharm. Res.* **25**, 1511–1520 (2008).

3. Gairaud, C. B. & Lappin, G. R. The synthesis of ω-nitrostyrenes1. *J. Org. Chem.* **18**, 1–3 (1953).

4. Ho, B.-T. et al. Amphetamine analogs. II. Methylated phenethylamines. *J. Med. Chem*. **13**, 134–135 (1970).

5. Kohno, M.; Sasao, S.; Murahashi, S.-I., Synthesis of Phenethylamines by Hydrogenation of β-Nitrostyrenes. *Bull. Chem. Soc. Jpn.* 1990, 63 (4), 1252-1254.

6. Kulkarni, P. M. et al. Novel Electrophilic and Photoaffinity Covalent Probes for Mapping the Cannabinoid 1 Receptor Allosteric Site(s). *J. Med. Chem.* **59**, 44–60 (2016).

7. Yoo, S. & Lee, S. Reduction of Organic Compounds with Sodium Borohydride-Copper(II) Sulfate System. *Synlett*. **1990**, 419–420 (1990).

8. Wing-Wah, S. Iodination of methoxyamphetamines with iodine and silver sulfate. *Tetrahedron Lett.* **34**, 6223–6224 (1993).

9. Szczepańska, K. et al. Structural modifications in the distal, regulatory region of histamine H3 receptor antagonists leading to the identification of a potent anti-obesity agent. *Eur. J. Med. Chem.* **213**, 113041 (2021).

CDCl_3_ 600 MHz

CDCl_3_ 151 MHz

CDCl_3_ 600 MHz

CDCl_3_ 151 MHz

D_2_O, 600 MHz

D_2_O, 151 MHz

**Supplementary files**

**5ht1a-model.pdb**

ATOM 1 HH31 ACE 1 62.460 26.570 76.040 0.00 0.00 H

ATOM 2 CH3 ACE 1 61.480 26.350 76.470 0.00 0.00 C

ATOM 3 HH32 ACE 1 61.660 25.440 77.060 0.00 0.00 H

ATOM 4 HH33 ACE 1 60.710 26.070 75.750 0.00 0.00 H

ATOM 5 C ACE 1 60.980 27.450 77.370 0.00 0.00 C

ATOM 6 O ACE 1 61.770 28.290 77.770 0.00 0.00 O

ATOM 7 N THR 1 59.690 27.470 77.670 0.00 0.00 N

ATOM 8 H THR 1 59.220 26.630 77.360 0.00 0.00 H

ATOM 9 CA THR 1 59.040 28.370 78.630 0.00 0.00 C

ATOM 10 HA THR 1 59.560 29.330 78.690 0.00 0.00 H

ATOM 11 CB THR 1 57.580 28.530 78.240 0.00 0.00 C

ATOM 12 HB THR 1 56.940 29.030 78.970 0.00 0.00 H

ATOM 13 CG2 THR 1 57.290 29.300 76.960 0.00 0.00 C

ATOM 14 HG21 THR 1 57.450 30.360 77.180 0.00 0.00 H

ATOM 15 HG22 THR 1 58.020 28.950 76.230 0.00 0.00 H

ATOM 16 HG23 THR 1 56.230 29.200 76.740 0.00 0.00 H

ATOM 17 OG1 THR 1 56.970 27.290 78.120 0.00 0.00 O

ATOM 18 HG1 THR 1 56.140 27.340 78.600 0.00 0.00 H

ATOM 19 C THR 1 59.080 27.670 80.010 0.00 0.00 C

ATOM 20 O THR 1 58.770 26.480 80.200 0.00 0.00 O

ATOM 21 N THR 2 59.470 28.470 81.000 0.00 0.00 N

ATOM 22 H THR 2 59.560 29.470 80.890 0.00 0.00 H

ATOM 23 CA THR 2 59.820 27.890 82.360 0.00 0.00 C

ATOM 24 HA THR 2 60.190 26.860 82.370 0.00 0.00 H

ATOM 25 CB THR 2 61.040 28.550 82.930 0.00 0.00 C

ATOM 26 HB THR 2 61.160 28.160 83.940 0.00 0.00 H

ATOM 27 CG2 THR 2 62.330 28.260 82.090 0.00 0.00 C

ATOM 28 HG21 THR 2 62.360 28.890 81.200 0.00 0.00 H

ATOM 29 HG22 THR 2 63.170 28.650 82.660 0.00 0.00 H

ATOM 30 HG23 THR 2 62.410 27.190 81.930 0.00 0.00 H

ATOM 31 OG1 THR 2 60.850 29.950 83.020 0.00 0.00 O

ATOM 32 HG1 THR 2 60.610 30.180 83.920 0.00 0.00 H

ATOM 33 C THR 2 58.620 27.980 83.340 0.00 0.00 C

ATOM 34 O THR 2 58.790 27.450 84.400 0.00 0.00 O

ATOM 35 N GLY 3 57.540 28.590 82.840 0.00 0.00 N

ATOM 36 H GLY 3 57.620 28.940 81.890 0.00 0.00 H

ATOM 37 CA GLY 3 56.320 28.850 83.660 0.00 0.00 C

ATOM 38 HA1 GLY 3 56.620 29.260 84.630 0.00 0.00 H

ATOM 39 HA2 GLY 3 55.690 27.960 83.670 0.00 0.00 H

ATOM 40 C GLY 3 55.470 29.970 82.950 0.00 0.00 C

ATOM 41 O GLY 3 55.930 30.550 81.950 0.00 0.00 O

ATOM 42 N ILE 4 54.270 30.350 83.450 0.00 0.00 N

ATOM 43 H ILE 4 53.800 29.890 84.210 0.00 0.00 H

ATOM 44 CA ILE 4 53.370 31.410 82.870 0.00 0.00 C

ATOM 45 HA ILE 4 52.960 31.060 81.920 0.00 0.00 H

ATOM 46 CB ILE 4 52.190 31.660 83.810 0.00 0.00 C

ATOM 47 HB ILE 4 52.610 31.910 84.790 0.00 0.00 H

ATOM 48 CG2 ILE 4 51.350 32.900 83.360 0.00 0.00 C

ATOM 49 HG21 ILE 4 51.950 33.800 83.510 0.00 0.00 H

ATOM 50 HG22 ILE 4 50.990 32.720 82.340 0.00 0.00 H

ATOM 51 HG23 ILE 4 50.410 32.900 83.910 0.00 0.00 H

ATOM 52 CG1 ILE 4 51.380 30.390 84.090 0.00 0.00 C

ATOM 53 HG11 ILE 4 50.490 30.750 84.610 0.00 0.00 H

ATOM 54 HG12 ILE 4 51.920 29.690 84.730 0.00 0.00 H

ATOM 55 CD ILE 4 50.960 29.650 82.770 0.00 0.00 C

ATOM 56 HD1 ILE 4 51.820 29.040 82.510 0.00 0.00 H

ATOM 57 HD2 ILE 4 50.190 28.910 82.990 0.00 0.00 H

ATOM 58 HD3 ILE 4 50.660 30.280 81.930 0.00 0.00 H

ATOM 59 C ILE 4 54.150 32.730 82.770 0.00 0.00 C

ATOM 60 O ILE 4 54.670 33.230 83.770 0.00 0.00 O

ATOM 61 N SER 5 54.260 33.260 81.550 0.00 0.00 N

ATOM 62 H SER 5 53.630 32.860 80.860 0.00 0.00 H

ATOM 63 CA SER 5 54.990 34.510 81.220 0.00 0.00 C

ATOM 64 HA SER 5 54.760 34.740 80.180 0.00 0.00 H

ATOM 65 CB SER 5 54.290 35.630 82.080 0.00 0.00 C

ATOM 66 HB1 SER 5 54.810 36.560 81.830 0.00 0.00 H

ATOM 67 HB2 SER 5 54.390 35.530 83.160 0.00 0.00 H

ATOM 68 OG SER 5 52.870 35.850 81.750 0.00 0.00 O

ATOM 69 HG SER 5 52.530 36.390 82.460 0.00 0.00 H

ATOM 70 C SER 5 56.510 34.490 81.320 0.00 0.00 C

ATOM 71 O SER 5 57.190 35.520 81.350 0.00 0.00 O

ATOM 72 N ASP 6 57.180 33.320 81.300 0.00 0.00 N

ATOM 73 H ASP 6 56.590 32.500 81.270 0.00 0.00 H

ATOM 74 CA ASP 6 58.600 33.070 81.530 0.00 0.00 C

ATOM 75 HA ASP 6 59.060 34.040 81.350 0.00 0.00 H

ATOM 76 CB ASP 6 58.800 32.570 82.940 0.00 0.00 C

ATOM 77 HB1 ASP 6 58.480 31.550 83.170 0.00 0.00 H

ATOM 78 HB2 ASP 6 58.200 33.260 83.530 0.00 0.00 H

ATOM 79 CG ASP 6 60.130 32.860 83.540 0.00 0.00 C

ATOM 80 OD1 ASP 6 61.170 33.040 82.870 0.00 0.00 O

ATOM 81 OD2 ASP 6 60.250 33.030 84.760 0.00 0.00 O1-

ATOM 82 C ASP 6 59.220 32.080 80.510 0.00 0.00 C

ATOM 83 O ASP 6 58.540 31.180 80.010 0.00 0.00 O

ATOM 84 N VAL 7 60.490 32.230 80.320 0.00 0.00 N

ATOM 85 H VAL 7 60.890 32.750 81.090 0.00 0.00 H

ATOM 86 CA VAL 7 61.420 31.790 79.220 0.00 0.00 C

ATOM 87 HA VAL 7 61.020 30.850 78.840 0.00 0.00 H

ATOM 88 CB VAL 7 61.340 32.790 78.070 0.00 0.00 C

ATOM 89 HB VAL 7 62.200 32.540 77.450 0.00 0.00 H

ATOM 90 CG1 VAL 7 60.000 32.790 77.270 0.00 0.00 C

ATOM 91 HG11 VAL 7 59.850 31.780 76.870 0.00 0.00 H

ATOM 92 HG12 VAL 7 59.240 33.160 77.960 0.00 0.00 H

ATOM 93 HG13 VAL 7 59.980 33.530 76.470 0.00 0.00 H

ATOM 94 CG2 VAL 7 61.660 34.230 78.550 0.00 0.00 C

ATOM 95 HG21 VAL 7 61.490 34.970 77.770 0.00 0.00 H

ATOM 96 HG22 VAL 7 61.000 34.650 79.300 0.00 0.00 H

ATOM 97 HG23 VAL 7 62.690 34.340 78.920 0.00 0.00 H

ATOM 98 C VAL 7 62.870 31.420 79.600 0.00 0.00 C

ATOM 99 O VAL 7 63.450 32.050 80.500 0.00 0.00 O

ATOM 100 N THR 8 63.400 30.440 78.870 0.00 0.00 N

ATOM 101 H THR 8 62.800 29.830 78.340 0.00 0.00 H

ATOM 102 CA THR 8 64.880 30.310 78.720 0.00 0.00 C

ATOM 103 HA THR 8 65.340 30.580 79.670 0.00 0.00 H

ATOM 104 CB THR 8 65.230 28.830 78.490 0.00 0.00 C

ATOM 105 HB THR 8 66.310 28.690 78.360 0.00 0.00 H

ATOM 106 CG2 THR 8 64.770 27.930 79.580 0.00 0.00 C

ATOM 107 HG21 THR 8 63.680 27.880 79.620 0.00 0.00 H

ATOM 108 HG22 THR 8 65.010 26.870 79.430 0.00 0.00 H

ATOM 109 HG23 THR 8 65.000 28.300 80.580 0.00 0.00 H

ATOM 110 OG1 THR 8 64.590 28.350 77.270 0.00 0.00 O

ATOM 111 HG1 THR 8 63.760 28.070 77.660 0.00 0.00 H

ATOM 112 C THR 8 65.460 31.230 77.580 0.00 0.00 C

ATOM 113 O THR 8 64.700 31.620 76.680 0.00 0.00 O

ATOM 114 N VAL 9 66.790 31.590 77.580 0.00 0.00 N

ATOM 115 H VAL 9 67.310 31.380 78.420 0.00 0.00 H

ATOM 116 CA VAL 9 67.430 32.300 76.440 0.00 0.00 C

ATOM 117 HA VAL 9 66.740 33.100 76.160 0.00 0.00 H

ATOM 118 CB VAL 9 68.900 32.770 76.830 0.00 0.00 C

ATOM 119 HB VAL 9 69.470 32.020 77.370 0.00 0.00 H

ATOM 120 CG1 VAL 9 69.760 33.220 75.630 0.00 0.00 C

ATOM 121 HG11 VAL 9 69.980 32.380 74.980 0.00 0.00 H

ATOM 122 HG12 VAL 9 69.340 34.070 75.090 0.00 0.00 H

ATOM 123 HG13 VAL 9 70.660 33.640 76.090 0.00 0.00 H

ATOM 124 CG2 VAL 9 68.680 33.930 77.810 0.00 0.00 C

ATOM 125 HG21 VAL 9 67.950 34.600 77.350 0.00 0.00 H

ATOM 126 HG22 VAL 9 68.390 33.600 78.810 0.00 0.00 H

ATOM 127 HG23 VAL 9 69.580 34.520 77.980 0.00 0.00 H

ATOM 128 C VAL 9 67.550 31.350 75.190 0.00 0.00 C

ATOM 129 O VAL 9 67.340 31.740 74.070 0.00 0.00 O

ATOM 130 N SER 10 67.830 30.070 75.370 0.00 0.00 N

ATOM 131 H SER 10 68.260 29.800 76.240 0.00 0.00 H

ATOM 132 CA SER 10 67.470 29.040 74.320 0.00 0.00 C

ATOM 133 HA SER 10 68.270 28.840 73.600 0.00 0.00 H

ATOM 134 CB SER 10 67.470 27.700 75.030 0.00 0.00 C

ATOM 135 HB1 SER 10 67.140 27.000 74.270 0.00 0.00 H

ATOM 136 HB2 SER 10 66.750 27.740 75.850 0.00 0.00 H

ATOM 137 OG SER 10 68.770 27.430 75.550 0.00 0.00 O

ATOM 138 HG SER 10 68.940 28.020 76.290 0.00 0.00 H

ATOM 139 C SER 10 66.140 29.330 73.560 0.00 0.00 C

ATOM 140 O SER 10 66.210 29.690 72.370 0.00 0.00 O

ATOM 141 N TYR 11 64.990 29.290 74.220 0.00 0.00 N

ATOM 142 H TYR 11 65.070 29.150 75.210 0.00 0.00 H

ATOM 143 CA TYR 11 63.730 29.380 73.460 0.00 0.00 C

ATOM 144 HA TYR 11 63.730 28.650 72.640 0.00 0.00 H

ATOM 145 CB TYR 11 62.500 29.100 74.340 0.00 0.00 C

ATOM 146 HB1 TYR 11 62.650 29.560 75.310 0.00 0.00 H

ATOM 147 HB2 TYR 11 62.420 28.020 74.480 0.00 0.00 H

ATOM 148 CG TYR 11 61.100 29.510 73.880 0.00 0.00 C

ATOM 149 CD1 TYR 11 60.390 28.650 72.950 0.00 0.00 C

ATOM 150 HD1 TYR 11 60.830 27.700 72.680 0.00 0.00 H

ATOM 151 CE1 TYR 11 59.190 29.170 72.420 0.00 0.00 C

ATOM 152 HE1 TYR 11 58.570 28.640 71.720 0.00 0.00 H

ATOM 153 CZ TYR 11 58.590 30.410 72.850 0.00 0.00 C

ATOM 154 OH TYR 11 57.410 30.770 72.280 0.00 0.00 O

ATOM 155 HH TYR 11 57.130 31.580 72.700 0.00 0.00 H

ATOM 156 CE2 TYR 11 59.200 31.140 73.880 0.00 0.00 C

ATOM 157 HE2 TYR 11 58.820 32.090 74.230 0.00 0.00 H

ATOM 158 CD2 TYR 11 60.470 30.690 74.360 0.00 0.00 C

ATOM 159 HD2 TYR 11 60.980 31.220 75.150 0.00 0.00 H

ATOM 160 C TYR 11 63.680 30.700 72.750 0.00 0.00 C

ATOM 161 O TYR 11 63.220 30.710 71.610 0.00 0.00 O

ATOM 162 N GLN 12 64.080 31.750 73.470 0.00 0.00 N

ATOM 163 H GLN 12 64.480 31.640 74.390 0.00 0.00 H

ATOM 164 CA GLN 12 64.100 33.160 72.940 0.00 0.00 C

ATOM 165 HA GLN 12 63.050 33.400 72.770 0.00 0.00 H

ATOM 166 CB GLN 12 64.690 34.210 73.920 0.00 0.00 C

ATOM 167 HB1 GLN 12 65.040 35.130 73.450 0.00 0.00 H

ATOM 168 HB2 GLN 12 65.500 33.680 74.420 0.00 0.00 H

ATOM 169 CG GLN 12 63.580 34.640 74.910 0.00 0.00 C

ATOM 170 HG1 GLN 12 63.070 33.730 75.250 0.00 0.00 H

ATOM 171 HG2 GLN 12 62.920 35.320 74.370 0.00 0.00 H

ATOM 172 CD GLN 12 64.220 35.310 76.130 0.00 0.00 C

ATOM 173 OE1 GLN 12 64.270 36.500 76.390 0.00 0.00 O

ATOM 174 NE2 GLN 12 64.760 34.490 77.030 0.00 0.00 N

ATOM 175 HE21 GLN 12 64.880 33.500 76.920 0.00 0.00 H

ATOM 176 HE22 GLN 12 65.070 34.890 77.900 0.00 0.00 H

ATOM 177 C GLN 12 64.990 33.180 71.650 0.00 0.00 C

ATOM 178 O GLN 12 64.550 33.760 70.690 0.00 0.00 O

ATOM 179 N VAL 13 66.180 32.520 71.570 0.00 0.00 N

ATOM 180 H VAL 13 66.510 32.080 72.410 0.00 0.00 H

ATOM 181 CA VAL 13 67.070 32.520 70.430 0.00 0.00 C

ATOM 182 HA VAL 13 67.060 33.540 70.030 0.00 0.00 H

ATOM 183 CB VAL 13 68.500 32.190 70.890 0.00 0.00 C

ATOM 184 HB VAL 13 68.340 31.280 71.480 0.00 0.00 H

ATOM 185 CG1 VAL 13 69.560 31.730 69.920 0.00 0.00 C

ATOM 186 HG11 VAL 13 69.350 30.780 69.430 0.00 0.00 H

ATOM 187 HG12 VAL 13 69.640 32.500 69.150 0.00 0.00 H

ATOM 188 HG13 VAL 13 70.530 31.560 70.380 0.00 0.00 H

ATOM 189 CG2 VAL 13 69.040 33.350 71.790 0.00 0.00 C

ATOM 190 HG21 VAL 13 69.680 32.830 72.490 0.00 0.00 H

ATOM 191 HG22 VAL 13 69.640 34.080 71.230 0.00 0.00 H

ATOM 192 HG23 VAL 13 68.270 33.910 72.320 0.00 0.00 H

ATOM 193 C VAL 13 66.650 31.650 69.300 0.00 0.00 C

ATOM 194 O VAL 13 66.770 32.060 68.190 0.00 0.00 O

ATOM 195 N ILE 14 66.110 30.430 69.600 0.00 0.00 N

ATOM 196 H ILE 14 66.290 30.090 70.530 0.00 0.00 H

ATOM 197 CA ILE 14 65.570 29.460 68.630 0.00 0.00 C

ATOM 198 HA ILE 14 66.310 29.320 67.840 0.00 0.00 H

ATOM 199 CB ILE 14 65.280 28.100 69.230 0.00 0.00 C

ATOM 200 HB ILE 14 64.670 28.190 70.130 0.00 0.00 H

ATOM 201 CG2 ILE 14 64.600 27.040 68.380 0.00 0.00 C

ATOM 202 HG21 ILE 14 65.150 26.940 67.440 0.00 0.00 H

ATOM 203 HG22 ILE 14 64.540 26.040 68.810 0.00 0.00 H

ATOM 204 HG23 ILE 14 63.560 27.290 68.170 0.00 0.00 H

ATOM 205 CG1 ILE 14 66.640 27.450 69.570 0.00 0.00 C

ATOM 206 HG11 ILE 14 67.390 28.230 69.710 0.00 0.00 H

ATOM 207 HG12 ILE 14 67.080 26.960 68.700 0.00 0.00 H

ATOM 208 CD ILE 14 66.720 26.610 70.760 0.00 0.00 C

ATOM 209 HD1 ILE 14 65.950 25.840 70.820 0.00 0.00 H

ATOM 210 HD2 ILE 14 67.670 26.080 70.740 0.00 0.00 H

ATOM 211 HD3 ILE 14 66.630 27.290 71.610 0.00 0.00 H

ATOM 212 C ILE 14 64.330 30.070 67.970 0.00 0.00 C

ATOM 213 O ILE 14 64.330 30.360 66.720 0.00 0.00 O

ATOM 214 N THR 15 63.330 30.440 68.800 0.00 0.00 N

ATOM 215 H THR 15 63.310 30.150 69.760 0.00 0.00 H

ATOM 216 CA THR 15 62.060 31.000 68.230 0.00 0.00 C

ATOM 217 HA THR 15 61.720 30.360 67.410 0.00 0.00 H

ATOM 218 CB THR 15 60.900 30.880 69.230 0.00 0.00 C

ATOM 219 HB THR 15 61.060 31.550 70.080 0.00 0.00 H

ATOM 220 CG2 THR 15 59.510 30.910 68.560 0.00 0.00 C

ATOM 221 HG21 THR 15 58.750 31.490 69.080 0.00 0.00 H

ATOM 222 HG22 THR 15 59.530 31.330 67.560 0.00 0.00 H

ATOM 223 HG23 THR 15 59.170 29.880 68.600 0.00 0.00 H

ATOM 224 OG1 THR 15 61.170 29.630 69.920 0.00 0.00 O

ATOM 225 HG1 THR 15 61.740 29.840 70.660 0.00 0.00 H

ATOM 226 C THR 15 62.160 32.460 67.710 0.00 0.00 C

ATOM 227 O THR 15 61.520 32.860 66.770 0.00 0.00 O

ATOM 228 N SER 16 63.120 33.270 68.200 0.00 0.00 N

ATOM 229 H SER 16 63.710 32.910 68.940 0.00 0.00 H

ATOM 230 CA SER 16 63.440 34.590 67.570 0.00 0.00 C

ATOM 231 HA SER 16 62.460 35.080 67.530 0.00 0.00 H

ATOM 232 CB SER 16 64.300 35.600 68.360 0.00 0.00 C

ATOM 233 HB1 SER 16 63.740 35.950 69.220 0.00 0.00 H

ATOM 234 HB2 SER 16 64.440 36.460 67.700 0.00 0.00 H

ATOM 235 OG SER 16 65.530 34.990 68.710 0.00 0.00 O

ATOM 236 HG SER 16 65.250 34.510 69.500 0.00 0.00 H

ATOM 237 C SER 16 64.170 34.440 66.220 0.00 0.00 C

ATOM 238 O SER 16 63.820 35.160 65.320 0.00 0.00 O

ATOM 239 N LEU 17 65.020 33.370 66.070 0.00 0.00 N

ATOM 240 H LEU 17 65.360 32.960 66.930 0.00 0.00 H

ATOM 241 CA LEU 17 65.600 33.000 64.780 0.00 0.00 C

ATOM 242 HA LEU 17 66.120 33.880 64.400 0.00 0.00 H

ATOM 243 CB LEU 17 66.560 31.840 65.020 0.00 0.00 C

ATOM 244 HB1 LEU 17 66.510 31.240 64.110 0.00 0.00 H

ATOM 245 HB2 LEU 17 66.160 31.320 65.890 0.00 0.00 H

ATOM 246 CG LEU 17 68.040 32.210 65.210 0.00 0.00 C

ATOM 247 HG LEU 17 68.110 32.930 66.020 0.00 0.00 H

ATOM 248 CD1 LEU 17 68.890 31.010 65.690 0.00 0.00 C

ATOM 249 HD11 LEU 17 69.940 31.300 65.740 0.00 0.00 H

ATOM 250 HD12 LEU 17 68.620 30.710 66.700 0.00 0.00 H

ATOM 251 HD13 LEU 17 68.730 30.180 65.010 0.00 0.00 H

ATOM 252 CD2 LEU 17 68.660 32.880 64.000 0.00 0.00 C

ATOM 253 HD21 LEU 17 68.920 32.090 63.290 0.00 0.00 H

ATOM 254 HD22 LEU 17 68.000 33.610 63.540 0.00 0.00 H

ATOM 255 HD23 LEU 17 69.560 33.440 64.260 0.00 0.00 H

ATOM 256 C LEU 17 64.480 32.600 63.810 0.00 0.00 C

ATOM 257 O LEU 17 64.430 33.180 62.730 0.00 0.00 O

ATOM 258 N LEU 18 63.630 31.700 64.240 0.00 0.00 N

ATOM 259 H LEU 18 63.820 31.410 65.190 0.00 0.00 H

ATOM 260 CA LEU 18 62.530 30.970 63.500 0.00 0.00 C

ATOM 261 HA LEU 18 62.980 30.420 62.670 0.00 0.00 H

ATOM 262 CB LEU 18 61.700 29.930 64.320 0.00 0.00 C

ATOM 263 HB1 LEU 18 61.170 30.470 65.110 0.00 0.00 H

ATOM 264 HB2 LEU 18 62.300 29.180 64.830 0.00 0.00 H

ATOM 265 CG LEU 18 60.610 29.190 63.600 0.00 0.00 C

ATOM 266 HG LEU 18 59.950 29.900 63.100 0.00 0.00 H

ATOM 267 CD1 LEU 18 61.170 28.240 62.550 0.00 0.00 C

ATOM 268 HD11 LEU 18 62.050 27.700 62.890 0.00 0.00 H

ATOM 269 HD12 LEU 18 60.370 27.750 62.000 0.00 0.00 H

ATOM 270 HD13 LEU 18 61.560 28.880 61.760 0.00 0.00 H

ATOM 271 CD2 LEU 18 59.870 28.290 64.550 0.00 0.00 C

ATOM 272 HD21 LEU 18 59.420 28.960 65.280 0.00 0.00 H

ATOM 273 HD22 LEU 18 59.130 27.620 64.120 0.00 0.00 H

ATOM 274 HD23 LEU 18 60.630 27.680 65.050 0.00 0.00 H

ATOM 275 C LEU 18 61.520 32.030 62.880 0.00 0.00 C

ATOM 276 O LEU 18 61.310 32.290 61.660 0.00 0.00 O

ATOM 277 N LEU 19 60.830 32.660 63.830 0.00 0.00 N

ATOM 278 H LEU 19 61.170 32.800 64.770 0.00 0.00 H

ATOM 279 CA LEU 19 59.810 33.620 63.450 0.00 0.00 C

ATOM 280 HA LEU 19 59.190 33.220 62.650 0.00 0.00 H

ATOM 281 CB LEU 19 58.860 33.970 64.640 0.00 0.00 C

ATOM 282 HB1 LEU 19 58.240 34.790 64.270 0.00 0.00 H

ATOM 283 HB2 LEU 19 59.520 34.250 65.470 0.00 0.00 H

ATOM 284 CG LEU 19 57.970 32.780 65.240 0.00 0.00 C

ATOM 285 HG LEU 19 58.630 32.040 65.690 0.00 0.00 H

ATOM 286 CD1 LEU 19 57.090 33.370 66.350 0.00 0.00 C

ATOM 287 HD11 LEU 19 57.710 33.630 67.200 0.00 0.00 H

ATOM 288 HD12 LEU 19 56.610 34.290 66.000 0.00 0.00 H

ATOM 289 HD13 LEU 19 56.220 32.760 66.580 0.00 0.00 H

ATOM 290 CD2 LEU 19 57.110 32.230 64.050 0.00 0.00 C

ATOM 291 HD21 LEU 19 57.860 31.720 63.440 0.00 0.00 H

ATOM 292 HD22 LEU 19 56.460 31.410 64.370 0.00 0.00 H

ATOM 293 HD23 LEU 19 56.620 32.990 63.440 0.00 0.00 H

ATOM 294 C LEU 19 60.370 34.990 62.930 0.00 0.00 C

ATOM 295 O LEU 19 59.700 35.550 62.080 0.00 0.00 O

ATOM 296 N GLY 20 61.570 35.390 63.280 0.00 0.00 N

ATOM 297 H GLY 20 62.130 34.790 63.860 0.00 0.00 H

ATOM 298 CA GLY 20 62.270 36.470 62.570 0.00 0.00 C

ATOM 299 HA1 GLY 20 63.200 36.800 63.030 0.00 0.00 H

ATOM 300 HA2 GLY 20 61.610 37.340 62.600 0.00 0.00 H

ATOM 301 C GLY 20 62.660 36.110 61.140 0.00 0.00 C

ATOM 302 O GLY 20 62.720 37.020 60.340 0.00 0.00 O

ATOM 303 N THR 21 62.850 34.840 60.780 0.00 0.00 N

ATOM 304 H THR 21 62.740 34.040 61.390 0.00 0.00 H

ATOM 305 CA THR 21 63.050 34.440 59.370 0.00 0.00 C

ATOM 306 HA THR 21 63.700 35.210 58.960 0.00 0.00 H

ATOM 307 CB THR 21 63.760 33.130 59.340 0.00 0.00 C

ATOM 308 HB THR 21 63.110 32.370 59.770 0.00 0.00 H

ATOM 309 CG2 THR 21 64.240 32.860 57.930 0.00 0.00 C

ATOM 310 HG21 THR 21 64.830 33.720 57.630 0.00 0.00 H

ATOM 311 HG22 THR 21 64.820 31.950 57.740 0.00 0.00 H

ATOM 312 HG23 THR 21 63.320 32.770 57.340 0.00 0.00 H

ATOM 313 OG1 THR 21 65.010 33.260 60.010 0.00 0.00 O

ATOM 314 HG1 THR 21 64.890 33.300 60.970 0.00 0.00 H

ATOM 315 C THR 21 61.680 34.350 58.580 0.00 0.00 C

ATOM 316 O THR 21 61.500 35.010 57.560 0.00 0.00 O

ATOM 317 N LEU 22 60.700 33.650 59.200 0.00 0.00 N

ATOM 318 H LEU 22 60.970 33.150 60.040 0.00 0.00 H

ATOM 319 CA LEU 22 59.370 33.400 58.640 0.00 0.00 C

ATOM 320 HA LEU 22 59.590 33.060 57.630 0.00 0.00 H

ATOM 321 CB LEU 22 58.660 32.340 59.500 0.00 0.00 C

ATOM 322 HB1 LEU 22 58.340 32.810 60.430 0.00 0.00 H

ATOM 323 HB2 LEU 22 59.400 31.560 59.700 0.00 0.00 H

ATOM 324 CG LEU 22 57.460 31.650 58.810 0.00 0.00 C

ATOM 325 HG LEU 22 56.740 32.400 58.460 0.00 0.00 H

ATOM 326 CD1 LEU 22 57.910 30.800 57.570 0.00 0.00 C

ATOM 327 HD11 LEU 22 58.590 29.990 57.820 0.00 0.00 H

ATOM 328 HD12 LEU 22 57.050 30.520 56.960 0.00 0.00 H

ATOM 329 HD13 LEU 22 58.510 31.390 56.880 0.00 0.00 H

ATOM 330 CD2 LEU 22 56.640 30.840 59.780 0.00 0.00 C

ATOM 331 HD21 LEU 22 56.120 31.490 60.480 0.00 0.00 H

ATOM 332 HD22 LEU 22 55.850 30.260 59.300 0.00 0.00 H

ATOM 333 HD23 LEU 22 57.250 30.120 60.340 0.00 0.00 H

ATOM 334 C LEU 22 58.530 34.660 58.560 0.00 0.00 C

ATOM 335 O LEU 22 57.800 34.760 57.580 0.00 0.00 O

ATOM 336 N ILE 23 58.580 35.610 59.400 0.00 0.00 N

ATOM 337 H ILE 23 59.100 35.420 60.250 0.00 0.00 H

ATOM 338 CA ILE 23 57.830 36.930 59.180 0.00 0.00 C

ATOM 339 HA ILE 23 56.810 36.710 58.860 0.00 0.00 H

ATOM 340 CB ILE 23 57.830 37.740 60.460 0.00 0.00 C

ATOM 341 HB ILE 23 58.780 37.920 60.970 0.00 0.00 H

ATOM 342 CG2 ILE 23 57.160 39.090 60.230 0.00 0.00 C

ATOM 343 HG21 ILE 23 57.790 39.680 59.570 0.00 0.00 H

ATOM 344 HG22 ILE 23 56.130 38.980 59.890 0.00 0.00 H

ATOM 345 HG23 ILE 23 57.070 39.630 61.180 0.00 0.00 H

ATOM 346 CG1 ILE 23 57.020 36.920 61.520 0.00 0.00 C

ATOM 347 HG11 ILE 23 57.240 35.860 61.590 0.00 0.00 H

ATOM 348 HG12 ILE 23 56.020 36.950 61.090 0.00 0.00 H

ATOM 349 CD ILE 23 56.950 37.530 62.960 0.00 0.00 C

ATOM 350 HD1 ILE 23 56.520 38.530 62.970 0.00 0.00 H

ATOM 351 HD2 ILE 23 56.420 36.890 63.670 0.00 0.00 H

ATOM 352 HD3 ILE 23 57.940 37.560 63.410 0.00 0.00 H

ATOM 353 C ILE 23 58.370 37.800 58.000 0.00 0.00 C

ATOM 354 O ILE 23 57.570 38.270 57.270 0.00 0.00 O

ATOM 355 N PHE 24 59.720 37.780 57.890 0.00 0.00 N

ATOM 356 H PHE 24 60.310 37.230 58.500 0.00 0.00 H

ATOM 357 CA PHE 24 60.410 38.560 56.830 0.00 0.00 C

ATOM 358 HA PHE 24 60.080 39.590 56.960 0.00 0.00 H

ATOM 359 CB PHE 24 61.850 38.560 57.150 0.00 0.00 C

ATOM 360 HB1 PHE 24 62.130 37.510 57.030 0.00 0.00 H

ATOM 361 HB2 PHE 24 62.070 38.790 58.190 0.00 0.00 H

ATOM 362 CG PHE 24 62.770 39.400 56.280 0.00 0.00 C

ATOM 363 CD1 PHE 24 63.080 38.960 54.980 0.00 0.00 C

ATOM 364 HD1 PHE 24 62.670 38.050 54.550 0.00 0.00 H

ATOM 365 CE1 PHE 24 64.040 39.710 54.200 0.00 0.00 C

ATOM 366 HE1 PHE 24 64.290 39.380 53.200 0.00 0.00 H

ATOM 367 CZ PHE 24 64.610 40.890 54.740 0.00 0.00 C

ATOM 368 HZ PHE 24 65.150 41.580 54.100 0.00 0.00 H

ATOM 369 CE2 PHE 24 64.310 41.270 56.070 0.00 0.00 C

ATOM 370 HE2 PHE 24 64.950 42.040 56.480 0.00 0.00 H

ATOM 371 CD2 PHE 24 63.410 40.540 56.850 0.00 0.00 C

ATOM 372 HD2 PHE 24 63.130 40.780 57.860 0.00 0.00 H

ATOM 373 C PHE 24 60.050 37.910 55.530 0.00 0.00 C

ATOM 374 O PHE 24 59.640 38.590 54.570 0.00 0.00 O

ATOM 375 N CYS 25 60.010 36.600 55.500 0.00 0.00 N

ATOM 376 H CYS 25 60.380 36.120 56.310 0.00 0.00 H

ATOM 377 CA CYS 25 59.420 35.880 54.370 0.00 0.00 C

ATOM 378 HA CYS 25 59.970 36.070 53.450 0.00 0.00 H

ATOM 379 CB CYS 25 59.540 34.360 54.580 0.00 0.00 C

ATOM 380 HB1 CYS 25 59.080 33.740 53.810 0.00 0.00 H

ATOM 381 HB2 CYS 25 58.990 34.100 55.490 0.00 0.00 H

ATOM 382 SG CYS 25 61.240 33.800 54.830 0.00 0.00 S

ATOM 383 HG CYS 25 61.590 34.730 55.720 0.00 0.00 H

ATOM 384 C CYS 25 57.930 36.300 53.990 0.00 0.00 C

ATOM 385 O CYS 25 57.600 36.590 52.860 0.00 0.00 O

ATOM 386 N ALA 26 56.970 36.270 54.950 0.00 0.00 N

ATOM 387 H ALA 26 57.200 35.950 55.880 0.00 0.00 H

ATOM 388 CA ALA 26 55.600 36.650 54.780 0.00 0.00 C

ATOM 389 HA ALA 26 55.160 36.010 54.010 0.00 0.00 H

ATOM 390 CB ALA 26 54.890 36.380 56.090 0.00 0.00 C

ATOM 391 HB1 ALA 26 55.320 36.930 56.930 0.00 0.00 H

ATOM 392 HB2 ALA 26 53.820 36.570 56.080 0.00 0.00 H

ATOM 393 HB3 ALA 26 54.910 35.300 56.240 0.00 0.00 H

ATOM 394 C ALA 26 55.520 38.090 54.210 0.00 0.00 C

ATOM 395 O ALA 26 54.860 38.280 53.160 0.00 0.00 O

ATOM 396 N VAL 27 56.190 39.040 54.850 0.00 0.00 N

ATOM 397 H VAL 27 56.700 38.810 55.690 0.00 0.00 H

ATOM 398 CA VAL 27 56.210 40.390 54.320 0.00 0.00 C

ATOM 399 HA VAL 27 55.240 40.880 54.420 0.00 0.00 H

ATOM 400 CB VAL 27 57.120 41.280 55.250 0.00 0.00 C

ATOM 401 HB VAL 27 57.990 40.650 55.400 0.00 0.00 H

ATOM 402 CG1 VAL 27 57.470 42.650 54.620 0.00 0.00 C

ATOM 403 HG11 VAL 27 58.220 43.170 55.210 0.00 0.00 H

ATOM 404 HG12 VAL 27 57.750 42.690 53.570 0.00 0.00 H

ATOM 405 HG13 VAL 27 56.620 43.330 54.610 0.00 0.00 H

ATOM 406 CG2 VAL 27 56.550 41.470 56.660 0.00 0.00 C

ATOM 407 HG21 VAL 27 55.470 41.610 56.730 0.00 0.00 H

ATOM 408 HG22 VAL 27 56.790 40.560 57.210 0.00 0.00 H

ATOM 409 HG23 VAL 27 57.130 42.180 57.260 0.00 0.00 H

ATOM 410 C VAL 27 56.640 40.500 52.890 0.00 0.00 C

ATOM 411 O VAL 27 56.020 41.180 52.060 0.00 0.00 O

ATOM 412 N LEU 28 57.740 39.760 52.620 0.00 0.00 N

ATOM 413 H LEU 28 58.230 39.280 53.360 0.00 0.00 H

ATOM 414 CA LEU 28 58.390 39.760 51.290 0.00 0.00 C

ATOM 415 HA LEU 28 58.560 40.830 51.150 0.00 0.00 H

ATOM 416 CB LEU 28 59.670 38.930 51.360 0.00 0.00 C

ATOM 417 HB1 LEU 28 59.380 37.940 51.700 0.00 0.00 H

ATOM 418 HB2 LEU 28 60.260 39.330 52.180 0.00 0.00 H

ATOM 419 CG LEU 28 60.560 38.820 50.090 0.00 0.00 C

ATOM 420 HG LEU 28 60.140 38.160 49.340 0.00 0.00 H

ATOM 421 CD1 LEU 28 61.000 40.190 49.520 0.00 0.00 C

ATOM 422 HD11 LEU 28 61.900 40.030 48.920 0.00 0.00 H

ATOM 423 HD12 LEU 28 60.140 40.600 49.000 0.00 0.00 H

ATOM 424 HD13 LEU 28 61.230 40.920 50.300 0.00 0.00 H

ATOM 425 CD2 LEU 28 61.890 38.170 50.730 0.00 0.00 C

ATOM 426 HD21 LEU 28 61.710 37.120 50.970 0.00 0.00 H

ATOM 427 HD22 LEU 28 62.640 38.260 49.950 0.00 0.00 H

ATOM 428 HD23 LEU 28 62.260 38.680 51.620 0.00 0.00 H

ATOM 429 C LEU 28 57.410 39.270 50.260 0.00 0.00 C

ATOM 430 O LEU 28 57.320 39.900 49.150 0.00 0.00 O

ATOM 431 N GLY 29 56.740 38.170 50.380 0.00 0.00 N

ATOM 432 H GLY 29 57.110 37.530 51.080 0.00 0.00 H

ATOM 433 CA GLY 29 55.940 37.600 49.260 0.00 0.00 C

ATOM 434 HA1 GLY 29 55.760 36.530 49.400 0.00 0.00 H

ATOM 435 HA2 GLY 29 56.510 37.690 48.330 0.00 0.00 H

ATOM 436 C GLY 29 54.630 38.350 49.070 0.00 0.00 C

ATOM 437 O GLY 29 54.340 38.710 47.890 0.00 0.00 O

ATOM 438 N ASN 30 53.960 38.940 50.130 0.00 0.00 N

ATOM 439 H ASN 30 54.180 38.730 51.100 0.00 0.00 H

ATOM 440 CA ASN 30 52.890 39.850 49.880 0.00 0.00 C

ATOM 441 HA ASN 30 52.190 39.380 49.190 0.00 0.00 H

ATOM 442 CB ASN 30 52.160 40.300 51.130 0.00 0.00 C

ATOM 443 HB1 ASN 30 51.380 41.030 50.910 0.00 0.00 H

ATOM 444 HB2 ASN 30 52.810 40.730 51.880 0.00 0.00 H

ATOM 445 CG ASN 30 51.310 39.210 51.720 0.00 0.00 C

ATOM 446 OD1 ASN 30 50.260 38.840 51.250 0.00 0.00 O

ATOM 447 ND2 ASN 30 51.950 38.470 52.610 0.00 0.00 N

ATOM 448 HD21 ASN 30 52.780 38.700 53.140 0.00 0.00 H

ATOM 449 HD22 ASN 30 51.530 37.560 52.740 0.00 0.00 H

ATOM 450 C ASN 30 53.370 41.130 49.170 0.00 0.00 C

ATOM 451 O ASN 30 52.730 41.680 48.300 0.00 0.00 O

ATOM 452 N ALA 31 54.530 41.690 49.570 0.00 0.00 N

ATOM 453 H ALA 31 54.990 41.310 50.390 0.00 0.00 H

ATOM 454 CA ALA 31 55.170 42.850 48.930 0.00 0.00 C

ATOM 455 HA ALA 31 54.500 43.700 49.100 0.00 0.00 H

ATOM 456 CB ALA 31 56.480 43.330 49.620 0.00 0.00 C

ATOM 457 HB1 ALA 31 56.350 43.410 50.700 0.00 0.00 H

ATOM 458 HB2 ALA 31 57.230 42.590 49.310 0.00 0.00 H

ATOM 459 HB3 ALA 31 56.780 44.340 49.360 0.00 0.00 H

ATOM 460 C ALA 31 55.420 42.620 47.400 0.00 0.00 C

ATOM 461 O ALA 31 54.940 43.360 46.600 0.00 0.00 O

ATOM 462 N CYS 32 56.000 41.440 47.100 0.00 0.00 N

ATOM 463 H CYS 32 56.370 40.780 47.770 0.00 0.00 H

ATOM 464 CA CYS 32 56.110 41.010 45.730 0.00 0.00 C

ATOM 465 HA CYS 32 56.680 41.750 45.170 0.00 0.00 H

ATOM 466 CB CYS 32 56.750 39.660 45.630 0.00 0.00 C

ATOM 467 HB1 CYS 32 56.760 39.290 44.610 0.00 0.00 H

ATOM 468 HB2 CYS 32 56.270 38.890 46.240 0.00 0.00 H

ATOM 469 SG CYS 32 58.420 39.870 46.290 0.00 0.00 S

ATOM 470 HG CYS 32 58.240 39.550 47.580 0.00 0.00 H

ATOM 471 C CYS 32 54.690 40.940 45.050 0.00 0.00 C

ATOM 472 O CYS 32 54.690 41.420 43.910 0.00 0.00 O

ATOM 473 N VAL 33 53.590 40.420 45.640 0.00 0.00 N

ATOM 474 H VAL 33 53.760 40.100 46.590 0.00 0.00 H

ATOM 475 CA VAL 33 52.210 40.460 45.180 0.00 0.00 C

ATOM 476 HA VAL 33 52.310 39.940 44.220 0.00 0.00 H

ATOM 477 CB VAL 33 51.270 39.670 46.070 0.00 0.00 C

ATOM 478 HB VAL 33 51.330 39.950 47.120 0.00 0.00 H

ATOM 479 CG1 VAL 33 49.790 39.820 45.660 0.00 0.00 C

ATOM 480 HG11 VAL 33 49.110 39.400 46.400 0.00 0.00 H

ATOM 481 HG12 VAL 33 49.510 40.870 45.660 0.00 0.00 H

ATOM 482 HG13 VAL 33 49.640 39.500 44.620 0.00 0.00 H

ATOM 483 CG2 VAL 33 51.490 38.140 46.080 0.00 0.00 C

ATOM 484 HG21 VAL 33 52.530 37.970 46.350 0.00 0.00 H

ATOM 485 HG22 VAL 33 50.860 37.560 46.760 0.00 0.00 H

ATOM 486 HG23 VAL 33 51.250 37.700 45.120 0.00 0.00 H

ATOM 487 C VAL 33 51.720 41.820 44.820 0.00 0.00 C

ATOM 488 O VAL 33 51.110 42.030 43.730 0.00 0.00 O

ATOM 489 N VAL 34 52.040 42.820 45.650 0.00 0.00 N

ATOM 490 H VAL 34 52.590 42.630 46.480 0.00 0.00 H

ATOM 491 CA VAL 34 51.640 44.220 45.450 0.00 0.00 C

ATOM 492 HA VAL 34 50.620 44.160 45.060 0.00 0.00 H

ATOM 493 CB VAL 34 51.630 45.150 46.750 0.00 0.00 C

ATOM 494 HB VAL 34 52.640 45.140 47.170 0.00 0.00 H

ATOM 495 CG1 VAL 34 51.310 46.610 46.470 0.00 0.00 C

ATOM 496 HG11 VAL 34 52.150 46.990 45.880 0.00 0.00 H

ATOM 497 HG12 VAL 34 50.420 46.730 45.870 0.00 0.00 H

ATOM 498 HG13 VAL 34 51.160 47.160 47.400 0.00 0.00 H

ATOM 499 CG2 VAL 34 50.750 44.550 47.870 0.00 0.00 C

ATOM 500 HG21 VAL 34 50.860 45.090 48.810 0.00 0.00 H

ATOM 501 HG22 VAL 34 49.780 44.610 47.380 0.00 0.00 H

ATOM 502 HG23 VAL 34 50.960 43.480 47.870 0.00 0.00 H

ATOM 503 C VAL 34 52.440 44.860 44.280 0.00 0.00 C

ATOM 504 O VAL 34 51.870 45.560 43.420 0.00 0.00 O

ATOM 505 N ALA 35 53.740 44.680 44.300 0.00 0.00 N

ATOM 506 H ALA 35 54.210 44.110 44.990 0.00 0.00 H

ATOM 507 CA ALA 35 54.610 45.120 43.230 0.00 0.00 C

ATOM 508 HA ALA 35 54.500 46.190 43.070 0.00 0.00 H

ATOM 509 CB ALA 35 56.070 44.720 43.640 0.00 0.00 C

ATOM 510 HB1 ALA 35 56.170 43.640 43.720 0.00 0.00 H

ATOM 511 HB2 ALA 35 56.760 45.070 42.870 0.00 0.00 H

ATOM 512 HB3 ALA 35 56.270 45.220 44.590 0.00 0.00 H

ATOM 513 C ALA 35 54.220 44.490 41.870 0.00 0.00 C

ATOM 514 O ALA 35 54.100 45.220 40.890 0.00 0.00 O

ATOM 515 N ALA 36 53.960 43.210 41.830 0.00 0.00 N

ATOM 516 H ALA 36 54.110 42.680 42.680 0.00 0.00 H

ATOM 517 CA ALA 36 53.460 42.470 40.640 0.00 0.00 C

ATOM 518 HA ALA 36 54.280 42.520 39.920 0.00 0.00 H

ATOM 519 CB ALA 36 53.230 41.020 41.080 0.00 0.00 C

ATOM 520 HB1 ALA 36 52.370 41.010 41.750 0.00 0.00 H

ATOM 521 HB2 ALA 36 52.900 40.530 40.160 0.00 0.00 H

ATOM 522 HB3 ALA 36 54.080 40.560 41.590 0.00 0.00 H

ATOM 523 C ALA 36 52.220 43.130 40.060 0.00 0.00 C

ATOM 524 O ALA 36 52.120 43.260 38.860 0.00 0.00 O

ATOM 525 N ILE 37 51.220 43.460 40.890 0.00 0.00 N

ATOM 526 H ILE 37 51.550 43.460 41.850 0.00 0.00 H

ATOM 527 CA ILE 37 50.040 44.230 40.510 0.00 0.00 C

ATOM 528 HA ILE 37 49.550 43.720 39.670 0.00 0.00 H

ATOM 529 CB ILE 37 48.980 44.320 41.710 0.00 0.00 C

ATOM 530 HB ILE 37 49.500 44.690 42.600 0.00 0.00 H

ATOM 531 CG2 ILE 37 47.900 45.370 41.340 0.00 0.00 C

ATOM 532 HG21 ILE 37 47.520 45.160 40.340 0.00 0.00 H

ATOM 533 HG22 ILE 37 47.030 45.410 42.000 0.00 0.00 H

ATOM 534 HG23 ILE 37 48.380 46.360 41.310 0.00 0.00 H

ATOM 535 CG1 ILE 37 48.360 42.890 41.990 0.00 0.00 C

ATOM 536 HG11 ILE 37 49.140 42.160 42.220 0.00 0.00 H

ATOM 537 HG12 ILE 37 47.750 42.520 41.170 0.00 0.00 H

ATOM 538 CD ILE 37 47.430 42.880 43.190 0.00 0.00 C

ATOM 539 HD1 ILE 37 47.240 41.830 43.420 0.00 0.00 H

ATOM 540 HD2 ILE 37 47.840 43.320 44.100 0.00 0.00 H

ATOM 541 HD3 ILE 37 46.510 43.430 43.000 0.00 0.00 H

ATOM 542 C ILE 37 50.350 45.630 39.980 0.00 0.00 C

ATOM 543 O ILE 37 49.800 46.090 38.970 0.00 0.00 O

ATOM 544 N ALA 38 51.240 46.330 40.640 0.00 0.00 N

ATOM 545 H ALA 38 51.520 45.880 41.500 0.00 0.00 H

ATOM 546 CA ALA 38 51.740 47.640 40.120 0.00 0.00 C

ATOM 547 HA ALA 38 50.810 48.200 39.980 0.00 0.00 H

ATOM 548 CB ALA 38 52.570 48.260 41.280 0.00 0.00 C

ATOM 549 HB1 ALA 38 52.740 49.320 41.130 0.00 0.00 H

ATOM 550 HB2 ALA 38 52.070 48.210 42.240 0.00 0.00 H

ATOM 551 HB3 ALA 38 53.530 47.740 41.250 0.00 0.00 H

ATOM 552 C ALA 38 52.510 47.550 38.840 0.00 0.00 C

ATOM 553 O ALA 38 52.570 48.480 38.040 0.00 0.00 O

ATOM 554 N LEU 39 53.240 46.420 38.630 0.00 0.00 N

ATOM 555 H LEU 39 53.160 45.820 39.440 0.00 0.00 H

ATOM 556 CA LEU 39 54.170 46.090 37.540 0.00 0.00 C

ATOM 557 HA LEU 39 54.570 46.990 37.080 0.00 0.00 H

ATOM 558 CB LEU 39 55.220 45.170 38.120 0.00 0.00 C

ATOM 559 HB1 LEU 39 54.710 44.240 38.370 0.00 0.00 H

ATOM 560 HB2 LEU 39 55.520 45.700 39.030 0.00 0.00 H

ATOM 561 CG LEU 39 56.450 44.810 37.270 0.00 0.00 C

ATOM 562 HG LEU 39 56.140 44.400 36.310 0.00 0.00 H

ATOM 563 CD1 LEU 39 57.220 46.100 36.930 0.00 0.00 C

ATOM 564 HD11 LEU 39 58.160 45.840 36.440 0.00 0.00 H

ATOM 565 HD12 LEU 39 56.640 46.710 36.250 0.00 0.00 H

ATOM 566 HD13 LEU 39 57.470 46.720 37.800 0.00 0.00 H

ATOM 567 CD2 LEU 39 57.140 43.650 38.050 0.00 0.00 C

ATOM 568 HD21 LEU 39 56.620 42.700 38.020 0.00 0.00 H

ATOM 569 HD22 LEU 39 58.100 43.480 37.550 0.00 0.00 H

ATOM 570 HD23 LEU 39 57.270 43.950 39.090 0.00 0.00 H

ATOM 571 C LEU 39 53.530 45.360 36.300 0.00 0.00 C

ATOM 572 O LEU 39 54.000 45.600 35.190 0.00 0.00 O

ATOM 573 N GLU 40 52.500 44.550 36.390 0.00 0.00 N

ATOM 574 H GLU 40 52.180 44.440 37.340 0.00 0.00 H

ATOM 575 CA GLU 40 51.990 43.630 35.370 0.00 0.00 C

ATOM 576 HA GLU 40 52.540 43.730 34.430 0.00 0.00 H

ATOM 577 CB GLU 40 52.020 42.220 35.950 0.00 0.00 C

ATOM 578 HB1 GLU 40 51.220 42.160 36.690 0.00 0.00 H

ATOM 579 HB2 GLU 40 52.990 41.990 36.380 0.00 0.00 H

ATOM 580 CG GLU 40 51.650 41.250 34.880 0.00 0.00 C

ATOM 581 HG1 GLU 40 50.740 41.580 34.370 0.00 0.00 H

ATOM 582 HG2 GLU 40 51.300 40.350 35.370 0.00 0.00 H

ATOM 583 CD GLU 40 52.780 40.930 33.880 0.00 0.00 C

ATOM 584 OE1 GLU 40 53.920 40.680 34.310 0.00 0.00 O

ATOM 585 OE2 GLU 40 52.680 41.130 32.630 0.00 0.00 O1-

ATOM 586 C GLU 40 50.510 44.140 35.150 0.00 0.00 C

ATOM 587 O GLU 40 49.620 43.860 35.950 0.00 0.00 O

ATOM 588 N ARG 41 50.190 44.880 34.050 0.00 0.00 N

ATOM 589 H ARG 41 50.810 44.780 33.250 0.00 0.00 H

ATOM 590 CA ARG 41 48.960 45.460 33.730 0.00 0.00 C

ATOM 591 HA ARG 41 48.780 46.180 34.530 0.00 0.00 H

ATOM 592 CB ARG 41 49.160 46.190 32.390 0.00 0.00 C

ATOM 593 HB1 ARG 41 49.430 45.450 31.630 0.00 0.00 H

ATOM 594 HB2 ARG 41 49.950 46.940 32.490 0.00 0.00 H

ATOM 595 CG ARG 41 47.980 46.890 31.740 0.00 0.00 C

ATOM 596 HG1 ARG 41 47.180 46.190 31.490 0.00 0.00 H

ATOM 597 HG2 ARG 41 48.380 47.540 30.960 0.00 0.00 H

ATOM 598 CD ARG 41 47.420 47.790 32.780 0.00 0.00 C

ATOM 599 HD1 ARG 41 48.250 48.370 33.190 0.00 0.00 H

ATOM 600 HD2 ARG 41 46.900 47.240 33.560 0.00 0.00 H

ATOM 601 NE ARG 41 46.500 48.780 32.230 0.00 0.00 N

ATOM 602 HE ARG 41 46.210 48.580 31.290 0.00 0.00 H

ATOM 603 CZ ARG 41 45.860 49.800 32.870 0.00 0.00 C

ATOM 604 NH1 ARG 41 46.020 49.970 34.180 0.00 0.00 N1+

ATOM 605 HH11 ARG 41 46.630 49.390 34.740 0.00 0.00 H

ATOM 606 HH12 ARG 41 45.810 50.870 34.590 0.00 0.00 H

ATOM 607 NH2 ARG 41 45.050 50.600 32.240 0.00 0.00 N

ATOM 608 HH21 ARG 41 45.040 50.620 31.230 0.00 0.00 H

ATOM 609 HH22 ARG 41 44.520 51.230 32.820 0.00 0.00 H

ATOM 610 C ARG 41 47.880 44.340 33.640 0.00 0.00 C

ATOM 611 O ARG 41 46.770 44.450 34.160 0.00 0.00 O

ATOM 612 N SER 42 48.250 43.220 33.050 0.00 0.00 N

ATOM 613 H SER 42 49.150 43.240 32.600 0.00 0.00 H

ATOM 614 CA SER 42 47.550 41.930 33.120 0.00 0.00 C

ATOM 615 HA SER 42 46.730 41.850 32.410 0.00 0.00 H

ATOM 616 CB SER 42 48.420 40.860 32.440 0.00 0.00 C

ATOM 617 HB1 SER 42 48.000 39.890 32.700 0.00 0.00 H

ATOM 618 HB2 SER 42 49.450 40.940 32.790 0.00 0.00 H

ATOM 619 OG SER 42 48.470 41.220 31.080 0.00 0.00 O

ATOM 620 HG SER 42 48.420 40.420 30.560 0.00 0.00 H

ATOM 621 C SER 42 47.100 41.400 34.500 0.00 0.00 C

ATOM 622 O SER 42 46.330 40.390 34.510 0.00 0.00 O

ATOM 623 N LEU 43 47.410 41.980 35.600 0.00 0.00 N

ATOM 624 H LEU 43 48.080 42.740 35.540 0.00 0.00 H

ATOM 625 CA LEU 43 47.000 41.710 37.020 0.00 0.00 C

ATOM 626 HA LEU 43 46.400 40.810 37.020 0.00 0.00 H

ATOM 627 CB LEU 43 48.320 41.360 37.880 0.00 0.00 C

ATOM 628 HB1 LEU 43 48.110 41.200 38.940 0.00 0.00 H

ATOM 629 HB2 LEU 43 48.950 42.250 37.890 0.00 0.00 H

ATOM 630 CG LEU 43 49.030 40.060 37.460 0.00 0.00 C

ATOM 631 HG LEU 43 49.190 40.050 36.380 0.00 0.00 H

ATOM 632 CD1 LEU 43 50.300 39.950 38.260 0.00 0.00 C

ATOM 633 HD11 LEU 43 50.170 39.980 39.340 0.00 0.00 H

ATOM 634 HD12 LEU 43 50.670 38.940 38.060 0.00 0.00 H

ATOM 635 HD13 LEU 43 51.030 40.710 38.010 0.00 0.00 H

ATOM 636 CD2 LEU 43 48.190 38.820 37.810 0.00 0.00 C

ATOM 637 HD21 LEU 43 47.310 38.810 37.170 0.00 0.00 H

ATOM 638 HD22 LEU 43 48.800 37.960 37.540 0.00 0.00 H

ATOM 639 HD23 LEU 43 48.000 38.890 38.880 0.00 0.00 H

ATOM 640 C LEU 43 46.250 42.870 37.690 0.00 0.00 C

ATOM 641 O LEU 43 45.800 42.690 38.840 0.00 0.00 O

ATOM 642 N GLN 44 45.910 43.900 36.960 0.00 0.00 N

ATOM 643 H GLN 44 46.100 43.840 35.970 0.00 0.00 H

ATOM 644 CA GLN 44 45.030 45.030 37.320 0.00 0.00 C

ATOM 645 HA GLN 44 44.930 45.100 38.400 0.00 0.00 H

ATOM 646 CB GLN 44 45.680 46.290 36.750 0.00 0.00 C

ATOM 647 HB1 GLN 44 45.090 47.140 37.070 0.00 0.00 H

ATOM 648 HB2 GLN 44 45.680 46.290 35.660 0.00 0.00 H

ATOM 649 CG GLN 44 47.120 46.460 37.300 0.00 0.00 C

ATOM 650 HG1 GLN 44 47.770 45.590 37.170 0.00 0.00 H

ATOM 651 HG2 GLN 44 47.090 46.760 38.340 0.00 0.00 H

ATOM 652 CD GLN 44 47.940 47.530 36.600 0.00 0.00 C

ATOM 653 OE1 GLN 44 47.370 48.410 35.980 0.00 0.00 O

ATOM 654 NE2 GLN 44 49.260 47.510 36.680 0.00 0.00 N

ATOM 655 HE21 GLN 44 49.720 46.820 37.260 0.00 0.00 H

ATOM 656 HE22 GLN 44 49.810 48.200 36.200 0.00 0.00 H

ATOM 657 C GLN 44 43.560 44.840 36.890 0.00 0.00 C

ATOM 658 O GLN 44 42.840 45.780 36.570 0.00 0.00 O

ATOM 659 N ASN 45 43.100 43.600 36.750 0.00 0.00 N

ATOM 660 H ASN 45 43.760 42.870 36.970 0.00 0.00 H

ATOM 661 CA ASN 45 41.750 43.150 36.380 0.00 0.00 C

ATOM 662 HA ASN 45 41.440 43.670 35.480 0.00 0.00 H

ATOM 663 CB ASN 45 41.820 41.650 35.900 0.00 0.00 C

ATOM 664 HB1 ASN 45 42.190 41.040 36.720 0.00 0.00 H

ATOM 665 HB2 ASN 45 42.450 41.570 35.010 0.00 0.00 H

ATOM 666 CG ASN 45 40.410 41.150 35.640 0.00 0.00 C

ATOM 667 OD1 ASN 45 39.610 40.760 36.480 0.00 0.00 O

ATOM 668 ND2 ASN 45 39.920 41.140 34.370 0.00 0.00 N

ATOM 669 HD21 ASN 45 40.370 41.760 33.710 0.00 0.00 H

ATOM 670 HD22 ASN 45 39.080 40.610 34.180 0.00 0.00 H

ATOM 671 C ASN 45 40.730 43.400 37.540 0.00 0.00 C

ATOM 672 O ASN 45 41.080 43.140 38.640 0.00 0.00 O

ATOM 673 N VAL 46 39.490 43.830 37.330 0.00 0.00 N

ATOM 674 H VAL 46 39.240 44.020 36.380 0.00 0.00 H

ATOM 675 CA VAL 46 38.340 43.920 38.270 0.00 0.00 C

ATOM 676 HA VAL 46 38.490 44.810 38.880 0.00 0.00 H

ATOM 677 CB VAL 46 37.060 44.250 37.440 0.00 0.00 C

ATOM 678 HB VAL 46 37.170 45.150 36.840 0.00 0.00 H

ATOM 679 CG1 VAL 46 36.630 43.170 36.450 0.00 0.00 C

ATOM 680 HG11 VAL 46 35.790 43.510 35.840 0.00 0.00 H

ATOM 681 HG12 VAL 46 37.380 42.740 35.790 0.00 0.00 H

ATOM 682 HG13 VAL 46 36.350 42.330 37.090 0.00 0.00 H

ATOM 683 CG2 VAL 46 35.790 44.530 38.370 0.00 0.00 C

ATOM 684 HG21 VAL 46 35.470 43.580 38.790 0.00 0.00 H

ATOM 685 HG22 VAL 46 36.260 45.150 39.130 0.00 0.00 H

ATOM 686 HG23 VAL 46 35.020 45.090 37.840 0.00 0.00 H

ATOM 687 C VAL 46 38.210 42.900 39.360 0.00 0.00 C

ATOM 688 O VAL 46 38.030 43.240 40.530 0.00 0.00 O

ATOM 689 N ALA 47 38.290 41.640 39.060 0.00 0.00 N

ATOM 690 H ALA 47 38.540 41.400 38.110 0.00 0.00 H

ATOM 691 CA ALA 47 38.210 40.560 40.030 0.00 0.00 C

ATOM 692 HA ALA 47 37.430 40.790 40.750 0.00 0.00 H

ATOM 693 CB ALA 47 37.920 39.330 39.180 0.00 0.00 C

ATOM 694 HB1 ALA 47 38.780 39.040 38.560 0.00 0.00 H

ATOM 695 HB2 ALA 47 37.720 38.520 39.870 0.00 0.00 H

ATOM 696 HB3 ALA 47 37.040 39.420 38.540 0.00 0.00 H

ATOM 697 C ALA 47 39.490 40.280 40.860 0.00 0.00 C

ATOM 698 O ALA 47 39.460 40.050 42.060 0.00 0.00 O

ATOM 699 N ASN 48 40.680 40.410 40.200 0.00 0.00 N

ATOM 700 H ASN 48 40.720 40.790 39.270 0.00 0.00 H

ATOM 701 CA ASN 48 41.970 40.230 40.820 0.00 0.00 C

ATOM 702 HA ASN 48 41.860 39.390 41.510 0.00 0.00 H

ATOM 703 CB ASN 48 43.160 39.880 39.820 0.00 0.00 C

ATOM 704 HB1 ASN 48 43.400 40.700 39.150 0.00 0.00 H

ATOM 705 HB2 ASN 48 42.820 39.040 39.210 0.00 0.00 H

ATOM 706 CG ASN 48 44.420 39.370 40.500 0.00 0.00 C

ATOM 707 OD1 ASN 48 44.240 38.360 41.210 0.00 0.00 O

ATOM 708 ND2 ASN 48 45.600 39.890 40.370 0.00 0.00 N

ATOM 709 HD21 ASN 48 45.670 40.860 40.100 0.00 0.00 H

ATOM 710 HD22 ASN 48 46.380 39.320 40.680 0.00 0.00 H

ATOM 711 C ASN 48 42.310 41.370 41.770 0.00 0.00 C

ATOM 712 O ASN 48 43.050 41.170 42.720 0.00 0.00 O

ATOM 713 N TYR 49 41.690 42.510 41.590 0.00 0.00 N

ATOM 714 H TYR 49 41.170 42.590 40.720 0.00 0.00 H

ATOM 715 CA TYR 49 41.760 43.630 42.600 0.00 0.00 C

ATOM 716 HA TYR 49 42.810 43.920 42.690 0.00 0.00 H

ATOM 717 CB TYR 49 40.950 44.860 42.220 0.00 0.00 C

ATOM 718 HB1 TYR 49 40.520 45.290 43.120 0.00 0.00 H

ATOM 719 HB2 TYR 49 40.140 44.440 41.620 0.00 0.00 H

ATOM 720 CG TYR 49 41.750 45.930 41.480 0.00 0.00 C

ATOM 721 CD1 TYR 49 42.770 46.550 42.170 0.00 0.00 C

ATOM 722 HD1 TYR 49 42.880 46.300 43.210 0.00 0.00 H

ATOM 723 CE1 TYR 49 43.550 47.480 41.540 0.00 0.00 C

ATOM 724 HE1 TYR 49 44.360 47.940 42.100 0.00 0.00 H

ATOM 725 CZ TYR 49 43.350 47.920 40.220 0.00 0.00 C

ATOM 726 OH TYR 49 44.140 48.890 39.630 0.00 0.00 O

ATOM 727 HH TYR 49 44.900 49.130 40.160 0.00 0.00 H

ATOM 728 CE2 TYR 49 42.220 47.350 39.540 0.00 0.00 C

ATOM 729 HE2 TYR 49 42.030 47.680 38.530 0.00 0.00 H

ATOM 730 CD2 TYR 49 41.400 46.410 40.220 0.00 0.00 C

ATOM 731 HD2 TYR 49 40.520 46.050 39.710 0.00 0.00 H

ATOM 732 C TYR 49 41.410 43.050 43.980 0.00 0.00 C

ATOM 733 O TYR 49 42.030 43.490 44.980 0.00 0.00 O

ATOM 734 N LEU 50 40.350 42.270 44.130 0.00 0.00 N

ATOM 735 H LEU 50 39.770 41.920 43.390 0.00 0.00 H

ATOM 736 CA LEU 50 39.940 41.570 45.390 0.00 0.00 C

ATOM 737 HA LEU 50 39.670 42.220 46.220 0.00 0.00 H

ATOM 738 CB LEU 50 38.680 40.680 45.120 0.00 0.00 C

ATOM 739 HB1 LEU 50 38.360 40.210 46.050 0.00 0.00 H

ATOM 740 HB2 LEU 50 38.890 39.810 44.500 0.00 0.00 H

ATOM 741 CG LEU 50 37.500 41.520 44.540 0.00 0.00 C

ATOM 742 HG LEU 50 37.860 41.960 43.620 0.00 0.00 H

ATOM 743 CD1 LEU 50 36.320 40.620 44.290 0.00 0.00 C

ATOM 744 HD11 LEU 50 36.290 39.900 45.110 0.00 0.00 H

ATOM 745 HD12 LEU 50 35.390 41.170 44.440 0.00 0.00 H

ATOM 746 HD13 LEU 50 36.370 40.200 43.290 0.00 0.00 H

ATOM 747 CD2 LEU 50 37.110 42.630 45.600 0.00 0.00 C

ATOM 748 HD21 LEU 50 37.930 43.260 45.950 0.00 0.00 H

ATOM 749 HD22 LEU 50 36.290 43.250 45.260 0.00 0.00 H

ATOM 750 HD23 LEU 50 36.730 42.160 46.510 0.00 0.00 H

ATOM 751 C LEU 50 41.030 40.700 45.980 0.00 0.00 C

ATOM 752 O LEU 50 41.340 40.910 47.170 0.00 0.00 O

ATOM 753 N ILE 51 41.770 39.930 45.180 0.00 0.00 N

ATOM 754 H ILE 51 41.540 39.840 44.200 0.00 0.00 H

ATOM 755 CA ILE 51 42.980 39.300 45.660 0.00 0.00 C

ATOM 756 HA ILE 51 42.950 38.640 46.530 0.00 0.00 H

ATOM 757 CB ILE 51 43.580 38.400 44.510 0.00 0.00 C

ATOM 758 HB ILE 51 43.600 39.070 43.640 0.00 0.00 H

ATOM 759 CG2 ILE 51 45.110 38.000 44.790 0.00 0.00 C

ATOM 760 HG21 ILE 51 45.700 38.890 44.540 0.00 0.00 H

ATOM 761 HG22 ILE 51 45.330 37.690 45.810 0.00 0.00 H

ATOM 762 HG23 ILE 51 45.390 37.190 44.130 0.00 0.00 H

ATOM 763 CG1 ILE 51 42.700 37.250 44.270 0.00 0.00 C

ATOM 764 HG11 ILE 51 43.180 36.850 43.380 0.00 0.00 H

ATOM 765 HG12 ILE 51 41.770 37.550 43.780 0.00 0.00 H

ATOM 766 CD ILE 51 42.520 36.210 45.340 0.00 0.00 C

ATOM 767 HD1 ILE 51 42.010 35.370 44.880 0.00 0.00 H

ATOM 768 HD2 ILE 51 43.430 35.820 45.800 0.00 0.00 H

ATOM 769 HD3 ILE 51 41.990 36.650 46.180 0.00 0.00 H

ATOM 770 C ILE 51 44.010 40.390 46.050 0.00 0.00 C

ATOM 771 O ILE 51 44.630 40.210 47.130 0.00 0.00 O

ATOM 772 N GLY 52 44.120 41.520 45.410 0.00 0.00 N

ATOM 773 H GLY 52 43.710 41.520 44.480 0.00 0.00 H

ATOM 774 CA GLY 52 44.980 42.690 45.820 0.00 0.00 C

ATOM 775 HA1 GLY 52 44.930 43.570 45.180 0.00 0.00 H

ATOM 776 HA2 GLY 52 46.020 42.410 45.660 0.00 0.00 H

ATOM 777 C GLY 52 44.640 43.200 47.210 0.00 0.00 C

ATOM 778 O GLY 52 45.490 43.380 48.060 0.00 0.00 O

ATOM 779 N SER 53 43.360 43.280 47.480 0.00 0.00 N

ATOM 780 H SER 53 42.720 43.100 46.720 0.00 0.00 H

ATOM 781 CA SER 53 42.940 43.800 48.760 0.00 0.00 C

ATOM 782 HA SER 53 43.650 44.560 49.080 0.00 0.00 H

ATOM 783 CB SER 53 41.530 44.290 48.700 0.00 0.00 C

ATOM 784 HB1 SER 53 40.820 43.480 48.520 0.00 0.00 H

ATOM 785 HB2 SER 53 41.500 45.070 47.940 0.00 0.00 H

ATOM 786 OG SER 53 41.190 44.790 49.920 0.00 0.00 O

ATOM 787 HG SER 53 40.250 44.600 50.010 0.00 0.00 H

ATOM 788 C SER 53 43.240 42.740 49.790 0.00 0.00 C

ATOM 789 O SER 53 43.920 43.060 50.790 0.00 0.00 O

ATOM 790 N LEU 54 42.960 41.460 49.510 0.00 0.00 N

ATOM 791 H LEU 54 42.610 41.280 48.580 0.00 0.00 H

ATOM 792 CA LEU 54 43.270 40.260 50.290 0.00 0.00 C

ATOM 793 HA LEU 54 42.900 40.420 51.300 0.00 0.00 H

ATOM 794 CB LEU 54 42.640 39.060 49.700 0.00 0.00 C

ATOM 795 HB1 LEU 54 42.950 38.840 48.680 0.00 0.00 H

ATOM 796 HB2 LEU 54 41.580 39.300 49.590 0.00 0.00 H

ATOM 797 CG LEU 54 42.740 37.760 50.500 0.00 0.00 C

ATOM 798 HG LEU 54 42.800 37.890 51.580 0.00 0.00 H

ATOM 799 CD1 LEU 54 41.540 36.800 50.170 0.00 0.00 C

ATOM 800 HD11 LEU 54 40.580 37.290 50.050 0.00 0.00 H

ATOM 801 HD12 LEU 54 41.630 36.390 49.160 0.00 0.00 H

ATOM 802 HD13 LEU 54 41.490 35.990 50.900 0.00 0.00 H

ATOM 803 CD2 LEU 54 44.030 36.990 50.130 0.00 0.00 C

ATOM 804 HD21 LEU 54 44.020 36.740 49.070 0.00 0.00 H

ATOM 805 HD22 LEU 54 44.980 37.520 50.230 0.00 0.00 H

ATOM 806 HD23 LEU 54 44.130 36.120 50.790 0.00 0.00 H

ATOM 807 C LEU 54 44.770 40.110 50.630 0.00 0.00 C

ATOM 808 O LEU 54 45.160 39.960 51.810 0.00 0.00 O

ATOM 809 N ALA 55 45.660 40.380 49.680 0.00 0.00 N

ATOM 810 H ALA 55 45.310 40.310 48.740 0.00 0.00 H

ATOM 811 CA ALA 55 47.130 40.370 49.880 0.00 0.00 C

ATOM 812 HA ALA 55 47.450 39.510 50.480 0.00 0.00 H

ATOM 813 CB ALA 55 47.800 40.140 48.530 0.00 0.00 C

ATOM 814 HB1 ALA 55 47.460 40.860 47.780 0.00 0.00 H

ATOM 815 HB2 ALA 55 48.810 40.430 48.790 0.00 0.00 H

ATOM 816 HB3 ALA 55 47.790 39.130 48.120 0.00 0.00 H

ATOM 817 C ALA 55 47.610 41.620 50.640 0.00 0.00 C

ATOM 818 O ALA 55 48.500 41.440 51.460 0.00 0.00 O

ATOM 819 N VAL 56 47.070 42.840 50.360 0.00 0.00 N

ATOM 820 H VAL 56 46.270 42.890 49.750 0.00 0.00 H

ATOM 821 CA VAL 56 47.430 43.990 51.170 0.00 0.00 C

ATOM 822 HA VAL 56 48.520 44.030 51.150 0.00 0.00 H

ATOM 823 CB VAL 56 46.830 45.190 50.550 0.00 0.00 C

ATOM 824 HB VAL 56 45.820 44.890 50.240 0.00 0.00 H

ATOM 825 CG1 VAL 56 46.720 46.400 51.490 0.00 0.00 C

ATOM 826 HG11 VAL 56 46.060 47.100 50.990 0.00 0.00 H

ATOM 827 HG12 VAL 56 46.240 46.080 52.410 0.00 0.00 H

ATOM 828 HG13 VAL 56 47.690 46.860 51.690 0.00 0.00 H

ATOM 829 CG2 VAL 56 47.490 45.640 49.230 0.00 0.00 C

ATOM 830 HG21 VAL 56 47.530 44.850 48.480 0.00 0.00 H

ATOM 831 HG22 VAL 56 46.900 46.360 48.670 0.00 0.00 H

ATOM 832 HG23 VAL 56 48.490 46.050 49.350 0.00 0.00 H

ATOM 833 C VAL 56 47.160 43.740 52.680 0.00 0.00 C

ATOM 834 O VAL 56 47.750 44.350 53.600 0.00 0.00 O

ATOM 835 N THR 57 46.230 42.840 53.000 0.00 0.00 N

ATOM 836 H THR 57 45.780 42.320 52.260 0.00 0.00 H

ATOM 837 CA THR 57 45.660 42.560 54.320 0.00 0.00 C

ATOM 838 HA THR 57 45.700 43.560 54.730 0.00 0.00 H

ATOM 839 CB THR 57 44.230 42.090 54.280 0.00 0.00 C

ATOM 840 HB THR 57 44.230 41.120 53.780 0.00 0.00 H

ATOM 841 CG2 THR 57 43.740 41.920 55.710 0.00 0.00 C

ATOM 842 HG21 THR 57 42.680 41.710 55.880 0.00 0.00 H

ATOM 843 HG22 THR 57 44.240 41.040 56.120 0.00 0.00 H

ATOM 844 HG23 THR 57 43.970 42.830 56.250 0.00 0.00 H

ATOM 845 OG1 THR 57 43.450 43.050 53.570 0.00 0.00 O

ATOM 846 HG1 THR 57 43.840 43.050 52.690 0.00 0.00 H

ATOM 847 C THR 57 46.660 41.600 54.940 0.00 0.00 C

ATOM 848 O THR 57 47.110 41.870 56.050 0.00 0.00 O

ATOM 849 N ASP 58 47.140 40.560 54.150 0.00 0.00 N

ATOM 850 H ASP 58 46.760 40.450 53.220 0.00 0.00 H

ATOM 851 CA ASP 58 48.210 39.660 54.490 0.00 0.00 C

ATOM 852 HA ASP 58 48.090 39.350 55.530 0.00 0.00 H

ATOM 853 CB ASP 58 48.160 38.320 53.610 0.00 0.00 C

ATOM 854 HB1 ASP 58 49.200 38.010 53.600 0.00 0.00 H

ATOM 855 HB2 ASP 58 47.950 38.580 52.570 0.00 0.00 H

ATOM 856 CG ASP 58 47.400 37.180 54.270 0.00 0.00 C

ATOM 857 OD1 ASP 58 46.490 36.680 53.600 0.00 0.00 O

ATOM 858 OD2 ASP 58 47.670 36.720 55.400 0.00 0.00 O1-

ATOM 859 C ASP 58 49.590 40.270 54.560 0.00 0.00 C

ATOM 860 O ASP 58 50.420 39.800 55.290 0.00 0.00 O

ATOM 861 N LEU 59 49.810 41.390 53.860 0.00 0.00 N

ATOM 862 H LEU 59 49.010 41.750 53.350 0.00 0.00 H

ATOM 863 CA LEU 59 50.930 42.350 54.090 0.00 0.00 C

ATOM 864 HA LEU 59 51.810 41.710 54.120 0.00 0.00 H

ATOM 865 CB LEU 59 50.970 43.380 52.910 0.00 0.00 C

ATOM 866 HB1 LEU 59 50.040 43.940 52.830 0.00 0.00 H

ATOM 867 HB2 LEU 59 51.130 42.720 52.060 0.00 0.00 H

ATOM 868 CG LEU 59 52.130 44.390 52.950 0.00 0.00 C

ATOM 869 HG LEU 59 52.010 45.070 53.800 0.00 0.00 H

ATOM 870 CD1 LEU 59 53.530 43.720 53.090 0.00 0.00 C

ATOM 871 HD11 LEU 59 53.650 43.170 54.020 0.00 0.00 H

ATOM 872 HD12 LEU 59 53.540 42.980 52.290 0.00 0.00 H

ATOM 873 HD13 LEU 59 54.300 44.460 52.900 0.00 0.00 H

ATOM 874 CD2 LEU 59 52.180 45.200 51.630 0.00 0.00 C

ATOM 875 HD21 LEU 59 52.590 44.670 50.770 0.00 0.00 H

ATOM 876 HD22 LEU 59 51.160 45.490 51.380 0.00 0.00 H

ATOM 877 HD23 LEU 59 52.790 46.100 51.730 0.00 0.00 H

ATOM 878 C LEU 59 50.760 43.030 55.470 0.00 0.00 C

ATOM 879 O LEU 59 51.660 42.930 56.280 0.00 0.00 O

ATOM 880 N MET 60 49.640 43.660 55.720 0.00 0.00 N

ATOM 881 H MET 60 49.030 43.780 54.930 0.00 0.00 H

ATOM 882 CA MET 60 49.250 44.290 57.000 0.00 0.00 C

ATOM 883 HA MET 60 50.010 45.050 57.180 0.00 0.00 H

ATOM 884 CB MET 60 47.870 45.100 56.960 0.00 0.00 C

ATOM 885 HB1 MET 60 47.050 44.430 56.690 0.00 0.00 H

ATOM 886 HB2 MET 60 47.770 45.870 56.190 0.00 0.00 H

ATOM 887 CG MET 60 47.540 45.950 58.200 0.00 0.00 C

ATOM 888 HG1 MET 60 48.450 46.530 58.350 0.00 0.00 H

ATOM 889 HG2 MET 60 47.480 45.320 59.090 0.00 0.00 H

ATOM 890 SD MET 60 46.150 47.010 58.170 0.00 0.00 S

ATOM 891 CE MET 60 44.710 45.980 57.950 0.00 0.00 C

ATOM 892 HE1 MET 60 45.000 45.000 58.350 0.00 0.00 H

ATOM 893 HE2 MET 60 43.900 46.440 58.520 0.00 0.00 H

ATOM 894 HE3 MET 60 44.530 45.790 56.890 0.00 0.00 H

ATOM 895 C MET 60 49.340 43.380 58.170 0.00 0.00 C

ATOM 896 O MET 60 49.740 43.920 59.240 0.00 0.00 O

ATOM 897 N VAL 61 49.090 42.110 57.950 0.00 0.00 N

ATOM 898 H VAL 61 48.450 41.920 57.190 0.00 0.00 H

ATOM 899 CA VAL 61 49.200 41.140 58.980 0.00 0.00 C

ATOM 900 HA VAL 61 48.900 41.530 59.950 0.00 0.00 H

ATOM 901 CB VAL 61 48.370 39.880 58.640 0.00 0.00 C

ATOM 902 HB VAL 61 48.590 39.640 57.610 0.00 0.00 H

ATOM 903 CG1 VAL 61 48.810 38.660 59.390 0.00 0.00 C

ATOM 904 HG11 VAL 61 49.830 38.360 59.140 0.00 0.00 H

ATOM 905 HG12 VAL 61 48.730 38.890 60.450 0.00 0.00 H

ATOM 906 HG13 VAL 61 48.210 37.780 59.180 0.00 0.00 H

ATOM 907 CG2 VAL 61 46.830 40.100 58.860 0.00 0.00 C

ATOM 908 HG21 VAL 61 46.200 39.250 58.590 0.00 0.00 H

ATOM 909 HG22 VAL 61 46.690 40.430 59.890 0.00 0.00 H

ATOM 910 HG23 VAL 61 46.560 41.000 58.310 0.00 0.00 H

ATOM 911 C VAL 61 50.660 40.830 59.260 0.00 0.00 C

ATOM 912 O VAL 61 51.100 41.000 60.410 0.00 0.00 O

ATOM 913 N SER 62 51.400 40.380 58.190 0.00 0.00 N

ATOM 914 H SER 62 50.890 40.000 57.410 0.00 0.00 H

ATOM 915 CA SER 62 52.860 40.220 58.150 0.00 0.00 C

ATOM 916 HA SER 62 53.070 39.310 58.720 0.00 0.00 H

ATOM 917 CB SER 62 53.440 39.970 56.780 0.00 0.00 C

ATOM 918 HB1 SER 62 54.510 39.840 56.890 0.00 0.00 H

ATOM 919 HB2 SER 62 53.230 40.810 56.120 0.00 0.00 H

ATOM 920 OG SER 62 52.910 38.830 56.150 0.00 0.00 O

ATOM 921 HG SER 62 52.130 39.150 55.680 0.00 0.00 H

ATOM 922 C SER 62 53.610 41.270 58.940 0.00 0.00 C

ATOM 923 O SER 62 54.290 41.070 59.950 0.00 0.00 O

ATOM 924 N VAL 63 53.440 42.530 58.470 0.00 0.00 N

ATOM 925 H VAL 63 52.740 42.690 57.760 0.00 0.00 H

ATOM 926 CA VAL 63 54.130 43.670 58.980 0.00 0.00 C

ATOM 927 HA VAL 63 55.220 43.590 58.970 0.00 0.00 H

ATOM 928 CB VAL 63 53.910 44.880 58.020 0.00 0.00 C

ATOM 929 HB VAL 63 52.900 44.830 57.620 0.00 0.00 H

ATOM 930 CG1 VAL 63 54.200 46.300 58.600 0.00 0.00 C

ATOM 931 HG11 VAL 63 54.260 47.060 57.820 0.00 0.00 H

ATOM 932 HG12 VAL 63 53.420 46.690 59.240 0.00 0.00 H

ATOM 933 HG13 VAL 63 55.150 46.290 59.140 0.00 0.00 H

ATOM 934 CG2 VAL 63 54.960 44.770 56.860 0.00 0.00 C

ATOM 935 HG21 VAL 63 55.960 44.700 57.270 0.00 0.00 H

ATOM 936 HG22 VAL 63 54.730 43.910 56.240 0.00 0.00 H

ATOM 937 HG23 VAL 63 54.900 45.610 56.160 0.00 0.00 H

ATOM 938 C VAL 63 53.760 44.050 60.480 0.00 0.00 C

ATOM 939 O VAL 63 54.690 44.410 61.180 0.00 0.00 O

ATOM 940 N LEU 64 52.490 43.920 60.910 0.00 0.00 N

ATOM 941 H LEU 64 51.820 43.620 60.210 0.00 0.00 H

ATOM 942 CA LEU 64 52.060 44.280 62.240 0.00 0.00 C

ATOM 943 HA LEU 64 52.940 44.670 62.750 0.00 0.00 H

ATOM 944 CB LEU 64 50.930 45.340 62.190 0.00 0.00 C

ATOM 945 HB1 LEU 64 50.550 45.570 63.180 0.00 0.00 H

ATOM 946 HB2 LEU 64 50.160 44.890 61.560 0.00 0.00 H

ATOM 947 CG LEU 64 51.350 46.600 61.450 0.00 0.00 C

ATOM 948 HG LEU 64 51.700 46.450 60.430 0.00 0.00 H

ATOM 949 CD1 LEU 64 50.140 47.560 61.380 0.00 0.00 C

ATOM 950 HD11 LEU 64 49.310 47.160 60.800 0.00 0.00 H

ATOM 951 HD12 LEU 64 49.720 47.660 62.390 0.00 0.00 H

ATOM 952 HD13 LEU 64 50.380 48.540 60.990 0.00 0.00 H

ATOM 953 CD2 LEU 64 52.520 47.390 62.170 0.00 0.00 C

ATOM 954 HD21 LEU 64 53.470 46.870 62.040 0.00 0.00 H

ATOM 955 HD22 LEU 64 52.630 48.400 61.760 0.00 0.00 H

ATOM 956 HD23 LEU 64 52.280 47.480 63.230 0.00 0.00 H

ATOM 957 C LEU 64 51.690 43.090 63.080 0.00 0.00 C

ATOM 958 O LEU 64 52.070 43.140 64.240 0.00 0.00 O

ATOM 959 N VAL 65 50.710 42.280 62.780 0.00 0.00 N

ATOM 960 H VAL 65 50.300 42.300 61.860 0.00 0.00 H

ATOM 961 CA VAL 65 50.130 41.270 63.690 0.00 0.00 C

ATOM 962 HA VAL 65 49.750 41.830 64.550 0.00 0.00 H

ATOM 963 CB VAL 65 48.930 40.500 63.020 0.00 0.00 C

ATOM 964 HB VAL 65 49.290 40.070 62.090 0.00 0.00 H

ATOM 965 CG1 VAL 65 48.510 39.330 63.940 0.00 0.00 C

ATOM 966 HG11 VAL 65 48.470 39.760 64.940 0.00 0.00 H

ATOM 967 HG12 VAL 65 47.510 38.970 63.680 0.00 0.00 H

ATOM 968 HG13 VAL 65 49.240 38.530 63.910 0.00 0.00 H

ATOM 969 CG2 VAL 65 47.730 41.460 62.760 0.00 0.00 C

ATOM 970 HG21 VAL 65 48.040 42.240 62.060 0.00 0.00 H

ATOM 971 HG22 VAL 65 47.030 40.840 62.190 0.00 0.00 H

ATOM 972 HG23 VAL 65 47.200 41.960 63.570 0.00 0.00 H

ATOM 973 C VAL 65 51.240 40.220 63.990 0.00 0.00 C

ATOM 974 O VAL 65 51.540 40.040 65.190 0.00 0.00 O

ATOM 975 N LEU 66 52.000 39.750 62.990 0.00 0.00 N

ATOM 976 H LEU 66 51.760 40.090 62.070 0.00 0.00 H

ATOM 977 CA LEU 66 53.020 38.720 63.200 0.00 0.00 C

ATOM 978 HA LEU 66 52.570 37.930 63.810 0.00 0.00 H

ATOM 979 CB LEU 66 53.550 38.000 62.000 0.00 0.00 C

ATOM 980 HB1 LEU 66 54.190 37.210 62.410 0.00 0.00 H

ATOM 981 HB2 LEU 66 54.150 38.630 61.350 0.00 0.00 H

ATOM 982 CG LEU 66 52.430 37.450 61.130 0.00 0.00 C

ATOM 983 HG LEU 66 51.760 38.240 60.800 0.00 0.00 H

ATOM 984 CD1 LEU 66 53.090 36.760 59.940 0.00 0.00 C

ATOM 985 HD11 LEU 66 52.350 36.460 59.190 0.00 0.00 H

ATOM 986 HD12 LEU 66 53.860 37.410 59.540 0.00 0.00 H

ATOM 987 HD13 LEU 66 53.620 35.830 60.140 0.00 0.00 H

ATOM 988 CD2 LEU 66 51.540 36.450 61.890 0.00 0.00 C

ATOM 989 HD21 LEU 66 50.730 36.110 61.250 0.00 0.00 H

ATOM 990 HD22 LEU 66 52.110 35.600 62.260 0.00 0.00 H

ATOM 991 HD23 LEU 66 51.040 37.060 62.650 0.00 0.00 H

ATOM 992 C LEU 66 54.080 39.140 64.200 0.00 0.00 C

ATOM 993 O LEU 66 54.210 38.410 65.180 0.00 0.00 O

ATOM 994 N PRO 67 54.800 40.300 64.000 0.00 0.00 N

ATOM 995 CD PRO 67 54.900 41.050 62.740 0.00 0.00 C

ATOM 996 HD1 PRO 67 53.910 41.330 62.360 0.00 0.00 H

ATOM 997 HD2 PRO 67 55.440 40.410 62.040 0.00 0.00 H

ATOM 998 CG PRO 67 55.650 42.370 63.030 0.00 0.00 C

ATOM 999 HG1 PRO 67 54.980 43.140 63.410 0.00 0.00 H

ATOM 1000 HG2 PRO 67 56.120 42.670 62.100 0.00 0.00 H

ATOM 1001 CB PRO 67 56.650 41.910 64.080 0.00 0.00 C

ATOM 1002 HB1 PRO 67 56.820 42.750 64.760 0.00 0.00 H

ATOM 1003 HB2 PRO 67 57.540 41.560 63.560 0.00 0.00 H

ATOM 1004 CA PRO 67 55.960 40.760 64.830 0.00 0.00 C

ATOM 1005 HA PRO 67 56.710 39.970 64.900 0.00 0.00 H

ATOM 1006 C PRO 67 55.480 40.990 66.290 0.00 0.00 C

ATOM 1007 O PRO 67 56.280 40.840 67.210 0.00 0.00 O

ATOM 1008 N MET 68 54.210 41.410 66.460 0.00 0.00 N

ATOM 1009 H MET 68 53.670 41.770 65.680 0.00 0.00 H

ATOM 1010 CA MET 68 53.690 41.690 67.790 0.00 0.00 C

ATOM 1011 HA MET 68 54.500 42.170 68.350 0.00 0.00 H

ATOM 1012 CB MET 68 52.600 42.740 67.710 0.00 0.00 C

ATOM 1013 HB1 MET 68 52.060 42.810 68.660 0.00 0.00 H

ATOM 1014 HB2 MET 68 51.780 42.320 67.120 0.00 0.00 H

ATOM 1015 CG MET 68 53.120 44.100 67.240 0.00 0.00 C

ATOM 1016 HG1 MET 68 52.320 44.670 66.760 0.00 0.00 H

ATOM 1017 HG2 MET 68 53.830 43.930 66.440 0.00 0.00 H

ATOM 1018 SD MET 68 53.960 44.930 68.610 0.00 0.00 S

ATOM 1019 CE MET 68 55.750 44.870 68.100 0.00 0.00 C

ATOM 1020 HE1 MET 68 56.180 43.900 67.860 0.00 0.00 H

ATOM 1021 HE2 MET 68 55.860 45.580 67.280 0.00 0.00 H

ATOM 1022 HE3 MET 68 56.380 45.200 68.930 0.00 0.00 H

ATOM 1023 C MET 68 53.310 40.330 68.440 0.00 0.00 C

ATOM 1024 O MET 68 53.550 40.150 69.580 0.00 0.00 O

ATOM 1025 N ALA 69 52.800 39.420 67.640 0.00 0.00 N

ATOM 1026 H ALA 69 52.540 39.750 66.730 0.00 0.00 H

ATOM 1027 CA ALA 69 52.450 38.060 68.020 0.00 0.00 C

ATOM 1028 HA ALA 69 51.810 38.130 68.900 0.00 0.00 H

ATOM 1029 CB ALA 69 51.750 37.290 66.940 0.00 0.00 C

ATOM 1030 HB1 ALA 69 52.420 36.820 66.220 0.00 0.00 H

ATOM 1031 HB2 ALA 69 51.180 36.480 67.390 0.00 0.00 H

ATOM 1032 HB3 ALA 69 50.950 37.950 66.570 0.00 0.00 H

ATOM 1033 C ALA 69 53.710 37.290 68.460 0.00 0.00 C

ATOM 1034 O ALA 69 53.640 36.650 69.460 0.00 0.00 O

ATOM 1035 N ALA 70 54.820 37.450 67.730 0.00 0.00 N

ATOM 1036 H ALA 70 54.680 37.870 66.820 0.00 0.00 H

ATOM 1037 CA ALA 70 56.140 36.990 68.060 0.00 0.00 C

ATOM 1038 HA ALA 70 56.210 35.910 68.200 0.00 0.00 H

ATOM 1039 CB ALA 70 56.940 37.380 66.780 0.00 0.00 C

ATOM 1040 HB1 ALA 70 58.000 37.320 67.050 0.00 0.00 H

ATOM 1041 HB2 ALA 70 56.750 36.650 66.000 0.00 0.00 H

ATOM 1042 HB3 ALA 70 56.670 38.370 66.420 0.00 0.00 H

ATOM 1043 C ALA 70 56.680 37.580 69.390 0.00 0.00 C

ATOM 1044 O ALA 70 57.110 36.840 70.250 0.00 0.00 O

ATOM 1045 N LEU 71 56.840 38.900 69.480 0.00 0.00 N

ATOM 1046 H LEU 71 56.480 39.430 68.700 0.00 0.00 H

ATOM 1047 CA LEU 71 57.330 39.610 70.680 0.00 0.00 C

ATOM 1048 HA LEU 71 58.360 39.280 70.740 0.00 0.00 H

ATOM 1049 CB LEU 71 57.260 41.140 70.320 0.00 0.00 C

ATOM 1050 HB1 LEU 71 56.290 41.430 69.930 0.00 0.00 H

ATOM 1051 HB2 LEU 71 58.000 41.380 69.560 0.00 0.00 H

ATOM 1052 CG LEU 71 57.560 42.040 71.520 0.00 0.00 C

ATOM 1053 HG LEU 71 56.890 41.810 72.340 0.00 0.00 H

ATOM 1054 CD1 LEU 71 59.000 41.850 71.980 0.00 0.00 C

ATOM 1055 HD11 LEU 71 59.630 42.130 71.140 0.00 0.00 H

ATOM 1056 HD12 LEU 71 59.230 42.540 72.800 0.00 0.00 H

ATOM 1057 HD13 LEU 71 59.160 40.810 72.270 0.00 0.00 H

ATOM 1058 CD2 LEU 71 57.360 43.470 71.150 0.00 0.00 C

ATOM 1059 HD21 LEU 71 57.510 44.040 72.060 0.00 0.00 H

ATOM 1060 HD22 LEU 71 58.040 43.780 70.350 0.00 0.00 H

ATOM 1061 HD23 LEU 71 56.370 43.630 70.710 0.00 0.00 H

ATOM 1062 C LEU 71 56.560 39.190 71.960 0.00 0.00 C

ATOM 1063 O LEU 71 57.180 39.060 73.030 0.00 0.00 O

ATOM 1064 N TYR 72 55.190 39.030 71.840 0.00 0.00 N

ATOM 1065 H TYR 72 54.680 39.370 71.040 0.00 0.00 H

ATOM 1066 CA TYR 72 54.450 38.390 72.890 0.00 0.00 C

ATOM 1067 HA TYR 72 54.660 38.860 73.860 0.00 0.00 H

ATOM 1068 CB TYR 72 52.960 38.590 72.440 0.00 0.00 C

ATOM 1069 HB1 TYR 72 52.780 38.130 71.470 0.00 0.00 H

ATOM 1070 HB2 TYR 72 52.820 39.670 72.300 0.00 0.00 H

ATOM 1071 CG TYR 72 51.890 37.880 73.310 0.00 0.00 C

ATOM 1072 CD1 TYR 72 50.770 37.270 72.700 0.00 0.00 C

ATOM 1073 HD1 TYR 72 50.660 37.230 71.630 0.00 0.00 H

ATOM 1074 CE1 TYR 72 49.760 36.720 73.510 0.00 0.00 C

ATOM 1075 HE1 TYR 72 48.950 36.160 73.080 0.00 0.00 H

ATOM 1076 CZ TYR 72 49.820 36.750 74.920 0.00 0.00 C

ATOM 1077 OH TYR 72 48.900 36.110 75.670 0.00 0.00 O

ATOM 1078 HH TYR 72 48.170 35.740 75.170 0.00 0.00 H

ATOM 1079 CE2 TYR 72 50.990 37.180 75.500 0.00 0.00 C

ATOM 1080 HE2 TYR 72 51.110 37.160 76.570 0.00 0.00 H

ATOM 1081 CD2 TYR 72 52.010 37.760 74.690 0.00 0.00 C

ATOM 1082 HD2 TYR 72 52.900 38.120 75.170 0.00 0.00 H

ATOM 1083 C TYR 72 54.740 36.880 73.090 0.00 0.00 C

ATOM 1084 O TYR 72 54.780 36.520 74.230 0.00 0.00 O

ATOM 1085 N GLN 73 54.950 36.070 72.060 0.00 0.00 N

ATOM 1086 H GLN 73 54.930 36.510 71.150 0.00 0.00 H

ATOM 1087 CA GLN 73 55.160 34.610 72.180 0.00 0.00 C

ATOM 1088 HA GLN 73 54.640 34.210 73.050 0.00 0.00 H

ATOM 1089 CB GLN 73 54.660 33.930 70.970 0.00 0.00 C

ATOM 1090 HB1 GLN 73 55.140 32.950 70.960 0.00 0.00 H

ATOM 1091 HB2 GLN 73 54.920 34.400 70.020 0.00 0.00 H

ATOM 1092 CG GLN 73 53.150 33.630 70.980 0.00 0.00 C

ATOM 1093 HG1 GLN 73 52.700 34.620 71.080 0.00 0.00 H

ATOM 1094 HG2 GLN 73 52.850 33.140 71.910 0.00 0.00 H

ATOM 1095 CD GLN 73 52.580 32.960 69.700 0.00 0.00 C

ATOM 1096 OE1 GLN 73 52.930 31.840 69.310 0.00 0.00 O

ATOM 1097 NE2 GLN 73 51.680 33.670 69.090 0.00 0.00 N

ATOM 1098 HE21 GLN 73 51.400 34.550 69.510 0.00 0.00 H

ATOM 1099 HE22 GLN 73 51.160 33.340 68.290 0.00 0.00 H

ATOM 1100 C GLN 73 56.580 34.190 72.500 0.00 0.00 C

ATOM 1101 O GLN 73 56.830 33.490 73.510 0.00 0.00 O

ATOM 1102 N VAL 74 57.540 34.800 71.820 0.00 0.00 N

ATOM 1103 H VAL 74 57.270 35.520 71.160 0.00 0.00 H

ATOM 1104 CA VAL 74 59.000 34.480 72.040 0.00 0.00 C

ATOM 1105 HA VAL 74 59.140 33.410 71.910 0.00 0.00 H

ATOM 1106 CB VAL 74 59.840 35.200 70.890 0.00 0.00 C

ATOM 1107 HB VAL 74 59.640 36.270 70.820 0.00 0.00 H

ATOM 1108 CG1 VAL 74 61.400 35.030 71.080 0.00 0.00 C

ATOM 1109 HG11 VAL 74 61.950 35.470 70.250 0.00 0.00 H

ATOM 1110 HG12 VAL 74 61.670 35.520 72.020 0.00 0.00 H

ATOM 1111 HG13 VAL 74 61.610 33.960 71.110 0.00 0.00 H

ATOM 1112 CG2 VAL 74 59.490 34.610 69.510 0.00 0.00 C

ATOM 1113 HG21 VAL 74 59.800 33.560 69.430 0.00 0.00 H

ATOM 1114 HG22 VAL 74 58.440 34.720 69.240 0.00 0.00 H

ATOM 1115 HG23 VAL 74 60.120 35.070 68.750 0.00 0.00 H

ATOM 1116 C VAL 74 59.510 34.860 73.450 0.00 0.00 C

ATOM 1117 O VAL 74 60.410 34.260 73.990 0.00 0.00 O

ATOM 1118 N LEU 75 59.020 36.030 74.020 0.00 0.00 N

ATOM 1119 H LEU 75 58.400 36.590 73.460 0.00 0.00 H

ATOM 1120 CA LEU 75 59.420 36.540 75.370 0.00 0.00 C

ATOM 1121 HA LEU 75 60.400 36.130 75.580 0.00 0.00 H

ATOM 1122 CB LEU 75 59.500 38.070 75.330 0.00 0.00 C

ATOM 1123 HB1 LEU 75 58.550 38.530 75.030 0.00 0.00 H

ATOM 1124 HB2 LEU 75 60.200 38.230 74.500 0.00 0.00 H

ATOM 1125 CG LEU 75 59.990 38.750 76.620 0.00 0.00 C

ATOM 1126 HG LEU 75 59.250 38.800 77.420 0.00 0.00 H

ATOM 1127 CD1 LEU 75 61.280 38.100 77.110 0.00 0.00 C

ATOM 1128 HD11 LEU 75 61.070 37.210 77.710 0.00 0.00 H

ATOM 1129 HD12 LEU 75 61.890 37.850 76.240 0.00 0.00 H

ATOM 1130 HD13 LEU 75 61.850 38.830 77.680 0.00 0.00 H

ATOM 1131 CD2 LEU 75 60.300 40.190 76.210 0.00 0.00 C

ATOM 1132 HD21 LEU 75 60.680 40.770 77.050 0.00 0.00 H

ATOM 1133 HD22 LEU 75 60.860 40.300 75.280 0.00 0.00 H

ATOM 1134 HD23 LEU 75 59.330 40.610 75.940 0.00 0.00 H

ATOM 1135 C LEU 75 58.420 36.110 76.470 0.00 0.00 C

ATOM 1136 O LEU 75 58.650 36.270 77.670 0.00 0.00 O

ATOM 1137 N ASN 76 57.230 35.770 76.040 0.00 0.00 N

ATOM 1138 H ASN 76 57.120 35.770 75.040 0.00 0.00 H

ATOM 1139 CA ASN 76 56.110 35.110 76.780 0.00 0.00 C

ATOM 1140 HA ASN 76 55.400 34.760 76.030 0.00 0.00 H

ATOM 1141 CB ASN 76 56.640 33.920 77.610 0.00 0.00 C

ATOM 1142 HB1 ASN 76 57.170 34.350 78.450 0.00 0.00 H

ATOM 1143 HB2 ASN 76 57.300 33.220 77.100 0.00 0.00 H

ATOM 1144 CG ASN 76 55.550 33.040 78.120 0.00 0.00 C

ATOM 1145 OD1 ASN 76 54.400 33.130 77.750 0.00 0.00 O

ATOM 1146 ND2 ASN 76 55.790 32.020 78.920 0.00 0.00 N

ATOM 1147 HD21 ASN 76 56.750 31.970 79.240 0.00 0.00 H

ATOM 1148 HD22 ASN 76 55.080 31.310 78.990 0.00 0.00 H

ATOM 1149 C ASN 76 55.290 36.080 77.660 0.00 0.00 C

ATOM 1150 O ASN 76 54.070 35.860 77.720 0.00 0.00 O

ATOM 1151 N LYS 77 55.880 37.080 78.250 0.00 0.00 N

ATOM 1152 H LYS 77 56.870 37.170 78.060 0.00 0.00 H

ATOM 1153 CA LYS 77 55.240 38.200 78.910 0.00 0.00 C

ATOM 1154 HA LYS 77 54.300 37.810 79.310 0.00 0.00 H

ATOM 1155 CB LYS 77 56.220 38.710 79.960 0.00 0.00 C

ATOM 1156 HB1 LYS 77 56.980 39.290 79.440 0.00 0.00 H

ATOM 1157 HB2 LYS 77 56.810 37.890 80.370 0.00 0.00 H

ATOM 1158 CG LYS 77 55.640 39.510 81.110 0.00 0.00 C

ATOM 1159 HG1 LYS 77 55.020 38.830 81.700 0.00 0.00 H

ATOM 1160 HG2 LYS 77 55.000 40.290 80.690 0.00 0.00 H

ATOM 1161 CD LYS 77 56.680 40.190 82.010 0.00 0.00 C

ATOM 1162 HD1 LYS 77 57.590 40.530 81.520 0.00 0.00 H

ATOM 1163 HD2 LYS 77 57.000 39.410 82.710 0.00 0.00 H

ATOM 1164 CE LYS 77 56.030 41.330 82.840 0.00 0.00 C

ATOM 1165 HE1 LYS 77 56.810 41.660 83.520 0.00 0.00 H

ATOM 1166 HE2 LYS 77 55.250 40.830 83.420 0.00 0.00 H

ATOM 1167 NZ LYS 77 55.500 42.410 81.970 0.00 0.00 N1+

ATOM 1168 HZ1 LYS 77 55.210 42.010 81.090 0.00 0.00 H

ATOM 1169 HZ2 LYS 77 54.690 42.930 82.290 0.00 0.00 H

ATOM 1170 HZ3 LYS 77 56.230 43.050 81.700 0.00 0.00 H

ATOM 1171 C LYS 77 54.910 39.270 77.900 0.00 0.00 C

ATOM 1172 O LYS 77 55.720 39.550 77.000 0.00 0.00 O

ATOM 1173 N TRP 78 53.740 39.880 78.040 0.00 0.00 N

ATOM 1174 H TRP 78 53.240 39.640 78.880 0.00 0.00 H

ATOM 1175 CA TRP 78 53.290 41.080 77.370 0.00 0.00 C

ATOM 1176 HA TRP 78 53.660 41.010 76.350 0.00 0.00 H

ATOM 1177 CB TRP 78 51.680 41.090 77.360 0.00 0.00 C

ATOM 1178 HB1 TRP 78 51.280 41.290 78.350 0.00 0.00 H

ATOM 1179 HB2 TRP 78 51.340 40.220 76.810 0.00 0.00 H

ATOM 1180 CG TRP 78 51.270 42.250 76.480 0.00 0.00 C

ATOM 1181 CD1 TRP 78 50.900 43.520 76.880 0.00 0.00 C

ATOM 1182 HD1 TRP 78 50.830 43.930 77.880 0.00 0.00 H

ATOM 1183 NE1 TRP 78 50.730 44.350 75.750 0.00 0.00 N

ATOM 1184 HE1 TRP 78 50.460 45.330 75.770 0.00 0.00 H

ATOM 1185 CE2 TRP 78 50.740 43.580 74.650 0.00 0.00 C

ATOM 1186 CZ2 TRP 78 50.450 43.950 73.290 0.00 0.00 C

ATOM 1187 HZ2 TRP 78 50.180 44.970 73.030 0.00 0.00 H

ATOM 1188 CH2 TRP 78 50.510 43.020 72.240 0.00 0.00 C

ATOM 1189 HH2 TRP 78 50.120 43.250 71.260 0.00 0.00 H

ATOM 1190 CZ3 TRP 78 50.800 41.700 72.600 0.00 0.00 C

ATOM 1191 HZ3 TRP 78 50.840 40.980 71.800 0.00 0.00 H

ATOM 1192 CE3 TRP 78 51.050 41.330 73.920 0.00 0.00 C

ATOM 1193 HE3 TRP 78 51.480 40.380 74.190 0.00 0.00 H

ATOM 1194 CD2 TRP 78 51.080 42.300 75.020 0.00 0.00 C

ATOM 1195 C TRP 78 54.010 42.320 78.060 0.00 0.00 C

ATOM 1196 O TRP 78 54.310 42.190 79.250 0.00 0.00 O

ATOM 1197 N THR 79 54.380 43.350 77.310 0.00 0.00 N

ATOM 1198 H THR 79 54.230 43.090 76.340 0.00 0.00 H

ATOM 1199 CA THR 79 55.230 44.480 77.710 0.00 0.00 C

ATOM 1200 HA THR 79 55.120 44.590 78.790 0.00 0.00 H

ATOM 1201 CB THR 79 56.680 44.230 77.340 0.00 0.00 C

ATOM 1202 HB THR 79 57.220 45.120 77.660 0.00 0.00 H

ATOM 1203 CG2 THR 79 57.250 42.940 77.920 0.00 0.00 C

ATOM 1204 HG21 THR 79 56.810 42.660 78.880 0.00 0.00 H

ATOM 1205 HG22 THR 79 57.200 42.080 77.250 0.00 0.00 H

ATOM 1206 HG23 THR 79 58.300 43.160 78.070 0.00 0.00 H

ATOM 1207 OG1 THR 79 56.660 44.100 75.970 0.00 0.00 O

ATOM 1208 HG1 THR 79 56.780 43.220 75.610 0.00 0.00 H

ATOM 1209 C THR 79 54.840 45.800 77.170 0.00 0.00 C

ATOM 1210 O THR 79 55.170 46.810 77.810 0.00 0.00 O

ATOM 1211 N LEU 80 54.030 45.880 76.070 0.00 0.00 N

ATOM 1212 H LEU 80 53.930 45.010 75.570 0.00 0.00 H

ATOM 1213 CA LEU 80 53.710 47.180 75.400 0.00 0.00 C

ATOM 1214 HA LEU 80 54.500 47.910 75.560 0.00 0.00 H

ATOM 1215 CB LEU 80 53.620 46.950 73.920 0.00 0.00 C

ATOM 1216 HB1 LEU 80 53.660 47.850 73.320 0.00 0.00 H

ATOM 1217 HB2 LEU 80 52.750 46.320 73.760 0.00 0.00 H

ATOM 1218 CG LEU 80 54.750 46.010 73.420 0.00 0.00 C

ATOM 1219 HG LEU 80 54.780 44.980 73.790 0.00 0.00 H

ATOM 1220 CD1 LEU 80 54.630 45.910 71.880 0.00 0.00 C

ATOM 1221 HD11 LEU 80 54.560 46.890 71.390 0.00 0.00 H

ATOM 1222 HD12 LEU 80 55.460 45.400 71.390 0.00 0.00 H

ATOM 1223 HD13 LEU 80 53.650 45.520 71.620 0.00 0.00 H

ATOM 1224 CD2 LEU 80 56.070 46.690 73.660 0.00 0.00 C

ATOM 1225 HD21 LEU 80 56.890 46.330 73.040 0.00 0.00 H

ATOM 1226 HD22 LEU 80 56.020 47.740 73.370 0.00 0.00 H

ATOM 1227 HD23 LEU 80 56.400 46.440 74.670 0.00 0.00 H

ATOM 1228 C LEU 80 52.450 47.910 75.940 0.00 0.00 C

ATOM 1229 O LEU 80 51.860 48.800 75.380 0.00 0.00 O

ATOM 1230 N GLY 81 51.830 47.480 77.010 0.00 0.00 N

ATOM 1231 H GLY 81 52.270 46.700 77.480 0.00 0.00 H

ATOM 1232 CA GLY 81 50.630 48.090 77.560 0.00 0.00 C

ATOM 1233 HA1 GLY 81 50.640 49.180 77.460 0.00 0.00 H

ATOM 1234 HA2 GLY 81 50.730 47.880 78.620 0.00 0.00 H

ATOM 1235 C GLY 81 49.260 47.550 77.020 0.00 0.00 C

ATOM 1236 O GLY 81 49.150 46.820 76.020 0.00 0.00 O

ATOM 1237 N GLN 82 48.220 48.010 77.720 0.00 0.00 N

ATOM 1238 H GLN 82 48.350 48.670 78.480 0.00 0.00 H

ATOM 1239 CA GLN 82 46.840 47.500 77.440 0.00 0.00 C

ATOM 1240 HA GLN 82 46.920 46.440 77.220 0.00 0.00 H

ATOM 1241 CB GLN 82 45.920 47.700 78.690 0.00 0.00 C

ATOM 1242 HB1 GLN 82 45.770 48.770 78.850 0.00 0.00 H

ATOM 1243 HB2 GLN 82 46.430 47.320 79.570 0.00 0.00 H

ATOM 1244 CG GLN 82 44.470 47.110 78.550 0.00 0.00 C

ATOM 1245 HG1 GLN 82 43.990 47.520 77.660 0.00 0.00 H

ATOM 1246 HG2 GLN 82 43.810 47.380 79.370 0.00 0.00 H

ATOM 1247 CD GLN 82 44.360 45.560 78.530 0.00 0.00 C

ATOM 1248 OE1 GLN 82 44.630 44.860 79.490 0.00 0.00 O

ATOM 1249 NE2 GLN 82 44.030 45.060 77.380 0.00 0.00 N

ATOM 1250 HE21 GLN 82 43.710 45.550 76.560 0.00 0.00 H

ATOM 1251 HE22 GLN 82 44.070 44.050 77.390 0.00 0.00 H

ATOM 1252 C GLN 82 46.230 48.140 76.190 0.00 0.00 C

ATOM 1253 O GLN 82 45.710 47.290 75.450 0.00 0.00 O

ATOM 1254 N VAL 83 46.470 49.430 75.990 0.00 0.00 N

ATOM 1255 H VAL 83 46.940 49.890 76.750 0.00 0.00 H

ATOM 1256 CA VAL 83 45.920 49.990 74.770 0.00 0.00 C

ATOM 1257 HA VAL 83 44.850 49.780 74.690 0.00 0.00 H

ATOM 1258 CB VAL 83 46.090 51.550 74.770 0.00 0.00 C

ATOM 1259 HB VAL 83 45.900 51.880 75.790 0.00 0.00 H

ATOM 1260 CG1 VAL 83 47.550 52.000 74.550 0.00 0.00 C

ATOM 1261 HG11 VAL 83 48.360 51.550 75.120 0.00 0.00 H

ATOM 1262 HG12 VAL 83 47.820 51.940 73.500 0.00 0.00 H

ATOM 1263 HG13 VAL 83 47.600 53.050 74.850 0.00 0.00 H

ATOM 1264 CG2 VAL 83 45.190 52.210 73.740 0.00 0.00 C

ATOM 1265 HG21 VAL 83 44.160 51.860 73.750 0.00 0.00 H

ATOM 1266 HG22 VAL 83 45.300 53.290 73.830 0.00 0.00 H

ATOM 1267 HG23 VAL 83 45.640 51.980 72.770 0.00 0.00 H

ATOM 1268 C VAL 83 46.520 49.490 73.420 0.00 0.00 C

ATOM 1269 O VAL 83 45.880 49.380 72.340 0.00 0.00 O

ATOM 1270 N THR 84 47.750 48.940 73.530 0.00 0.00 N

ATOM 1271 H THR 84 48.240 49.110 74.400 0.00 0.00 H

ATOM 1272 CA THR 84 48.460 48.170 72.520 0.00 0.00 C

ATOM 1273 HA THR 84 48.380 48.640 71.540 0.00 0.00 H

ATOM 1274 CB THR 84 49.960 48.150 72.900 0.00 0.00 C

ATOM 1275 HB THR 84 50.090 47.370 73.640 0.00 0.00 H

ATOM 1276 CG2 THR 84 50.860 47.710 71.680 0.00 0.00 C

ATOM 1277 HG21 THR 84 50.790 46.710 71.260 0.00 0.00 H

ATOM 1278 HG22 THR 84 50.560 48.360 70.850 0.00 0.00 H

ATOM 1279 HG23 THR 84 51.910 47.840 71.930 0.00 0.00 H

ATOM 1280 OG1 THR 84 50.370 49.400 73.320 0.00 0.00 O

ATOM 1281 HG1 THR 84 51.000 49.190 74.020 0.00 0.00 H

ATOM 1282 C THR 84 47.950 46.720 72.440 0.00 0.00 C

ATOM 1283 O THR 84 47.930 46.130 71.400 0.00 0.00 O

ATOM 1284 N CYS 85 47.570 46.130 73.550 0.00 0.00 N

ATOM 1285 H CYS 85 47.570 46.630 74.430 0.00 0.00 H

ATOM 1286 CA CYS 85 46.870 44.790 73.530 0.00 0.00 C

ATOM 1287 HA CYS 85 47.490 44.080 72.970 0.00 0.00 H

ATOM 1288 CB CYS 85 46.810 44.310 74.980 0.00 0.00 C

ATOM 1289 HB1 CYS 85 46.440 45.070 75.660 0.00 0.00 H

ATOM 1290 HB2 CYS 85 47.860 44.150 75.230 0.00 0.00 H

ATOM 1291 SG CYS 85 45.910 42.710 75.320 0.00 0.00 S

ATOM 1292 C CYS 85 45.460 44.850 72.800 0.00 0.00 C

ATOM 1293 O CYS 85 45.100 43.870 72.090 0.00 0.00 O

ATOM 1294 N ASP 86 44.710 45.920 73.050 0.00 0.00 N

ATOM 1295 H ASP 86 44.880 46.450 73.890 0.00 0.00 H

ATOM 1296 CA ASP 86 43.400 46.150 72.440 0.00 0.00 C

ATOM 1297 HA ASP 86 42.850 45.220 72.600 0.00 0.00 H

ATOM 1298 CB ASP 86 42.740 47.380 73.090 0.00 0.00 C

ATOM 1299 HB1 ASP 86 41.890 47.640 72.470 0.00 0.00 H

ATOM 1300 HB2 ASP 86 43.380 48.260 73.040 0.00 0.00 H

ATOM 1301 CG ASP 86 42.130 47.140 74.560 0.00 0.00 C

ATOM 1302 OD1 ASP 86 41.450 48.080 74.970 0.00 0.00 O

ATOM 1303 OD2 ASP 86 42.270 46.060 75.170 0.00 0.00 O1-

ATOM 1304 C ASP 86 43.500 46.360 70.930 0.00 0.00 C

ATOM 1305 O ASP 86 42.740 45.680 70.160 0.00 0.00 O

ATOM 1306 N LEU 87 44.480 47.120 70.520 0.00 0.00 N

ATOM 1307 H LEU 87 45.170 47.470 71.170 0.00 0.00 H

ATOM 1308 CA LEU 87 44.770 47.400 69.080 0.00 0.00 C

ATOM 1309 HA LEU 87 43.850 47.670 68.570 0.00 0.00 H

ATOM 1310 CB LEU 87 45.830 48.470 68.990 0.00 0.00 C

ATOM 1311 HB1 LEU 87 46.680 48.040 69.510 0.00 0.00 H

ATOM 1312 HB2 LEU 87 45.390 49.320 69.510 0.00 0.00 H

ATOM 1313 CG LEU 87 46.420 49.000 67.650 0.00 0.00 C

ATOM 1314 HG LEU 87 46.660 48.100 67.100 0.00 0.00 H

ATOM 1315 CD1 LEU 87 45.270 49.670 66.870 0.00 0.00 C

ATOM 1316 HD11 LEU 87 45.660 49.950 65.890 0.00 0.00 H

ATOM 1317 HD12 LEU 87 44.310 49.150 66.840 0.00 0.00 H

ATOM 1318 HD13 LEU 87 45.050 50.630 67.340 0.00 0.00 H

ATOM 1319 CD2 LEU 87 47.680 49.840 67.870 0.00 0.00 C

ATOM 1320 HD21 LEU 87 48.410 49.240 68.420 0.00 0.00 H

ATOM 1321 HD22 LEU 87 48.120 50.140 66.920 0.00 0.00 H

ATOM 1322 HD23 LEU 87 47.410 50.750 68.410 0.00 0.00 H

ATOM 1323 C LEU 87 45.240 46.100 68.330 0.00 0.00 C

ATOM 1324 O LEU 87 44.830 45.940 67.240 0.00 0.00 O

ATOM 1325 N PHE 88 46.060 45.300 68.960 0.00 0.00 N

ATOM 1326 H PHE 88 46.320 45.500 69.920 0.00 0.00 H

ATOM 1327 CA PHE 88 46.560 44.010 68.450 0.00 0.00 C

ATOM 1328 HA PHE 88 47.100 44.110 67.510 0.00 0.00 H

ATOM 1329 CB PHE 88 47.600 43.520 69.470 0.00 0.00 C

ATOM 1330 HB1 PHE 88 47.100 43.580 70.430 0.00 0.00 H

ATOM 1331 HB2 PHE 88 48.380 44.260 69.630 0.00 0.00 H

ATOM 1332 CG PHE 88 48.200 42.200 69.300 0.00 0.00 C

ATOM 1333 CD1 PHE 88 48.180 41.300 70.360 0.00 0.00 C

ATOM 1334 HD1 PHE 88 47.710 41.600 71.290 0.00 0.00 H

ATOM 1335 CE1 PHE 88 48.750 40.030 70.240 0.00 0.00 C

ATOM 1336 HE1 PHE 88 48.680 39.360 71.090 0.00 0.00 H

ATOM 1337 CZ PHE 88 49.410 39.620 69.060 0.00 0.00 C

ATOM 1338 HZ PHE 88 49.710 38.600 68.860 0.00 0.00 H

ATOM 1339 CE2 PHE 88 49.450 40.600 67.980 0.00 0.00 C

ATOM 1340 HE2 PHE 88 49.840 40.320 67.010 0.00 0.00 H

ATOM 1341 CD2 PHE 88 48.780 41.830 68.080 0.00 0.00 C

ATOM 1342 HD2 PHE 88 48.750 42.470 67.210 0.00 0.00 H

ATOM 1343 C PHE 88 45.310 43.030 68.410 0.00 0.00 C

ATOM 1344 O PHE 88 45.140 42.320 67.410 0.00 0.00 O

ATOM 1345 N ILE 89 44.380 43.040 69.420 0.00 0.00 N

ATOM 1346 H ILE 89 44.530 43.640 70.220 0.00 0.00 H

ATOM 1347 CA ILE 89 43.130 42.290 69.260 0.00 0.00 C

ATOM 1348 HA ILE 89 43.410 41.310 68.880 0.00 0.00 H

ATOM 1349 CB ILE 89 42.340 42.170 70.640 0.00 0.00 C

ATOM 1350 HB ILE 89 42.480 43.120 71.170 0.00 0.00 H

ATOM 1351 CG2 ILE 89 40.790 41.790 70.610 0.00 0.00 C

ATOM 1352 HG21 ILE 89 40.310 41.820 71.590 0.00 0.00 H

ATOM 1353 HG22 ILE 89 40.180 42.440 69.990 0.00 0.00 H

ATOM 1354 HG23 ILE 89 40.650 40.820 70.130 0.00 0.00 H

ATOM 1355 CG1 ILE 89 43.080 41.090 71.450 0.00 0.00 C

ATOM 1356 HG11 ILE 89 44.150 41.110 71.230 0.00 0.00 H

ATOM 1357 HG12 ILE 89 42.780 40.160 70.960 0.00 0.00 H

ATOM 1358 CD ILE 89 42.890 41.120 72.990 0.00 0.00 C

ATOM 1359 HD1 ILE 89 43.390 42.020 73.340 0.00 0.00 H

ATOM 1360 HD2 ILE 89 41.860 41.240 73.320 0.00 0.00 H

ATOM 1361 HD3 ILE 89 43.320 40.260 73.500 0.00 0.00 H

ATOM 1362 C ILE 89 42.310 42.830 68.080 0.00 0.00 C

ATOM 1363 O ILE 89 41.840 42.070 67.280 0.00 0.00 O

ATOM 1364 N ALA 90 42.190 44.160 67.970 0.00 0.00 N

ATOM 1365 H ALA 90 42.620 44.760 68.660 0.00 0.00 H

ATOM 1366 CA ALA 90 41.310 44.810 66.990 0.00 0.00 C

ATOM 1367 HA ALA 90 40.310 44.390 67.070 0.00 0.00 H

ATOM 1368 CB ALA 90 41.340 46.330 67.220 0.00 0.00 C

ATOM 1369 HB1 ALA 90 42.330 46.710 66.950 0.00 0.00 H

ATOM 1370 HB2 ALA 90 40.620 46.780 66.550 0.00 0.00 H

ATOM 1371 HB3 ALA 90 41.070 46.550 68.260 0.00 0.00 H

ATOM 1372 C ALA 90 41.760 44.460 65.580 0.00 0.00 C

ATOM 1373 O ALA 90 40.930 44.060 64.800 0.00 0.00 O

ATOM 1374 N LEU 91 43.060 44.620 65.220 0.00 0.00 N

ATOM 1375 H LEU 91 43.780 44.900 65.870 0.00 0.00 H

ATOM 1376 CA LEU 91 43.580 44.280 63.890 0.00 0.00 C

ATOM 1377 HA LEU 91 43.090 44.930 63.160 0.00 0.00 H

ATOM 1378 CB LEU 91 45.010 44.820 63.830 0.00 0.00 C

ATOM 1379 HB1 LEU 91 45.510 44.230 64.600 0.00 0.00 H

ATOM 1380 HB2 LEU 91 45.130 45.870 64.120 0.00 0.00 H

ATOM 1381 CG LEU 91 45.710 44.680 62.460 0.00 0.00 C

ATOM 1382 HG LEU 91 45.510 43.680 62.070 0.00 0.00 H

ATOM 1383 CD1 LEU 91 45.170 45.680 61.470 0.00 0.00 C

ATOM 1384 HD11 LEU 91 45.850 46.530 61.380 0.00 0.00 H

ATOM 1385 HD12 LEU 91 44.910 45.130 60.570 0.00 0.00 H

ATOM 1386 HD13 LEU 91 44.200 46.090 61.760 0.00 0.00 H

ATOM 1387 CD2 LEU 91 47.230 44.990 62.690 0.00 0.00 C

ATOM 1388 HD21 LEU 91 47.790 44.260 63.280 0.00 0.00 H

ATOM 1389 HD22 LEU 91 47.760 44.790 61.760 0.00 0.00 H

ATOM 1390 HD23 LEU 91 47.400 46.010 63.020 0.00 0.00 H

ATOM 1391 C LEU 91 43.430 42.770 63.500 0.00 0.00 C

ATOM 1392 O LEU 91 42.940 42.440 62.390 0.00 0.00 O

ATOM 1393 N ASP 92 43.930 41.900 64.360 0.00 0.00 N

ATOM 1394 H ASP 92 44.280 42.350 65.190 0.00 0.00 H

ATOM 1395 CA ASP 92 43.850 40.450 64.190 0.00 0.00 C

ATOM 1396 HA ASP 92 44.460 40.160 63.330 0.00 0.00 H

ATOM 1397 CB ASP 92 44.570 39.830 65.400 0.00 0.00 C

ATOM 1398 HB1 ASP 92 44.110 40.250 66.290 0.00 0.00 H

ATOM 1399 HB2 ASP 92 45.570 40.240 65.470 0.00 0.00 H

ATOM 1400 CG ASP 92 44.650 38.310 65.410 0.00 0.00 C

ATOM 1401 OD1 ASP 92 44.250 37.690 66.420 0.00 0.00 O

ATOM 1402 OD2 ASP 92 45.040 37.790 64.340 0.00 0.00 O1-

ATOM 1403 C ASP 92 42.380 39.920 64.030 0.00 0.00 C

ATOM 1404 O ASP 92 42.060 39.160 63.060 0.00 0.00 O

ATOM 1405 N VAL 93 41.480 40.360 64.920 0.00 0.00 N

ATOM 1406 H VAL 93 41.770 40.960 65.680 0.00 0.00 H

ATOM 1407 CA VAL 93 40.080 40.070 64.760 0.00 0.00 C

ATOM 1408 HA VAL 93 39.980 39.000 64.920 0.00 0.00 H

ATOM 1409 CB VAL 93 39.250 40.660 65.960 0.00 0.00 C

ATOM 1410 HB VAL 93 39.570 41.700 66.020 0.00 0.00 H

ATOM 1411 CG1 VAL 93 37.760 40.760 65.700 0.00 0.00 C

ATOM 1412 HG11 VAL 93 37.260 41.040 66.620 0.00 0.00 H

ATOM 1413 HG12 VAL 93 37.600 41.460 64.880 0.00 0.00 H

ATOM 1414 HG13 VAL 93 37.440 39.780 65.340 0.00 0.00 H

ATOM 1415 CG2 VAL 93 39.530 39.930 67.270 0.00 0.00 C

ATOM 1416 HG21 VAL 93 39.180 40.520 68.110 0.00 0.00 H

ATOM 1417 HG22 VAL 93 39.100 38.930 67.110 0.00 0.00 H

ATOM 1418 HG23 VAL 93 40.600 39.750 67.380 0.00 0.00 H

ATOM 1419 C VAL 93 39.470 40.540 63.400 0.00 0.00 C

ATOM 1420 O VAL 93 38.770 39.770 62.770 0.00 0.00 O

ATOM 1421 N LEU 94 39.750 41.790 63.000 0.00 0.00 N

ATOM 1422 H LEU 94 40.230 42.340 63.700 0.00 0.00 H

ATOM 1423 CA LEU 94 39.300 42.540 61.820 0.00 0.00 C

ATOM 1424 HA LEU 94 38.210 42.440 61.740 0.00 0.00 H

ATOM 1425 CB LEU 94 39.610 44.010 61.990 0.00 0.00 C

ATOM 1426 HB1 LEU 94 40.690 44.140 62.020 0.00 0.00 H

ATOM 1427 HB2 LEU 94 39.250 44.410 62.930 0.00 0.00 H

ATOM 1428 CG LEU 94 39.090 44.870 60.790 0.00 0.00 C

ATOM 1429 HG LEU 94 39.370 44.610 59.780 0.00 0.00 H

ATOM 1430 CD1 LEU 94 37.550 45.080 60.730 0.00 0.00 C

ATOM 1431 HD11 LEU 94 37.100 44.240 60.190 0.00 0.00 H

ATOM 1432 HD12 LEU 94 37.100 45.040 61.720 0.00 0.00 H

ATOM 1433 HD13 LEU 94 37.320 45.960 60.130 0.00 0.00 H

ATOM 1434 CD2 LEU 94 39.740 46.200 61.170 0.00 0.00 C

ATOM 1435 HD21 LEU 94 40.810 46.120 60.990 0.00 0.00 H

ATOM 1436 HD22 LEU 94 39.380 46.970 60.480 0.00 0.00 H

ATOM 1437 HD23 LEU 94 39.640 46.480 62.220 0.00 0.00 H

ATOM 1438 C LEU 94 39.870 41.860 60.550 0.00 0.00 C

ATOM 1439 O LEU 94 39.160 41.610 59.600 0.00 0.00 O

ATOM 1440 N CYS 95 41.180 41.610 60.440 0.00 0.00 N

ATOM 1441 H CYS 95 41.730 41.720 61.280 0.00 0.00 H

ATOM 1442 CA CYS 95 41.830 41.220 59.130 0.00 0.00 C

ATOM 1443 HA CYS 95 41.650 41.970 58.360 0.00 0.00 H

ATOM 1444 CB CYS 95 43.350 40.900 59.410 0.00 0.00 C

ATOM 1445 HB1 CYS 95 43.780 40.190 58.700 0.00 0.00 H

ATOM 1446 HB2 CYS 95 43.440 40.500 60.420 0.00 0.00 H

ATOM 1447 SG CYS 95 44.270 42.520 59.350 0.00 0.00 S

ATOM 1448 HG CYS 95 43.470 43.120 60.230 0.00 0.00 H

ATOM 1449 C CYS 95 41.190 39.940 58.600 0.00 0.00 C

ATOM 1450 O CYS 95 40.790 39.890 57.460 0.00 0.00 O

ATOM 1451 N CYS 96 41.140 38.870 59.440 0.00 0.00 N

ATOM 1452 H CYS 96 41.470 38.880 60.390 0.00 0.00 H

ATOM 1453 CA CYS 96 40.750 37.490 59.050 0.00 0.00 C

ATOM 1454 HA CYS 96 41.470 37.100 58.330 0.00 0.00 H

ATOM 1455 CB CYS 96 40.940 36.690 60.360 0.00 0.00 C

ATOM 1456 HB1 CYS 96 40.390 37.090 61.210 0.00 0.00 H

ATOM 1457 HB2 CYS 96 41.940 36.760 60.790 0.00 0.00 H

ATOM 1458 SG CYS 96 40.610 34.930 60.040 0.00 0.00 S

ATOM 1459 HG CYS 96 41.640 34.830 59.200 0.00 0.00 H

ATOM 1460 C CYS 96 39.340 37.310 58.460 0.00 0.00 C

ATOM 1461 O CYS 96 39.220 36.620 57.420 0.00 0.00 O

ATOM 1462 N THR 97 38.300 37.840 59.050 0.00 0.00 N

ATOM 1463 H THR 97 38.370 38.350 59.930 0.00 0.00 H

ATOM 1464 CA THR 97 36.890 37.870 58.590 0.00 0.00 C

ATOM 1465 HA THR 97 36.480 36.870 58.460 0.00 0.00 H

ATOM 1466 CB THR 97 36.110 38.460 59.720 0.00 0.00 C

ATOM 1467 HB THR 97 36.340 39.520 59.850 0.00 0.00 H

ATOM 1468 CG2 THR 97 34.610 38.350 59.380 0.00 0.00 C

ATOM 1469 HG21 THR 97 34.360 37.330 59.070 0.00 0.00 H

ATOM 1470 HG22 THR 97 34.120 38.470 60.350 0.00 0.00 H

ATOM 1471 HG23 THR 97 34.360 39.070 58.610 0.00 0.00 H

ATOM 1472 OG1 THR 97 36.320 37.630 60.840 0.00 0.00 O

ATOM 1473 HG1 THR 97 36.080 36.760 60.510 0.00 0.00 H

ATOM 1474 C THR 97 36.750 38.750 57.340 0.00 0.00 C

ATOM 1475 O THR 97 36.200 38.330 56.340 0.00 0.00 O

ATOM 1476 N SER 98 37.290 39.960 57.390 0.00 0.00 N

ATOM 1477 H SER 98 37.780 40.320 58.190 0.00 0.00 H

ATOM 1478 CA SER 98 37.410 40.800 56.170 0.00 0.00 C

ATOM 1479 HA SER 98 36.400 41.070 55.850 0.00 0.00 H

ATOM 1480 CB SER 98 38.100 42.140 56.520 0.00 0.00 C

ATOM 1481 HB1 SER 98 37.930 42.780 55.660 0.00 0.00 H

ATOM 1482 HB2 SER 98 39.140 41.890 56.740 0.00 0.00 H

ATOM 1483 OG SER 98 37.610 42.740 57.690 0.00 0.00 O

ATOM 1484 HG SER 98 38.240 42.480 58.360 0.00 0.00 H

ATOM 1485 C SER 98 38.110 40.090 54.980 0.00 0.00 C

ATOM 1486 O SER 98 37.710 40.300 53.840 0.00 0.00 O

ATOM 1487 N SER 99 39.050 39.130 55.170 0.00 0.00 N

ATOM 1488 H SER 99 39.460 39.050 56.090 0.00 0.00 H

ATOM 1489 CA SER 99 39.700 38.330 54.110 0.00 0.00 C

ATOM 1490 HA SER 99 39.970 39.000 53.280 0.00 0.00 H

ATOM 1491 CB SER 99 40.970 37.810 54.680 0.00 0.00 C

ATOM 1492 HB1 SER 99 41.480 37.110 54.010 0.00 0.00 H

ATOM 1493 HB2 SER 99 40.690 37.190 55.540 0.00 0.00 H

ATOM 1494 OG SER 99 41.910 38.840 54.960 0.00 0.00 O

ATOM 1495 HG SER 99 42.680 38.290 55.160 0.00 0.00 H

ATOM 1496 C SER 99 38.950 37.190 53.530 0.00 0.00 C

ATOM 1497 O SER 99 39.060 36.920 52.400 0.00 0.00 O

ATOM 1498 N ILE 100 38.150 36.420 54.300 0.00 0.00 N

ATOM 1499 H ILE 100 38.010 36.710 55.260 0.00 0.00 H

ATOM 1500 CA ILE 100 37.170 35.430 53.820 0.00 0.00 C

ATOM 1501 HA ILE 100 37.520 34.860 52.960 0.00 0.00 H

ATOM 1502 CB ILE 100 36.830 34.400 54.940 0.00 0.00 C

ATOM 1503 HB ILE 100 37.780 34.010 55.300 0.00 0.00 H

ATOM 1504 CG2 ILE 100 36.230 35.000 56.170 0.00 0.00 C

ATOM 1505 HG21 ILE 100 35.440 35.700 55.920 0.00 0.00 H

ATOM 1506 HG22 ILE 100 35.860 34.350 56.960 0.00 0.00 H

ATOM 1507 HG23 ILE 100 36.890 35.770 56.580 0.00 0.00 H

ATOM 1508 CG1 ILE 100 35.950 33.240 54.410 0.00 0.00 C

ATOM 1509 HG11 ILE 100 36.230 32.980 53.390 0.00 0.00 H

ATOM 1510 HG12 ILE 100 34.920 33.570 54.370 0.00 0.00 H

ATOM 1511 CD ILE 100 36.040 31.930 55.220 0.00 0.00 C

ATOM 1512 HD1 ILE 100 37.010 31.420 55.180 0.00 0.00 H

ATOM 1513 HD2 ILE 100 35.780 32.090 56.270 0.00 0.00 H

ATOM 1514 HD3 ILE 100 35.300 31.220 54.840 0.00 0.00 H

ATOM 1515 C ILE 100 35.920 36.130 53.210 0.00 0.00 C

ATOM 1516 O ILE 100 35.340 35.590 52.290 0.00 0.00 O

ATOM 1517 N LEU 101 35.610 37.350 53.600 0.00 0.00 N

ATOM 1518 H LEU 101 36.020 37.710 54.450 0.00 0.00 H

ATOM 1519 CA LEU 101 34.610 38.230 52.930 0.00 0.00 C

ATOM 1520 HA LEU 101 33.810 37.550 52.650 0.00 0.00 H

ATOM 1521 CB LEU 101 34.230 39.330 53.890 0.00 0.00 C

ATOM 1522 HB1 LEU 101 33.700 40.120 53.350 0.00 0.00 H

ATOM 1523 HB2 LEU 101 35.130 39.730 54.340 0.00 0.00 H

ATOM 1524 CG LEU 101 33.230 38.910 54.990 0.00 0.00 C

ATOM 1525 HG LEU 101 33.670 38.150 55.630 0.00 0.00 H

ATOM 1526 CD1 LEU 101 32.970 40.230 55.750 0.00 0.00 C

ATOM 1527 HD11 LEU 101 32.410 40.020 56.670 0.00 0.00 H

ATOM 1528 HD12 LEU 101 33.890 40.740 56.030 0.00 0.00 H

ATOM 1529 HD13 LEU 101 32.340 40.870 55.140 0.00 0.00 H

ATOM 1530 CD2 LEU 101 31.900 38.360 54.370 0.00 0.00 C

ATOM 1531 HD21 LEU 101 31.970 37.350 53.970 0.00 0.00 H

ATOM 1532 HD22 LEU 101 31.160 38.290 55.170 0.00 0.00 H

ATOM 1533 HD23 LEU 101 31.590 39.070 53.600 0.00 0.00 H

ATOM 1534 C LEU 101 35.170 38.630 51.640 0.00 0.00 C

ATOM 1535 O LEU 101 34.440 38.590 50.660 0.00 0.00 O

ATOM 1536 N HIS 102 36.420 39.040 51.510 0.00 0.00 N

ATOM 1537 H HIS 102 37.000 39.250 52.300 0.00 0.00 H

ATOM 1538 CA HIS 102 37.040 39.210 50.170 0.00 0.00 C

ATOM 1539 HA HIS 102 36.400 39.910 49.640 0.00 0.00 H

ATOM 1540 CB HIS 102 38.470 39.800 50.230 0.00 0.00 C

ATOM 1541 HB1 HIS 102 39.020 39.470 49.360 0.00 0.00 H

ATOM 1542 HB2 HIS 102 38.990 39.420 51.110 0.00 0.00 H

ATOM 1543 CG HIS 102 38.390 41.290 50.310 0.00 0.00 C

ATOM 1544 ND1 HIS 102 38.490 41.940 51.550 0.00 0.00 N

ATOM 1545 HD1 HIS 102 38.550 41.450 52.430 0.00 0.00 H

ATOM 1546 CE1 HIS 102 38.420 43.270 51.290 0.00 0.00 C

ATOM 1547 HE1 HIS 102 38.520 44.100 51.970 0.00 0.00 H

ATOM 1548 NE2 HIS 102 38.490 43.490 49.960 0.00 0.00 N

ATOM 1549 CD2 HIS 102 38.390 42.250 49.350 0.00 0.00 C

ATOM 1550 HD2 HIS 102 38.300 42.070 48.290 0.00 0.00 H

ATOM 1551 C HIS 102 37.050 37.930 49.340 0.00 0.00 C

ATOM 1552 O HIS 102 36.880 37.980 48.170 0.00 0.00 O

ATOM 1553 N LEU 103 37.180 36.740 49.970 0.00 0.00 N

ATOM 1554 H LEU 103 37.030 36.710 50.970 0.00 0.00 H

ATOM 1555 CA LEU 103 37.140 35.450 49.190 0.00 0.00 C

ATOM 1556 HA LEU 103 37.590 35.550 48.210 0.00 0.00 H

ATOM 1557 CB LEU 103 37.800 34.430 50.170 0.00 0.00 C

ATOM 1558 HB1 LEU 103 37.050 34.010 50.840 0.00 0.00 H

ATOM 1559 HB2 LEU 103 38.630 34.920 50.670 0.00 0.00 H

ATOM 1560 CG LEU 103 38.500 33.260 49.480 0.00 0.00 C

ATOM 1561 HG LEU 103 37.810 32.890 48.720 0.00 0.00 H

ATOM 1562 CD1 LEU 103 39.830 33.740 48.720 0.00 0.00 C

ATOM 1563 HD11 LEU 103 40.450 34.340 49.390 0.00 0.00 H

ATOM 1564 HD12 LEU 103 40.300 32.870 48.250 0.00 0.00 H

ATOM 1565 HD13 LEU 103 39.630 34.410 47.890 0.00 0.00 H

ATOM 1566 CD2 LEU 103 38.970 32.130 50.500 0.00 0.00 C

ATOM 1567 HD21 LEU 103 38.160 32.060 51.230 0.00 0.00 H

ATOM 1568 HD22 LEU 103 39.100 31.140 50.070 0.00 0.00 H

ATOM 1569 HD23 LEU 103 39.870 32.530 50.980 0.00 0.00 H

ATOM 1570 C LEU 103 35.670 35.120 48.790 0.00 0.00 C

ATOM 1571 O LEU 103 35.410 34.700 47.670 0.00 0.00 O

ATOM 1572 N CYS 104 34.620 35.240 49.650 0.00 0.00 N

ATOM 1573 H CYS 104 34.780 35.300 50.650 0.00 0.00 H

ATOM 1574 CA CYS 104 33.210 35.260 49.330 0.00 0.00 C

ATOM 1575 HA CYS 104 32.940 34.260 49.000 0.00 0.00 H

ATOM 1576 CB CYS 104 32.330 35.510 50.550 0.00 0.00 C

ATOM 1577 HB1 CYS 104 32.460 36.530 50.910 0.00 0.00 H

ATOM 1578 HB2 CYS 104 32.680 34.810 51.310 0.00 0.00 H

ATOM 1579 SG CYS 104 30.630 35.020 50.120 0.00 0.00 S

ATOM 1580 HG CYS 104 30.680 35.500 48.880 0.00 0.00 H

ATOM 1581 C CYS 104 32.890 36.190 48.150 0.00 0.00 C

ATOM 1582 O CYS 104 32.390 35.740 47.080 0.00 0.00 O

ATOM 1583 N ALA 105 33.210 37.470 48.260 0.00 0.00 N

ATOM 1584 H ALA 105 33.800 37.750 49.030 0.00 0.00 H

ATOM 1585 CA ALA 105 33.200 38.410 47.120 0.00 0.00 C

ATOM 1586 HA ALA 105 32.180 38.470 46.750 0.00 0.00 H

ATOM 1587 CB ALA 105 33.640 39.740 47.550 0.00 0.00 C

ATOM 1588 HB1 ALA 105 34.420 39.680 48.310 0.00 0.00 H

ATOM 1589 HB2 ALA 105 33.980 40.330 46.700 0.00 0.00 H

ATOM 1590 HB3 ALA 105 32.790 40.130 48.110 0.00 0.00 H

ATOM 1591 C ALA 105 33.940 37.910 45.910 0.00 0.00 C

ATOM 1592 O ALA 105 33.590 38.380 44.820 0.00 0.00 O

ATOM 1593 N ILE 106 34.860 36.910 45.940 0.00 0.00 N

ATOM 1594 H ILE 106 35.300 36.730 46.830 0.00 0.00 H

ATOM 1595 CA ILE 106 35.470 36.350 44.690 0.00 0.00 C

ATOM 1596 HA ILE 106 35.590 37.090 43.900 0.00 0.00 H

ATOM 1597 CB ILE 106 36.910 35.820 44.920 0.00 0.00 C

ATOM 1598 HB ILE 106 36.950 35.220 45.820 0.00 0.00 H

ATOM 1599 CG2 ILE 106 37.540 34.890 43.800 0.00 0.00 C

ATOM 1600 HG21 ILE 106 36.790 34.200 43.390 0.00 0.00 H

ATOM 1601 HG22 ILE 106 38.050 35.430 43.010 0.00 0.00 H

ATOM 1602 HG23 ILE 106 38.360 34.310 44.230 0.00 0.00 H

ATOM 1603 CG1 ILE 106 37.830 37.090 45.050 0.00 0.00 C

ATOM 1604 HG11 ILE 106 37.300 37.880 45.580 0.00 0.00 H

ATOM 1605 HG12 ILE 106 38.010 37.480 44.040 0.00 0.00 H

ATOM 1606 CD ILE 106 39.120 36.950 45.840 0.00 0.00 C

ATOM 1607 HD1 ILE 106 39.770 36.140 45.500 0.00 0.00 H

ATOM 1608 HD2 ILE 106 39.770 37.820 45.830 0.00 0.00 H

ATOM 1609 HD3 ILE 106 38.740 36.650 46.820 0.00 0.00 H

ATOM 1610 C ILE 106 34.560 35.330 44.080 0.00 0.00 C

ATOM 1611 O ILE 106 34.470 35.300 42.880 0.00 0.00 O

ATOM 1612 N ALA 107 33.890 34.460 44.910 0.00 0.00 N

ATOM 1613 H ALA 107 34.020 34.520 45.910 0.00 0.00 H

ATOM 1614 CA ALA 107 33.020 33.380 44.430 0.00 0.00 C

ATOM 1615 HA ALA 107 33.470 32.980 43.520 0.00 0.00 H

ATOM 1616 CB ALA 107 32.930 32.290 45.580 0.00 0.00 C

ATOM 1617 HB1 ALA 107 31.910 31.920 45.660 0.00 0.00 H

ATOM 1618 HB2 ALA 107 33.540 31.410 45.360 0.00 0.00 H

ATOM 1619 HB3 ALA 107 33.180 32.720 46.550 0.00 0.00 H

ATOM 1620 C ALA 107 31.620 33.890 44.060 0.00 0.00 C

ATOM 1621 O ALA 107 31.020 33.290 43.140 0.00 0.00 O

ATOM 1622 N LEU 108 31.210 35.000 44.740 0.00 0.00 N

ATOM 1623 H LEU 108 31.940 35.330 45.360 0.00 0.00 H

ATOM 1624 CA LEU 108 30.080 35.850 44.350 0.00 0.00 C

ATOM 1625 HA LEU 108 29.220 35.240 44.110 0.00 0.00 H

ATOM 1626 CB LEU 108 29.660 36.700 45.660 0.00 0.00 C

ATOM 1627 HB1 LEU 108 30.340 37.530 45.870 0.00 0.00 H

ATOM 1628 HB2 LEU 108 29.620 36.100 46.570 0.00 0.00 H

ATOM 1629 CG LEU 108 28.290 37.460 45.510 0.00 0.00 C

ATOM 1630 HG LEU 108 28.280 38.070 44.610 0.00 0.00 H

ATOM 1631 CD1 LEU 108 27.060 36.520 45.550 0.00 0.00 C

ATOM 1632 HD11 LEU 108 27.160 35.810 44.730 0.00 0.00 H

ATOM 1633 HD12 LEU 108 26.870 36.010 46.500 0.00 0.00 H

ATOM 1634 HD13 LEU 108 26.170 37.140 45.410 0.00 0.00 H

ATOM 1635 CD2 LEU 108 28.050 38.480 46.650 0.00 0.00 C

ATOM 1636 HD21 LEU 108 28.110 37.970 47.610 0.00 0.00 H

ATOM 1637 HD22 LEU 108 28.800 39.270 46.580 0.00 0.00 H

ATOM 1638 HD23 LEU 108 27.170 39.080 46.400 0.00 0.00 H

ATOM 1639 C LEU 108 30.280 36.650 43.020 0.00 0.00 C

ATOM 1640 O LEU 108 29.320 36.660 42.210 0.00 0.00 O

ATOM 1641 N ASP 109 31.440 37.270 42.720 0.00 0.00 N

ATOM 1642 H ASP 109 32.150 37.440 43.420 0.00 0.00 H

ATOM 1643 CA ASP 109 31.730 37.820 41.420 0.00 0.00 C

ATOM 1644 HA ASP 109 30.930 38.490 41.140 0.00 0.00 H

ATOM 1645 CB ASP 109 33.040 38.610 41.600 0.00 0.00 C

ATOM 1646 HB1 ASP 109 33.870 38.020 41.990 0.00 0.00 H

ATOM 1647 HB2 ASP 109 32.880 39.430 42.290 0.00 0.00 H

ATOM 1648 CG ASP 109 33.580 39.030 40.320 0.00 0.00 C

ATOM 1649 OD1 ASP 109 34.710 38.550 39.970 0.00 0.00 O

ATOM 1650 OD2 ASP 109 33.030 39.910 39.680 0.00 0.00 O1-

ATOM 1651 C ASP 109 31.750 36.680 40.360 0.00 0.00 C

ATOM 1652 O ASP 109 31.280 36.900 39.250 0.00 0.00 O

ATOM 1653 N ARG 110 32.290 35.430 40.550 0.00 0.00 N

ATOM 1654 H ARG 110 32.640 35.250 41.480 0.00 0.00 H

ATOM 1655 CA ARG 110 32.290 34.210 39.620 0.00 0.00 C

ATOM 1656 HA ARG 110 32.770 34.490 38.680 0.00 0.00 H

ATOM 1657 CB ARG 110 33.290 33.170 40.220 0.00 0.00 C

ATOM 1658 HB1 ARG 110 32.930 32.810 41.190 0.00 0.00 H

ATOM 1659 HB2 ARG 110 34.230 33.690 40.430 0.00 0.00 H

ATOM 1660 CG ARG 110 33.600 31.930 39.260 0.00 0.00 C

ATOM 1661 HG1 ARG 110 32.650 31.470 39.030 0.00 0.00 H

ATOM 1662 HG2 ARG 110 34.100 31.190 39.880 0.00 0.00 H

ATOM 1663 CD ARG 110 34.210 32.240 37.920 0.00 0.00 C

ATOM 1664 HD1 ARG 110 35.160 32.740 38.110 0.00 0.00 H

ATOM 1665 HD2 ARG 110 33.510 32.940 37.470 0.00 0.00 H

ATOM 1666 NE ARG 110 34.420 31.090 37.120 0.00 0.00 N

ATOM 1667 HE ARG 110 34.560 30.200 37.580 0.00 0.00 H

ATOM 1668 CZ ARG 110 34.660 31.110 35.810 0.00 0.00 C

ATOM 1669 NH1 ARG 110 34.790 32.280 35.210 0.00 0.00 N1+

ATOM 1670 HH11 ARG 110 34.610 33.110 35.760 0.00 0.00 H

ATOM 1671 HH12 ARG 110 34.860 32.320 34.210 0.00 0.00 H

ATOM 1672 NH2 ARG 110 34.880 29.950 35.150 0.00 0.00 N

ATOM 1673 HH21 ARG 110 34.580 29.140 35.670 0.00 0.00 H

ATOM 1674 HH22 ARG 110 35.020 29.880 34.150 0.00 0.00 H

ATOM 1675 C ARG 110 30.890 33.740 39.350 0.00 0.00 C

ATOM 1676 O ARG 110 30.440 33.410 38.250 0.00 0.00 O

ATOM 1677 N TYR 111 30.090 33.670 40.410 0.00 0.00 N

ATOM 1678 H TYR 111 30.600 33.790 41.280 0.00 0.00 H

ATOM 1679 CA TYR 111 28.630 33.330 40.410 0.00 0.00 C

ATOM 1680 HA TYR 111 28.390 32.370 39.950 0.00 0.00 H

ATOM 1681 CB TYR 111 28.110 33.400 41.820 0.00 0.00 C

ATOM 1682 HB1 TYR 111 28.480 34.280 42.350 0.00 0.00 H

ATOM 1683 HB2 TYR 111 28.510 32.570 42.390 0.00 0.00 H

ATOM 1684 CG TYR 111 26.620 33.270 41.920 0.00 0.00 C

ATOM 1685 CD1 TYR 111 25.810 34.450 42.030 0.00 0.00 C

ATOM 1686 HD1 TYR 111 26.210 35.450 42.120 0.00 0.00 H

ATOM 1687 CE1 TYR 111 24.440 34.300 42.020 0.00 0.00 C

ATOM 1688 HE1 TYR 111 23.830 35.180 42.170 0.00 0.00 H

ATOM 1689 CZ TYR 111 23.750 33.060 41.930 0.00 0.00 C

ATOM 1690 OH TYR 111 22.430 32.940 42.150 0.00 0.00 O

ATOM 1691 HH TYR 111 22.000 33.790 42.300 0.00 0.00 H

ATOM 1692 CE2 TYR 111 24.590 31.960 41.750 0.00 0.00 C

ATOM 1693 HE2 TYR 111 24.150 30.980 41.750 0.00 0.00 H

ATOM 1694 CD2 TYR 111 26.020 32.030 41.730 0.00 0.00 C

ATOM 1695 HD2 TYR 111 26.570 31.120 41.570 0.00 0.00 H

ATOM 1696 C TYR 111 27.830 34.400 39.490 0.00 0.00 C

ATOM 1697 O TYR 111 27.220 33.920 38.560 0.00 0.00 O

ATOM 1698 N TRP 112 27.940 35.710 39.740 0.00 0.00 N

ATOM 1699 H TRP 112 28.430 36.090 40.530 0.00 0.00 H

ATOM 1700 CA TRP 112 27.100 36.650 38.970 0.00 0.00 C

ATOM 1701 HA TRP 112 26.040 36.400 39.040 0.00 0.00 H

ATOM 1702 CB TRP 112 27.200 38.030 39.630 0.00 0.00 C

ATOM 1703 HB1 TRP 112 27.060 38.800 38.870 0.00 0.00 H

ATOM 1704 HB2 TRP 112 28.190 38.310 39.990 0.00 0.00 H

ATOM 1705 CG TRP 112 26.290 38.420 40.750 0.00 0.00 C

ATOM 1706 CD1 TRP 112 25.100 37.840 40.990 0.00 0.00 C

ATOM 1707 HD1 TRP 112 24.760 36.960 40.460 0.00 0.00 H

ATOM 1708 NE1 TRP 112 24.560 38.410 42.160 0.00 0.00 N

ATOM 1709 HE1 TRP 112 23.650 38.190 42.540 0.00 0.00 H

ATOM 1710 CE2 TRP 112 25.360 39.340 42.760 0.00 0.00 C

ATOM 1711 CZ2 TRP 112 25.270 40.120 43.860 0.00 0.00 C

ATOM 1712 HZ2 TRP 112 24.450 40.000 44.560 0.00 0.00 H

ATOM 1713 CH2 TRP 112 26.270 41.080 44.100 0.00 0.00 C

ATOM 1714 HH2 TRP 112 26.120 41.750 44.940 0.00 0.00 H

ATOM 1715 CZ3 TRP 112 27.400 41.130 43.320 0.00 0.00 C

ATOM 1716 HZ3 TRP 112 28.060 41.920 43.630 0.00 0.00 H

ATOM 1717 CE3 TRP 112 27.430 40.400 42.140 0.00 0.00 C

ATOM 1718 HE3 TRP 112 28.270 40.490 41.460 0.00 0.00 H

ATOM 1719 CD2 TRP 112 26.430 39.420 41.780 0.00 0.00 C

ATOM 1720 C TRP 112 27.530 36.680 37.500 0.00 0.00 C

ATOM 1721 O TRP 112 26.680 36.870 36.610 0.00 0.00 O

ATOM 1722 N ALA 113 28.840 36.480 37.240 0.00 0.00 N

ATOM 1723 H ALA 113 29.420 36.570 38.060 0.00 0.00 H

ATOM 1724 CA ALA 113 29.520 36.420 35.950 0.00 0.00 C

ATOM 1725 HA ALA 113 29.090 37.190 35.320 0.00 0.00 H

ATOM 1726 CB ALA 113 31.000 36.800 36.110 0.00 0.00 C

ATOM 1727 HB1 ALA 113 31.350 37.000 35.100 0.00 0.00 H

ATOM 1728 HB2 ALA 113 31.030 37.720 36.700 0.00 0.00 H

ATOM 1729 HB3 ALA 113 31.510 35.930 36.520 0.00 0.00 H

ATOM 1730 C ALA 113 29.280 35.160 35.140 0.00 0.00 C

ATOM 1731 O ALA 113 29.190 35.230 33.900 0.00 0.00 O

ATOM 1732 N ILE 114 28.920 34.080 35.830 0.00 0.00 N

ATOM 1733 H ILE 114 29.060 34.000 36.820 0.00 0.00 H

ATOM 1734 CA ILE 114 28.470 32.860 35.130 0.00 0.00 C

ATOM 1735 HA ILE 114 28.900 32.740 34.140 0.00 0.00 H

ATOM 1736 CB ILE 114 28.770 31.510 35.860 0.00 0.00 C

ATOM 1737 HB ILE 114 28.550 31.780 36.890 0.00 0.00 H

ATOM 1738 CG2 ILE 114 27.880 30.330 35.390 0.00 0.00 C

ATOM 1739 HG21 ILE 114 26.850 30.560 35.630 0.00 0.00 H

ATOM 1740 HG22 ILE 114 28.040 30.220 34.320 0.00 0.00 H

ATOM 1741 HG23 ILE 114 28.040 29.390 35.910 0.00 0.00 H

ATOM 1742 CG1 ILE 114 30.260 31.140 35.720 0.00 0.00 C

ATOM 1743 HG11 ILE 114 30.800 32.060 35.920 0.00 0.00 H

ATOM 1744 HG12 ILE 114 30.510 30.870 34.690 0.00 0.00 H

ATOM 1745 CD ILE 114 30.770 30.070 36.710 0.00 0.00 C

ATOM 1746 HD1 ILE 114 31.770 29.870 36.320 0.00 0.00 H

ATOM 1747 HD2 ILE 114 30.770 30.460 37.730 0.00 0.00 H

ATOM 1748 HD3 ILE 114 30.060 29.250 36.700 0.00 0.00 H

ATOM 1749 C ILE 114 26.960 32.950 34.790 0.00 0.00 C

ATOM 1750 O ILE 114 26.530 32.560 33.730 0.00 0.00 O

ATOM 1751 N THR 115 26.200 33.480 35.750 0.00 0.00 N

ATOM 1752 H THR 115 26.740 33.700 36.570 0.00 0.00 H

ATOM 1753 CA THR 115 24.740 33.640 35.790 0.00 0.00 C

ATOM 1754 HA THR 115 24.370 32.630 35.600 0.00 0.00 H

ATOM 1755 CB THR 115 24.120 34.130 37.130 0.00 0.00 C

ATOM 1756 HB THR 115 24.460 35.130 37.400 0.00 0.00 H

ATOM 1757 CG2 THR 115 22.660 33.950 37.260 0.00 0.00 C

ATOM 1758 HG21 THR 115 22.360 32.980 36.870 0.00 0.00 H

ATOM 1759 HG22 THR 115 22.310 34.000 38.290 0.00 0.00 H

ATOM 1760 HG23 THR 115 22.130 34.750 36.750 0.00 0.00 H

ATOM 1761 OG1 THR 115 24.540 33.240 38.130 0.00 0.00 O

ATOM 1762 HG1 THR 115 25.470 33.370 38.330 0.00 0.00 H

ATOM 1763 C THR 115 24.210 34.570 34.690 0.00 0.00 C

ATOM 1764 O THR 115 23.200 34.200 34.060 0.00 0.00 O

ATOM 1765 N ASP 116 24.710 35.870 34.630 0.00 0.00 N

ATOM 1766 H ASP 116 25.450 36.100 35.280 0.00 0.00 H

ATOM 1767 CA ASP 116 24.040 36.960 33.910 0.00 0.00 C

ATOM 1768 HA ASP 116 23.000 36.690 33.750 0.00 0.00 H

ATOM 1769 CB ASP 116 23.870 38.240 34.740 0.00 0.00 C

ATOM 1770 HB1 ASP 116 24.830 38.760 34.800 0.00 0.00 H

ATOM 1771 HB2 ASP 116 23.890 38.010 35.800 0.00 0.00 H

ATOM 1772 CG ASP 116 22.710 39.170 34.320 0.00 0.00 C

ATOM 1773 OD1 ASP 116 21.810 39.490 35.160 0.00 0.00 O

ATOM 1774 OD2 ASP 116 22.710 39.590 33.160 0.00 0.00 O1-

ATOM 1775 C ASP 116 24.680 37.300 32.630 0.00 0.00 C

ATOM 1776 O ASP 116 25.870 37.630 32.680 0.00 0.00 O

ATOM 1777 N PRO 117 23.870 37.300 31.550 0.00 0.00 N

ATOM 1778 CD PRO 117 22.590 36.570 31.440 0.00 0.00 C

ATOM 1779 HD1 PRO 117 21.740 37.220 31.620 0.00 0.00 H

ATOM 1780 HD2 PRO 117 22.680 35.670 32.050 0.00 0.00 H

ATOM 1781 CG PRO 117 22.520 36.120 29.960 0.00 0.00 C

ATOM 1782 HG1 PRO 117 21.560 36.330 29.490 0.00 0.00 H

ATOM 1783 HG2 PRO 117 22.740 35.060 29.860 0.00 0.00 H

ATOM 1784 CB PRO 117 23.640 36.850 29.200 0.00 0.00 C

ATOM 1785 HB1 PRO 117 23.230 37.490 28.420 0.00 0.00 H

ATOM 1786 HB2 PRO 117 24.330 36.180 28.690 0.00 0.00 H

ATOM 1787 CA PRO 117 24.410 37.710 30.230 0.00 0.00 C

ATOM 1788 HA PRO 117 25.460 37.430 30.190 0.00 0.00 H

ATOM 1789 C PRO 117 24.410 39.180 29.830 0.00 0.00 C

ATOM 1790 O PRO 117 25.290 39.670 29.150 0.00 0.00 O

ATOM 1791 N ILE 118 23.470 39.980 30.300 0.00 0.00 N

ATOM 1792 H ILE 118 23.030 39.590 31.130 0.00 0.00 H

ATOM 1793 CA ILE 118 23.060 41.320 29.840 0.00 0.00 C

ATOM 1794 HA ILE 118 23.600 41.640 28.950 0.00 0.00 H

ATOM 1795 CB ILE 118 21.480 41.320 29.560 0.00 0.00 C

ATOM 1796 HB ILE 118 21.000 41.170 30.520 0.00 0.00 H

ATOM 1797 CG2 ILE 118 21.140 42.660 28.820 0.00 0.00 C

ATOM 1798 HG21 ILE 118 20.100 42.620 28.500 0.00 0.00 H

ATOM 1799 HG22 ILE 118 21.200 43.550 29.450 0.00 0.00 H

ATOM 1800 HG23 ILE 118 21.820 42.740 27.970 0.00 0.00 H

ATOM 1801 CG1 ILE 118 21.010 40.270 28.600 0.00 0.00 C

ATOM 1802 HG11 ILE 118 19.920 40.370 28.600 0.00 0.00 H

ATOM 1803 HG12 ILE 118 21.120 39.250 28.950 0.00 0.00 H

ATOM 1804 CD ILE 118 21.440 40.250 27.130 0.00 0.00 C

ATOM 1805 HD1 ILE 118 20.780 40.970 26.640 0.00 0.00 H

ATOM 1806 HD2 ILE 118 22.470 40.530 26.940 0.00 0.00 H

ATOM 1807 HD3 ILE 118 21.190 39.270 26.730 0.00 0.00 H

ATOM 1808 C ILE 118 23.430 42.370 30.850 0.00 0.00 C

ATOM 1809 O ILE 118 23.970 43.440 30.480 0.00 0.00 O

ATOM 1810 N ASP 119 23.150 42.270 32.150 0.00 0.00 N

ATOM 1811 H ASP 119 22.920 41.360 32.540 0.00 0.00 H

ATOM 1812 CA ASP 119 23.170 43.440 32.990 0.00 0.00 C

ATOM 1813 HA ASP 119 22.900 44.370 32.490 0.00 0.00 H

ATOM 1814 CB ASP 119 21.950 43.200 33.840 0.00 0.00 C

ATOM 1815 HB1 ASP 119 21.990 42.230 34.330 0.00 0.00 H

ATOM 1816 HB2 ASP 119 21.070 43.300 33.210 0.00 0.00 H

ATOM 1817 CG ASP 119 21.820 44.340 34.950 0.00 0.00 C

ATOM 1818 OD1 ASP 119 21.300 45.360 34.600 0.00 0.00 O

ATOM 1819 OD2 ASP 119 22.210 44.140 36.150 0.00 0.00 O1-

ATOM 1820 C ASP 119 24.470 43.560 33.870 0.00 0.00 C

ATOM 1821 O ASP 119 25.120 44.670 33.870 0.00 0.00 O

ATOM 1822 N TYR 120 25.040 42.450 34.420 0.00 0.00 N

ATOM 1823 H TYR 120 24.600 41.560 34.250 0.00 0.00 H

ATOM 1824 CA TYR 120 26.290 42.530 35.280 0.00 0.00 C

ATOM 1825 HA TYR 120 26.260 43.330 36.020 0.00 0.00 H

ATOM 1826 CB TYR 120 26.460 41.180 35.960 0.00 0.00 C

ATOM 1827 HB1 TYR 120 26.660 40.410 35.220 0.00 0.00 H

ATOM 1828 HB2 TYR 120 25.540 41.020 36.530 0.00 0.00 H

ATOM 1829 CG TYR 120 27.650 41.100 36.960 0.00 0.00 C

ATOM 1830 CD1 TYR 120 27.620 41.990 38.070 0.00 0.00 C

ATOM 1831 HD1 TYR 120 26.940 42.830 38.110 0.00 0.00 H

ATOM 1832 CE1 TYR 120 28.640 41.970 39.060 0.00 0.00 C

ATOM 1833 HE1 TYR 120 28.650 42.690 39.870 0.00 0.00 H

ATOM 1834 CZ TYR 120 29.670 41.060 38.940 0.00 0.00 C

ATOM 1835 OH TYR 120 30.660 41.190 39.860 0.00 0.00 O

ATOM 1836 HH TYR 120 31.480 40.750 39.640 0.00 0.00 H

ATOM 1837 CE2 TYR 120 29.690 40.130 37.860 0.00 0.00 C

ATOM 1838 HE2 TYR 120 30.440 39.360 37.880 0.00 0.00 H

ATOM 1839 CD2 TYR 120 28.770 40.210 36.790 0.00 0.00 C

ATOM 1840 HD2 TYR 120 29.000 39.600 35.930 0.00 0.00 H

ATOM 1841 C TYR 120 27.550 42.960 34.480 0.00 0.00 C

ATOM 1842 O TYR 120 28.440 43.630 35.030 0.00 0.00 O

ATOM 1843 N VAL 121 27.630 42.500 33.220 0.00 0.00 N

ATOM 1844 H VAL 121 26.900 41.910 32.850 0.00 0.00 H

ATOM 1845 CA VAL 121 28.750 42.930 32.330 0.00 0.00 C

ATOM 1846 HA VAL 121 29.650 42.600 32.850 0.00 0.00 H

ATOM 1847 CB VAL 121 28.750 42.070 30.990 0.00 0.00 C

ATOM 1848 HB VAL 121 28.600 41.050 31.320 0.00 0.00 H

ATOM 1849 CG1 VAL 121 27.630 42.480 29.970 0.00 0.00 C

ATOM 1850 HG11 VAL 121 28.010 43.440 29.640 0.00 0.00 H

ATOM 1851 HG12 VAL 121 27.480 41.800 29.140 0.00 0.00 H

ATOM 1852 HG13 VAL 121 26.670 42.470 30.500 0.00 0.00 H

ATOM 1853 CG2 VAL 121 30.140 42.140 30.390 0.00 0.00 C

ATOM 1854 HG21 VAL 121 30.840 42.000 31.220 0.00 0.00 H

ATOM 1855 HG22 VAL 121 30.270 41.400 29.600 0.00 0.00 H

ATOM 1856 HG23 VAL 121 30.300 43.100 29.910 0.00 0.00 H

ATOM 1857 C VAL 121 28.790 44.370 31.990 0.00 0.00 C

ATOM 1858 O VAL 121 29.900 44.820 31.840 0.00 0.00 O

ATOM 1859 N ASN 122 27.670 45.140 31.940 0.00 0.00 N

ATOM 1860 H ASN 122 26.840 44.660 32.270 0.00 0.00 H

ATOM 1861 CA ASN 122 27.780 46.610 31.790 0.00 0.00 C

ATOM 1862 HA ASN 122 28.410 46.700 30.900 0.00 0.00 H

ATOM 1863 CB ASN 122 26.360 47.170 31.440 0.00 0.00 C

ATOM 1864 HB1 ASN 122 25.610 47.090 32.230 0.00 0.00 H

ATOM 1865 HB2 ASN 122 26.010 46.690 30.530 0.00 0.00 H

ATOM 1866 CG ASN 122 26.310 48.650 31.110 0.00 0.00 C

ATOM 1867 OD1 ASN 122 26.020 49.540 31.910 0.00 0.00 O

ATOM 1868 ND2 ASN 122 26.820 49.070 29.960 0.00 0.00 N

ATOM 1869 HD21 ASN 122 27.210 48.410 29.300 0.00 0.00 H

ATOM 1870 HD22 ASN 122 26.900 50.060 29.750 0.00 0.00 H

ATOM 1871 C ASN 122 28.550 47.210 33.010 0.00 0.00 C

ATOM 1872 O ASN 122 29.410 48.090 32.890 0.00 0.00 O

ATOM 1873 N LYS 123 28.210 46.680 34.180 0.00 0.00 N

ATOM 1874 H LYS 123 27.540 45.930 34.120 0.00 0.00 H

ATOM 1875 CA LYS 123 28.480 47.220 35.520 0.00 0.00 C

ATOM 1876 HA LYS 123 28.530 48.300 35.450 0.00 0.00 H

ATOM 1877 CB LYS 123 27.350 46.760 36.470 0.00 0.00 C

ATOM 1878 HB1 LYS 123 27.690 47.150 37.430 0.00 0.00 H

ATOM 1879 HB2 LYS 123 27.270 45.670 36.400 0.00 0.00 H

ATOM 1880 CG LYS 123 26.030 47.350 36.070 0.00 0.00 C

ATOM 1881 HG1 LYS 123 25.710 47.200 35.040 0.00 0.00 H

ATOM 1882 HG2 LYS 123 26.020 48.430 36.220 0.00 0.00 H

ATOM 1883 CD LYS 123 24.870 46.820 36.900 0.00 0.00 C

ATOM 1884 HD1 LYS 123 24.960 46.940 37.980 0.00 0.00 H

ATOM 1885 HD2 LYS 123 24.840 45.730 36.790 0.00 0.00 H

ATOM 1886 CE LYS 123 23.520 47.370 36.410 0.00 0.00 C

ATOM 1887 HE1 LYS 123 23.380 47.400 35.320 0.00 0.00 H

ATOM 1888 HE2 LYS 123 23.510 48.430 36.670 0.00 0.00 H

ATOM 1889 NZ LYS 123 22.340 46.870 37.060 0.00 0.00 N1+

ATOM 1890 HZ1 LYS 123 22.530 46.710 38.040 0.00 0.00 H

ATOM 1891 HZ2 LYS 123 21.550 47.490 37.040 0.00 0.00 H

ATOM 1892 HZ3 LYS 123 22.070 45.990 36.630 0.00 0.00 H

ATOM 1893 C LYS 123 29.860 46.910 36.090 0.00 0.00 C

ATOM 1894 O LYS 123 30.390 47.730 36.870 0.00 0.00 O

ATOM 1895 N ARG 124 30.430 45.740 35.730 0.00 0.00 N

ATOM 1896 H ARG 124 30.010 45.140 35.030 0.00 0.00 H

ATOM 1897 CA ARG 124 31.560 45.100 36.490 0.00 0.00 C

ATOM 1898 HA ARG 124 31.280 45.140 37.540 0.00 0.00 H

ATOM 1899 CB ARG 124 31.580 43.640 36.130 0.00 0.00 C

ATOM 1900 HB1 ARG 124 31.600 43.450 35.060 0.00 0.00 H

ATOM 1901 HB2 ARG 124 30.680 43.210 36.570 0.00 0.00 H

ATOM 1902 CG ARG 124 32.620 42.720 36.790 0.00 0.00 C

ATOM 1903 HG1 ARG 124 32.520 42.690 37.880 0.00 0.00 H

ATOM 1904 HG2 ARG 124 33.640 42.990 36.550 0.00 0.00 H

ATOM 1905 CD ARG 124 32.520 41.330 36.120 0.00 0.00 C

ATOM 1906 HD1 ARG 124 32.900 41.400 35.100 0.00 0.00 H

ATOM 1907 HD2 ARG 124 31.440 41.180 36.150 0.00 0.00 H

ATOM 1908 NE ARG 124 33.310 40.290 36.850 0.00 0.00 N

ATOM 1909 HE ARG 124 33.050 40.120 37.810 0.00 0.00 H

ATOM 1910 CZ ARG 124 34.360 39.580 36.460 0.00 0.00 C

ATOM 1911 NH1 ARG 124 34.890 39.640 35.330 0.00 0.00 N1+

ATOM 1912 HH11 ARG 124 34.620 40.310 34.620 0.00 0.00 H

ATOM 1913 HH12 ARG 124 35.650 39.010 35.110 0.00 0.00 H

ATOM 1914 NH2 ARG 124 34.900 38.720 37.280 0.00 0.00 N

ATOM 1915 HH21 ARG 124 34.490 38.650 38.200 0.00 0.00 H

ATOM 1916 HH22 ARG 124 35.730 38.200 37.040 0.00 0.00 H

ATOM 1917 C ARG 124 32.890 45.820 36.110 0.00 0.00 C

ATOM 1918 O ARG 124 33.490 45.490 35.090 0.00 0.00 O

ATOM 1919 N THR 125 33.350 46.860 36.850 0.00 0.00 N

ATOM 1920 H THR 125 32.750 47.140 37.600 0.00 0.00 H

ATOM 1921 CA THR 125 34.480 47.710 36.550 0.00 0.00 C

ATOM 1922 HA THR 125 35.100 47.200 35.800 0.00 0.00 H

ATOM 1923 CB THR 125 33.890 48.970 35.870 0.00 0.00 C

ATOM 1924 HB THR 125 34.790 49.550 35.660 0.00 0.00 H

ATOM 1925 CG2 THR 125 33.080 48.710 34.630 0.00 0.00 C

ATOM 1926 HG21 THR 125 33.540 47.910 34.050 0.00 0.00 H

ATOM 1927 HG22 THR 125 32.080 48.340 34.850 0.00 0.00 H

ATOM 1928 HG23 THR 125 33.020 49.670 34.120 0.00 0.00 H

ATOM 1929 OG1 THR 125 33.200 49.640 36.880 0.00 0.00 O

ATOM 1930 HG1 THR 125 32.960 50.530 36.610 0.00 0.00 H

ATOM 1931 C THR 125 35.410 48.020 37.800 0.00 0.00 C

ATOM 1932 O THR 125 34.960 47.790 38.890 0.00 0.00 O

ATOM 1933 N PRO 126 36.690 48.380 37.660 0.00 0.00 N

ATOM 1934 CD PRO 126 37.350 48.480 36.430 0.00 0.00 C

ATOM 1935 HD1 PRO 126 37.200 49.480 36.020 0.00 0.00 H

ATOM 1936 HD2 PRO 126 37.230 47.650 35.740 0.00 0.00 H

ATOM 1937 CG PRO 126 38.830 48.340 36.750 0.00 0.00 C

ATOM 1938 HG1 PRO 126 39.550 48.770 36.050 0.00 0.00 H

ATOM 1939 HG2 PRO 126 39.060 47.280 36.850 0.00 0.00 H

ATOM 1940 CB PRO 126 38.940 48.940 38.170 0.00 0.00 C

ATOM 1941 HB1 PRO 126 39.080 50.020 38.110 0.00 0.00 H

ATOM 1942 HB2 PRO 126 39.820 48.590 38.700 0.00 0.00 H

ATOM 1943 CA PRO 126 37.630 48.570 38.750 0.00 0.00 C

ATOM 1944 HA PRO 126 37.790 47.590 39.200 0.00 0.00 H

ATOM 1945 C PRO 126 37.110 49.590 39.780 0.00 0.00 C

ATOM 1946 O PRO 126 37.020 49.240 40.940 0.00 0.00 O

ATOM 1947 N ARG 127 36.690 50.730 39.280 0.00 0.00 N

ATOM 1948 H ARG 127 36.900 50.930 38.310 0.00 0.00 H

ATOM 1949 CA ARG 127 35.810 51.610 40.020 0.00 0.00 C

ATOM 1950 HA ARG 127 36.400 52.140 40.770 0.00 0.00 H

ATOM 1951 CB ARG 127 35.210 52.660 39.120 0.00 0.00 C

ATOM 1952 HB1 ARG 127 34.730 52.150 38.280 0.00 0.00 H

ATOM 1953 HB2 ARG 127 36.070 53.220 38.750 0.00 0.00 H

ATOM 1954 CG ARG 127 34.290 53.650 39.750 0.00 0.00 C

ATOM 1955 HG1 ARG 127 34.920 54.210 40.430 0.00 0.00 H

ATOM 1956 HG2 ARG 127 33.370 53.220 40.140 0.00 0.00 H

ATOM 1957 CD ARG 127 33.720 54.450 38.600 0.00 0.00 C

ATOM 1958 HD1 ARG 127 32.780 54.960 38.830 0.00 0.00 H

ATOM 1959 HD2 ARG 127 33.400 53.760 37.810 0.00 0.00 H

ATOM 1960 NE ARG 127 34.640 55.400 37.910 0.00 0.00 N

ATOM 1961 HE ARG 127 35.140 55.050 37.110 0.00 0.00 H

ATOM 1962 CZ ARG 127 34.780 56.660 38.260 0.00 0.00 C

ATOM 1963 NH1 ARG 127 34.200 57.180 39.300 0.00 0.00 N1+

ATOM 1964 HH11 ARG 127 33.610 56.650 39.920 0.00 0.00 H

ATOM 1965 HH12 ARG 127 34.560 58.050 39.650 0.00 0.00 H

ATOM 1966 NH2 ARG 127 35.580 57.480 37.550 0.00 0.00 N

ATOM 1967 HH21 ARG 127 36.150 57.110 36.800 0.00 0.00 H

ATOM 1968 HH22 ARG 127 35.410 58.470 37.680 0.00 0.00 H

ATOM 1969 C ARG 127 34.710 50.960 40.820 0.00 0.00 C

ATOM 1970 O ARG 127 34.500 51.310 41.970 0.00 0.00 O

ATOM 1971 N ARG 128 33.980 49.920 40.320 0.00 0.00 N

ATOM 1972 H ARG 128 34.170 49.620 39.370 0.00 0.00 H

ATOM 1973 CA ARG 128 32.970 49.150 41.040 0.00 0.00 C

ATOM 1974 HA ARG 128 32.360 49.880 41.560 0.00 0.00 H

ATOM 1975 CB ARG 128 32.100 48.420 39.970 0.00 0.00 C

ATOM 1976 HB1 ARG 128 32.570 47.490 39.630 0.00 0.00 H

ATOM 1977 HB2 ARG 128 31.900 49.130 39.170 0.00 0.00 H

ATOM 1978 CG ARG 128 30.750 48.110 40.670 0.00 0.00 C

ATOM 1979 HG1 ARG 128 30.190 49.020 40.880 0.00 0.00 H

ATOM 1980 HG2 ARG 128 30.960 47.520 41.560 0.00 0.00 H

ATOM 1981 CD ARG 128 29.810 47.140 39.890 0.00 0.00 C

ATOM 1982 HD1 ARG 128 30.460 46.350 39.540 0.00 0.00 H

ATOM 1983 HD2 ARG 128 29.400 47.650 39.020 0.00 0.00 H

ATOM 1984 NE ARG 128 28.740 46.670 40.710 0.00 0.00 N

ATOM 1985 HE ARG 128 28.860 45.840 41.280 0.00 0.00 H

ATOM 1986 CZ ARG 128 27.490 47.050 40.740 0.00 0.00 C

ATOM 1987 NH1 ARG 128 27.030 48.200 40.250 0.00 0.00 N1+

ATOM 1988 HH11 ARG 128 27.540 48.710 39.550 0.00 0.00 H

ATOM 1989 HH12 ARG 128 26.020 48.300 40.270 0.00 0.00 H

ATOM 1990 NH2 ARG 128 26.630 46.220 41.360 0.00 0.00 N

ATOM 1991 HH21 ARG 128 26.920 45.360 41.800 0.00 0.00 H

ATOM 1992 HH22 ARG 128 25.650 46.410 41.520 0.00 0.00 H

ATOM 1993 C ARG 128 33.620 48.290 42.080 0.00 0.00 C

ATOM 1994 O ARG 128 33.140 48.320 43.220 0.00 0.00 O

ATOM 1995 N ALA 129 34.810 47.700 41.850 0.00 0.00 N

ATOM 1996 H ALA 129 35.200 47.870 40.930 0.00 0.00 H

ATOM 1997 CA ALA 129 35.490 46.820 42.790 0.00 0.00 C

ATOM 1998 HA ALA 129 34.770 46.080 43.130 0.00 0.00 H

ATOM 1999 CB ALA 129 36.590 46.030 42.140 0.00 0.00 C

ATOM 2000 HB1 ALA 129 37.340 46.630 41.620 0.00 0.00 H

ATOM 2001 HB2 ALA 129 37.060 45.290 42.790 0.00 0.00 H

ATOM 2002 HB3 ALA 129 36.090 45.430 41.370 0.00 0.00 H

ATOM 2003 C ALA 129 36.010 47.570 43.980 0.00 0.00 C

ATOM 2004 O ALA 129 35.820 47.190 45.090 0.00 0.00 O

ATOM 2005 N ALA 130 36.440 48.800 43.750 0.00 0.00 N

ATOM 2006 H ALA 130 36.390 49.140 42.790 0.00 0.00 H

ATOM 2007 CA ALA 130 36.790 49.750 44.730 0.00 0.00 C

ATOM 2008 HA ALA 130 37.680 49.460 45.290 0.00 0.00 H

ATOM 2009 CB ALA 130 37.190 51.130 44.070 0.00 0.00 C

ATOM 2010 HB1 ALA 130 37.970 50.980 43.320 0.00 0.00 H

ATOM 2011 HB2 ALA 130 36.350 51.720 43.690 0.00 0.00 H

ATOM 2012 HB3 ALA 130 37.680 51.760 44.810 0.00 0.00 H

ATOM 2013 C ALA 130 35.660 50.020 45.760 0.00 0.00 C

ATOM 2014 O ALA 130 35.890 49.990 46.990 0.00 0.00 O

ATOM 2015 N ALA 131 34.480 50.260 45.290 0.00 0.00 N

ATOM 2016 H ALA 131 34.360 50.140 44.290 0.00 0.00 H

ATOM 2017 CA ALA 131 33.260 50.260 46.070 0.00 0.00 C

ATOM 2018 HA ALA 131 33.480 51.000 46.840 0.00 0.00 H

ATOM 2019 CB ALA 131 32.090 50.750 45.250 0.00 0.00 C

ATOM 2020 HB1 ALA 131 32.330 51.570 44.580 0.00 0.00 H

ATOM 2021 HB2 ALA 131 31.620 49.950 44.670 0.00 0.00 H

ATOM 2022 HB3 ALA 131 31.280 51.070 45.910 0.00 0.00 H

ATOM 2023 C ALA 131 32.910 48.940 46.710 0.00 0.00 C

ATOM 2024 O ALA 131 32.520 48.970 47.880 0.00 0.00 O

ATOM 2025 N LEU 132 33.200 47.810 46.110 0.00 0.00 N

ATOM 2026 H LEU 132 33.750 47.940 45.280 0.00 0.00 H

ATOM 2027 CA LEU 132 32.950 46.530 46.690 0.00 0.00 C

ATOM 2028 HA LEU 132 31.950 46.530 47.130 0.00 0.00 H

ATOM 2029 CB LEU 132 32.980 45.440 45.640 0.00 0.00 C

ATOM 2030 HB1 LEU 132 33.890 45.480 45.040 0.00 0.00 H

ATOM 2031 HB2 LEU 132 32.140 45.590 44.960 0.00 0.00 H

ATOM 2032 CG LEU 132 32.910 43.940 46.140 0.00 0.00 C

ATOM 2033 HG LEU 132 33.570 43.670 46.970 0.00 0.00 H

ATOM 2034 CD1 LEU 132 31.520 43.680 46.630 0.00 0.00 C

ATOM 2035 HD11 LEU 132 31.360 42.720 47.130 0.00 0.00 H

ATOM 2036 HD12 LEU 132 31.310 44.410 47.420 0.00 0.00 H

ATOM 2037 HD13 LEU 132 30.820 43.820 45.810 0.00 0.00 H

ATOM 2038 CD2 LEU 132 33.160 43.040 44.920 0.00 0.00 C

ATOM 2039 HD21 LEU 132 32.640 43.450 44.050 0.00 0.00 H

ATOM 2040 HD22 LEU 132 34.210 42.880 44.710 0.00 0.00 H

ATOM 2041 HD23 LEU 132 32.750 42.040 45.060 0.00 0.00 H

ATOM 2042 C LEU 132 33.910 46.250 47.870 0.00 0.00 C

ATOM 2043 O LEU 132 33.420 45.850 48.900 0.00 0.00 O

ATOM 2044 N ILE 133 35.200 46.630 47.690 0.00 0.00 N

ATOM 2045 H ILE 133 35.450 47.020 46.800 0.00 0.00 H

ATOM 2046 CA ILE 133 36.310 46.510 48.670 0.00 0.00 C

ATOM 2047 HA ILE 133 36.350 45.490 49.070 0.00 0.00 H

ATOM 2048 CB ILE 133 37.740 46.810 48.160 0.00 0.00 C

ATOM 2049 HB ILE 133 37.600 47.740 47.610 0.00 0.00 H

ATOM 2050 CG2 ILE 133 38.740 46.920 49.290 0.00 0.00 C

ATOM 2051 HG21 ILE 133 39.710 47.000 48.800 0.00 0.00 H

ATOM 2052 HG22 ILE 133 38.570 47.820 49.880 0.00 0.00 H

ATOM 2053 HG23 ILE 133 38.740 46.010 49.880 0.00 0.00 H

ATOM 2054 CG1 ILE 133 38.190 45.710 47.130 0.00 0.00 C

ATOM 2055 HG11 ILE 133 37.300 45.200 46.760 0.00 0.00 H

ATOM 2056 HG12 ILE 133 38.840 44.940 47.540 0.00 0.00 H

ATOM 2057 CD ILE 133 39.070 46.230 45.970 0.00 0.00 C

ATOM 2058 HD1 ILE 133 39.000 45.440 45.220 0.00 0.00 H

ATOM 2059 HD2 ILE 133 38.550 47.130 45.630 0.00 0.00 H

ATOM 2060 HD3 ILE 133 40.070 46.410 46.340 0.00 0.00 H

ATOM 2061 C ILE 133 35.980 47.280 49.950 0.00 0.00 C

ATOM 2062 O ILE 133 36.160 46.800 51.060 0.00 0.00 O

ATOM 2063 N SER 134 35.480 48.500 49.720 0.00 0.00 N

ATOM 2064 H SER 134 35.400 48.750 48.750 0.00 0.00 H

ATOM 2065 CA SER 134 35.110 49.480 50.750 0.00 0.00 C

ATOM 2066 HA SER 134 35.890 49.780 51.450 0.00 0.00 H

ATOM 2067 CB SER 134 34.790 50.860 50.170 0.00 0.00 C

ATOM 2068 HB1 SER 134 34.510 51.490 51.010 0.00 0.00 H

ATOM 2069 HB2 SER 134 33.870 50.870 49.580 0.00 0.00 H

ATOM 2070 OG SER 134 35.900 51.300 49.440 0.00 0.00 O

ATOM 2071 HG SER 134 35.940 50.720 48.670 0.00 0.00 H

ATOM 2072 C SER 134 33.910 48.950 51.610 0.00 0.00 C

ATOM 2073 O SER 134 33.930 49.030 52.790 0.00 0.00 O

ATOM 2074 N LEU 135 32.880 48.360 50.920 0.00 0.00 N

ATOM 2075 H LEU 135 32.920 48.400 49.910 0.00 0.00 H

ATOM 2076 CA LEU 135 31.750 47.610 51.620 0.00 0.00 C

ATOM 2077 HA LEU 135 31.230 48.100 52.440 0.00 0.00 H

ATOM 2078 CB LEU 135 30.570 47.350 50.640 0.00 0.00 C

ATOM 2079 HB1 LEU 135 29.780 46.760 51.120 0.00 0.00 H

ATOM 2080 HB2 LEU 135 30.990 46.790 49.800 0.00 0.00 H

ATOM 2081 CG LEU 135 29.800 48.590 50.030 0.00 0.00 C

ATOM 2082 HG LEU 135 30.610 49.180 49.600 0.00 0.00 H

ATOM 2083 CD1 LEU 135 28.790 48.160 48.960 0.00 0.00 C

ATOM 2084 HD11 LEU 135 27.830 47.820 49.360 0.00 0.00 H

ATOM 2085 HD12 LEU 135 28.450 49.030 48.390 0.00 0.00 H

ATOM 2086 HD13 LEU 135 29.250 47.410 48.320 0.00 0.00 H

ATOM 2087 CD2 LEU 135 29.090 49.330 51.110 0.00 0.00 C

ATOM 2088 HD21 LEU 135 29.850 49.880 51.650 0.00 0.00 H

ATOM 2089 HD22 LEU 135 28.400 50.080 50.720 0.00 0.00 H

ATOM 2090 HD23 LEU 135 28.580 48.680 51.820 0.00 0.00 H

ATOM 2091 C LEU 135 32.280 46.260 52.210 0.00 0.00 C

ATOM 2092 O LEU 135 31.730 45.840 53.220 0.00 0.00 O

ATOM 2093 N THR 136 33.270 45.570 51.650 0.00 0.00 N

ATOM 2094 H THR 136 33.720 46.010 50.860 0.00 0.00 H

ATOM 2095 CA THR 136 33.840 44.290 52.180 0.00 0.00 C

ATOM 2096 HA THR 136 32.960 43.660 52.280 0.00 0.00 H

ATOM 2097 CB THR 136 34.700 43.580 51.160 0.00 0.00 C

ATOM 2098 HB THR 136 35.690 44.030 51.070 0.00 0.00 H

ATOM 2099 CG2 THR 136 34.860 42.060 51.620 0.00 0.00 C

ATOM 2100 HG21 THR 136 35.330 42.090 52.600 0.00 0.00 H

ATOM 2101 HG22 THR 136 33.850 41.640 51.570 0.00 0.00 H

ATOM 2102 HG23 THR 136 35.490 41.590 50.870 0.00 0.00 H

ATOM 2103 OG1 THR 136 34.060 43.480 49.910 0.00 0.00 O

ATOM 2104 HG1 THR 136 33.860 44.370 49.630 0.00 0.00 H

ATOM 2105 C THR 136 34.500 44.560 53.550 0.00 0.00 C

ATOM 2106 O THR 136 34.230 43.920 54.590 0.00 0.00 O

ATOM 2107 N TRP 137 35.320 45.640 53.580 0.00 0.00 N

ATOM 2108 H TRP 137 35.440 46.180 52.730 0.00 0.00 H

ATOM 2109 CA TRP 137 35.910 46.130 54.850 0.00 0.00 C

ATOM 2110 HA TRP 137 36.400 45.270 55.310 0.00 0.00 H

ATOM 2111 CB TRP 137 36.920 47.200 54.560 0.00 0.00 C

ATOM 2112 HB1 TRP 137 37.160 47.760 55.460 0.00 0.00 H

ATOM 2113 HB2 TRP 137 36.540 47.950 53.860 0.00 0.00 H

ATOM 2114 CG TRP 137 38.290 46.690 54.230 0.00 0.00 C

ATOM 2115 CD1 TRP 137 38.940 46.980 53.080 0.00 0.00 C

ATOM 2116 HD1 TRP 137 38.470 47.620 52.340 0.00 0.00 H

ATOM 2117 NE1 TRP 137 40.230 46.420 53.030 0.00 0.00 N

ATOM 2118 HE1 TRP 137 40.940 46.540 52.330 0.00 0.00 H

ATOM 2119 CE2 TRP 137 40.490 45.870 54.280 0.00 0.00 C

ATOM 2120 CZ2 TRP 137 41.660 45.410 54.750 0.00 0.00 C

ATOM 2121 HZ2 TRP 137 42.530 45.380 54.110 0.00 0.00 H

ATOM 2122 CH2 TRP 137 41.760 44.930 56.090 0.00 0.00 C

ATOM 2123 HH2 TRP 137 42.690 44.530 56.460 0.00 0.00 H

ATOM 2124 CZ3 TRP 137 40.610 44.980 56.870 0.00 0.00 C

ATOM 2125 HZ3 TRP 137 40.710 44.600 57.880 0.00 0.00 H

ATOM 2126 CE3 TRP 137 39.470 45.540 56.340 0.00 0.00 C

ATOM 2127 HE3 TRP 137 38.650 45.620 57.040 0.00 0.00 H

ATOM 2128 CD2 TRP 137 39.320 46.020 55.040 0.00 0.00 C

ATOM 2129 C TRP 137 34.910 46.580 55.840 0.00 0.00 C

ATOM 2130 O TRP 137 34.940 46.170 56.990 0.00 0.00 O

ATOM 2131 N LEU 138 33.910 47.400 55.510 0.00 0.00 N

ATOM 2132 H LEU 138 33.970 47.780 54.580 0.00 0.00 H

ATOM 2133 CA LEU 138 32.860 47.830 56.430 0.00 0.00 C

ATOM 2134 HA LEU 138 33.320 48.420 57.220 0.00 0.00 H

ATOM 2135 CB LEU 138 31.960 48.740 55.570 0.00 0.00 C

ATOM 2136 HB1 LEU 138 31.580 48.130 54.750 0.00 0.00 H

ATOM 2137 HB2 LEU 138 32.460 49.580 55.080 0.00 0.00 H

ATOM 2138 CG LEU 138 30.760 49.260 56.250 0.00 0.00 C

ATOM 2139 HG LEU 138 30.030 48.500 56.520 0.00 0.00 H

ATOM 2140 CD1 LEU 138 31.120 50.110 57.460 0.00 0.00 C

ATOM 2141 HD11 LEU 138 31.560 49.470 58.220 0.00 0.00 H

ATOM 2142 HD12 LEU 138 31.770 50.970 57.290 0.00 0.00 H

ATOM 2143 HD13 LEU 138 30.250 50.600 57.920 0.00 0.00 H

ATOM 2144 CD2 LEU 138 30.060 50.240 55.360 0.00 0.00 C

ATOM 2145 HD21 LEU 138 30.770 50.980 55.010 0.00 0.00 H

ATOM 2146 HD22 LEU 138 29.820 49.760 54.410 0.00 0.00 H

ATOM 2147 HD23 LEU 138 29.180 50.680 55.820 0.00 0.00 H

ATOM 2148 C LEU 138 31.900 46.720 56.930 0.00 0.00 C

ATOM 2149 O LEU 138 31.520 46.660 58.100 0.00 0.00 O

ATOM 2150 N ILE 139 31.650 45.660 56.200 0.00 0.00 N

ATOM 2151 H ILE 139 31.830 45.710 55.210 0.00 0.00 H

ATOM 2152 CA ILE 139 30.840 44.510 56.730 0.00 0.00 C

ATOM 2153 HA ILE 139 30.010 44.860 57.340 0.00 0.00 H

ATOM 2154 CB ILE 139 30.220 43.790 55.530 0.00 0.00 C

ATOM 2155 HB ILE 139 31.030 43.720 54.810 0.00 0.00 H

ATOM 2156 CG2 ILE 139 29.700 42.380 55.940 0.00 0.00 C

ATOM 2157 HG21 ILE 139 28.940 42.440 56.730 0.00 0.00 H

ATOM 2158 HG22 ILE 139 29.300 41.890 55.050 0.00 0.00 H

ATOM 2159 HG23 ILE 139 30.500 41.660 56.110 0.00 0.00 H

ATOM 2160 CG1 ILE 139 29.050 44.690 54.930 0.00 0.00 C

ATOM 2161 HG11 ILE 139 29.510 45.680 54.840 0.00 0.00 H

ATOM 2162 HG12 ILE 139 28.190 44.790 55.600 0.00 0.00 H

ATOM 2163 CD ILE 139 28.590 44.330 53.490 0.00 0.00 C

ATOM 2164 HD1 ILE 139 28.080 45.210 53.120 0.00 0.00 H

ATOM 2165 HD2 ILE 139 29.500 44.170 52.930 0.00 0.00 H

ATOM 2166 HD3 ILE 139 27.930 43.460 53.440 0.00 0.00 H

ATOM 2167 C ILE 139 31.690 43.510 57.550 0.00 0.00 C

ATOM 2168 O ILE 139 31.280 42.990 58.540 0.00 0.00 O

ATOM 2169 N GLY 140 32.950 43.450 57.190 0.00 0.00 N

ATOM 2170 H GLY 140 33.240 43.940 56.360 0.00 0.00 H

ATOM 2171 CA GLY 140 34.110 42.840 57.890 0.00 0.00 C

ATOM 2172 HA1 GLY 140 35.020 42.910 57.290 0.00 0.00 H

ATOM 2173 HA2 GLY 140 33.750 41.830 58.070 0.00 0.00 H

ATOM 2174 C GLY 140 34.350 43.540 59.300 0.00 0.00 C

ATOM 2175 O GLY 140 34.750 42.790 60.170 0.00 0.00 O

ATOM 2176 N PHE 141 34.070 44.790 59.450 0.00 0.00 N

ATOM 2177 H PHE 141 33.910 45.290 58.580 0.00 0.00 H

ATOM 2178 CA PHE 141 33.880 45.460 60.750 0.00 0.00 C

ATOM 2179 HA PHE 141 34.700 45.160 61.410 0.00 0.00 H

ATOM 2180 CB PHE 141 33.970 47.000 60.430 0.00 0.00 C

ATOM 2181 HB1 PHE 141 33.420 47.280 59.530 0.00 0.00 H

ATOM 2182 HB2 PHE 141 35.010 47.140 60.130 0.00 0.00 H

ATOM 2183 CG PHE 141 33.690 47.870 61.590 0.00 0.00 C

ATOM 2184 CD1 PHE 141 32.600 48.740 61.590 0.00 0.00 C

ATOM 2185 HD1 PHE 141 31.950 48.680 60.730 0.00 0.00 H

ATOM 2186 CE1 PHE 141 32.400 49.660 62.620 0.00 0.00 C

ATOM 2187 HE1 PHE 141 31.480 50.220 62.560 0.00 0.00 H

ATOM 2188 CZ PHE 141 33.390 49.820 63.630 0.00 0.00 C

ATOM 2189 HZ PHE 141 33.320 50.540 64.430 0.00 0.00 H

ATOM 2190 CE2 PHE 141 34.530 48.960 63.600 0.00 0.00 C

ATOM 2191 HE2 PHE 141 35.290 48.960 64.380 0.00 0.00 H

ATOM 2192 CD2 PHE 141 34.660 47.990 62.550 0.00 0.00 C

ATOM 2193 HD2 PHE 141 35.570 47.410 62.540 0.00 0.00 H

ATOM 2194 C PHE 141 32.550 45.180 61.460 0.00 0.00 C

ATOM 2195 O PHE 141 32.480 44.620 62.540 0.00 0.00 O

ATOM 2196 N LEU 142 31.410 45.390 60.800 0.00 0.00 N

ATOM 2197 H LEU 142 31.480 45.540 59.810 0.00 0.00 H

ATOM 2198 CA LEU 142 30.000 45.330 61.400 0.00 0.00 C

ATOM 2199 HA LEU 142 29.870 46.050 62.210 0.00 0.00 H

ATOM 2200 CB LEU 142 28.980 45.810 60.370 0.00 0.00 C

ATOM 2201 HB1 LEU 142 27.980 45.630 60.760 0.00 0.00 H

ATOM 2202 HB2 LEU 142 29.180 45.210 59.480 0.00 0.00 H

ATOM 2203 CG LEU 142 29.170 47.340 60.180 0.00 0.00 C

ATOM 2204 HG LEU 142 30.190 47.710 60.050 0.00 0.00 H

ATOM 2205 CD1 LEU 142 28.310 47.680 58.880 0.00 0.00 C

ATOM 2206 HD11 LEU 142 27.280 47.360 59.070 0.00 0.00 H

ATOM 2207 HD12 LEU 142 28.330 48.720 58.560 0.00 0.00 H

ATOM 2208 HD13 LEU 142 28.650 47.140 58.000 0.00 0.00 H

ATOM 2209 CD2 LEU 142 28.640 48.180 61.330 0.00 0.00 C

ATOM 2210 HD21 LEU 142 28.890 49.230 61.190 0.00 0.00 H

ATOM 2211 HD22 LEU 142 27.560 48.140 61.450 0.00 0.00 H

ATOM 2212 HD23 LEU 142 29.060 47.880 62.290 0.00 0.00 H

ATOM 2213 C LEU 142 29.650 43.910 61.990 0.00 0.00 C

ATOM 2214 O LEU 142 28.890 43.780 62.970 0.00 0.00 O

ATOM 2215 N ILE 143 30.190 42.840 61.410 0.00 0.00 N

ATOM 2216 H ILE 143 30.720 43.060 60.580 0.00 0.00 H

ATOM 2217 CA ILE 143 30.110 41.450 61.880 0.00 0.00 C

ATOM 2218 HA ILE 143 29.080 41.210 62.160 0.00 0.00 H

ATOM 2219 CB ILE 143 30.590 40.620 60.610 0.00 0.00 C

ATOM 2220 HB ILE 143 30.050 41.010 59.740 0.00 0.00 H

ATOM 2221 CG2 ILE 143 32.130 40.650 60.360 0.00 0.00 C

ATOM 2222 HG21 ILE 143 32.510 41.630 60.630 0.00 0.00 H

ATOM 2223 HG22 ILE 143 32.550 39.880 61.010 0.00 0.00 H

ATOM 2224 HG23 ILE 143 32.270 40.450 59.290 0.00 0.00 H

ATOM 2225 CG1 ILE 143 30.060 39.240 60.730 0.00 0.00 C

ATOM 2226 HG11 ILE 143 28.980 39.230 60.890 0.00 0.00 H

ATOM 2227 HG12 ILE 143 30.540 38.760 61.580 0.00 0.00 H

ATOM 2228 CD ILE 143 30.350 38.370 59.440 0.00 0.00 C

ATOM 2229 HD1 ILE 143 30.110 37.330 59.650 0.00 0.00 H

ATOM 2230 HD2 ILE 143 29.640 38.640 58.650 0.00 0.00 H

ATOM 2231 HD3 ILE 143 31.400 38.430 59.140 0.00 0.00 H

ATOM 2232 C ILE 143 31.020 41.120 63.150 0.00 0.00 C

ATOM 2233 O ILE 143 30.680 40.250 63.880 0.00 0.00 O

ATOM 2234 N SER 144 32.150 41.870 63.370 0.00 0.00 N

ATOM 2235 H SER 144 32.360 42.570 62.670 0.00 0.00 H

ATOM 2236 CA SER 144 33.250 41.450 64.270 0.00 0.00 C

ATOM 2237 HA SER 144 33.100 40.400 64.540 0.00 0.00 H

ATOM 2238 CB SER 144 34.570 41.610 63.470 0.00 0.00 C

ATOM 2239 HB1 SER 144 35.320 41.330 64.210 0.00 0.00 H

ATOM 2240 HB2 SER 144 34.630 42.640 63.130 0.00 0.00 H

ATOM 2241 OG SER 144 34.710 40.760 62.320 0.00 0.00 O

ATOM 2242 HG SER 144 34.900 41.350 61.590 0.00 0.00 H

ATOM 2243 C SER 144 33.270 42.290 65.550 0.00 0.00 C

ATOM 2244 O SER 144 34.240 42.250 66.280 0.00 0.00 O

ATOM 2245 N ILE 145 32.220 43.160 65.800 0.00 0.00 N

ATOM 2246 H ILE 145 31.470 43.130 65.130 0.00 0.00 H

ATOM 2247 CA ILE 145 32.240 44.040 66.990 0.00 0.00 C

ATOM 2248 HA ILE 145 33.140 44.640 66.900 0.00 0.00 H

ATOM 2249 CB ILE 145 31.100 45.080 66.890 0.00 0.00 C

ATOM 2250 HB ILE 145 30.200 44.480 66.770 0.00 0.00 H

ATOM 2251 CG2 ILE 145 30.830 45.860 68.200 0.00 0.00 C

ATOM 2252 HG21 ILE 145 30.750 45.200 69.070 0.00 0.00 H

ATOM 2253 HG22 ILE 145 31.670 46.510 68.450 0.00 0.00 H

ATOM 2254 HG23 ILE 145 29.880 46.340 68.010 0.00 0.00 H

ATOM 2255 CG1 ILE 145 31.310 45.990 65.640 0.00 0.00 C

ATOM 2256 HG11 ILE 145 31.480 45.430 64.720 0.00 0.00 H

ATOM 2257 HG12 ILE 145 32.250 46.510 65.830 0.00 0.00 H

ATOM 2258 CD ILE 145 30.190 46.990 65.370 0.00 0.00 C

ATOM 2259 HD1 ILE 145 30.560 47.590 64.530 0.00 0.00 H

ATOM 2260 HD2 ILE 145 29.310 46.400 65.100 0.00 0.00 H

ATOM 2261 HD3 ILE 145 30.010 47.680 66.190 0.00 0.00 H

ATOM 2262 C ILE 145 32.330 43.340 68.360 0.00 0.00 C

ATOM 2263 O ILE 145 33.010 43.890 69.170 0.00 0.00 O

ATOM 2264 N PRO 146 31.770 42.150 68.660 0.00 0.00 N

ATOM 2265 CD PRO 146 30.560 41.550 68.010 0.00 0.00 C

ATOM 2266 HD1 PRO 146 30.710 40.750 67.280 0.00 0.00 H

ATOM 2267 HD2 PRO 146 29.920 42.280 67.500 0.00 0.00 H

ATOM 2268 CG PRO 146 29.780 40.940 69.120 0.00 0.00 C

ATOM 2269 HG1 PRO 146 29.250 40.100 68.680 0.00 0.00 H

ATOM 2270 HG2 PRO 146 29.050 41.580 69.610 0.00 0.00 H

ATOM 2271 CB PRO 146 30.800 40.350 70.060 0.00 0.00 C

ATOM 2272 HB1 PRO 146 31.120 39.340 69.820 0.00 0.00 H

ATOM 2273 HB2 PRO 146 30.380 40.320 71.070 0.00 0.00 H

ATOM 2274 CA PRO 146 31.820 41.490 69.950 0.00 0.00 C

ATOM 2275 HA PRO 146 31.570 42.260 70.690 0.00 0.00 H

ATOM 2276 C PRO 146 33.260 41.190 70.470 0.00 0.00 C

ATOM 2277 O PRO 146 33.630 41.640 71.540 0.00 0.00 O

ATOM 2278 N PRO 147 34.130 40.480 69.670 0.00 0.00 N

ATOM 2279 CD PRO 147 33.650 39.510 68.670 0.00 0.00 C

ATOM 2280 HD1 PRO 147 32.990 40.000 67.960 0.00 0.00 H

ATOM 2281 HD2 PRO 147 33.170 38.710 69.240 0.00 0.00 H

ATOM 2282 CG PRO 147 34.970 39.090 67.940 0.00 0.00 C

ATOM 2283 HG1 PRO 147 35.160 39.810 67.150 0.00 0.00 H

ATOM 2284 HG2 PRO 147 34.850 38.040 67.690 0.00 0.00 H

ATOM 2285 CB PRO 147 36.100 39.180 68.980 0.00 0.00 C

ATOM 2286 HB1 PRO 147 37.090 39.390 68.580 0.00 0.00 H

ATOM 2287 HB2 PRO 147 35.940 38.280 69.570 0.00 0.00 H

ATOM 2288 CA PRO 147 35.620 40.340 69.890 0.00 0.00 C

ATOM 2289 HA PRO 147 35.740 39.960 70.900 0.00 0.00 H

ATOM 2290 C PRO 147 36.470 41.600 69.660 0.00 0.00 C

ATOM 2291 O PRO 147 37.610 41.700 70.110 0.00 0.00 O

ATOM 2292 N MET 148 35.900 42.650 69.040 0.00 0.00 N

ATOM 2293 H MET 148 35.060 42.480 68.510 0.00 0.00 H

ATOM 2294 CA MET 148 36.700 43.820 68.720 0.00 0.00 C

ATOM 2295 HA MET 148 37.740 43.550 68.580 0.00 0.00 H

ATOM 2296 CB MET 148 36.180 44.390 67.360 0.00 0.00 C

ATOM 2297 HB1 MET 148 35.150 44.750 67.470 0.00 0.00 H

ATOM 2298 HB2 MET 148 36.210 43.560 66.650 0.00 0.00 H

ATOM 2299 CG MET 148 37.030 45.570 66.900 0.00 0.00 C

ATOM 2300 HG1 MET 148 38.050 45.190 66.810 0.00 0.00 H

ATOM 2301 HG2 MET 148 37.000 46.320 67.690 0.00 0.00 H

ATOM 2302 SD MET 148 36.440 46.260 65.310 0.00 0.00 S

ATOM 2303 CE MET 148 37.370 47.770 65.160 0.00 0.00 C

ATOM 2304 HE1 MET 148 37.260 48.210 64.170 0.00 0.00 H

ATOM 2305 HE2 MET 148 36.950 48.470 65.880 0.00 0.00 H

ATOM 2306 HE3 MET 148 38.410 47.560 65.420 0.00 0.00 H

ATOM 2307 C MET 148 36.620 44.830 69.850 0.00 0.00 C

ATOM 2308 O MET 148 37.650 45.350 70.260 0.00 0.00 O

ATOM 2309 N LEU 149 35.420 45.070 70.390 0.00 0.00 N

ATOM 2310 H LEU 149 34.650 44.470 70.150 0.00 0.00 H

ATOM 2311 CA LEU 149 35.120 46.270 71.180 0.00 0.00 C

ATOM 2312 HA LEU 149 35.990 46.730 71.640 0.00 0.00 H

ATOM 2313 CB LEU 149 34.640 47.330 70.160 0.00 0.00 C

ATOM 2314 HB1 LEU 149 33.570 47.140 70.060 0.00 0.00 H

ATOM 2315 HB2 LEU 149 35.060 47.260 69.160 0.00 0.00 H

ATOM 2316 CG LEU 149 34.890 48.750 70.670 0.00 0.00 C

ATOM 2317 HG LEU 149 34.630 48.830 71.720 0.00 0.00 H

ATOM 2318 CD1 LEU 149 36.430 49.200 70.690 0.00 0.00 C

ATOM 2319 HD11 LEU 149 36.530 50.170 71.180 0.00 0.00 H

ATOM 2320 HD12 LEU 149 36.940 48.610 71.450 0.00 0.00 H

ATOM 2321 HD13 LEU 149 36.990 49.320 69.770 0.00 0.00 H

ATOM 2322 CD2 LEU 149 34.080 49.770 69.820 0.00 0.00 C

ATOM 2323 HD21 LEU 149 33.020 49.520 69.780 0.00 0.00 H

ATOM 2324 HD22 LEU 149 34.260 50.810 70.100 0.00 0.00 H

ATOM 2325 HD23 LEU 149 34.430 49.600 68.800 0.00 0.00 H

ATOM 2326 C LEU 149 34.180 45.810 72.290 0.00 0.00 C

ATOM 2327 O LEU 149 34.310 46.360 73.410 0.00 0.00 O

ATOM 2328 N GLY 150 33.290 44.860 71.990 0.00 0.00 N

ATOM 2329 H GLY 150 33.250 44.460 71.060 0.00 0.00 H

ATOM 2330 CA GLY 150 32.320 44.400 73.010 0.00 0.00 C

ATOM 2331 HA1 GLY 150 31.380 44.070 72.550 0.00 0.00 H

ATOM 2332 HA2 GLY 150 32.080 45.230 73.670 0.00 0.00 H

ATOM 2333 C GLY 150 32.770 43.400 74.040 0.00 0.00 C

ATOM 2334 O GLY 150 32.220 42.300 74.200 0.00 0.00 O

ATOM 2335 N TRP 151 33.840 43.780 74.760 0.00 0.00 N

ATOM 2336 H TRP 151 34.200 44.690 74.520 0.00 0.00 H

ATOM 2337 CA TRP 151 34.670 43.070 75.760 0.00 0.00 C

ATOM 2338 HA TRP 151 33.980 42.610 76.470 0.00 0.00 H

ATOM 2339 CB TRP 151 35.380 41.940 75.050 0.00 0.00 C

ATOM 2340 HB1 TRP 151 34.700 41.370 74.420 0.00 0.00 H

ATOM 2341 HB2 TRP 151 35.720 41.260 75.820 0.00 0.00 H

ATOM 2342 CG TRP 151 36.610 42.370 74.270 0.00 0.00 C

ATOM 2343 CD1 TRP 151 36.540 42.950 73.060 0.00 0.00 C

ATOM 2344 HD1 TRP 151 35.670 42.930 72.420 0.00 0.00 H

ATOM 2345 NE1 TRP 151 37.740 43.380 72.630 0.00 0.00 N

ATOM 2346 HE1 TRP 151 37.960 43.780 71.730 0.00 0.00 H

ATOM 2347 CE2 TRP 151 38.700 43.300 73.630 0.00 0.00 C

ATOM 2348 CZ2 TRP 151 40.040 43.670 73.750 0.00 0.00 C

ATOM 2349 HZ2 TRP 151 40.460 44.290 72.970 0.00 0.00 H

ATOM 2350 CH2 TRP 151 40.660 43.450 74.990 0.00 0.00 C

ATOM 2351 HH2 TRP 151 41.730 43.620 75.040 0.00 0.00 H

ATOM 2352 CZ3 TRP 151 40.140 42.700 75.990 0.00 0.00 C

ATOM 2353 HZ3 TRP 151 40.770 42.470 76.840 0.00 0.00 H

ATOM 2354 CE3 TRP 151 38.820 42.310 75.850 0.00 0.00 C

ATOM 2355 HE3 TRP 151 38.400 41.760 76.680 0.00 0.00 H

ATOM 2356 CD2 TRP 151 38.030 42.580 74.690 0.00 0.00 C

ATOM 2357 C TRP 151 35.680 44.070 76.430 0.00 0.00 C

ATOM 2358 O TRP 151 36.110 45.100 75.820 0.00 0.00 O

ATOM 2359 N ARG 152 36.190 43.650 77.590 0.00 0.00 N

ATOM 2360 H ARG 152 35.680 42.870 77.980 0.00 0.00 H

ATOM 2361 CA ARG 152 37.370 44.120 78.340 0.00 0.00 C

ATOM 2362 HA ARG 152 38.230 44.510 77.780 0.00 0.00 H

ATOM 2363 CB ARG 152 36.810 45.390 79.040 0.00 0.00 C

ATOM 2364 HB1 ARG 152 36.040 45.150 79.770 0.00 0.00 H

ATOM 2365 HB2 ARG 152 36.310 46.120 78.410 0.00 0.00 H

ATOM 2366 CG ARG 152 37.920 46.080 79.830 0.00 0.00 C

ATOM 2367 HG1 ARG 152 38.650 46.380 79.090 0.00 0.00 H

ATOM 2368 HG2 ARG 152 38.330 45.410 80.590 0.00 0.00 H

ATOM 2369 CD ARG 152 37.400 47.360 80.500 0.00 0.00 C

ATOM 2370 HD1 ARG 152 36.530 47.270 81.150 0.00 0.00 H

ATOM 2371 HD2 ARG 152 36.990 47.960 79.680 0.00 0.00 H

ATOM 2372 NE ARG 152 38.490 48.070 81.150 0.00 0.00 N

ATOM 2373 HE ARG 152 39.250 48.330 80.530 0.00 0.00 H

ATOM 2374 CZ ARG 152 38.560 48.290 82.470 0.00 0.00 C

ATOM 2375 NH1 ARG 152 37.660 47.990 83.400 0.00 0.00 N1+

ATOM 2376 HH11 ARG 152 36.820 47.460 83.210 0.00 0.00 H

ATOM 2377 HH12 ARG 152 37.900 48.320 84.320 0.00 0.00 H

ATOM 2378 NH2 ARG 152 39.590 48.920 82.870 0.00 0.00 N

ATOM 2379 HH21 ARG 152 40.290 49.120 82.180 0.00 0.00 H

ATOM 2380 HH22 ARG 152 39.620 49.400 83.760 0.00 0.00 H

ATOM 2381 C ARG 152 37.780 43.030 79.330 0.00 0.00 C

ATOM 2382 O ARG 152 37.060 42.750 80.310 0.00 0.00 O

ATOM 2383 N THR 153 39.030 42.650 79.340 0.00 0.00 N

ATOM 2384 H THR 153 39.550 42.930 78.530 0.00 0.00 H

ATOM 2385 CA THR 153 39.760 41.610 80.210 0.00 0.00 C

ATOM 2386 HA THR 153 39.050 40.780 80.260 0.00 0.00 H

ATOM 2387 CB THR 153 40.920 40.920 79.460 0.00 0.00 C

ATOM 2388 HB THR 153 41.620 41.740 79.310 0.00 0.00 H

ATOM 2389 CG2 THR 153 41.590 39.830 80.200 0.00 0.00 C

ATOM 2390 HG21 THR 153 42.450 39.540 79.600 0.00 0.00 H

ATOM 2391 HG22 THR 153 41.980 40.240 81.140 0.00 0.00 H

ATOM 2392 HG23 THR 153 40.840 39.040 80.230 0.00 0.00 H

ATOM 2393 OG1 THR 153 40.410 40.360 78.290 0.00 0.00 O

ATOM 2394 HG1 THR 153 41.230 40.030 77.900 0.00 0.00 H

ATOM 2395 C THR 153 40.070 42.160 81.570 0.00 0.00 C

ATOM 2396 O THR 153 39.900 41.340 82.510 0.00 0.00 O

ATOM 2397 N PRO 154 40.600 43.390 81.770 0.00 0.00 N

ATOM 2398 CD PRO 154 40.990 44.270 80.730 0.00 0.00 C

ATOM 2399 HD1 PRO 154 40.100 44.570 80.180 0.00 0.00 H

ATOM 2400 HD2 PRO 154 41.700 43.740 80.080 0.00 0.00 H

ATOM 2401 CG PRO 154 41.630 45.460 81.420 0.00 0.00 C

ATOM 2402 HG1 PRO 154 40.820 46.140 81.640 0.00 0.00 H

ATOM 2403 HG2 PRO 154 42.460 45.930 80.880 0.00 0.00 H

ATOM 2404 CB PRO 154 42.030 44.910 82.780 0.00 0.00 C

ATOM 2405 HB1 PRO 154 42.160 45.610 83.600 0.00 0.00 H

ATOM 2406 HB2 PRO 154 42.890 44.250 82.610 0.00 0.00 H

ATOM 2407 CA PRO 154 40.950 43.910 83.080 0.00 0.00 C

ATOM 2408 HA PRO 154 41.290 43.050 83.650 0.00 0.00 H

ATOM 2409 C PRO 154 39.710 44.480 83.770 0.00 0.00 C

ATOM 2410 O PRO 154 38.620 44.730 83.190 0.00 0.00 O

ATOM 2411 N GLU 155 39.850 44.920 85.030 0.00 0.00 N

ATOM 2412 H GLU 155 40.690 44.690 85.550 0.00 0.00 H

ATOM 2413 CA GLU 155 38.940 45.750 85.740 0.00 0.00 C

ATOM 2414 HA GLU 155 38.020 45.980 85.200 0.00 0.00 H

ATOM 2415 CB GLU 155 38.520 45.110 87.090 0.00 0.00 C

ATOM 2416 HB1 GLU 155 37.970 44.180 86.960 0.00 0.00 H

ATOM 2417 HB2 GLU 155 37.900 45.780 87.680 0.00 0.00 H

ATOM 2418 CG GLU 155 39.610 44.640 88.090 0.00 0.00 C

ATOM 2419 HG1 GLU 155 40.180 45.500 88.450 0.00 0.00 H

ATOM 2420 HG2 GLU 155 40.250 43.930 87.570 0.00 0.00 H

ATOM 2421 CD GLU 155 38.950 43.960 89.320 0.00 0.00 C

ATOM 2422 OE1 GLU 155 39.370 42.840 89.690 0.00 0.00 O

ATOM 2423 OE2 GLU 155 38.100 44.570 90.010 0.00 0.00 O1-

ATOM 2424 C GLU 155 39.550 47.160 86.010 0.00 0.00 C

ATOM 2425 O GLU 155 38.840 48.140 86.080 0.00 0.00 O

ATOM 2426 N ASP 156 40.910 47.250 86.060 0.00 0.00 N

ATOM 2427 H ASP 156 41.440 46.400 86.200 0.00 0.00 H

ATOM 2428 CA ASP 156 41.700 48.490 86.470 0.00 0.00 C

ATOM 2429 HA ASP 156 41.160 49.410 86.240 0.00 0.00 H

ATOM 2430 CB ASP 156 42.090 48.380 87.950 0.00 0.00 C

ATOM 2431 HB1 ASP 156 42.780 47.540 88.090 0.00 0.00 H

ATOM 2432 HB2 ASP 156 41.260 48.210 88.630 0.00 0.00 H

ATOM 2433 CG ASP 156 42.780 49.620 88.450 0.00 0.00 C

ATOM 2434 OD1 ASP 156 41.990 50.610 88.660 0.00 0.00 O

ATOM 2435 OD2 ASP 156 44.050 49.720 88.440 0.00 0.00 O1-

ATOM 2436 C ASP 156 43.020 48.590 85.670 0.00 0.00 C

ATOM 2437 O ASP 156 43.460 49.730 85.280 0.00 0.00 O

ATOM 2438 N ARG 157 43.720 47.430 85.430 0.00 0.00 N

ATOM 2439 H ARG 157 43.190 46.580 85.560 0.00 0.00 H

ATOM 2440 CA ARG 157 44.990 47.260 84.770 0.00 0.00 C

ATOM 2441 HA ARG 157 45.750 47.780 85.350 0.00 0.00 H

ATOM 2442 CB ARG 157 45.290 45.780 84.800 0.00 0.00 C

ATOM 2443 HB1 ARG 157 44.500 45.180 84.350 0.00 0.00 H

ATOM 2444 HB2 ARG 157 45.380 45.460 85.840 0.00 0.00 H

ATOM 2445 CG ARG 157 46.560 45.420 83.970 0.00 0.00 C

ATOM 2446 HG1 ARG 157 47.460 45.960 84.280 0.00 0.00 H

ATOM 2447 HG2 ARG 157 46.480 45.570 82.900 0.00 0.00 H

ATOM 2448 CD ARG 157 46.900 43.910 84.230 0.00 0.00 C

ATOM 2449 HD1 ARG 157 47.030 43.720 85.300 0.00 0.00 H

ATOM 2450 HD2 ARG 157 47.810 43.550 83.750 0.00 0.00 H

ATOM 2451 NE ARG 157 45.840 43.020 83.630 0.00 0.00 N

ATOM 2452 HE ARG 157 45.590 43.240 82.680 0.00 0.00 H

ATOM 2453 CZ ARG 157 45.250 41.970 84.140 0.00 0.00 C

ATOM 2454 NH1 ARG 157 45.440 41.560 85.370 0.00 0.00 N1+

ATOM 2455 HH11 ARG 157 45.970 42.200 85.950 0.00 0.00 H

ATOM 2456 HH12 ARG 157 44.870 40.850 85.810 0.00 0.00 H

ATOM 2457 NH2 ARG 157 44.520 41.200 83.390 0.00 0.00 N

ATOM 2458 HH21 ARG 157 44.390 41.540 82.450 0.00 0.00 H

ATOM 2459 HH22 ARG 157 44.110 40.350 83.730 0.00 0.00 H

ATOM 2460 C ARG 157 45.020 47.970 83.410 0.00 0.00 C

ATOM 2461 O ARG 157 44.270 47.720 82.470 0.00 0.00 O

ATOM 2462 N SER 158 46.150 48.660 83.250 0.00 0.00 N

ATOM 2463 H SER 158 46.810 48.560 84.010 0.00 0.00 H

ATOM 2464 CA SER 158 46.660 49.150 81.950 0.00 0.00 C

ATOM 2465 HA SER 158 46.030 48.940 81.090 0.00 0.00 H

ATOM 2466 CB SER 158 46.860 50.670 82.090 0.00 0.00 C

ATOM 2467 HB1 SER 158 47.490 51.020 81.270 0.00 0.00 H

ATOM 2468 HB2 SER 158 47.280 50.880 83.080 0.00 0.00 H

ATOM 2469 OG SER 158 45.680 51.370 81.790 0.00 0.00 O

ATOM 2470 HG SER 158 45.870 52.280 82.030 0.00 0.00 H

ATOM 2471 C SER 158 48.040 48.590 81.580 0.00 0.00 C

ATOM 2472 O SER 158 48.570 48.830 80.500 0.00 0.00 O

ATOM 2473 N ASP 159 48.590 47.690 82.380 0.00 0.00 N

ATOM 2474 H ASP 159 48.180 47.600 83.300 0.00 0.00 H

ATOM 2475 CA ASP 159 49.960 47.040 82.140 0.00 0.00 C

ATOM 2476 HA ASP 159 50.350 47.310 81.160 0.00 0.00 H

ATOM 2477 CB ASP 159 51.030 47.570 83.150 0.00 0.00 C

ATOM 2478 HB1 ASP 159 50.710 47.300 84.150 0.00 0.00 H

ATOM 2479 HB2 ASP 159 50.970 48.660 83.080 0.00 0.00 H

ATOM 2480 CG ASP 159 52.400 47.000 82.870 0.00 0.00 C

ATOM 2481 OD1 ASP 159 52.640 46.940 81.620 0.00 0.00 O

ATOM 2482 OD2 ASP 159 53.310 46.900 83.760 0.00 0.00 O1-

ATOM 2483 C ASP 159 49.830 45.500 82.160 0.00 0.00 C

ATOM 2484 O ASP 159 50.250 44.880 83.190 0.00 0.00 O

ATOM 2485 N PRO 160 49.190 44.860 81.170 0.00 0.00 N

ATOM 2486 CD PRO 160 48.580 45.540 80.030 0.00 0.00 C

ATOM 2487 HD1 PRO 160 49.350 46.100 79.480 0.00 0.00 H

ATOM 2488 HD2 PRO 160 47.800 46.280 80.240 0.00 0.00 H

ATOM 2489 CG PRO 160 47.990 44.460 79.050 0.00 0.00 C

ATOM 2490 HG1 PRO 160 48.310 44.650 78.030 0.00 0.00 H

ATOM 2491 HG2 PRO 160 46.900 44.380 79.130 0.00 0.00 H

ATOM 2492 CB PRO 160 48.530 43.170 79.700 0.00 0.00 C

ATOM 2493 HB1 PRO 160 49.260 42.620 79.120 0.00 0.00 H

ATOM 2494 HB2 PRO 160 47.630 42.570 79.860 0.00 0.00 H

ATOM 2495 CA PRO 160 49.210 43.390 81.040 0.00 0.00 C

ATOM 2496 HA PRO 160 48.520 43.000 81.790 0.00 0.00 H

ATOM 2497 C PRO 160 50.580 42.710 81.130 0.00 0.00 C

ATOM 2498 O PRO 160 51.560 43.060 80.500 0.00 0.00 O

ATOM 2499 N ASP 161 50.590 41.650 81.930 0.00 0.00 N

ATOM 2500 H ASP 161 49.720 41.360 82.340 0.00 0.00 H

ATOM 2501 CA ASP 161 51.610 40.620 82.090 0.00 0.00 C

ATOM 2502 HA ASP 161 52.570 41.150 82.080 0.00 0.00 H

ATOM 2503 CB ASP 161 51.360 39.820 83.350 0.00 0.00 C

ATOM 2504 HB1 ASP 161 50.460 39.220 83.210 0.00 0.00 H

ATOM 2505 HB2 ASP 161 51.120 40.480 84.180 0.00 0.00 H

ATOM 2506 CG ASP 161 52.550 38.860 83.720 0.00 0.00 C

ATOM 2507 OD1 ASP 161 52.380 37.580 83.730 0.00 0.00 O

ATOM 2508 OD2 ASP 161 53.570 39.390 84.110 0.00 0.00 O1-

ATOM 2509 C ASP 161 51.510 39.690 80.930 0.00 0.00 C

ATOM 2510 O ASP 161 52.520 39.130 80.510 0.00 0.00 O

ATOM 2511 N ALA 162 50.360 39.560 80.320 0.00 0.00 N

ATOM 2512 H ALA 162 49.660 40.010 80.890 0.00 0.00 H

ATOM 2513 CA ALA 162 50.020 38.740 79.130 0.00 0.00 C

ATOM 2514 HA ALA 162 50.880 38.660 78.460 0.00 0.00 H

ATOM 2515 CB ALA 162 49.750 37.290 79.630 0.00 0.00 C

ATOM 2516 HB1 ALA 162 50.600 37.000 80.260 0.00 0.00 H

ATOM 2517 HB2 ALA 162 48.840 37.150 80.210 0.00 0.00 H

ATOM 2518 HB3 ALA 162 49.860 36.570 78.830 0.00 0.00 H

ATOM 2519 C ALA 162 48.830 39.360 78.310 0.00 0.00 C

ATOM 2520 O ALA 162 48.050 40.190 78.900 0.00 0.00 O

ATOM 2521 N CYS 163 48.500 38.950 77.080 0.00 0.00 N

ATOM 2522 H CYS 163 49.160 38.340 76.620 0.00 0.00 H

ATOM 2523 CA CYS 163 47.390 39.590 76.290 0.00 0.00 C

ATOM 2524 HA CYS 163 46.870 40.310 76.920 0.00 0.00 H

ATOM 2525 CB CYS 163 47.990 40.380 75.160 0.00 0.00 C

ATOM 2526 HB1 CYS 163 48.610 39.700 74.570 0.00 0.00 H

ATOM 2527 HB2 CYS 163 48.700 41.020 75.680 0.00 0.00 H

ATOM 2528 SG CYS 163 46.870 41.320 74.140 0.00 0.00 S

ATOM 2529 C CYS 163 46.290 38.620 75.850 0.00 0.00 C

ATOM 2530 O CYS 163 46.420 37.830 74.930 0.00 0.00 O

ATOM 2531 N THR 164 45.100 38.740 76.510 0.00 0.00 N

ATOM 2532 H THR 164 45.020 39.420 77.260 0.00 0.00 H

ATOM 2533 CA THR 164 44.070 37.750 76.350 0.00 0.00 C

ATOM 2534 HA THR 164 44.240 37.110 75.480 0.00 0.00 H

ATOM 2535 CB THR 164 44.080 36.830 77.580 0.00 0.00 C

ATOM 2536 HB THR 164 43.790 37.400 78.470 0.00 0.00 H

ATOM 2537 CG2 THR 164 43.190 35.520 77.340 0.00 0.00 C

ATOM 2538 HG21 THR 164 42.160 35.840 77.520 0.00 0.00 H

ATOM 2539 HG22 THR 164 43.280 35.200 76.310 0.00 0.00 H

ATOM 2540 HG23 THR 164 43.540 34.730 78.010 0.00 0.00 H

ATOM 2541 OG1 THR 164 45.320 36.310 77.750 0.00 0.00 O

ATOM 2542 HG1 THR 164 45.620 35.950 76.900 0.00 0.00 H

ATOM 2543 C THR 164 42.670 38.420 76.360 0.00 0.00 C

ATOM 2544 O THR 164 42.520 39.480 76.970 0.00 0.00 O

ATOM 2545 N ILE 165 41.640 37.850 75.670 0.00 0.00 N

ATOM 2546 H ILE 165 41.760 36.980 75.170 0.00 0.00 H

ATOM 2547 CA ILE 165 40.350 38.530 75.630 0.00 0.00 C

ATOM 2548 HA ILE 165 40.480 39.590 75.870 0.00 0.00 H

ATOM 2549 CB ILE 165 39.660 38.350 74.250 0.00 0.00 C

ATOM 2550 HB ILE 165 40.490 38.210 73.550 0.00 0.00 H

ATOM 2551 CG2 ILE 165 38.930 37.080 74.200 0.00 0.00 C

ATOM 2552 HG21 ILE 165 39.470 36.290 74.730 0.00 0.00 H

ATOM 2553 HG22 ILE 165 37.930 36.970 74.630 0.00 0.00 H

ATOM 2554 HG23 ILE 165 38.790 36.710 73.190 0.00 0.00 H

ATOM 2555 CG1 ILE 165 38.830 39.530 73.710 0.00 0.00 C

ATOM 2556 HG11 ILE 165 39.620 40.280 73.630 0.00 0.00 H

ATOM 2557 HG12 ILE 165 38.080 39.900 74.420 0.00 0.00 H

ATOM 2558 CD ILE 165 38.150 39.380 72.400 0.00 0.00 C

ATOM 2559 HD1 ILE 165 38.790 39.230 71.530 0.00 0.00 H

ATOM 2560 HD2 ILE 165 37.480 38.520 72.330 0.00 0.00 H

ATOM 2561 HD3 ILE 165 37.590 40.310 72.310 0.00 0.00 H

ATOM 2562 C ILE 165 39.500 38.090 76.840 0.00 0.00 C

ATOM 2563 O ILE 165 39.830 37.090 77.490 0.00 0.00 O

ATOM 2564 N SER 166 38.350 38.630 77.050 0.00 0.00 N

ATOM 2565 H SER 166 38.160 39.540 76.650 0.00 0.00 H

ATOM 2566 CA SER 166 37.350 38.070 77.900 0.00 0.00 C

ATOM 2567 HA SER 166 37.710 37.370 78.650 0.00 0.00 H

ATOM 2568 CB SER 166 36.660 39.180 78.710 0.00 0.00 C

ATOM 2569 HB1 SER 166 37.220 39.250 79.650 0.00 0.00 H

ATOM 2570 HB2 SER 166 35.710 38.800 79.080 0.00 0.00 H

ATOM 2571 OG SER 166 36.610 40.340 77.950 0.00 0.00 O

ATOM 2572 HG SER 166 35.790 40.740 78.260 0.00 0.00 H

ATOM 2573 C SER 166 36.270 37.200 77.140 0.00 0.00 C

ATOM 2574 O SER 166 35.970 37.340 76.000 0.00 0.00 O

ATOM 2575 N LYS 167 35.800 36.160 77.880 0.00 0.00 N

ATOM 2576 H LYS 167 36.190 36.060 78.810 0.00 0.00 H

ATOM 2577 CA LYS 167 34.920 34.990 77.400 0.00 0.00 C

ATOM 2578 HA LYS 167 34.510 35.320 76.440 0.00 0.00 H

ATOM 2579 CB LYS 167 35.830 33.730 77.200 0.00 0.00 C

ATOM 2580 HB1 LYS 167 36.650 33.940 76.510 0.00 0.00 H

ATOM 2581 HB2 LYS 167 35.200 32.980 76.730 0.00 0.00 H

ATOM 2582 CG LYS 167 36.500 33.310 78.510 0.00 0.00 C

ATOM 2583 HG1 LYS 167 35.790 33.030 79.290 0.00 0.00 H

ATOM 2584 HG2 LYS 167 37.090 34.160 78.870 0.00 0.00 H

ATOM 2585 CD LYS 167 37.440 32.040 78.400 0.00 0.00 C

ATOM 2586 HD1 LYS 167 37.970 32.100 77.450 0.00 0.00 H

ATOM 2587 HD2 LYS 167 36.870 31.120 78.260 0.00 0.00 H

ATOM 2588 CE LYS 167 38.350 32.000 79.620 0.00 0.00 C

ATOM 2589 HE1 LYS 167 37.750 31.990 80.530 0.00 0.00 H

ATOM 2590 HE2 LYS 167 38.930 32.910 79.750 0.00 0.00 H

ATOM 2591 NZ LYS 167 39.250 30.820 79.610 0.00 0.00 N1+

ATOM 2592 HZ1 LYS 167 38.800 29.920 79.620 0.00 0.00 H

ATOM 2593 HZ2 LYS 167 39.810 30.770 78.770 0.00 0.00 H

ATOM 2594 HZ3 LYS 167 39.850 30.810 80.430 0.00 0.00 H

ATOM 2595 C LYS 167 33.710 34.700 78.320 0.00 0.00 C

ATOM 2596 O LYS 167 32.790 33.940 77.980 0.00 0.00 O

ATOM 2597 N ASP 168 33.790 35.340 79.500 0.00 0.00 N

ATOM 2598 H ASP 168 34.630 35.870 79.690 0.00 0.00 H

ATOM 2599 CA ASP 168 32.810 35.080 80.590 0.00 0.00 C

ATOM 2600 HA ASP 168 33.000 34.090 81.010 0.00 0.00 H

ATOM 2601 CB ASP 168 33.140 36.070 81.750 0.00 0.00 C

ATOM 2602 HB1 ASP 168 32.890 37.060 81.360 0.00 0.00 H

ATOM 2603 HB2 ASP 168 34.160 36.160 82.100 0.00 0.00 H

ATOM 2604 CG ASP 168 32.210 35.900 82.930 0.00 0.00 C

ATOM 2605 OD1 ASP 168 32.090 34.790 83.500 0.00 0.00 O

ATOM 2606 OD2 ASP 168 31.660 36.950 83.390 0.00 0.00 O1-

ATOM 2607 C ASP 168 31.320 35.170 80.180 0.00 0.00 C

ATOM 2608 O ASP 168 30.510 34.610 80.900 0.00 0.00 O

ATOM 2609 N HIS 169 31.050 36.070 79.210 0.00 0.00 N

ATOM 2610 H HIS 169 31.840 36.400 78.660 0.00 0.00 H

ATOM 2611 CA HIS 169 29.690 36.540 78.850 0.00 0.00 C

ATOM 2612 HA HIS 169 29.170 36.750 79.780 0.00 0.00 H

ATOM 2613 CB HIS 169 30.010 37.860 78.040 0.00 0.00 C

ATOM 2614 HB1 HIS 169 30.260 38.520 78.870 0.00 0.00 H

ATOM 2615 HB2 HIS 169 29.090 38.230 77.600 0.00 0.00 H

ATOM 2616 CG HIS 169 31.130 37.690 77.040 0.00 0.00 C

ATOM 2617 ND1 HIS 169 31.000 36.830 75.910 0.00 0.00 N

ATOM 2618 CE1 HIS 169 32.170 36.840 75.290 0.00 0.00 C

ATOM 2619 HE1 HIS 169 32.350 36.390 74.330 0.00 0.00 H

ATOM 2620 NE2 HIS 169 33.060 37.650 75.940 0.00 0.00 N

ATOM 2621 HE2 HIS 169 34.000 37.880 75.650 0.00 0.00 H

ATOM 2622 CD2 HIS 169 32.400 38.150 77.050 0.00 0.00 C

ATOM 2623 HD2 HIS 169 32.790 38.880 77.750 0.00 0.00 H

ATOM 2624 C HIS 169 28.860 35.560 77.950 0.00 0.00 C

ATOM 2625 O HIS 169 27.680 35.720 77.760 0.00 0.00 O

ATOM 2626 N GLY 170 29.550 34.460 77.540 0.00 0.00 N

ATOM 2627 H GLY 170 30.540 34.460 77.770 0.00 0.00 H

ATOM 2628 CA GLY 170 28.910 33.390 76.810 0.00 0.00 C

ATOM 2629 HA1 GLY 170 27.850 33.370 77.070 0.00 0.00 H

ATOM 2630 HA2 GLY 170 29.420 32.450 76.990 0.00 0.00 H

ATOM 2631 C GLY 170 28.800 33.480 75.230 0.00 0.00 C

ATOM 2632 O GLY 170 28.770 32.490 74.520 0.00 0.00 O

ATOM 2633 N TYR 171 28.930 34.680 74.670 0.00 0.00 N

ATOM 2634 H TYR 171 29.370 35.410 75.220 0.00 0.00 H

ATOM 2635 CA TYR 171 28.680 34.920 73.270 0.00 0.00 C

ATOM 2636 HA TYR 171 27.730 34.450 73.030 0.00 0.00 H

ATOM 2637 CB TYR 171 28.370 36.420 73.130 0.00 0.00 C

ATOM 2638 HB1 TYR 171 29.300 36.990 73.100 0.00 0.00 H

ATOM 2639 HB2 TYR 171 27.730 36.760 73.940 0.00 0.00 H

ATOM 2640 CG TYR 171 27.680 36.860 71.890 0.00 0.00 C

ATOM 2641 CD1 TYR 171 26.270 37.130 71.860 0.00 0.00 C

ATOM 2642 HD1 TYR 171 25.770 37.060 72.810 0.00 0.00 H

ATOM 2643 CE1 TYR 171 25.570 37.290 70.590 0.00 0.00 C

ATOM 2644 HE1 TYR 171 24.490 37.410 70.570 0.00 0.00 H

ATOM 2645 CZ TYR 171 26.400 37.410 69.450 0.00 0.00 C

ATOM 2646 OH TYR 171 25.950 37.960 68.310 0.00 0.00 O

ATOM 2647 HH TYR 171 26.640 37.950 67.630 0.00 0.00 H

ATOM 2648 CE2 TYR 171 27.800 37.160 69.440 0.00 0.00 C

ATOM 2649 HE2 TYR 171 28.340 37.280 68.520 0.00 0.00 H

ATOM 2650 CD2 TYR 171 28.410 36.880 70.700 0.00 0.00 C

ATOM 2651 HD2 TYR 171 29.440 36.590 70.810 0.00 0.00 H

ATOM 2652 C TYR 171 29.690 34.220 72.370 0.00 0.00 C

ATOM 2653 O TYR 171 29.610 34.270 71.170 0.00 0.00 O

ATOM 2654 N THR 172 30.680 33.540 72.960 0.00 0.00 N

ATOM 2655 H THR 172 30.600 33.310 73.950 0.00 0.00 H

ATOM 2656 CA THR 172 31.960 33.220 72.250 0.00 0.00 C

ATOM 2657 HA THR 172 32.420 34.150 71.920 0.00 0.00 H

ATOM 2658 CB THR 172 32.930 32.590 73.230 0.00 0.00 C

ATOM 2659 HB THR 172 33.900 32.480 72.750 0.00 0.00 H

ATOM 2660 CG2 THR 172 33.240 33.470 74.340 0.00 0.00 C

ATOM 2661 HG21 THR 172 32.630 33.200 75.210 0.00 0.00 H

ATOM 2662 HG22 THR 172 34.300 33.340 74.550 0.00 0.00 H

ATOM 2663 HG23 THR 172 33.230 34.520 74.050 0.00 0.00 H

ATOM 2664 OG1 THR 172 32.460 31.420 73.730 0.00 0.00 O

ATOM 2665 HG1 THR 172 32.980 31.150 74.490 0.00 0.00 H

ATOM 2666 C THR 172 31.860 32.390 71.010 0.00 0.00 C

ATOM 2667 O THR 172 32.440 32.650 69.960 0.00 0.00 O

ATOM 2668 N ILE 173 30.930 31.430 71.050 0.00 0.00 N

ATOM 2669 H ILE 173 30.750 31.090 71.980 0.00 0.00 H

ATOM 2670 CA ILE 173 30.690 30.530 69.930 0.00 0.00 C

ATOM 2671 HA ILE 173 31.660 30.360 69.460 0.00 0.00 H

ATOM 2672 CB ILE 173 30.260 29.160 70.480 0.00 0.00 C

ATOM 2673 HB ILE 173 30.170 28.510 69.610 0.00 0.00 H

ATOM 2674 CG2 ILE 173 31.230 28.540 71.530 0.00 0.00 C

ATOM 2675 HG21 ILE 173 32.270 28.730 71.260 0.00 0.00 H

ATOM 2676 HG22 ILE 173 31.030 28.980 72.500 0.00 0.00 H

ATOM 2677 HG23 ILE 173 31.140 27.450 71.560 0.00 0.00 H

ATOM 2678 CG1 ILE 173 28.780 29.200 71.140 0.00 0.00 C

ATOM 2679 HG11 ILE 173 28.090 29.650 70.420 0.00 0.00 H

ATOM 2680 HG12 ILE 173 28.910 29.840 72.010 0.00 0.00 H

ATOM 2681 CD ILE 173 28.190 27.850 71.460 0.00 0.00 C

ATOM 2682 HD1 ILE 173 28.870 27.370 72.160 0.00 0.00 H

ATOM 2683 HD2 ILE 173 27.310 28.020 72.080 0.00 0.00 H

ATOM 2684 HD3 ILE 173 28.080 27.290 70.530 0.00 0.00 H

ATOM 2685 C ILE 173 29.720 31.090 68.950 0.00 0.00 C

ATOM 2686 O ILE 173 29.580 30.520 67.880 0.00 0.00 O

ATOM 2687 N TYR 174 28.960 32.180 69.200 0.00 0.00 N

ATOM 2688 H TYR 174 29.140 32.610 70.090 0.00 0.00 H

ATOM 2689 CA TYR 174 28.130 32.820 68.220 0.00 0.00 C

ATOM 2690 HA TYR 174 27.860 32.040 67.500 0.00 0.00 H

ATOM 2691 CB TYR 174 26.870 33.190 68.870 0.00 0.00 C

ATOM 2692 HB1 TYR 174 26.140 33.380 68.090 0.00 0.00 H

ATOM 2693 HB2 TYR 174 27.150 34.060 69.460 0.00 0.00 H

ATOM 2694 CG TYR 174 26.330 32.060 69.800 0.00 0.00 C

ATOM 2695 CD1 TYR 174 25.220 31.230 69.430 0.00 0.00 C

ATOM 2696 HD1 TYR 174 24.840 31.270 68.420 0.00 0.00 H

ATOM 2697 CE1 TYR 174 24.670 30.350 70.330 0.00 0.00 C

ATOM 2698 HE1 TYR 174 23.790 29.790 70.060 0.00 0.00 H

ATOM 2699 CZ TYR 174 25.160 30.380 71.670 0.00 0.00 C

ATOM 2700 OH TYR 174 24.530 29.640 72.600 0.00 0.00 O

ATOM 2701 HH TYR 174 24.840 29.830 73.490 0.00 0.00 H

ATOM 2702 CE2 TYR 174 26.310 31.150 72.080 0.00 0.00 C

ATOM 2703 HE2 TYR 174 26.660 31.050 73.090 0.00 0.00 H

ATOM 2704 CD2 TYR 174 26.800 32.010 71.120 0.00 0.00 C

ATOM 2705 HD2 TYR 174 27.580 32.680 71.460 0.00 0.00 H

ATOM 2706 C TYR 174 28.870 33.950 67.460 0.00 0.00 C

ATOM 2707 O TYR 174 28.560 34.220 66.300 0.00 0.00 O

ATOM 2708 N SER 175 29.840 34.670 68.090 0.00 0.00 N

ATOM 2709 H SER 175 29.980 34.460 69.070 0.00 0.00 H

ATOM 2710 CA SER 175 30.910 35.460 67.400 0.00 0.00 C

ATOM 2711 HA SER 175 30.510 36.120 66.640 0.00 0.00 H

ATOM 2712 CB SER 175 31.490 36.380 68.480 0.00 0.00 C

ATOM 2713 HB1 SER 175 30.780 37.100 68.890 0.00 0.00 H

ATOM 2714 HB2 SER 175 32.440 36.810 68.140 0.00 0.00 H

ATOM 2715 OG SER 175 31.850 35.580 69.620 0.00 0.00 O

ATOM 2716 HG SER 175 31.750 36.090 70.430 0.00 0.00 H

ATOM 2717 C SER 175 31.890 34.590 66.620 0.00 0.00 C

ATOM 2718 O SER 175 32.270 34.940 65.450 0.00 0.00 O

ATOM 2719 N THR 176 32.230 33.430 67.100 0.00 0.00 N

ATOM 2720 H THR 176 31.940 33.180 68.030 0.00 0.00 H

ATOM 2721 CA THR 176 32.980 32.430 66.310 0.00 0.00 C

ATOM 2722 HA THR 176 33.960 32.800 66.000 0.00 0.00 H

ATOM 2723 CB THR 176 33.360 31.230 67.220 0.00 0.00 C

ATOM 2724 HB THR 176 32.460 30.900 67.730 0.00 0.00 H

ATOM 2725 CG2 THR 176 34.080 30.050 66.470 0.00 0.00 C

ATOM 2726 HG21 THR 176 34.450 29.310 67.180 0.00 0.00 H

ATOM 2727 HG22 THR 176 33.290 29.670 65.820 0.00 0.00 H

ATOM 2728 HG23 THR 176 34.860 30.400 65.800 0.00 0.00 H

ATOM 2729 OG1 THR 176 34.270 31.660 68.180 0.00 0.00 O

ATOM 2730 HG1 THR 176 33.880 31.570 69.050 0.00 0.00 H

ATOM 2731 C THR 176 32.200 31.950 65.060 0.00 0.00 C

ATOM 2732 O THR 176 32.700 31.980 63.890 0.00 0.00 O

ATOM 2733 N PHE 177 30.930 31.600 65.230 0.00 0.00 N

ATOM 2734 H PHE 177 30.520 31.620 66.160 0.00 0.00 H

ATOM 2735 CA PHE 177 30.070 31.280 64.120 0.00 0.00 C

ATOM 2736 HA PHE 177 30.360 30.300 63.730 0.00 0.00 H

ATOM 2737 CB PHE 177 28.600 30.990 64.680 0.00 0.00 C

ATOM 2738 HB1 PHE 177 28.150 31.860 65.160 0.00 0.00 H

ATOM 2739 HB2 PHE 177 28.640 30.150 65.370 0.00 0.00 H

ATOM 2740 CG PHE 177 27.570 30.520 63.630 0.00 0.00 C

ATOM 2741 CD1 PHE 177 26.230 31.010 63.680 0.00 0.00 C

ATOM 2742 HD1 PHE 177 26.030 31.790 64.400 0.00 0.00 H

ATOM 2743 CE1 PHE 177 25.240 30.540 62.840 0.00 0.00 C

ATOM 2744 HE1 PHE 177 24.290 31.060 62.850 0.00 0.00 H

ATOM 2745 CZ PHE 177 25.550 29.510 61.920 0.00 0.00 C

ATOM 2746 HZ PHE 177 24.810 29.060 61.280 0.00 0.00 H

ATOM 2747 CE2 PHE 177 26.850 29.060 61.790 0.00 0.00 C

ATOM 2748 HE2 PHE 177 27.220 28.330 61.090 0.00 0.00 H

ATOM 2749 CD2 PHE 177 27.850 29.550 62.600 0.00 0.00 C

ATOM 2750 HD2 PHE 177 28.860 29.160 62.570 0.00 0.00 H

ATOM 2751 C PHE 177 29.950 32.390 63.030 0.00 0.00 C

ATOM 2752 O PHE 177 30.000 32.020 61.850 0.00 0.00 O

ATOM 2753 N GLY 178 29.990 33.680 63.410 0.00 0.00 N

ATOM 2754 H GLY 178 30.030 33.970 64.380 0.00 0.00 H

ATOM 2755 CA GLY 178 30.140 34.800 62.480 0.00 0.00 C

ATOM 2756 HA1 GLY 178 29.950 35.770 62.930 0.00 0.00 H

ATOM 2757 HA2 GLY 178 29.360 34.650 61.730 0.00 0.00 H

ATOM 2758 C GLY 178 31.520 35.060 61.920 0.00 0.00 C

ATOM 2759 O GLY 178 31.560 35.530 60.820 0.00 0.00 O

ATOM 2760 N ALA 179 32.640 34.880 62.660 0.00 0.00 N

ATOM 2761 H ALA 179 32.510 34.770 63.660 0.00 0.00 H

ATOM 2762 CA ALA 179 34.020 35.250 62.180 0.00 0.00 C

ATOM 2763 HA ALA 179 34.140 36.320 62.020 0.00 0.00 H

ATOM 2764 CB ALA 179 35.080 34.750 63.240 0.00 0.00 C

ATOM 2765 HB1 ALA 179 34.700 33.890 63.800 0.00 0.00 H

ATOM 2766 HB2 ALA 179 36.020 34.450 62.770 0.00 0.00 H

ATOM 2767 HB3 ALA 179 35.180 35.480 64.040 0.00 0.00 H

ATOM 2768 C ALA 179 34.410 34.580 60.870 0.00 0.00 C

ATOM 2769 O ALA 179 35.270 34.980 60.160 0.00 0.00 O

ATOM 2770 N PHE 180 33.920 33.400 60.730 0.00 0.00 N

ATOM 2771 H PHE 180 33.190 33.140 61.390 0.00 0.00 H

ATOM 2772 CA PHE 180 34.420 32.420 59.770 0.00 0.00 C

ATOM 2773 HA PHE 180 34.800 32.950 58.900 0.00 0.00 H

ATOM 2774 CB PHE 180 35.550 31.590 60.410 0.00 0.00 C

ATOM 2775 HB1 PHE 180 35.110 30.870 61.100 0.00 0.00 H

ATOM 2776 HB2 PHE 180 36.250 32.190 60.990 0.00 0.00 H

ATOM 2777 CG PHE 180 36.370 30.740 59.460 0.00 0.00 C

ATOM 2778 CD1 PHE 180 36.250 29.310 59.500 0.00 0.00 C

ATOM 2779 HD1 PHE 180 35.670 28.810 60.270 0.00 0.00 H

ATOM 2780 CE1 PHE 180 37.080 28.540 58.620 0.00 0.00 C

ATOM 2781 HE1 PHE 180 36.910 27.470 58.640 0.00 0.00 H

ATOM 2782 CZ PHE 180 37.950 29.180 57.730 0.00 0.00 C

ATOM 2783 HZ PHE 180 38.620 28.650 57.060 0.00 0.00 H

ATOM 2784 CE2 PHE 180 37.930 30.570 57.670 0.00 0.00 C

ATOM 2785 HE2 PHE 180 38.500 31.170 56.980 0.00 0.00 H

ATOM 2786 CD2 PHE 180 37.190 31.320 58.540 0.00 0.00 C

ATOM 2787 HD2 PHE 180 37.200 32.400 58.410 0.00 0.00 H

ATOM 2788 C PHE 180 33.290 31.550 59.240 0.00 0.00 C

ATOM 2789 O PHE 180 33.200 31.320 58.050 0.00 0.00 O

ATOM 2790 N TYR 181 32.270 31.120 60.000 0.00 0.00 N

ATOM 2791 H TYR 181 32.320 31.330 60.990 0.00 0.00 H

ATOM 2792 CA TYR 181 31.500 29.950 59.590 0.00 0.00 C

ATOM 2793 HA TYR 181 32.140 29.270 59.040 0.00 0.00 H

ATOM 2794 CB TYR 181 31.010 29.130 60.720 0.00 0.00 C

ATOM 2795 HB1 TYR 181 30.230 28.430 60.410 0.00 0.00 H

ATOM 2796 HB2 TYR 181 30.590 29.770 61.500 0.00 0.00 H

ATOM 2797 CG TYR 181 32.050 28.200 61.330 0.00 0.00 C

ATOM 2798 CD1 TYR 181 32.210 26.960 60.700 0.00 0.00 C

ATOM 2799 HD1 TYR 181 31.640 26.680 59.830 0.00 0.00 H

ATOM 2800 CE1 TYR 181 33.130 26.000 61.170 0.00 0.00 C

ATOM 2801 HE1 TYR 181 33.170 24.980 60.830 0.00 0.00 H

ATOM 2802 CZ TYR 181 33.810 26.270 62.380 0.00 0.00 C

ATOM 2803 OH TYR 181 34.680 25.340 62.800 0.00 0.00 O

ATOM 2804 HH TYR 181 35.190 25.630 63.560 0.00 0.00 H

ATOM 2805 CE2 TYR 181 33.630 27.510 62.990 0.00 0.00 C

ATOM 2806 HE2 TYR 181 34.130 27.680 63.930 0.00 0.00 H

ATOM 2807 CD2 TYR 181 32.810 28.540 62.460 0.00 0.00 C

ATOM 2808 HD2 TYR 181 32.640 29.470 62.980 0.00 0.00 H

ATOM 2809 C TYR 181 30.360 30.390 58.680 0.00 0.00 C

ATOM 2810 O TYR 181 30.230 29.930 57.540 0.00 0.00 O

ATOM 2811 N ILE 182 29.640 31.420 59.170 0.00 0.00 N

ATOM 2812 H ILE 182 29.850 31.740 60.100 0.00 0.00 H

ATOM 2813 CA ILE 182 28.650 32.160 58.300 0.00 0.00 C

ATOM 2814 HA ILE 182 27.900 31.420 58.010 0.00 0.00 H

ATOM 2815 CB ILE 182 27.820 33.230 59.020 0.00 0.00 C

ATOM 2816 HB ILE 182 28.530 33.940 59.450 0.00 0.00 H

ATOM 2817 CG2 ILE 182 27.010 34.050 57.970 0.00 0.00 C

ATOM 2818 HG21 ILE 182 27.640 34.570 57.240 0.00 0.00 H

ATOM 2819 HG22 ILE 182 26.520 33.190 57.510 0.00 0.00 H

ATOM 2820 HG23 ILE 182 26.280 34.710 58.440 0.00 0.00 H

ATOM 2821 CG1 ILE 182 26.900 32.530 60.060 0.00 0.00 C

ATOM 2822 HG11 ILE 182 27.550 31.810 60.580 0.00 0.00 H

ATOM 2823 HG12 ILE 182 26.060 32.050 59.560 0.00 0.00 H

ATOM 2824 CD ILE 182 26.320 33.490 61.160 0.00 0.00 C

ATOM 2825 HD1 ILE 182 25.890 34.390 60.720 0.00 0.00 H

ATOM 2826 HD2 ILE 182 25.470 32.960 61.600 0.00 0.00 H

ATOM 2827 HD3 ILE 182 27.070 33.660 61.930 0.00 0.00 H

ATOM 2828 C ILE 182 29.350 32.520 56.890 0.00 0.00 C

ATOM 2829 O ILE 182 28.830 32.070 55.920 0.00 0.00 O

ATOM 2830 N PRO 183 30.500 33.270 56.840 0.00 0.00 N

ATOM 2831 CD PRO 183 31.040 34.060 57.910 0.00 0.00 C

ATOM 2832 HD1 PRO 183 31.590 33.440 58.620 0.00 0.00 H

ATOM 2833 HD2 PRO 183 30.260 34.660 58.380 0.00 0.00 H

ATOM 2834 CG PRO 183 32.000 35.050 57.260 0.00 0.00 C

ATOM 2835 HG1 PRO 183 32.780 35.180 58.020 0.00 0.00 H

ATOM 2836 HG2 PRO 183 31.500 36.000 57.100 0.00 0.00 H

ATOM 2837 CB PRO 183 32.440 34.400 56.010 0.00 0.00 C

ATOM 2838 HB1 PRO 183 33.240 33.690 56.240 0.00 0.00 H

ATOM 2839 HB2 PRO 183 32.760 35.110 55.250 0.00 0.00 H

ATOM 2840 CA PRO 183 31.220 33.540 55.620 0.00 0.00 C

ATOM 2841 HA PRO 183 30.650 34.170 54.940 0.00 0.00 H

ATOM 2842 C PRO 183 31.560 32.300 54.790 0.00 0.00 C

ATOM 2843 O PRO 183 31.410 32.370 53.610 0.00 0.00 O

ATOM 2844 N LEU 184 32.010 31.150 55.410 0.00 0.00 N

ATOM 2845 H LEU 184 32.220 31.290 56.390 0.00 0.00 H

ATOM 2846 CA LEU 184 32.340 29.850 54.750 0.00 0.00 C

ATOM 2847 HA LEU 184 33.120 29.980 54.000 0.00 0.00 H

ATOM 2848 CB LEU 184 32.990 28.980 55.800 0.00 0.00 C

ATOM 2849 HB1 LEU 184 32.300 28.610 56.560 0.00 0.00 H

ATOM 2850 HB2 LEU 184 33.690 29.570 56.390 0.00 0.00 H

ATOM 2851 CG LEU 184 33.740 27.660 55.390 0.00 0.00 C

ATOM 2852 HG LEU 184 32.980 27.040 54.910 0.00 0.00 H

ATOM 2853 CD1 LEU 184 34.900 27.980 54.420 0.00 0.00 C

ATOM 2854 HD11 LEU 184 34.500 28.360 53.480 0.00 0.00 H

ATOM 2855 HD12 LEU 184 35.500 28.740 54.930 0.00 0.00 H

ATOM 2856 HD13 LEU 184 35.430 27.050 54.260 0.00 0.00 H

ATOM 2857 CD2 LEU 184 34.280 26.980 56.670 0.00 0.00 C

ATOM 2858 HD21 LEU 184 34.470 27.790 57.380 0.00 0.00 H

ATOM 2859 HD22 LEU 184 33.500 26.340 57.090 0.00 0.00 H

ATOM 2860 HD23 LEU 184 35.180 26.390 56.510 0.00 0.00 H

ATOM 2861 C LEU 184 31.080 29.180 54.060 0.00 0.00 C

ATOM 2862 O LEU 184 31.280 28.790 52.910 0.00 0.00 O

ATOM 2863 N LEU 185 29.860 29.180 54.730 0.00 0.00 N

ATOM 2864 H LEU 185 29.870 29.510 55.680 0.00 0.00 H

ATOM 2865 CA LEU 185 28.630 28.680 54.200 0.00 0.00 C

ATOM 2866 HA LEU 185 28.800 27.670 53.800 0.00 0.00 H

ATOM 2867 CB LEU 185 27.660 28.510 55.400 0.00 0.00 C

ATOM 2868 HB1 LEU 185 27.340 29.440 55.860 0.00 0.00 H

ATOM 2869 HB2 LEU 185 28.200 27.970 56.190 0.00 0.00 H

ATOM 2870 CG LEU 185 26.390 27.670 55.070 0.00 0.00 C

ATOM 2871 HG LEU 185 25.860 28.180 54.260 0.00 0.00 H

ATOM 2872 CD1 LEU 185 26.740 26.240 54.650 0.00 0.00 C

ATOM 2873 HD11 LEU 185 27.460 25.720 55.280 0.00 0.00 H

ATOM 2874 HD12 LEU 185 25.840 25.660 54.440 0.00 0.00 H

ATOM 2875 HD13 LEU 185 27.190 26.350 53.670 0.00 0.00 H

ATOM 2876 CD2 LEU 185 25.410 27.800 56.240 0.00 0.00 C

ATOM 2877 HD21 LEU 185 25.870 27.360 57.120 0.00 0.00 H

ATOM 2878 HD22 LEU 185 25.190 28.860 56.360 0.00 0.00 H

ATOM 2879 HD23 LEU 185 24.440 27.340 56.030 0.00 0.00 H

ATOM 2880 C LEU 185 28.210 29.460 52.970 0.00 0.00 C

ATOM 2881 O LEU 185 28.000 28.850 51.910 0.00 0.00 O

ATOM 2882 N LEU 186 28.110 30.790 53.110 0.00 0.00 N

ATOM 2883 H LEU 186 28.440 31.190 53.980 0.00 0.00 H

ATOM 2884 CA LEU 186 27.780 31.550 51.940 0.00 0.00 C

ATOM 2885 HA LEU 186 26.750 31.380 51.620 0.00 0.00 H

ATOM 2886 CB LEU 186 27.780 33.050 52.400 0.00 0.00 C

ATOM 2887 HB1 LEU 186 28.720 33.210 52.920 0.00 0.00 H

ATOM 2888 HB2 LEU 186 27.090 33.110 53.240 0.00 0.00 H

ATOM 2889 CG LEU 186 27.550 34.150 51.310 0.00 0.00 C

ATOM 2890 HG LEU 186 28.260 34.000 50.500 0.00 0.00 H

ATOM 2891 CD1 LEU 186 26.150 33.930 50.640 0.00 0.00 C

ATOM 2892 HD11 LEU 186 25.370 33.800 51.390 0.00 0.00 H

ATOM 2893 HD12 LEU 186 25.980 34.700 49.890 0.00 0.00 H

ATOM 2894 HD13 LEU 186 26.050 33.040 50.020 0.00 0.00 H

ATOM 2895 CD2 LEU 186 27.520 35.640 51.850 0.00 0.00 C

ATOM 2896 HD21 LEU 186 26.700 35.910 52.500 0.00 0.00 H

ATOM 2897 HD22 LEU 186 28.380 35.860 52.480 0.00 0.00 H

ATOM 2898 HD23 LEU 186 27.590 36.370 51.040 0.00 0.00 H

ATOM 2899 C LEU 186 28.720 31.280 50.720 0.00 0.00 C

ATOM 2900 O LEU 186 28.380 30.850 49.640 0.00 0.00 O

ATOM 2901 N MET 187 30.060 31.490 50.870 0.00 0.00 N

ATOM 2902 H MET 187 30.350 31.980 51.700 0.00 0.00 H

ATOM 2903 CA MET 187 31.060 31.190 49.840 0.00 0.00 C

ATOM 2904 HA MET 187 30.960 31.850 48.980 0.00 0.00 H

ATOM 2905 CB MET 187 32.510 31.340 50.410 0.00 0.00 C

ATOM 2906 HB1 MET 187 32.530 30.690 51.280 0.00 0.00 H

ATOM 2907 HB2 MET 187 32.620 32.390 50.690 0.00 0.00 H

ATOM 2908 CG MET 187 33.550 30.930 49.410 0.00 0.00 C

ATOM 2909 HG1 MET 187 33.520 31.420 48.430 0.00 0.00 H

ATOM 2910 HG2 MET 187 33.430 29.850 49.410 0.00 0.00 H

ATOM 2911 SD MET 187 35.310 31.200 49.860 0.00 0.00 S

ATOM 2912 CE MET 187 35.260 30.620 51.590 0.00 0.00 C

ATOM 2913 HE1 MET 187 34.990 31.380 52.330 0.00 0.00 H

ATOM 2914 HE2 MET 187 34.460 29.890 51.650 0.00 0.00 H

ATOM 2915 HE3 MET 187 36.240 30.200 51.830 0.00 0.00 H

ATOM 2916 C MET 187 30.950 29.720 49.280 0.00 0.00 C

ATOM 2917 O MET 187 31.170 29.550 48.080 0.00 0.00 O

ATOM 2918 N LEU 188 30.670 28.700 50.120 0.00 0.00 N

ATOM 2919 H LEU 188 30.530 28.890 51.100 0.00 0.00 H

ATOM 2920 CA LEU 188 30.680 27.280 49.670 0.00 0.00 C

ATOM 2921 HA LEU 188 31.550 27.070 49.040 0.00 0.00 H

ATOM 2922 CB LEU 188 30.800 26.350 50.910 0.00 0.00 C

ATOM 2923 HB1 LEU 188 30.000 26.530 51.630 0.00 0.00 H

ATOM 2924 HB2 LEU 188 31.750 26.490 51.420 0.00 0.00 H

ATOM 2925 CG LEU 188 30.760 24.890 50.660 0.00 0.00 C

ATOM 2926 HG LEU 188 29.800 24.670 50.200 0.00 0.00 H

ATOM 2927 CD1 LEU 188 31.800 24.470 49.580 0.00 0.00 C

ATOM 2928 HD11 LEU 188 31.570 24.840 48.580 0.00 0.00 H

ATOM 2929 HD12 LEU 188 32.820 24.750 49.850 0.00 0.00 H

ATOM 2930 HD13 LEU 188 31.830 23.390 49.510 0.00 0.00 H

ATOM 2931 CD2 LEU 188 31.030 24.120 51.920 0.00 0.00 C

ATOM 2932 HD21 LEU 188 32.090 24.280 52.140 0.00 0.00 H

ATOM 2933 HD22 LEU 188 30.360 24.350 52.750 0.00 0.00 H

ATOM 2934 HD23 LEU 188 31.030 23.090 51.550 0.00 0.00 H

ATOM 2935 C LEU 188 29.360 27.020 48.870 0.00 0.00 C

ATOM 2936 O LEU 188 29.440 26.340 47.890 0.00 0.00 O

ATOM 2937 N VAL 189 28.250 27.650 49.280 0.00 0.00 N

ATOM 2938 H VAL 189 28.220 28.270 50.090 0.00 0.00 H

ATOM 2939 CA VAL 189 26.950 27.500 48.550 0.00 0.00 C

ATOM 2940 HA VAL 189 26.820 26.410 48.600 0.00 0.00 H

ATOM 2941 CB VAL 189 25.650 27.960 49.280 0.00 0.00 C

ATOM 2942 HB VAL 189 25.700 29.030 49.490 0.00 0.00 H

ATOM 2943 CG1 VAL 189 24.330 27.710 48.470 0.00 0.00 C

ATOM 2944 HG11 VAL 189 24.420 28.280 47.550 0.00 0.00 H

ATOM 2945 HG12 VAL 189 24.300 26.650 48.220 0.00 0.00 H

ATOM 2946 HG13 VAL 189 23.450 28.060 49.020 0.00 0.00 H

ATOM 2947 CG2 VAL 189 25.370 27.120 50.610 0.00 0.00 C

ATOM 2948 HG21 VAL 189 24.460 27.370 51.140 0.00 0.00 H

ATOM 2949 HG22 VAL 189 25.290 26.070 50.320 0.00 0.00 H

ATOM 2950 HG23 VAL 189 26.280 27.190 51.220 0.00 0.00 H

ATOM 2951 C VAL 189 27.110 28.100 47.120 0.00 0.00 C

ATOM 2952 O VAL 189 26.750 27.510 46.120 0.00 0.00 O

ATOM 2953 N LEU 190 27.910 29.160 46.980 0.00 0.00 N

ATOM 2954 H LEU 190 28.480 29.550 47.710 0.00 0.00 H

ATOM 2955 CA LEU 190 28.280 29.700 45.660 0.00 0.00 C

ATOM 2956 HA LEU 190 27.350 29.850 45.110 0.00 0.00 H

ATOM 2957 CB LEU 190 28.990 31.000 45.900 0.00 0.00 C

ATOM 2958 HB1 LEU 190 29.300 31.350 44.910 0.00 0.00 H

ATOM 2959 HB2 LEU 190 29.860 30.770 46.520 0.00 0.00 H

ATOM 2960 CG LEU 190 28.030 32.040 46.560 0.00 0.00 C

ATOM 2961 HG LEU 190 27.470 31.570 47.370 0.00 0.00 H

ATOM 2962 CD1 LEU 190 29.010 33.080 47.130 0.00 0.00 C

ATOM 2963 HD11 LEU 190 28.510 33.990 47.460 0.00 0.00 H

ATOM 2964 HD12 LEU 190 29.460 32.490 47.930 0.00 0.00 H

ATOM 2965 HD13 LEU 190 29.830 33.250 46.420 0.00 0.00 H

ATOM 2966 CD2 LEU 190 27.040 32.710 45.490 0.00 0.00 C

ATOM 2967 HD21 LEU 190 26.190 32.050 45.290 0.00 0.00 H

ATOM 2968 HD22 LEU 190 26.680 33.620 45.970 0.00 0.00 H

ATOM 2969 HD23 LEU 190 27.570 32.930 44.570 0.00 0.00 H

ATOM 2970 C LEU 190 29.140 28.770 44.850 0.00 0.00 C

ATOM 2971 O LEU 190 28.930 28.590 43.620 0.00 0.00 O

ATOM 2972 N TYR 191 30.200 28.150 45.460 0.00 0.00 N

ATOM 2973 H TYR 191 30.320 28.460 46.410 0.00 0.00 H

ATOM 2974 CA TYR 191 31.160 27.210 44.850 0.00 0.00 C

ATOM 2975 HA TYR 191 31.680 27.640 43.990 0.00 0.00 H

ATOM 2976 CB TYR 191 32.300 27.120 45.790 0.00 0.00 C

ATOM 2977 HB1 TYR 191 31.890 26.730 46.720 0.00 0.00 H

ATOM 2978 HB2 TYR 191 32.620 28.140 45.960 0.00 0.00 H

ATOM 2979 CG TYR 191 33.580 26.380 45.280 0.00 0.00 C

ATOM 2980 CD1 TYR 191 33.740 25.050 45.760 0.00 0.00 C

ATOM 2981 HD1 TYR 191 33.070 24.560 46.450 0.00 0.00 H

ATOM 2982 CE1 TYR 191 34.840 24.310 45.230 0.00 0.00 C

ATOM 2983 HE1 TYR 191 34.920 23.260 45.480 0.00 0.00 H

ATOM 2984 CZ TYR 191 35.750 24.960 44.350 0.00 0.00 C

ATOM 2985 OH TYR 191 36.780 24.280 43.810 0.00 0.00 O

ATOM 2986 HH TYR 191 37.240 24.600 43.030 0.00 0.00 H

ATOM 2987 CE2 TYR 191 35.490 26.260 43.910 0.00 0.00 C

ATOM 2988 HE2 TYR 191 36.240 26.630 43.230 0.00 0.00 H

ATOM 2989 CD2 TYR 191 34.370 26.980 44.320 0.00 0.00 C

ATOM 2990 HD2 TYR 191 34.070 27.970 44.020 0.00 0.00 H

ATOM 2991 C TYR 191 30.630 25.810 44.420 0.00 0.00 C

ATOM 2992 O TYR 191 31.040 25.310 43.340 0.00 0.00 O

ATOM 2993 N GLY 192 29.690 25.290 45.160 0.00 0.00 N

ATOM 2994 H GLY 192 29.380 25.760 46.000 0.00 0.00 H

ATOM 2995 CA GLY 192 28.840 24.090 44.750 0.00 0.00 C

ATOM 2996 HA1 GLY 192 28.290 23.850 45.660 0.00 0.00 H

ATOM 2997 HA2 GLY 192 29.470 23.280 44.370 0.00 0.00 H

ATOM 2998 C GLY 192 27.890 24.490 43.510 0.00 0.00 C

ATOM 2999 O GLY 192 27.780 23.700 42.590 0.00 0.00 O

ATOM 3000 N ARG 193 27.240 25.720 43.610 0.00 0.00 N

ATOM 3001 H ARG 193 27.350 26.290 44.430 0.00 0.00 H

ATOM 3002 CA ARG 193 26.460 26.220 42.410 0.00 0.00 C

ATOM 3003 HA ARG 193 25.800 25.390 42.150 0.00 0.00 H

ATOM 3004 CB ARG 193 25.550 27.340 42.910 0.00 0.00 C

ATOM 3005 HB1 ARG 193 26.110 28.190 43.310 0.00 0.00 H

ATOM 3006 HB2 ARG 193 24.950 27.030 43.770 0.00 0.00 H

ATOM 3007 CG ARG 193 24.540 27.920 41.880 0.00 0.00 C

ATOM 3008 HG1 ARG 193 25.130 28.220 41.020 0.00 0.00 H

ATOM 3009 HG2 ARG 193 24.150 28.860 42.280 0.00 0.00 H

ATOM 3010 CD ARG 193 23.560 26.800 41.420 0.00 0.00 C

ATOM 3011 HD1 ARG 193 23.100 26.260 42.240 0.00 0.00 H

ATOM 3012 HD2 ARG 193 24.220 26.220 40.770 0.00 0.00 H

ATOM 3013 NE ARG 193 22.490 27.390 40.600 0.00 0.00 N

ATOM 3014 HE ARG 193 22.510 28.400 40.610 0.00 0.00 H

ATOM 3015 CZ ARG 193 21.410 26.780 40.100 0.00 0.00 C

ATOM 3016 NH1 ARG 193 21.100 25.520 40.130 0.00 0.00 N1+

ATOM 3017 HH11 ARG 193 21.690 24.820 40.560 0.00 0.00 H

ATOM 3018 HH12 ARG 193 20.210 25.360 39.690 0.00 0.00 H

ATOM 3019 NH2 ARG 193 20.580 27.480 39.440 0.00 0.00 N

ATOM 3020 HH21 ARG 193 20.670 28.490 39.350 0.00 0.00 H

ATOM 3021 HH22 ARG 193 19.770 27.090 38.980 0.00 0.00 H

ATOM 3022 C ARG 193 27.340 26.440 41.150 0.00 0.00 C

ATOM 3023 O ARG 193 26.910 26.030 40.090 0.00 0.00 O

ATOM 3024 N ILE 194 28.610 26.810 41.390 0.00 0.00 N

ATOM 3025 H ILE 194 28.820 27.060 42.350 0.00 0.00 H

ATOM 3026 CA ILE 194 29.690 26.810 40.360 0.00 0.00 C

ATOM 3027 HA ILE 194 29.260 27.280 39.480 0.00 0.00 H

ATOM 3028 CB ILE 194 30.910 27.650 40.760 0.00 0.00 C

ATOM 3029 HB ILE 194 31.150 27.430 41.810 0.00 0.00 H

ATOM 3030 CG2 ILE 194 32.200 27.350 39.950 0.00 0.00 C

ATOM 3031 HG21 ILE 194 32.500 26.310 39.850 0.00 0.00 H

ATOM 3032 HG22 ILE 194 32.170 27.870 38.990 0.00 0.00 H

ATOM 3033 HG23 ILE 194 33.010 27.880 40.450 0.00 0.00 H

ATOM 3034 CG1 ILE 194 30.590 29.200 40.820 0.00 0.00 C

ATOM 3035 HG11 ILE 194 29.570 29.390 41.150 0.00 0.00 H

ATOM 3036 HG12 ILE 194 30.730 29.650 39.840 0.00 0.00 H

ATOM 3037 CD ILE 194 31.410 29.970 41.870 0.00 0.00 C

ATOM 3038 HD1 ILE 194 31.120 31.020 41.900 0.00 0.00 H

ATOM 3039 HD2 ILE 194 31.170 29.520 42.830 0.00 0.00 H

ATOM 3040 HD3 ILE 194 32.480 29.880 41.720 0.00 0.00 H

ATOM 3041 C ILE 194 29.890 25.370 39.870 0.00 0.00 C

ATOM 3042 O ILE 194 29.960 25.190 38.670 0.00 0.00 O

ATOM 3043 N PHE 195 29.950 24.300 40.720 0.00 0.00 N

ATOM 3044 H PHE 195 29.970 24.520 41.700 0.00 0.00 H

ATOM 3045 CA PHE 195 30.030 22.850 40.330 0.00 0.00 C

ATOM 3046 HA PHE 195 30.950 22.670 39.770 0.00 0.00 H

ATOM 3047 CB PHE 195 30.140 21.900 41.540 0.00 0.00 C

ATOM 3048 HB1 PHE 195 29.200 21.900 42.090 0.00 0.00 H

ATOM 3049 HB2 PHE 195 30.880 22.310 42.230 0.00 0.00 H

ATOM 3050 CG PHE 195 30.540 20.460 41.180 0.00 0.00 C

ATOM 3051 CD1 PHE 195 31.820 20.120 40.680 0.00 0.00 C

ATOM 3052 HD1 PHE 195 32.610 20.850 40.620 0.00 0.00 H

ATOM 3053 CE1 PHE 195 32.200 18.740 40.410 0.00 0.00 C

ATOM 3054 HE1 PHE 195 33.160 18.480 40.000 0.00 0.00 H

ATOM 3055 CZ PHE 195 31.200 17.740 40.600 0.00 0.00 C

ATOM 3056 HZ PHE 195 31.410 16.690 40.440 0.00 0.00 H

ATOM 3057 CE2 PHE 195 29.990 18.040 41.160 0.00 0.00 C

ATOM 3058 HE2 PHE 195 29.420 17.160 41.430 0.00 0.00 H

ATOM 3059 CD2 PHE 195 29.630 19.370 41.270 0.00 0.00 C

ATOM 3060 HD2 PHE 195 28.640 19.570 41.640 0.00 0.00 H

ATOM 3061 C PHE 195 28.940 22.330 39.430 0.00 0.00 C

ATOM 3062 O PHE 195 29.230 21.700 38.430 0.00 0.00 O

ATOM 3063 N ARG 196 27.730 22.840 39.620 0.00 0.00 N

ATOM 3064 H ARG 196 27.700 23.500 40.380 0.00 0.00 H

ATOM 3065 CA ARG 196 26.630 22.680 38.700 0.00 0.00 C

ATOM 3066 HA ARG 196 26.460 21.600 38.690 0.00 0.00 H

ATOM 3067 CB ARG 196 25.300 23.330 39.140 0.00 0.00 C

ATOM 3068 HB1 ARG 196 25.320 24.410 39.240 0.00 0.00 H

ATOM 3069 HB2 ARG 196 25.130 22.870 40.110 0.00 0.00 H

ATOM 3070 CG ARG 196 24.010 22.980 38.410 0.00 0.00 C

ATOM 3071 HG1 ARG 196 24.180 23.330 37.390 0.00 0.00 H

ATOM 3072 HG2 ARG 196 23.210 23.520 38.910 0.00 0.00 H

ATOM 3073 CD ARG 196 23.770 21.400 38.460 0.00 0.00 C

ATOM 3074 HD1 ARG 196 23.710 21.000 39.470 0.00 0.00 H

ATOM 3075 HD2 ARG 196 24.600 20.960 37.910 0.00 0.00 H

ATOM 3076 NE ARG 196 22.500 21.170 37.810 0.00 0.00 N

ATOM 3077 HE ARG 196 22.130 21.840 37.150 0.00 0.00 H

ATOM 3078 CZ ARG 196 21.750 20.090 37.910 0.00 0.00 C

ATOM 3079 NH1 ARG 196 22.000 19.130 38.800 0.00 0.00 N1+

ATOM 3080 HH11 ARG 196 22.630 19.350 39.560 0.00 0.00 H

ATOM 3081 HH12 ARG 196 21.470 18.270 38.750 0.00 0.00 H

ATOM 3082 NH2 ARG 196 20.790 19.870 37.070 0.00 0.00 N

ATOM 3083 HH21 ARG 196 20.460 20.570 36.430 0.00 0.00 H

ATOM 3084 HH22 ARG 196 20.370 18.950 37.030 0.00 0.00 H

ATOM 3085 C ARG 196 26.910 23.220 37.290 0.00 0.00 C

ATOM 3086 O ARG 196 26.510 22.540 36.340 0.00 0.00 O

ATOM 3087 N ALA 197 27.660 24.280 37.090 0.00 0.00 N

ATOM 3088 H ALA 197 27.900 24.820 37.910 0.00 0.00 H

ATOM 3089 CA ALA 197 27.970 24.860 35.750 0.00 0.00 C

ATOM 3090 HA ALA 197 27.090 24.710 35.130 0.00 0.00 H

ATOM 3091 CB ALA 197 28.300 26.400 35.800 0.00 0.00 C

ATOM 3092 HB1 ALA 197 29.190 26.600 36.390 0.00 0.00 H

ATOM 3093 HB2 ALA 197 28.450 26.720 34.770 0.00 0.00 H

ATOM 3094 HB3 ALA 197 27.490 26.940 36.290 0.00 0.00 H

ATOM 3095 C ALA 197 29.190 24.160 35.080 0.00 0.00 C

ATOM 3096 O ALA 197 29.340 24.220 33.860 0.00 0.00 O

ATOM 3097 N ALA 198 29.970 23.440 35.870 0.00 0.00 N

ATOM 3098 H ALA 198 29.680 23.450 36.830 0.00 0.00 H

ATOM 3099 CA ALA 198 30.930 22.430 35.430 0.00 0.00 C

ATOM 3100 HA ALA 198 31.440 22.690 34.500 0.00 0.00 H

ATOM 3101 CB ALA 198 31.960 22.150 36.480 0.00 0.00 C

ATOM 3102 HB1 ALA 198 31.550 21.880 37.460 0.00 0.00 H

ATOM 3103 HB2 ALA 198 32.650 21.390 36.110 0.00 0.00 H

ATOM 3104 HB3 ALA 198 32.620 22.980 36.720 0.00 0.00 H

ATOM 3105 C ALA 198 30.260 21.060 34.890 0.00 0.00 C

ATOM 3106 O ALA 198 30.440 20.690 33.740 0.00 0.00 O

ATOM 3107 N ARG 199 29.400 20.420 35.730 0.00 0.00 N

ATOM 3108 H ARG 199 29.310 20.770 36.670 0.00 0.00 H

ATOM 3109 CA ARG 199 28.730 19.210 35.380 0.00 0.00 C

ATOM 3110 HA ARG 199 29.370 18.490 34.870 0.00 0.00 H

ATOM 3111 CB ARG 199 28.080 18.560 36.570 0.00 0.00 C

ATOM 3112 HB1 ARG 199 27.480 17.680 36.310 0.00 0.00 H

ATOM 3113 HB2 ARG 199 27.500 19.390 36.990 0.00 0.00 H

ATOM 3114 CG ARG 199 29.150 18.130 37.610 0.00 0.00 C

ATOM 3115 HG1 ARG 199 28.610 17.920 38.540 0.00 0.00 H

ATOM 3116 HG2 ARG 199 29.800 18.980 37.830 0.00 0.00 H

ATOM 3117 CD ARG 199 30.050 16.930 37.270 0.00 0.00 C

ATOM 3118 HD1 ARG 199 30.970 16.940 37.850 0.00 0.00 H

ATOM 3119 HD2 ARG 199 30.420 17.080 36.260 0.00 0.00 H

ATOM 3120 NE ARG 199 29.340 15.630 37.380 0.00 0.00 N

ATOM 3121 HE ARG 199 28.330 15.650 37.460 0.00 0.00 H

ATOM 3122 CZ ARG 199 29.860 14.430 37.400 0.00 0.00 C

ATOM 3123 NH1 ARG 199 31.170 14.390 37.610 0.00 0.00 N1+

ATOM 3124 HH11 ARG 199 31.630 15.290 37.630 0.00 0.00 H

ATOM 3125 HH12 ARG 199 31.510 13.530 38.010 0.00 0.00 H

ATOM 3126 NH2 ARG 199 29.140 13.340 37.500 0.00 0.00 N

ATOM 3127 HH21 ARG 199 28.140 13.470 37.430 0.00 0.00 H

ATOM 3128 HH22 ARG 199 29.480 12.390 37.530 0.00 0.00 H

ATOM 3129 C ARG 199 27.660 19.450 34.330 0.00 0.00 C

ATOM 3130 O ARG 199 27.490 18.570 33.430 0.00 0.00 O

ATOM 3131 N PHE 200 26.980 20.640 34.300 0.00 0.00 N

ATOM 3132 H PHE 200 27.360 21.370 34.880 0.00 0.00 H

ATOM 3133 CA PHE 200 25.880 20.910 33.510 0.00 0.00 C

ATOM 3134 HA PHE 200 25.930 20.380 32.550 0.00 0.00 H

ATOM 3135 CB PHE 200 24.660 20.460 34.360 0.00 0.00 C

ATOM 3136 HB1 PHE 200 24.560 21.020 35.280 0.00 0.00 H

ATOM 3137 HB2 PHE 200 24.790 19.390 34.540 0.00 0.00 H

ATOM 3138 CG PHE 200 23.400 20.520 33.490 0.00 0.00 C

ATOM 3139 CD1 PHE 200 23.290 19.770 32.330 0.00 0.00 C

ATOM 3140 HD1 PHE 200 24.180 19.190 32.120 0.00 0.00 H

ATOM 3141 CE1 PHE 200 22.210 19.980 31.470 0.00 0.00 C

ATOM 3142 HE1 PHE 200 22.110 19.610 30.460 0.00 0.00 H

ATOM 3143 CZ PHE 200 21.130 20.750 31.890 0.00 0.00 C

ATOM 3144 HZ PHE 200 20.310 20.820 31.190 0.00 0.00 H

ATOM 3145 CE2 PHE 200 21.210 21.500 33.070 0.00 0.00 C

ATOM 3146 HE2 PHE 200 20.510 22.260 33.380 0.00 0.00 H

ATOM 3147 CD2 PHE 200 22.370 21.380 33.840 0.00 0.00 C

ATOM 3148 HD2 PHE 200 22.420 22.100 34.640 0.00 0.00 H

ATOM 3149 C PHE 200 25.940 22.400 33.140 0.00 0.00 C

ATOM 3150 O PHE 200 26.880 22.780 32.410 0.00 0.00 O

ATOM 3151 N ARG 201 24.980 23.300 33.510 0.00 0.00 N

ATOM 3152 H ARG 201 24.250 22.870 34.060 0.00 0.00 H

ATOM 3153 CA ARG 201 24.800 24.710 33.040 0.00 0.00 C

ATOM 3154 HA ARG 201 25.730 25.260 33.170 0.00 0.00 H

ATOM 3155 CB ARG 201 24.410 24.600 31.540 0.00 0.00 C

ATOM 3156 HB1 ARG 201 25.190 24.100 30.970 0.00 0.00 H

ATOM 3157 HB2 ARG 201 24.330 25.650 31.250 0.00 0.00 H

ATOM 3158 CG ARG 201 23.120 23.830 31.270 0.00 0.00 C

ATOM 3159 HG1 ARG 201 22.280 24.250 31.820 0.00 0.00 H

ATOM 3160 HG2 ARG 201 23.200 22.810 31.660 0.00 0.00 H

ATOM 3161 CD ARG 201 22.940 23.960 29.820 0.00 0.00 C

ATOM 3162 HD1 ARG 201 23.840 23.630 29.310 0.00 0.00 H

ATOM 3163 HD2 ARG 201 22.780 24.990 29.510 0.00 0.00 H

ATOM 3164 NE ARG 201 21.880 23.120 29.300 0.00 0.00 N

ATOM 3165 HE ARG 201 20.960 23.530 29.320 0.00 0.00 H

ATOM 3166 CZ ARG 201 21.940 21.930 28.700 0.00 0.00 C

ATOM 3167 NH1 ARG 201 23.080 21.250 28.650 0.00 0.00 N1+

ATOM 3168 HH11 ARG 201 23.900 21.730 28.990 0.00 0.00 H

ATOM 3169 HH12 ARG 201 23.140 20.370 28.170 0.00 0.00 H

ATOM 3170 NH2 ARG 201 20.760 21.450 28.290 0.00 0.00 N

ATOM 3171 HH21 ARG 201 19.860 21.880 28.410 0.00 0.00 H

ATOM 3172 HH22 ARG 201 20.680 20.470 28.060 0.00 0.00 H

ATOM 3173 C ARG 201 23.700 25.450 33.870 0.00 0.00 C

ATOM 3174 O ARG 201 22.710 24.790 34.330 0.00 0.00 O

ATOM 3175 N ILE 202 23.960 26.710 34.140 0.00 0.00 N

ATOM 3176 H ILE 202 24.820 27.080 33.760 0.00 0.00 H

ATOM 3177 CA ILE 202 22.990 27.590 35.000 0.00 0.00 C

ATOM 3178 HA ILE 202 22.030 27.080 35.040 0.00 0.00 H

ATOM 3179 CB ILE 202 23.510 27.610 36.480 0.00 0.00 C

ATOM 3180 HB ILE 202 22.720 28.010 37.100 0.00 0.00 H

ATOM 3181 CG2 ILE 202 23.590 26.250 37.200 0.00 0.00 C

ATOM 3182 HG21 ILE 202 23.880 26.200 38.250 0.00 0.00 H

ATOM 3183 HG22 ILE 202 22.740 25.630 36.930 0.00 0.00 H

ATOM 3184 HG23 ILE 202 24.430 25.820 36.650 0.00 0.00 H

ATOM 3185 CG1 ILE 202 24.680 28.650 36.610 0.00 0.00 C

ATOM 3186 HG11 ILE 202 24.220 29.630 36.490 0.00 0.00 H

ATOM 3187 HG12 ILE 202 25.310 28.430 35.740 0.00 0.00 H

ATOM 3188 CD ILE 202 25.460 28.610 37.950 0.00 0.00 C

ATOM 3189 HD1 ILE 202 24.770 28.720 38.800 0.00 0.00 H

ATOM 3190 HD2 ILE 202 26.000 27.690 38.150 0.00 0.00 H

ATOM 3191 HD3 ILE 202 26.180 29.430 37.960 0.00 0.00 H

ATOM 3192 C ILE 202 22.650 28.970 34.410 0.00 0.00 C

ATOM 3193 O ILE 202 23.050 29.180 33.280 0.00 0.00 O

TER

ATOM 3194 N NME 202 21.910 29.900 35.100 0.00 0.00 N

ATOM 3195 H NME 202 21.530 29.730 36.020 0.00 0.00 H

ATOM 3196 CH3 NME 202 21.450 31.150 34.490 0.00 0.00 C

ATOM 3197 HH31 NME 202 22.310 31.820 34.380 0.00 0.00 H

ATOM 3198 HH32 NME 202 21.020 30.900 33.520 0.00 0.00 H

ATOM 3199 HH33 NME 202 20.730 31.570 35.190 0.00 0.00 H

ATOM 3200 HH31 ACE 203 25.410 34.550 30.320 0.00 0.00 H

ATOM 3201 CH3 ACE 203 26.380 34.280 30.750 0.00 0.00 C

ATOM 3202 HH32 ACE 203 26.640 33.270 30.440 0.00 0.00 H

ATOM 3203 HH33 ACE 203 26.330 34.360 31.840 0.00 0.00 H

ATOM 3204 C ACE 203 27.450 35.230 30.250 0.00 0.00 C

ATOM 3205 O ACE 203 27.560 35.320 29.020 0.00 0.00 O

ATOM 3206 N LEU 203 28.280 35.790 31.120 0.00 0.00 N

ATOM 3207 H LEU 203 27.990 35.690 32.080 0.00 0.00 H

ATOM 3208 CA LEU 203 29.520 36.390 30.740 0.00 0.00 C

ATOM 3209 HA LEU 203 29.520 36.680 29.690 0.00 0.00 H

ATOM 3210 CB LEU 203 29.830 37.660 31.640 0.00 0.00 C

ATOM 3211 HB1 LEU 203 29.490 37.550 32.670 0.00 0.00 H

ATOM 3212 HB2 LEU 203 29.380 38.520 31.150 0.00 0.00 H

ATOM 3213 CG LEU 203 31.270 38.030 31.810 0.00 0.00 C

ATOM 3214 HG LEU 203 31.700 37.120 32.230 0.00 0.00 H

ATOM 3215 CD1 LEU 203 32.000 38.300 30.450 0.00 0.00 C

ATOM 3216 HD11 LEU 203 32.620 39.190 30.380 0.00 0.00 H

ATOM 3217 HD12 LEU 203 32.640 37.450 30.200 0.00 0.00 H

ATOM 3218 HD13 LEU 203 31.190 38.370 29.730 0.00 0.00 H

ATOM 3219 CD2 LEU 203 31.470 39.300 32.680 0.00 0.00 C

ATOM 3220 HD21 LEU 203 31.160 39.020 33.690 0.00 0.00 H

ATOM 3221 HD22 LEU 203 32.520 39.580 32.590 0.00 0.00 H

ATOM 3222 HD23 LEU 203 30.790 40.060 32.300 0.00 0.00 H

ATOM 3223 C LEU 203 30.650 35.320 30.690 0.00 0.00 C

ATOM 3224 O LEU 203 31.410 35.190 29.700 0.00 0.00 O

ATOM 3225 N ALA 204 30.820 34.480 31.690 0.00 0.00 N

ATOM 3226 H ALA 204 30.260 34.500 32.540 0.00 0.00 H

ATOM 3227 CA ALA 204 31.740 33.380 31.690 0.00 0.00 C

ATOM 3228 HA ALA 204 32.530 33.490 30.940 0.00 0.00 H

ATOM 3229 CB ALA 204 32.460 33.250 33.100 0.00 0.00 C

ATOM 3230 HB1 ALA 204 31.650 33.060 33.810 0.00 0.00 H

ATOM 3231 HB2 ALA 204 33.100 32.370 33.150 0.00 0.00 H

ATOM 3232 HB3 ALA 204 32.990 34.180 33.320 0.00 0.00 H

ATOM 3233 C ALA 204 31.060 32.020 31.420 0.00 0.00 C

ATOM 3234 O ALA 204 29.860 31.860 31.640 0.00 0.00 O

ATOM 3235 N ARG 205 31.940 31.120 30.980 0.00 0.00 N

ATOM 3236 H ARG 205 32.850 31.500 30.800 0.00 0.00 H

ATOM 3237 CA ARG 205 31.740 29.680 30.860 0.00 0.00 C

ATOM 3238 HA ARG 205 30.690 29.380 30.920 0.00 0.00 H

ATOM 3239 CB ARG 205 32.190 29.190 29.410 0.00 0.00 C

ATOM 3240 HB1 ARG 205 32.590 28.180 29.520 0.00 0.00 H

ATOM 3241 HB2 ARG 205 33.020 29.820 29.080 0.00 0.00 H

ATOM 3242 CG ARG 205 31.000 29.260 28.450 0.00 0.00 C

ATOM 3243 HG1 ARG 205 30.350 30.110 28.660 0.00 0.00 H

ATOM 3244 HG2 ARG 205 30.420 28.380 28.720 0.00 0.00 H

ATOM 3245 CD ARG 205 31.440 29.220 27.010 0.00 0.00 C

ATOM 3246 HD1 ARG 205 30.520 29.140 26.440 0.00 0.00 H

ATOM 3247 HD2 ARG 205 31.940 28.270 26.790 0.00 0.00 H

ATOM 3248 NE ARG 205 32.250 30.350 26.570 0.00 0.00 N

ATOM 3249 HE ARG 205 32.240 31.160 27.170 0.00 0.00 H

ATOM 3250 CZ ARG 205 32.600 30.530 25.310 0.00 0.00 C

ATOM 3251 NH1 ARG 205 32.410 29.600 24.370 0.00 0.00 N1+

ATOM 3252 HH11 ARG 205 31.890 28.750 24.540 0.00 0.00 H

ATOM 3253 HH12 ARG 205 32.680 29.880 23.440 0.00 0.00 H

ATOM 3254 NH2 ARG 205 33.110 31.660 24.890 0.00 0.00 N

ATOM 3255 HH21 ARG 205 33.260 32.480 25.460 0.00 0.00 H

ATOM 3256 HH22 ARG 205 33.360 31.700 23.910 0.00 0.00 H

ATOM 3257 C ARG 205 32.570 28.960 31.920 0.00 0.00 C

ATOM 3258 O ARG 205 33.580 29.500 32.440 0.00 0.00 O

ATOM 3259 N GLU 206 32.040 27.840 32.460 0.00 0.00 N

ATOM 3260 H GLU 206 31.220 27.400 32.060 0.00 0.00 H

ATOM 3261 CA GLU 206 32.580 26.990 33.490 0.00 0.00 C

ATOM 3262 HA GLU 206 33.620 27.210 33.700 0.00 0.00 H

ATOM 3263 CB GLU 206 31.730 27.120 34.740 0.00 0.00 C

ATOM 3264 HB1 GLU 206 30.770 26.700 34.460 0.00 0.00 H

ATOM 3265 HB2 GLU 206 31.640 28.170 35.030 0.00 0.00 H

ATOM 3266 CG GLU 206 32.280 26.240 35.870 0.00 0.00 C

ATOM 3267 HG1 GLU 206 32.130 25.230 35.490 0.00 0.00 H

ATOM 3268 HG2 GLU 206 31.740 26.420 36.800 0.00 0.00 H

ATOM 3269 CD GLU 206 33.780 26.450 36.170 0.00 0.00 C

ATOM 3270 OE1 GLU 206 34.680 25.600 35.960 0.00 0.00 O

ATOM 3271 OE2 GLU 206 34.030 27.570 36.670 0.00 0.00 O1-

ATOM 3272 C GLU 206 32.610 25.550 32.940 0.00 0.00 C

ATOM 3273 O GLU 206 31.750 25.190 32.120 0.00 0.00 O

ATOM 3274 N ARG 207 33.610 24.730 33.360 0.00 0.00 N

ATOM 3275 H ARG 207 34.220 25.160 34.040 0.00 0.00 H

ATOM 3276 CA ARG 207 33.750 23.300 32.990 0.00 0.00 C

ATOM 3277 HA ARG 207 32.750 22.940 33.250 0.00 0.00 H

ATOM 3278 CB ARG 207 33.900 23.080 31.500 0.00 0.00 C

ATOM 3279 HB1 ARG 207 34.760 23.640 31.140 0.00 0.00 H

ATOM 3280 HB2 ARG 207 32.960 23.370 31.040 0.00 0.00 H

ATOM 3281 CG ARG 207 34.010 21.570 31.090 0.00 0.00 C

ATOM 3282 HG1 ARG 207 34.950 21.230 31.530 0.00 0.00 H

ATOM 3283 HG2 ARG 207 34.210 21.570 30.010 0.00 0.00 H

ATOM 3284 CD ARG 207 32.890 20.660 31.630 0.00 0.00 C

ATOM 3285 HD1 ARG 207 31.920 20.950 31.240 0.00 0.00 H

ATOM 3286 HD2 ARG 207 32.810 20.770 32.710 0.00 0.00 H

ATOM 3287 NE ARG 207 33.180 19.250 31.380 0.00 0.00 N

ATOM 3288 HE ARG 207 34.110 19.000 31.050 0.00 0.00 H

ATOM 3289 CZ ARG 207 32.420 18.210 31.650 0.00 0.00 C

ATOM 3290 NH1 ARG 207 31.310 18.270 32.330 0.00 0.00 N1+

ATOM 3291 HH11 ARG 207 31.020 19.170 32.680 0.00 0.00 H

ATOM 3292 HH12 ARG 207 30.830 17.450 32.670 0.00 0.00 H

ATOM 3293 NH2 ARG 207 32.850 17.030 31.320 0.00 0.00 N

ATOM 3294 HH21 ARG 207 33.820 16.990 31.070 0.00 0.00 H

ATOM 3295 HH22 ARG 207 32.450 16.160 31.660 0.00 0.00 H

ATOM 3296 C ARG 207 34.890 22.700 33.780 0.00 0.00 C

ATOM 3297 O ARG 207 34.630 21.660 34.320 0.00 0.00 O

ATOM 3298 N LYS 208 35.970 23.400 33.980 0.00 0.00 N

ATOM 3299 H LYS 208 36.060 24.200 33.370 0.00 0.00 H

ATOM 3300 CA LYS 208 37.100 22.940 34.850 0.00 0.00 C

ATOM 3301 HA LYS 208 36.720 22.150 35.510 0.00 0.00 H

ATOM 3302 CB LYS 208 38.250 22.290 34.030 0.00 0.00 C

ATOM 3303 HB1 LYS 208 37.950 21.340 33.600 0.00 0.00 H

ATOM 3304 HB2 LYS 208 39.050 22.090 34.750 0.00 0.00 H

ATOM 3305 CG LYS 208 38.800 23.290 33.030 0.00 0.00 C

ATOM 3306 HG1 LYS 208 39.370 24.050 33.560 0.00 0.00 H

ATOM 3307 HG2 LYS 208 37.990 23.680 32.410 0.00 0.00 H

ATOM 3308 CD LYS 208 39.680 22.470 32.020 0.00 0.00 C

ATOM 3309 HD1 LYS 208 38.960 22.040 31.320 0.00 0.00 H

ATOM 3310 HD2 LYS 208 40.140 21.600 32.480 0.00 0.00 H

ATOM 3311 CE LYS 208 40.640 23.390 31.270 0.00 0.00 C

ATOM 3312 HE1 LYS 208 41.390 23.810 31.940 0.00 0.00 H

ATOM 3313 HE2 LYS 208 40.100 24.190 30.780 0.00 0.00 H

ATOM 3314 NZ LYS 208 41.370 22.660 30.180 0.00 0.00 N1+

ATOM 3315 HZ1 LYS 208 41.740 21.760 30.450 0.00 0.00 H

ATOM 3316 HZ2 LYS 208 40.680 22.460 29.460 0.00 0.00 H

ATOM 3317 HZ3 LYS 208 42.180 23.180 29.900 0.00 0.00 H

ATOM 3318 C LYS 208 37.640 24.100 35.720 0.00 0.00 C

ATOM 3319 O LYS 208 38.340 23.750 36.710 0.00 0.00 O

ATOM 3320 N THR 209 37.320 25.390 35.580 0.00 0.00 N

ATOM 3321 H THR 209 36.670 25.610 34.840 0.00 0.00 H

ATOM 3322 CA THR 209 37.790 26.520 36.460 0.00 0.00 C

ATOM 3323 HA THR 209 38.880 26.510 36.440 0.00 0.00 H

ATOM 3324 CB THR 209 37.370 27.900 35.930 0.00 0.00 C

ATOM 3325 HB THR 209 36.300 27.950 36.130 0.00 0.00 H

ATOM 3326 CG2 THR 209 38.140 28.970 36.650 0.00 0.00 C

ATOM 3327 HG21 THR 209 37.800 29.220 37.650 0.00 0.00 H

ATOM 3328 HG22 THR 209 39.210 28.740 36.670 0.00 0.00 H

ATOM 3329 HG23 THR 209 37.980 29.880 36.070 0.00 0.00 H

ATOM 3330 OG1 THR 209 37.670 27.940 34.610 0.00 0.00 O

ATOM 3331 HG1 THR 209 38.620 27.850 34.520 0.00 0.00 H

ATOM 3332 C THR 209 37.380 26.330 37.870 0.00 0.00 C

ATOM 3333 O THR 209 38.070 26.780 38.740 0.00 0.00 O

ATOM 3334 N VAL 210 36.340 25.610 38.230 0.00 0.00 N

ATOM 3335 H VAL 210 35.710 25.520 37.450 0.00 0.00 H

ATOM 3336 CA VAL 210 35.980 25.210 39.640 0.00 0.00 C

ATOM 3337 HA VAL 210 35.690 26.140 40.120 0.00 0.00 H

ATOM 3338 CB VAL 210 34.710 24.380 39.720 0.00 0.00 C

ATOM 3339 HB VAL 210 33.940 24.980 39.230 0.00 0.00 H

ATOM 3340 CG1 VAL 210 34.790 23.080 38.820 0.00 0.00 C

ATOM 3341 HG11 VAL 210 34.930 23.360 37.770 0.00 0.00 H

ATOM 3342 HG12 VAL 210 35.670 22.550 39.210 0.00 0.00 H

ATOM 3343 HG13 VAL 210 33.970 22.370 38.950 0.00 0.00 H

ATOM 3344 CG2 VAL 210 34.230 24.130 41.180 0.00 0.00 C

ATOM 3345 HG21 VAL 210 34.180 25.050 41.770 0.00 0.00 H

ATOM 3346 HG22 VAL 210 33.270 23.620 41.120 0.00 0.00 H

ATOM 3347 HG23 VAL 210 34.970 23.480 41.640 0.00 0.00 H

ATOM 3348 C VAL 210 37.200 24.610 40.290 0.00 0.00 C

ATOM 3349 O VAL 210 37.590 24.940 41.380 0.00 0.00 O

ATOM 3350 N LYS 211 37.830 23.660 39.610 0.00 0.00 N

ATOM 3351 H LYS 211 37.500 23.530 38.670 0.00 0.00 H

ATOM 3352 CA LYS 211 39.000 22.990 40.120 0.00 0.00 C

ATOM 3353 HA LYS 211 38.780 22.650 41.130 0.00 0.00 H

ATOM 3354 CB LYS 211 39.070 21.730 39.220 0.00 0.00 C

ATOM 3355 HB1 LYS 211 39.560 22.160 38.340 0.00 0.00 H

ATOM 3356 HB2 LYS 211 38.170 21.180 38.950 0.00 0.00 H

ATOM 3357 CG LYS 211 40.100 20.690 39.740 0.00 0.00 C

ATOM 3358 HG1 LYS 211 39.980 20.350 40.770 0.00 0.00 H

ATOM 3359 HG2 LYS 211 40.960 21.350 39.680 0.00 0.00 H

ATOM 3360 CD LYS 211 40.320 19.520 38.750 0.00 0.00 C

ATOM 3361 HD1 LYS 211 39.470 18.830 38.820 0.00 0.00 H

ATOM 3362 HD2 LYS 211 41.200 18.980 39.100 0.00 0.00 H

ATOM 3363 CE LYS 211 40.320 19.790 37.250 0.00 0.00 C

ATOM 3364 HE1 LYS 211 41.100 20.500 36.960 0.00 0.00 H

ATOM 3365 HE2 LYS 211 39.350 20.230 37.030 0.00 0.00 H

ATOM 3366 NZ LYS 211 40.530 18.510 36.450 0.00 0.00 N1+

ATOM 3367 HZ1 LYS 211 41.320 17.950 36.730 0.00 0.00 H

ATOM 3368 HZ2 LYS 211 39.710 17.930 36.430 0.00 0.00 H

ATOM 3369 HZ3 LYS 211 40.830 18.780 35.530 0.00 0.00 H

ATOM 3370 C LYS 211 40.220 23.960 40.200 0.00 0.00 C

ATOM 3371 O LYS 211 40.940 23.950 41.180 0.00 0.00 O

ATOM 3372 N THR 212 40.420 24.860 39.260 0.00 0.00 N

ATOM 3373 H THR 212 39.860 24.840 38.410 0.00 0.00 H

ATOM 3374 CA THR 212 41.460 25.920 39.330 0.00 0.00 C

ATOM 3375 HA THR 212 42.430 25.470 39.560 0.00 0.00 H

ATOM 3376 CB THR 212 41.530 26.620 38.040 0.00 0.00 C

ATOM 3377 HB THR 212 40.720 27.350 37.970 0.00 0.00 H

ATOM 3378 CG2 THR 212 42.850 27.380 38.020 0.00 0.00 C

ATOM 3379 HG21 THR 212 42.850 28.130 38.810 0.00 0.00 H

ATOM 3380 HG22 THR 212 43.680 26.700 38.140 0.00 0.00 H

ATOM 3381 HG23 THR 212 43.040 27.800 37.030 0.00 0.00 H

ATOM 3382 OG1 THR 212 41.470 25.690 36.970 0.00 0.00 O

ATOM 3383 HG1 THR 212 42.200 25.070 37.100 0.00 0.00 H

ATOM 3384 C THR 212 41.130 26.880 40.540 0.00 0.00 C

ATOM 3385 O THR 212 42.100 27.090 41.290 0.00 0.00 O

ATOM 3386 N LEU 213 39.990 27.450 40.720 0.00 0.00 N

ATOM 3387 H LEU 213 39.170 27.190 40.190 0.00 0.00 H

ATOM 3388 CA LEU 213 39.660 28.400 41.810 0.00 0.00 C

ATOM 3389 HA LEU 213 40.320 29.270 41.740 0.00 0.00 H

ATOM 3390 CB LEU 213 38.200 28.780 41.590 0.00 0.00 C

ATOM 3391 HB1 LEU 213 37.670 27.840 41.440 0.00 0.00 H

ATOM 3392 HB2 LEU 213 38.050 29.280 40.630 0.00 0.00 H

ATOM 3393 CG LEU 213 37.640 29.750 42.630 0.00 0.00 C

ATOM 3394 HG LEU 213 37.680 29.270 43.610 0.00 0.00 H

ATOM 3395 CD1 LEU 213 38.300 31.140 42.590 0.00 0.00 C

ATOM 3396 HD11 LEU 213 39.390 31.080 42.530 0.00 0.00 H

ATOM 3397 HD12 LEU 213 37.820 31.610 41.730 0.00 0.00 H

ATOM 3398 HD13 LEU 213 37.930 31.710 43.440 0.00 0.00 H

ATOM 3399 CD2 LEU 213 36.150 29.600 42.370 0.00 0.00 C

ATOM 3400 HD21 LEU 213 35.910 30.170 41.470 0.00 0.00 H

ATOM 3401 HD22 LEU 213 35.730 28.610 42.200 0.00 0.00 H

ATOM 3402 HD23 LEU 213 35.570 30.080 43.160 0.00 0.00 H

ATOM 3403 C LEU 213 39.920 27.790 43.230 0.00 0.00 C

ATOM 3404 O LEU 213 40.530 28.540 44.130 0.00 0.00 O

ATOM 3405 N GLY 214 39.640 26.490 43.440 0.00 0.00 N

ATOM 3406 H GLY 214 39.060 26.070 42.720 0.00 0.00 H

ATOM 3407 CA GLY 214 39.850 25.690 44.640 0.00 0.00 C

ATOM 3408 HA1 GLY 214 39.420 24.710 44.410 0.00 0.00 H

ATOM 3409 HA2 GLY 214 39.350 26.240 45.430 0.00 0.00 H

ATOM 3410 C GLY 214 41.300 25.450 44.980 0.00 0.00 C

ATOM 3411 O GLY 214 41.620 25.100 46.070 0.00 0.00 O

ATOM 3412 N ILE 215 42.310 25.700 44.120 0.00 0.00 N

ATOM 3413 H ILE 215 42.010 25.840 43.160 0.00 0.00 H

ATOM 3414 CA ILE 215 43.760 25.890 44.530 0.00 0.00 C

ATOM 3415 HA ILE 215 44.080 24.980 45.030 0.00 0.00 H

ATOM 3416 CB ILE 215 44.800 26.160 43.390 0.00 0.00 C

ATOM 3417 HB ILE 215 44.440 27.070 42.920 0.00 0.00 H

ATOM 3418 CG2 ILE 215 46.260 26.360 43.890 0.00 0.00 C

ATOM 3419 HG21 ILE 215 46.940 26.720 43.120 0.00 0.00 H

ATOM 3420 HG22 ILE 215 46.370 27.130 44.650 0.00 0.00 H

ATOM 3421 HG23 ILE 215 46.600 25.380 44.240 0.00 0.00 H

ATOM 3422 CG1 ILE 215 44.820 25.050 42.210 0.00 0.00 C

ATOM 3423 HG11 ILE 215 43.800 24.760 41.980 0.00 0.00 H

ATOM 3424 HG12 ILE 215 45.220 24.070 42.480 0.00 0.00 H

ATOM 3425 CD ILE 215 45.510 25.560 40.950 0.00 0.00 C

ATOM 3426 HD1 ILE 215 46.580 25.370 41.030 0.00 0.00 H

ATOM 3427 HD2 ILE 215 45.220 24.960 40.090 0.00 0.00 H

ATOM 3428 HD3 ILE 215 45.360 26.630 40.820 0.00 0.00 H

ATOM 3429 C ILE 215 43.860 26.960 45.610 0.00 0.00 C

ATOM 3430 O ILE 215 44.340 26.620 46.710 0.00 0.00 O

ATOM 3431 N ILE 216 43.630 28.240 45.290 0.00 0.00 N

ATOM 3432 H ILE 216 43.310 28.480 44.360 0.00 0.00 H

ATOM 3433 CA ILE 216 43.790 29.390 46.210 0.00 0.00 C

ATOM 3434 HA ILE 216 44.760 29.240 46.690 0.00 0.00 H

ATOM 3435 CB ILE 216 43.700 30.680 45.450 0.00 0.00 C

ATOM 3436 HB ILE 216 43.640 31.530 46.130 0.00 0.00 H

ATOM 3437 CG2 ILE 216 45.000 30.820 44.650 0.00 0.00 C

ATOM 3438 HG21 ILE 216 44.970 31.640 43.940 0.00 0.00 H

ATOM 3439 HG22 ILE 216 45.840 30.950 45.340 0.00 0.00 H

ATOM 3440 HG23 ILE 216 45.120 29.850 44.170 0.00 0.00 H

ATOM 3441 CG1 ILE 216 42.420 30.870 44.590 0.00 0.00 C

ATOM 3442 HG11 ILE 216 41.520 30.700 45.170 0.00 0.00 H

ATOM 3443 HG12 ILE 216 42.400 30.350 43.630 0.00 0.00 H

ATOM 3444 CD ILE 216 42.360 32.410 44.240 0.00 0.00 C

ATOM 3445 HD1 ILE 216 43.240 32.820 43.730 0.00 0.00 H

ATOM 3446 HD2 ILE 216 41.540 32.510 43.540 0.00 0.00 H

ATOM 3447 HD3 ILE 216 42.200 33.070 45.100 0.00 0.00 H

ATOM 3448 C ILE 216 42.710 29.370 47.310 0.00 0.00 C

ATOM 3449 O ILE 216 43.050 29.520 48.470 0.00 0.00 O

ATOM 3450 N MET 217 41.450 29.060 46.940 0.00 0.00 N

ATOM 3451 H MET 217 41.310 29.110 45.940 0.00 0.00 H

ATOM 3452 CA MET 217 40.300 29.090 47.850 0.00 0.00 C

ATOM 3453 HA MET 217 40.330 30.050 48.370 0.00 0.00 H

ATOM 3454 CB MET 217 38.940 28.920 47.130 0.00 0.00 C

ATOM 3455 HB1 MET 217 38.230 28.570 47.870 0.00 0.00 H

ATOM 3456 HB2 MET 217 38.910 28.360 46.200 0.00 0.00 H

ATOM 3457 CG MET 217 38.420 30.350 46.770 0.00 0.00 C

ATOM 3458 HG1 MET 217 39.120 30.700 46.010 0.00 0.00 H

ATOM 3459 HG2 MET 217 38.590 31.070 47.570 0.00 0.00 H

ATOM 3460 SD MET 217 36.760 30.440 46.250 0.00 0.00 S

ATOM 3461 CE MET 217 36.460 32.200 46.120 0.00 0.00 C

ATOM 3462 HE1 MET 217 36.750 32.670 45.170 0.00 0.00 H

ATOM 3463 HE2 MET 217 35.370 32.210 46.230 0.00 0.00 H

ATOM 3464 HE3 MET 217 36.910 32.760 46.940 0.00 0.00 H

ATOM 3465 C MET 217 40.520 27.990 48.910 0.00 0.00 C

ATOM 3466 O MET 217 40.360 28.260 50.100 0.00 0.00 O

ATOM 3467 N GLY 218 40.920 26.840 48.410 0.00 0.00 N

ATOM 3468 H GLY 218 41.020 26.750 47.410 0.00 0.00 H

ATOM 3469 CA GLY 218 41.370 25.680 49.160 0.00 0.00 C

ATOM 3470 HA1 GLY 218 41.630 24.830 48.530 0.00 0.00 H

ATOM 3471 HA2 GLY 218 40.550 25.430 49.830 0.00 0.00 H

ATOM 3472 C GLY 218 42.550 25.950 50.080 0.00 0.00 C

ATOM 3473 O GLY 218 42.210 25.760 51.220 0.00 0.00 O

ATOM 3474 N THR 219 43.690 26.490 49.590 0.00 0.00 N

ATOM 3475 H THR 219 43.830 26.690 48.610 0.00 0.00 H

ATOM 3476 CA THR 219 44.850 26.870 50.460 0.00 0.00 C

ATOM 3477 HA THR 219 45.390 26.010 50.850 0.00 0.00 H

ATOM 3478 CB THR 219 45.910 27.700 49.710 0.00 0.00 C

ATOM 3479 HB THR 219 45.480 28.640 49.350 0.00 0.00 H

ATOM 3480 CG2 THR 219 47.240 28.110 50.430 0.00 0.00 C

ATOM 3481 HG21 THR 219 47.770 28.730 49.710 0.00 0.00 H

ATOM 3482 HG22 THR 219 47.140 28.700 51.340 0.00 0.00 H

ATOM 3483 HG23 THR 219 47.790 27.250 50.810 0.00 0.00 H

ATOM 3484 OG1 THR 219 46.290 26.930 48.610 0.00 0.00 O

ATOM 3485 HG1 THR 219 45.600 26.970 47.940 0.00 0.00 H

ATOM 3486 C THR 219 44.490 27.770 51.620 0.00 0.00 C

ATOM 3487 O THR 219 44.960 27.580 52.700 0.00 0.00 O

ATOM 3488 N PHE 220 43.650 28.770 51.330 0.00 0.00 N

ATOM 3489 H PHE 220 43.360 28.890 50.370 0.00 0.00 H

ATOM 3490 CA PHE 220 43.080 29.660 52.310 0.00 0.00 C

ATOM 3491 HA PHE 220 43.920 30.120 52.840 0.00 0.00 H

ATOM 3492 CB PHE 220 42.220 30.710 51.530 0.00 0.00 C

ATOM 3493 HB1 PHE 220 41.410 30.210 51.010 0.00 0.00 H

ATOM 3494 HB2 PHE 220 42.850 31.090 50.730 0.00 0.00 H

ATOM 3495 CG PHE 220 41.710 31.830 52.400 0.00 0.00 C

ATOM 3496 CD1 PHE 220 40.540 31.650 53.200 0.00 0.00 C

ATOM 3497 HD1 PHE 220 40.000 30.730 53.280 0.00 0.00 H

ATOM 3498 CE1 PHE 220 39.990 32.710 53.940 0.00 0.00 C

ATOM 3499 HE1 PHE 220 39.030 32.570 54.420 0.00 0.00 H

ATOM 3500 CZ PHE 220 40.650 33.920 53.990 0.00 0.00 C

ATOM 3501 HZ PHE 220 40.180 34.760 54.460 0.00 0.00 H

ATOM 3502 CE2 PHE 220 41.870 34.080 53.270 0.00 0.00 C

ATOM 3503 HE2 PHE 220 42.460 34.990 53.300 0.00 0.00 H

ATOM 3504 CD2 PHE 220 42.280 33.150 52.310 0.00 0.00 C

ATOM 3505 HD2 PHE 220 43.130 33.380 51.680 0.00 0.00 H

ATOM 3506 C PHE 220 42.200 28.880 53.270 0.00 0.00 C

ATOM 3507 O PHE 220 42.360 29.090 54.470 0.00 0.00 O

ATOM 3508 N ILE 221 41.240 28.030 52.890 0.00 0.00 N

ATOM 3509 H ILE 221 41.150 27.730 51.930 0.00 0.00 H

ATOM 3510 CA ILE 221 40.370 27.350 53.890 0.00 0.00 C

ATOM 3511 HA ILE 221 40.180 28.080 54.670 0.00 0.00 H

ATOM 3512 CB ILE 221 39.020 26.840 53.240 0.00 0.00 C

ATOM 3513 HB ILE 221 39.360 26.280 52.370 0.00 0.00 H

ATOM 3514 CG2 ILE 221 38.150 26.050 54.200 0.00 0.00 C

ATOM 3515 HG21 ILE 221 37.940 26.640 55.090 0.00 0.00 H

ATOM 3516 HG22 ILE 221 37.190 25.900 53.710 0.00 0.00 H

ATOM 3517 HG23 ILE 221 38.640 25.100 54.410 0.00 0.00 H

ATOM 3518 CG1 ILE 221 38.250 28.070 52.600 0.00 0.00 C

ATOM 3519 HG11 ILE 221 38.970 28.800 52.220 0.00 0.00 H

ATOM 3520 HG12 ILE 221 37.850 28.620 53.450 0.00 0.00 H

ATOM 3521 CD ILE 221 37.190 27.710 51.520 0.00 0.00 C

ATOM 3522 HD1 ILE 221 37.090 28.480 50.760 0.00 0.00 H

ATOM 3523 HD2 ILE 221 37.410 26.760 51.040 0.00 0.00 H

ATOM 3524 HD3 ILE 221 36.230 27.650 52.040 0.00 0.00 H

ATOM 3525 C ILE 221 41.170 26.320 54.620 0.00 0.00 C

ATOM 3526 O ILE 221 40.850 26.100 55.780 0.00 0.00 O

ATOM 3527 N LEU 222 42.200 25.720 54.000 0.00 0.00 N

ATOM 3528 H LEU 222 42.300 25.900 53.010 0.00 0.00 H

ATOM 3529 CA LEU 222 43.070 24.760 54.680 0.00 0.00 C

ATOM 3530 HA LEU 222 42.440 24.030 55.180 0.00 0.00 H

ATOM 3531 CB LEU 222 43.970 24.140 53.560 0.00 0.00 C

ATOM 3532 HB1 LEU 222 44.430 24.980 53.050 0.00 0.00 H

ATOM 3533 HB2 LEU 222 43.330 23.650 52.820 0.00 0.00 H

ATOM 3534 CG LEU 222 44.960 23.110 54.060 0.00 0.00 C

ATOM 3535 HG LEU 222 45.710 23.620 54.670 0.00 0.00 H

ATOM 3536 CD1 LEU 222 44.430 21.920 54.820 0.00 0.00 C

ATOM 3537 HD11 LEU 222 43.700 21.320 54.270 0.00 0.00 H

ATOM 3538 HD12 LEU 222 45.250 21.250 55.100 0.00 0.00 H

ATOM 3539 HD13 LEU 222 43.820 22.260 55.660 0.00 0.00 H

ATOM 3540 CD2 LEU 222 45.660 22.670 52.730 0.00 0.00 C

ATOM 3541 HD21 LEU 222 46.410 21.890 52.850 0.00 0.00 H

ATOM 3542 HD22 LEU 222 44.920 22.320 52.000 0.00 0.00 H

ATOM 3543 HD23 LEU 222 46.140 23.510 52.230 0.00 0.00 H

ATOM 3544 C LEU 222 43.900 25.450 55.700 0.00 0.00 C

ATOM 3545 O LEU 222 44.140 24.760 56.690 0.00 0.00 O

ATOM 3546 N CYS 223 44.460 26.600 55.490 0.00 0.00 N

ATOM 3547 H CYS 223 44.480 27.160 54.650 0.00 0.00 H

ATOM 3548 CA CYS 223 45.150 27.310 56.640 0.00 0.00 C

ATOM 3549 HA CYS 223 45.780 26.560 57.130 0.00 0.00 H

ATOM 3550 CB CYS 223 46.090 28.430 56.130 0.00 0.00 C

ATOM 3551 HB1 CYS 223 46.580 28.900 56.980 0.00 0.00 H

ATOM 3552 HB2 CYS 223 45.600 29.160 55.500 0.00 0.00 H

ATOM 3553 SG CYS 223 47.290 27.700 55.010 0.00 0.00 S

ATOM 3554 HG CYS 223 46.370 27.330 54.110 0.00 0.00 H

ATOM 3555 C CYS 223 44.220 27.800 57.730 0.00 0.00 C

ATOM 3556 O CYS 223 44.580 27.760 58.900 0.00 0.00 O

ATOM 3557 N TRP 224 42.940 28.130 57.480 0.00 0.00 N

ATOM 3558 H TRP 224 42.650 28.180 56.510 0.00 0.00 H

ATOM 3559 CA TRP 224 42.120 28.830 58.430 0.00 0.00 C

ATOM 3560 HA TRP 224 42.710 29.370 59.180 0.00 0.00 H

ATOM 3561 CB TRP 224 41.570 30.090 57.690 0.00 0.00 C

ATOM 3562 HB1 TRP 224 40.720 30.410 58.290 0.00 0.00 H

ATOM 3563 HB2 TRP 224 41.210 29.870 56.690 0.00 0.00 H

ATOM 3564 CG TRP 224 42.490 31.230 57.580 0.00 0.00 C

ATOM 3565 CD1 TRP 224 43.040 31.740 56.470 0.00 0.00 C

ATOM 3566 HD1 TRP 224 42.790 31.500 55.450 0.00 0.00 H

ATOM 3567 NE1 TRP 224 43.670 32.910 56.800 0.00 0.00 N

ATOM 3568 HE1 TRP 224 44.230 33.360 56.090 0.00 0.00 H

ATOM 3569 CE2 TRP 224 43.740 33.010 58.130 0.00 0.00 C

ATOM 3570 CZ2 TRP 224 44.380 33.980 58.940 0.00 0.00 C

ATOM 3571 HZ2 TRP 224 45.010 34.730 58.480 0.00 0.00 H

ATOM 3572 CH2 TRP 224 44.260 33.890 60.350 0.00 0.00 C

ATOM 3573 HH2 TRP 224 44.610 34.650 61.030 0.00 0.00 H

ATOM 3574 CZ3 TRP 224 43.430 32.860 60.860 0.00 0.00 C

ATOM 3575 HZ3 TRP 224 43.230 32.760 61.920 0.00 0.00 H

ATOM 3576 CE3 TRP 224 42.890 31.820 60.090 0.00 0.00 C

ATOM 3577 HE3 TRP 224 42.220 31.090 60.510 0.00 0.00 H

ATOM 3578 CD2 TRP 224 42.980 31.960 58.700 0.00 0.00 C

ATOM 3579 C TRP 224 41.140 27.920 59.130 0.00 0.00 C

ATOM 3580 O TRP 224 40.800 28.210 60.220 0.00 0.00 O

ATOM 3581 N LEU 225 40.670 26.830 58.500 0.00 0.00 N

ATOM 3582 H LEU 225 41.160 26.610 57.650 0.00 0.00 H

ATOM 3583 CA LEU 225 39.620 25.990 59.170 0.00 0.00 C

ATOM 3584 HA LEU 225 38.870 26.700 59.520 0.00 0.00 H

ATOM 3585 CB LEU 225 38.850 25.170 58.090 0.00 0.00 C

ATOM 3586 HB1 LEU 225 39.660 24.680 57.550 0.00 0.00 H

ATOM 3587 HB2 LEU 225 38.350 25.830 57.390 0.00 0.00 H

ATOM 3588 CG LEU 225 37.800 24.110 58.590 0.00 0.00 C

ATOM 3589 HG LEU 225 38.390 23.440 59.220 0.00 0.00 H

ATOM 3590 CD1 LEU 225 36.590 24.800 59.360 0.00 0.00 C

ATOM 3591 HD11 LEU 225 36.870 25.590 60.060 0.00 0.00 H

ATOM 3592 HD12 LEU 225 35.750 25.120 58.740 0.00 0.00 H

ATOM 3593 HD13 LEU 225 36.180 24.060 60.050 0.00 0.00 H

ATOM 3594 CD2 LEU 225 37.210 23.370 57.370 0.00 0.00 C

ATOM 3595 HD21 LEU 225 38.070 22.980 56.820 0.00 0.00 H

ATOM 3596 HD22 LEU 225 36.680 22.470 57.650 0.00 0.00 H

ATOM 3597 HD23 LEU 225 36.570 24.000 56.750 0.00 0.00 H

ATOM 3598 C LEU 225 40.070 25.150 60.490 0.00 0.00 C

ATOM 3599 O LEU 225 39.320 25.230 61.460 0.00 0.00 O

ATOM 3600 N PRO 226 41.230 24.480 60.630 0.00 0.00 N

ATOM 3601 CD PRO 226 42.170 24.300 59.540 0.00 0.00 C

ATOM 3602 HD1 PRO 226 42.780 25.190 59.390 0.00 0.00 H

ATOM 3603 HD2 PRO 226 41.690 24.000 58.610 0.00 0.00 H

ATOM 3604 CG PRO 226 43.030 23.070 59.870 0.00 0.00 C

ATOM 3605 HG1 PRO 226 44.010 23.140 59.390 0.00 0.00 H

ATOM 3606 HG2 PRO 226 42.550 22.120 59.590 0.00 0.00 H

ATOM 3607 CB PRO 226 43.080 23.390 61.390 0.00 0.00 C

ATOM 3608 HB1 PRO 226 43.770 24.210 61.600 0.00 0.00 H

ATOM 3609 HB2 PRO 226 43.380 22.520 61.980 0.00 0.00 H

ATOM 3610 CA PRO 226 41.700 23.790 61.780 0.00 0.00 C

ATOM 3611 HA PRO 226 41.140 22.870 61.980 0.00 0.00 H

ATOM 3612 C PRO 226 41.640 24.640 63.070 0.00 0.00 C

ATOM 3613 O PRO 226 41.240 24.210 64.140 0.00 0.00 O

ATOM 3614 N PHE 227 42.010 25.930 62.890 0.00 0.00 N

ATOM 3615 H PHE 227 42.320 26.250 61.990 0.00 0.00 H

ATOM 3616 CA PHE 227 41.900 27.010 63.840 0.00 0.00 C

ATOM 3617 HA PHE 227 42.530 26.720 64.680 0.00 0.00 H

ATOM 3618 CB PHE 227 42.490 28.300 63.300 0.00 0.00 C

ATOM 3619 HB1 PHE 227 41.960 28.530 62.380 0.00 0.00 H

ATOM 3620 HB2 PHE 227 43.510 28.040 63.040 0.00 0.00 H

ATOM 3621 CG PHE 227 42.520 29.440 64.330 0.00 0.00 C

ATOM 3622 CD1 PHE 227 43.470 29.520 65.390 0.00 0.00 C

ATOM 3623 HD1 PHE 227 44.240 28.770 65.520 0.00 0.00 H

ATOM 3624 CE1 PHE 227 43.460 30.600 66.240 0.00 0.00 C

ATOM 3625 HE1 PHE 227 44.250 30.730 66.970 0.00 0.00 H

ATOM 3626 CZ PHE 227 42.460 31.590 66.190 0.00 0.00 C

ATOM 3627 HZ PHE 227 42.460 32.410 66.890 0.00 0.00 H

ATOM 3628 CE2 PHE 227 41.540 31.460 65.140 0.00 0.00 C

ATOM 3629 HE2 PHE 227 40.750 32.180 64.960 0.00 0.00 H

ATOM 3630 CD2 PHE 227 41.490 30.370 64.250 0.00 0.00 C

ATOM 3631 HD2 PHE 227 40.640 30.290 63.590 0.00 0.00 H

ATOM 3632 C PHE 227 40.480 27.250 64.360 0.00 0.00 C

ATOM 3633 O PHE 227 40.250 27.180 65.550 0.00 0.00 O

ATOM 3634 N PHE 228 39.490 27.470 63.480 0.00 0.00 N

ATOM 3635 H PHE 228 39.610 27.470 62.470 0.00 0.00 H

ATOM 3636 CA PHE 228 38.140 27.770 63.870 0.00 0.00 C

ATOM 3637 HA PHE 228 38.190 28.350 64.790 0.00 0.00 H

ATOM 3638 CB PHE 228 37.470 28.550 62.710 0.00 0.00 C

ATOM 3639 HB1 PHE 228 36.400 28.570 62.890 0.00 0.00 H

ATOM 3640 HB2 PHE 228 37.830 28.010 61.830 0.00 0.00 H

ATOM 3641 CG PHE 228 37.890 29.960 62.700 0.00 0.00 C

ATOM 3642 CD1 PHE 228 37.430 30.860 63.740 0.00 0.00 C

ATOM 3643 HD1 PHE 228 36.830 30.420 64.510 0.00 0.00 H

ATOM 3644 CE1 PHE 228 37.860 32.150 63.800 0.00 0.00 C

ATOM 3645 HE1 PHE 228 37.530 32.830 64.570 0.00 0.00 H

ATOM 3646 CZ PHE 228 38.800 32.680 62.800 0.00 0.00 C

ATOM 3647 HZ PHE 228 39.210 33.680 62.860 0.00 0.00 H

ATOM 3648 CE2 PHE 228 39.210 31.860 61.720 0.00 0.00 C

ATOM 3649 HE2 PHE 228 39.840 32.270 60.950 0.00 0.00 H

ATOM 3650 CD2 PHE 228 38.700 30.520 61.710 0.00 0.00 C

ATOM 3651 HD2 PHE 228 38.970 29.810 60.940 0.00 0.00 H

ATOM 3652 C PHE 228 37.400 26.500 64.350 0.00 0.00 C

ATOM 3653 O PHE 228 36.310 26.710 64.770 0.00 0.00 O

ATOM 3654 N ILE 229 37.900 25.340 64.200 0.00 0.00 N

ATOM 3655 H ILE 229 38.790 25.380 63.720 0.00 0.00 H

ATOM 3656 CA ILE 229 37.490 24.070 64.760 0.00 0.00 C

ATOM 3657 HA ILE 229 36.400 24.050 64.760 0.00 0.00 H

ATOM 3658 CB ILE 229 38.040 22.870 63.960 0.00 0.00 C

ATOM 3659 HB ILE 229 39.060 23.080 63.640 0.00 0.00 H

ATOM 3660 CG2 ILE 229 38.010 21.480 64.630 0.00 0.00 C

ATOM 3661 HG21 ILE 229 38.520 21.510 65.590 0.00 0.00 H

ATOM 3662 HG22 ILE 229 37.030 21.020 64.730 0.00 0.00 H

ATOM 3663 HG23 ILE 229 38.510 20.780 63.970 0.00 0.00 H

ATOM 3664 CG1 ILE 229 37.240 22.820 62.580 0.00 0.00 C

ATOM 3665 HG11 ILE 229 37.130 23.820 62.160 0.00 0.00 H

ATOM 3666 HG12 ILE 229 36.230 22.590 62.910 0.00 0.00 H

ATOM 3667 CD ILE 229 37.770 21.840 61.530 0.00 0.00 C

ATOM 3668 HD1 ILE 229 37.560 20.840 61.920 0.00 0.00 H

ATOM 3669 HD2 ILE 229 37.250 22.000 60.590 0.00 0.00 H

ATOM 3670 HD3 ILE 229 38.840 21.960 61.370 0.00 0.00 H

ATOM 3671 C ILE 229 37.930 24.050 66.250 0.00 0.00 C

ATOM 3672 O ILE 229 37.120 23.790 67.110 0.00 0.00 O

ATOM 3673 N VAL 230 39.210 24.360 66.630 0.00 0.00 N

ATOM 3674 H VAL 230 39.850 24.470 65.850 0.00 0.00 H

ATOM 3675 CA VAL 230 39.690 24.500 68.010 0.00 0.00 C

ATOM 3676 HA VAL 230 39.380 23.600 68.530 0.00 0.00 H

ATOM 3677 CB VAL 230 41.280 24.450 68.010 0.00 0.00 C

ATOM 3678 HB VAL 230 41.580 23.570 67.450 0.00 0.00 H

ATOM 3679 CG1 VAL 230 41.950 25.520 67.290 0.00 0.00 C

ATOM 3680 HG11 VAL 230 41.830 26.510 67.730 0.00 0.00 H

ATOM 3681 HG12 VAL 230 43.040 25.400 67.240 0.00 0.00 H

ATOM 3682 HG13 VAL 230 41.620 25.550 66.250 0.00 0.00 H

ATOM 3683 CG2 VAL 230 41.840 24.370 69.460 0.00 0.00 C

ATOM 3684 HG21 VAL 230 41.460 25.220 70.030 0.00 0.00 H

ATOM 3685 HG22 VAL 230 41.470 23.460 69.950 0.00 0.00 H

ATOM 3686 HG23 VAL 230 42.920 24.280 69.370 0.00 0.00 H

ATOM 3687 C VAL 230 38.950 25.690 68.710 0.00 0.00 C

ATOM 3688 O VAL 230 38.690 25.620 69.920 0.00 0.00 O

ATOM 3689 N ALA 231 38.470 26.740 67.960 0.00 0.00 N

ATOM 3690 H ALA 231 38.740 26.890 67.000 0.00 0.00 H

ATOM 3691 CA ALA 231 37.680 27.850 68.590 0.00 0.00 C

ATOM 3692 HA ALA 231 38.350 28.260 69.340 0.00 0.00 H

ATOM 3693 CB ALA 231 37.400 28.880 67.510 0.00 0.00 C

ATOM 3694 HB1 ALA 231 38.290 29.330 67.080 0.00 0.00 H

ATOM 3695 HB2 ALA 231 36.810 28.380 66.740 0.00 0.00 H

ATOM 3696 HB3 ALA 231 36.870 29.680 68.040 0.00 0.00 H

ATOM 3697 C ALA 231 36.510 27.200 69.330 0.00 0.00 C

ATOM 3698 O ALA 231 36.070 27.700 70.340 0.00 0.00 O

ATOM 3699 N LEU 232 35.940 26.140 68.730 0.00 0.00 N

ATOM 3700 H LEU 232 36.400 25.850 67.880 0.00 0.00 H

ATOM 3701 CA LEU 232 34.720 25.430 69.200 0.00 0.00 C

ATOM 3702 HA LEU 232 34.220 26.090 69.910 0.00 0.00 H

ATOM 3703 CB LEU 232 33.750 25.230 68.030 0.00 0.00 C

ATOM 3704 HB1 LEU 232 32.930 24.670 68.480 0.00 0.00 H

ATOM 3705 HB2 LEU 232 34.160 24.630 67.220 0.00 0.00 H

ATOM 3706 CG LEU 232 33.160 26.550 67.410 0.00 0.00 C

ATOM 3707 HG LEU 232 33.960 27.210 67.090 0.00 0.00 H

ATOM 3708 CD1 LEU 232 32.420 26.130 66.150 0.00 0.00 C

ATOM 3709 HD11 LEU 232 31.770 25.270 66.310 0.00 0.00 H

ATOM 3710 HD12 LEU 232 31.800 26.940 65.780 0.00 0.00 H

ATOM 3711 HD13 LEU 232 33.200 25.880 65.430 0.00 0.00 H

ATOM 3712 CD2 LEU 232 32.290 27.240 68.440 0.00 0.00 C

ATOM 3713 HD21 LEU 232 31.840 28.120 67.980 0.00 0.00 H

ATOM 3714 HD22 LEU 232 31.520 26.550 68.790 0.00 0.00 H

ATOM 3715 HD23 LEU 232 32.920 27.650 69.230 0.00 0.00 H

ATOM 3716 C LEU 232 34.940 24.120 69.960 0.00 0.00 C

ATOM 3717 O LEU 232 34.100 23.870 70.760 0.00 0.00 O

ATOM 3718 N VAL 233 36.100 23.410 69.890 0.00 0.00 N

ATOM 3719 H VAL 233 36.660 23.580 69.070 0.00 0.00 H

ATOM 3720 CA VAL 233 36.540 22.360 70.840 0.00 0.00 C

ATOM 3721 HA VAL 233 35.770 21.600 70.990 0.00 0.00 H

ATOM 3722 CB VAL 233 37.680 21.550 70.200 0.00 0.00 C

ATOM 3723 HB VAL 233 38.380 22.230 69.710 0.00 0.00 H

ATOM 3724 CG1 VAL 233 38.460 20.620 71.130 0.00 0.00 C

ATOM 3725 HG11 VAL 233 39.070 19.980 70.480 0.00 0.00 H

ATOM 3726 HG12 VAL 233 39.080 21.230 71.780 0.00 0.00 H

ATOM 3727 HG13 VAL 233 37.730 19.980 71.630 0.00 0.00 H

ATOM 3728 CG2 VAL 233 37.160 20.700 69.010 0.00 0.00 C

ATOM 3729 HG21 VAL 233 37.930 20.040 68.610 0.00 0.00 H

ATOM 3730 HG22 VAL 233 36.350 20.110 69.420 0.00 0.00 H

ATOM 3731 HG23 VAL 233 36.850 21.390 68.220 0.00 0.00 H

ATOM 3732 C VAL 233 36.920 22.830 72.250 0.00 0.00 C

ATOM 3733 O VAL 233 36.510 22.300 73.290 0.00 0.00 O

ATOM 3734 N LEU 234 37.740 23.920 72.260 0.00 0.00 N

ATOM 3735 H LEU 234 38.100 24.250 71.370 0.00 0.00 H

ATOM 3736 CA LEU 234 38.140 24.630 73.490 0.00 0.00 C

ATOM 3737 HA LEU 234 38.930 23.990 73.870 0.00 0.00 H

ATOM 3738 CB LEU 234 38.890 25.950 73.030 0.00 0.00 C

ATOM 3739 HB1 LEU 234 38.300 26.460 72.270 0.00 0.00 H

ATOM 3740 HB2 LEU 234 39.890 25.740 72.660 0.00 0.00 H

ATOM 3741 CG LEU 234 39.140 26.870 74.250 0.00 0.00 C

ATOM 3742 HG LEU 234 38.210 27.010 74.790 0.00 0.00 H

ATOM 3743 CD1 LEU 234 40.100 26.420 75.290 0.00 0.00 C

ATOM 3744 HD11 LEU 234 41.130 26.360 74.930 0.00 0.00 H

ATOM 3745 HD12 LEU 234 40.190 27.050 76.170 0.00 0.00 H

ATOM 3746 HD13 LEU 234 39.740 25.460 75.660 0.00 0.00 H

ATOM 3747 CD2 LEU 234 39.590 28.310 73.780 0.00 0.00 C

ATOM 3748 HD21 LEU 234 40.210 28.700 74.580 0.00 0.00 H

ATOM 3749 HD22 LEU 234 40.350 28.240 73.010 0.00 0.00 H

ATOM 3750 HD23 LEU 234 38.710 28.870 73.450 0.00 0.00 H

ATOM 3751 C LEU 234 37.110 24.630 74.600 0.00 0.00 C

ATOM 3752 O LEU 234 37.470 23.990 75.650 0.00 0.00 O

ATOM 3753 N PRO 235 36.000 25.450 74.500 0.00 0.00 N

ATOM 3754 CD PRO 235 35.540 25.950 73.180 0.00 0.00 C

ATOM 3755 HD1 PRO 235 35.340 25.080 72.560 0.00 0.00 H

ATOM 3756 HD2 PRO 235 36.230 26.690 72.770 0.00 0.00 H

ATOM 3757 CG PRO 235 34.260 26.690 73.510 0.00 0.00 C

ATOM 3758 HG1 PRO 235 33.590 26.630 72.650 0.00 0.00 H

ATOM 3759 HG2 PRO 235 34.460 27.730 73.750 0.00 0.00 H

ATOM 3760 CB PRO 235 33.790 25.790 74.700 0.00 0.00 C

ATOM 3761 HB1 PRO 235 33.460 24.860 74.240 0.00 0.00 H

ATOM 3762 HB2 PRO 235 32.990 26.260 75.270 0.00 0.00 H

ATOM 3763 CA PRO 235 35.010 25.550 75.560 0.00 0.00 C

ATOM 3764 HA PRO 235 35.210 26.410 76.200 0.00 0.00 H

ATOM 3765 C PRO 235 34.790 24.290 76.480 0.00 0.00 C

ATOM 3766 O PRO 235 35.040 24.340 77.620 0.00 0.00 O

ATOM 3767 N PHE 236 34.280 23.140 75.980 0.00 0.00 N

ATOM 3768 H PHE 236 34.080 23.080 75.000 0.00 0.00 H

ATOM 3769 CA PHE 236 33.950 21.890 76.670 0.00 0.00 C

ATOM 3770 HA PHE 236 33.320 22.270 77.480 0.00 0.00 H

ATOM 3771 CB PHE 236 33.030 21.160 75.660 0.00 0.00 C

ATOM 3772 HB1 PHE 236 32.120 21.750 75.560 0.00 0.00 H

ATOM 3773 HB2 PHE 236 32.670 20.220 76.090 0.00 0.00 H

ATOM 3774 CG PHE 236 33.550 20.930 74.210 0.00 0.00 C

ATOM 3775 CD1 PHE 236 32.940 21.640 73.170 0.00 0.00 C

ATOM 3776 HD1 PHE 236 32.250 22.450 73.340 0.00 0.00 H

ATOM 3777 CE1 PHE 236 33.180 21.150 71.900 0.00 0.00 C

ATOM 3778 HE1 PHE 236 32.660 21.720 71.140 0.00 0.00 H

ATOM 3779 CZ PHE 236 33.910 20.040 71.600 0.00 0.00 C

ATOM 3780 HZ PHE 236 34.080 19.680 70.590 0.00 0.00 H

ATOM 3781 CE2 PHE 236 34.660 19.420 72.580 0.00 0.00 C

ATOM 3782 HE2 PHE 236 35.320 18.590 72.380 0.00 0.00 H

ATOM 3783 CD2 PHE 236 34.430 19.830 73.930 0.00 0.00 C

ATOM 3784 HD2 PHE 236 34.890 19.260 74.720 0.00 0.00 H

ATOM 3785 C PHE 236 35.230 21.060 77.100 0.00 0.00 C

ATOM 3786 O PHE 236 35.160 20.370 78.130 0.00 0.00 O

ATOM 3787 N CYS 237 36.390 21.190 76.420 0.00 0.00 N

ATOM 3788 H CYS 237 36.420 21.750 75.580 0.00 0.00 H

ATOM 3789 CA CYS 237 37.660 20.550 76.840 0.00 0.00 C

ATOM 3790 HA CYS 237 37.420 19.640 77.380 0.00 0.00 H

ATOM 3791 CB CYS 237 38.510 20.340 75.520 0.00 0.00 C

ATOM 3792 HB1 CYS 237 39.520 20.180 75.900 0.00 0.00 H

ATOM 3793 HB2 CYS 237 38.340 21.250 74.950 0.00 0.00 H

ATOM 3794 SG CYS 237 37.790 18.980 74.650 0.00 0.00 S

ATOM 3795 HG CYS 237 37.080 19.650 73.740 0.00 0.00 H

ATOM 3796 C CYS 237 38.370 21.420 77.940 0.00 0.00 C

ATOM 3797 O CYS 237 39.120 20.830 78.680 0.00 0.00 O

ATOM 3798 N GLU 238 38.080 22.720 78.200 0.00 0.00 N

ATOM 3799 H GLU 238 37.480 23.180 77.530 0.00 0.00 H

ATOM 3800 CA GLU 238 38.650 23.580 79.340 0.00 0.00 C

ATOM 3801 HA GLU 238 39.670 23.210 79.400 0.00 0.00 H

ATOM 3802 CB GLU 238 38.440 25.060 79.170 0.00 0.00 C

ATOM 3803 HB1 GLU 238 37.460 25.390 79.520 0.00 0.00 H

ATOM 3804 HB2 GLU 238 38.640 25.260 78.120 0.00 0.00 H

ATOM 3805 CG GLU 238 39.550 25.840 79.950 0.00 0.00 C

ATOM 3806 HG1 GLU 238 40.570 25.490 79.850 0.00 0.00 H

ATOM 3807 HG2 GLU 238 39.490 25.580 81.010 0.00 0.00 H

ATOM 3808 CD GLU 238 39.560 27.360 79.800 0.00 0.00 C

ATOM 3809 OE1 GLU 238 38.600 27.930 79.180 0.00 0.00 O

ATOM 3810 OE2 GLU 238 40.620 27.950 80.150 0.00 0.00 O1-

ATOM 3811 C GLU 238 37.800 23.150 80.630 0.00 0.00 C

ATOM 3812 O GLU 238 38.420 22.930 81.690 0.00 0.00 O

ATOM 3813 N SER 239 36.540 22.790 80.470 0.00 0.00 N

ATOM 3814 H SER 239 36.170 23.160 79.610 0.00 0.00 H

ATOM 3815 CA SER 239 35.800 22.080 81.500 0.00 0.00 C

ATOM 3816 HA SER 239 36.010 22.550 82.460 0.00 0.00 H

ATOM 3817 CB SER 239 34.290 22.170 81.230 0.00 0.00 C

ATOM 3818 HB1 SER 239 33.670 21.970 82.100 0.00 0.00 H

ATOM 3819 HB2 SER 239 33.990 21.440 80.480 0.00 0.00 H

ATOM 3820 OG SER 239 33.790 23.400 80.750 0.00 0.00 O

ATOM 3821 HG SER 239 33.040 23.200 80.180 0.00 0.00 H

ATOM 3822 C SER 239 36.140 20.600 81.650 0.00 0.00 C

ATOM 3823 O SER 239 35.280 19.840 82.060 0.00 0.00 O

ATOM 3824 N SER 240 37.330 20.160 81.170 0.00 0.00 N

ATOM 3825 H SER 240 38.080 20.840 81.130 0.00 0.00 H

ATOM 3826 CA SER 240 37.840 18.770 81.230 0.00 0.00 C

ATOM 3827 HA SER 240 37.210 18.130 81.860 0.00 0.00 H

ATOM 3828 CB SER 240 37.880 18.160 79.850 0.00 0.00 C

ATOM 3829 HB1 SER 240 38.120 17.100 79.910 0.00 0.00 H

ATOM 3830 HB2 SER 240 38.620 18.550 79.150 0.00 0.00 H

ATOM 3831 OG SER 240 36.590 18.230 79.310 0.00 0.00 O

ATOM 3832 HG SER 240 36.280 19.130 79.230 0.00 0.00 H

ATOM 3833 C SER 240 39.300 18.720 81.690 0.00 0.00 C

ATOM 3834 O SER 240 40.010 19.710 81.820 0.00 0.00 O

ATOM 3835 N CYS 241 39.860 17.550 81.990 0.00 0.00 N

ATOM 3836 H CYS 241 39.390 16.660 81.850 0.00 0.00 H

ATOM 3837 CA CYS 241 41.200 17.390 82.490 0.00 0.00 C

ATOM 3838 HA CYS 241 41.320 18.060 83.340 0.00 0.00 H

ATOM 3839 CB CYS 241 41.310 15.940 83.000 0.00 0.00 C

ATOM 3840 HB1 CYS 241 40.700 15.730 83.870 0.00 0.00 H

ATOM 3841 HB2 CYS 241 42.320 15.730 83.360 0.00 0.00 H

ATOM 3842 SG CYS 241 40.980 14.870 81.610 0.00 0.00 S

ATOM 3843 HG CYS 241 42.110 15.180 80.980 0.00 0.00 H

ATOM 3844 C CYS 241 42.370 17.820 81.530 0.00 0.00 C

ATOM 3845 O CYS 241 43.440 18.030 82.020 0.00 0.00 O

ATOM 3846 N HIS 242 42.100 18.080 80.240 0.00 0.00 N

ATOM 3847 H HIS 242 41.140 18.000 79.950 0.00 0.00 H

ATOM 3848 CA HIS 242 43.090 18.490 79.240 0.00 0.00 C

ATOM 3849 HA HIS 242 43.800 19.150 79.730 0.00 0.00 H

ATOM 3850 CB HIS 242 43.860 17.150 78.960 0.00 0.00 C

ATOM 3851 HB1 HIS 242 43.180 16.310 78.830 0.00 0.00 H

ATOM 3852 HB2 HIS 242 44.530 16.830 79.750 0.00 0.00 H

ATOM 3853 CG HIS 242 44.790 17.200 77.830 0.00 0.00 C

ATOM 3854 ND1 HIS 242 45.860 18.100 77.630 0.00 0.00 N

ATOM 3855 CE1 HIS 242 46.370 17.740 76.450 0.00 0.00 C

ATOM 3856 HE1 HIS 242 47.320 18.100 76.080 0.00 0.00 H

ATOM 3857 NE2 HIS 242 45.640 16.750 75.820 0.00 0.00 N

ATOM 3858 HE2 HIS 242 45.950 16.210 75.010 0.00 0.00 H

ATOM 3859 CD2 HIS 242 44.700 16.370 76.760 0.00 0.00 C

ATOM 3860 HD2 HIS 242 44.050 15.510 76.670 0.00 0.00 H

ATOM 3861 C HIS 242 42.550 19.030 78.040 0.00 0.00 C

ATOM 3862 O HIS 242 41.960 18.390 77.140 0.00 0.00 O

ATOM 3863 N MET 243 42.790 20.350 77.950 0.00 0.00 N

ATOM 3864 H MET 243 43.130 20.740 78.820 0.00 0.00 H

ATOM 3865 CA MET 243 42.780 21.170 76.770 0.00 0.00 C

ATOM 3866 HA MET 243 42.080 20.750 76.050 0.00 0.00 H

ATOM 3867 CB MET 243 42.240 22.570 77.000 0.00 0.00 C

ATOM 3868 HB1 MET 243 42.790 23.110 77.760 0.00 0.00 H

ATOM 3869 HB2 MET 243 41.230 22.370 77.370 0.00 0.00 H

ATOM 3870 CG MET 243 42.150 23.400 75.760 0.00 0.00 C

ATOM 3871 HG1 MET 243 43.180 23.520 75.420 0.00 0.00 H

ATOM 3872 HG2 MET 243 41.760 24.400 75.930 0.00 0.00 H

ATOM 3873 SD MET 243 41.060 22.680 74.480 0.00 0.00 S

ATOM 3874 CE MET 243 41.820 23.600 73.090 0.00 0.00 C

ATOM 3875 HE1 MET 243 41.690 24.680 73.200 0.00 0.00 H

ATOM 3876 HE2 MET 243 42.880 23.350 73.010 0.00 0.00 H

ATOM 3877 HE3 MET 243 41.400 23.210 72.160 0.00 0.00 H

ATOM 3878 C MET 243 44.230 21.250 76.180 0.00 0.00 C

ATOM 3879 O MET 243 45.160 21.960 76.720 0.00 0.00 O

ATOM 3880 N PRO 244 44.490 20.780 74.910 0.00 0.00 N

ATOM 3881 CD PRO 244 43.570 19.920 74.120 0.00 0.00 C

ATOM 3882 HD1 PRO 244 42.650 20.400 73.780 0.00 0.00 H

ATOM 3883 HD2 PRO 244 43.510 19.110 74.840 0.00 0.00 H

ATOM 3884 CG PRO 244 44.170 19.500 72.820 0.00 0.00 C

ATOM 3885 HG1 PRO 244 43.690 20.040 72.000 0.00 0.00 H

ATOM 3886 HG2 PRO 244 44.190 18.440 72.590 0.00 0.00 H

ATOM 3887 CB PRO 244 45.640 19.820 73.060 0.00 0.00 C

ATOM 3888 HB1 PRO 244 46.170 20.120 72.160 0.00 0.00 H

ATOM 3889 HB2 PRO 244 46.070 18.880 73.420 0.00 0.00 H

ATOM 3890 CA PRO 244 45.710 20.930 74.140 0.00 0.00 C

ATOM 3891 HA PRO 244 46.520 20.800 74.850 0.00 0.00 H

ATOM 3892 C PRO 244 45.710 22.340 73.600 0.00 0.00 C

ATOM 3893 O PRO 244 45.260 22.600 72.510 0.00 0.00 O

ATOM 3894 N THR 245 46.380 23.250 74.380 0.00 0.00 N

ATOM 3895 H THR 245 46.980 22.890 75.110 0.00 0.00 H

ATOM 3896 CA THR 245 46.500 24.680 73.960 0.00 0.00 C

ATOM 3897 HA THR 245 45.610 25.050 73.460 0.00 0.00 H

ATOM 3898 CB THR 245 46.700 25.610 75.180 0.00 0.00 C

ATOM 3899 HB THR 245 46.880 26.620 74.810 0.00 0.00 H

ATOM 3900 CG2 THR 245 45.510 25.620 76.060 0.00 0.00 C

ATOM 3901 HG21 THR 245 45.160 24.630 76.370 0.00 0.00 H

ATOM 3902 HG22 THR 245 45.730 26.180 76.960 0.00 0.00 H

ATOM 3903 HG23 THR 245 44.650 26.060 75.560 0.00 0.00 H

ATOM 3904 OG1 THR 245 47.840 25.230 75.920 0.00 0.00 O

ATOM 3905 HG1 THR 245 47.810 24.270 75.920 0.00 0.00 H

ATOM 3906 C THR 245 47.710 24.680 73.080 0.00 0.00 C

ATOM 3907 O THR 245 47.700 25.460 72.130 0.00 0.00 O

ATOM 3908 N LEU 246 48.770 23.890 73.370 0.00 0.00 N

ATOM 3909 H LEU 246 48.750 23.370 74.240 0.00 0.00 H

ATOM 3910 CA LEU 246 49.920 23.870 72.470 0.00 0.00 C

ATOM 3911 HA LEU 246 50.180 24.930 72.420 0.00 0.00 H

ATOM 3912 CB LEU 246 51.030 23.090 73.170 0.00 0.00 C

ATOM 3913 HB1 LEU 246 50.650 22.070 73.160 0.00 0.00 H

ATOM 3914 HB2 LEU 246 51.020 23.460 74.200 0.00 0.00 H

ATOM 3915 CG LEU 246 52.490 23.110 72.630 0.00 0.00 C

ATOM 3916 HG LEU 246 52.520 22.650 71.650 0.00 0.00 H

ATOM 3917 CD1 LEU 246 53.060 24.580 72.420 0.00 0.00 C

ATOM 3918 HD11 LEU 246 53.050 25.160 73.350 0.00 0.00 H

ATOM 3919 HD12 LEU 246 54.030 24.500 71.940 0.00 0.00 H

ATOM 3920 HD13 LEU 246 52.440 25.170 71.740 0.00 0.00 H

ATOM 3921 CD2 LEU 246 53.430 22.360 73.520 0.00 0.00 C

ATOM 3922 HD21 LEU 246 53.370 22.750 74.540 0.00 0.00 H

ATOM 3923 HD22 LEU 246 53.250 21.290 73.550 0.00 0.00 H

ATOM 3924 HD23 LEU 246 54.450 22.600 73.220 0.00 0.00 H

ATOM 3925 C LEU 246 49.590 23.500 71.020 0.00 0.00 C

ATOM 3926 O LEU 246 50.100 24.180 70.130 0.00 0.00 O

ATOM 3927 N LEU 247 48.790 22.500 70.630 0.00 0.00 N

ATOM 3928 H LEU 247 48.480 21.880 71.370 0.00 0.00 H

ATOM 3929 CA LEU 247 48.170 22.330 69.320 0.00 0.00 C

ATOM 3930 HA LEU 247 48.920 22.050 68.580 0.00 0.00 H

ATOM 3931 CB LEU 247 47.190 21.160 69.370 0.00 0.00 C

ATOM 3932 HB1 LEU 247 46.460 21.300 70.170 0.00 0.00 H

ATOM 3933 HB2 LEU 247 47.790 20.310 69.700 0.00 0.00 H

ATOM 3934 CG LEU 247 46.500 20.990 68.020 0.00 0.00 C

ATOM 3935 HG LEU 247 47.270 21.040 67.250 0.00 0.00 H

ATOM 3936 CD1 LEU 247 45.950 19.510 68.120 0.00 0.00 C

ATOM 3937 HD11 LEU 247 45.330 19.220 67.260 0.00 0.00 H

ATOM 3938 HD12 LEU 247 46.810 18.870 67.930 0.00 0.00 H

ATOM 3939 HD13 LEU 247 45.430 19.360 69.060 0.00 0.00 H

ATOM 3940 CD2 LEU 247 45.390 21.970 67.640 0.00 0.00 C

ATOM 3941 HD21 LEU 247 45.850 22.900 67.320 0.00 0.00 H

ATOM 3942 HD22 LEU 247 44.970 21.600 66.710 0.00 0.00 H

ATOM 3943 HD23 LEU 247 44.680 22.160 68.450 0.00 0.00 H

ATOM 3944 C LEU 247 47.600 23.680 68.900 0.00 0.00 C

ATOM 3945 O LEU 247 48.020 24.270 67.890 0.00 0.00 O

ATOM 3946 N GLY 248 46.650 24.210 69.620 0.00 0.00 N

ATOM 3947 H GLY 248 46.240 23.680 70.380 0.00 0.00 H

ATOM 3948 CA GLY 248 45.910 25.440 69.310 0.00 0.00 C

ATOM 3949 HA1 GLY 248 45.450 25.740 70.250 0.00 0.00 H

ATOM 3950 HA2 GLY 248 45.100 25.230 68.610 0.00 0.00 H

ATOM 3951 C GLY 248 46.820 26.610 68.950 0.00 0.00 C

ATOM 3952 O GLY 248 46.530 27.260 68.000 0.00 0.00 O

ATOM 3953 N ALA 249 47.900 26.870 69.690 0.00 0.00 N

ATOM 3954 H ALA 249 47.910 26.370 70.560 0.00 0.00 H

ATOM 3955 CA ALA 249 48.970 27.800 69.380 0.00 0.00 C

ATOM 3956 HA ALA 249 48.550 28.790 69.220 0.00 0.00 H

ATOM 3957 CB ALA 249 50.020 27.850 70.540 0.00 0.00 C

ATOM 3958 HB1 ALA 249 49.530 28.090 71.480 0.00 0.00 H

ATOM 3959 HB2 ALA 249 50.650 26.970 70.720 0.00 0.00 H

ATOM 3960 HB3 ALA 249 50.750 28.620 70.310 0.00 0.00 H

ATOM 3961 C ALA 249 49.820 27.600 68.140 0.00 0.00 C

ATOM 3962 O ALA 249 50.110 28.530 67.380 0.00 0.00 O

ATOM 3963 N ILE 250 50.120 26.390 67.750 0.00 0.00 N

ATOM 3964 H ILE 250 49.850 25.550 68.250 0.00 0.00 H

ATOM 3965 CA ILE 250 50.790 26.050 66.400 0.00 0.00 C

ATOM 3966 HA ILE 250 51.510 26.820 66.140 0.00 0.00 H

ATOM 3967 CB ILE 250 51.400 24.650 66.490 0.00 0.00 C

ATOM 3968 HB ILE 250 50.550 24.020 66.750 0.00 0.00 H

ATOM 3969 CG2 ILE 250 51.960 24.110 65.090 0.00 0.00 C

ATOM 3970 HG21 ILE 250 52.790 24.660 64.660 0.00 0.00 H

ATOM 3971 HG22 ILE 250 52.370 23.100 65.200 0.00 0.00 H

ATOM 3972 HG23 ILE 250 51.150 24.080 64.350 0.00 0.00 H

ATOM 3973 CG1 ILE 250 52.510 24.600 67.550 0.00 0.00 C

ATOM 3974 HG11 ILE 250 52.080 24.980 68.470 0.00 0.00 H

ATOM 3975 HG12 ILE 250 53.230 25.320 67.160 0.00 0.00 H

ATOM 3976 CD ILE 250 53.000 23.160 68.010 0.00 0.00 C

ATOM 3977 HD1 ILE 250 53.450 22.700 67.130 0.00 0.00 H

ATOM 3978 HD2 ILE 250 53.830 23.250 68.710 0.00 0.00 H

ATOM 3979 HD3 ILE 250 52.200 22.610 68.510 0.00 0.00 H

ATOM 3980 C ILE 250 49.810 26.170 65.170 0.00 0.00 C

ATOM 3981 O ILE 250 50.230 26.570 64.080 0.00 0.00 O

ATOM 3982 N ILE 251 48.580 25.690 65.290 0.00 0.00 N

ATOM 3983 H ILE 251 48.390 25.390 66.240 0.00 0.00 H

ATOM 3984 CA ILE 251 47.530 25.740 64.220 0.00 0.00 C

ATOM 3985 HA ILE 251 48.000 25.370 63.310 0.00 0.00 H

ATOM 3986 CB ILE 251 46.470 24.750 64.720 0.00 0.00 C

ATOM 3987 HB ILE 251 46.810 24.210 65.610 0.00 0.00 H

ATOM 3988 CG2 ILE 251 45.030 25.270 64.980 0.00 0.00 C

ATOM 3989 HG21 ILE 251 44.540 25.710 64.110 0.00 0.00 H

ATOM 3990 HG22 ILE 251 44.330 24.510 65.320 0.00 0.00 H

ATOM 3991 HG23 ILE 251 45.140 26.060 65.730 0.00 0.00 H

ATOM 3992 CG1 ILE 251 46.300 23.540 63.710 0.00 0.00 C

ATOM 3993 HG11 ILE 251 46.390 24.010 62.720 0.00 0.00 H

ATOM 3994 HG12 ILE 251 45.360 22.990 63.760 0.00 0.00 H

ATOM 3995 CD ILE 251 47.500 22.550 63.860 0.00 0.00 C

ATOM 3996 HD1 ILE 251 48.500 22.990 63.860 0.00 0.00 H

ATOM 3997 HD2 ILE 251 47.350 22.020 64.810 0.00 0.00 H

ATOM 3998 HD3 ILE 251 47.510 21.880 63.000 0.00 0.00 H

ATOM 3999 C ILE 251 47.050 27.230 63.970 0.00 0.00 C

ATOM 4000 O ILE 251 46.770 27.640 62.820 0.00 0.00 O

ATOM 4001 N ASN 252 47.080 28.070 65.020 0.00 0.00 N

ATOM 4002 H ASN 252 47.220 27.690 65.940 0.00 0.00 H

ATOM 4003 CA ASN 252 47.010 29.490 64.880 0.00 0.00 C

ATOM 4004 HA ASN 252 46.010 29.710 64.490 0.00 0.00 H

ATOM 4005 CB ASN 252 47.150 30.140 66.280 0.00 0.00 C

ATOM 4006 HB1 ASN 252 48.110 29.850 66.710 0.00 0.00 H

ATOM 4007 HB2 ASN 252 46.350 29.940 66.990 0.00 0.00 H

ATOM 4008 CG ASN 252 47.060 31.640 66.280 0.00 0.00 C

ATOM 4009 OD1 ASN 252 46.290 32.240 65.520 0.00 0.00 O

ATOM 4010 ND2 ASN 252 47.790 32.280 67.190 0.00 0.00 N

ATOM 4011 HD21 ASN 252 48.520 31.800 67.700 0.00 0.00 H

ATOM 4012 HD22 ASN 252 47.630 33.270 67.290 0.00 0.00 H

ATOM 4013 C ASN 252 48.140 29.990 63.910 0.00 0.00 C

ATOM 4014 O ASN 252 47.930 30.860 63.080 0.00 0.00 O

ATOM 4015 N TRP 253 49.310 29.350 63.910 0.00 0.00 N

ATOM 4016 H TRP 253 49.470 28.510 64.450 0.00 0.00 H

ATOM 4017 CA TRP 253 50.330 29.770 63.020 0.00 0.00 C

ATOM 4018 HA TRP 253 50.220 30.820 62.750 0.00 0.00 H

ATOM 4019 CB TRP 253 51.750 29.530 63.650 0.00 0.00 C

ATOM 4020 HB1 TRP 253 52.560 29.410 62.940 0.00 0.00 H

ATOM 4021 HB2 TRP 253 51.810 28.590 64.190 0.00 0.00 H

ATOM 4022 CG TRP 253 52.270 30.610 64.580 0.00 0.00 C

ATOM 4023 CD1 TRP 253 52.370 30.580 65.960 0.00 0.00 C

ATOM 4024 HD1 TRP 253 51.920 29.830 66.590 0.00 0.00 H

ATOM 4025 NE1 TRP 253 52.860 31.780 66.380 0.00 0.00 N

ATOM 4026 HE1 TRP 253 52.960 31.880 67.380 0.00 0.00 H

ATOM 4027 CE2 TRP 253 53.260 32.620 65.390 0.00 0.00 C

ATOM 4028 CZ2 TRP 253 53.900 33.880 65.320 0.00 0.00 C

ATOM 4029 HZ2 TRP 253 54.080 34.480 66.200 0.00 0.00 H

ATOM 4030 CH2 TRP 253 54.280 34.410 64.110 0.00 0.00 C

ATOM 4031 HH2 TRP 253 54.760 35.370 64.030 0.00 0.00 H

ATOM 4032 CZ3 TRP 253 53.930 33.650 62.910 0.00 0.00 C

ATOM 4033 HZ3 TRP 253 54.300 33.970 61.940 0.00 0.00 H

ATOM 4034 CE3 TRP 253 53.330 32.380 62.970 0.00 0.00 C

ATOM 4035 HE3 TRP 253 53.000 31.870 62.080 0.00 0.00 H

ATOM 4036 CD2 TRP 253 53.020 31.800 64.210 0.00 0.00 C

ATOM 4037 C TRP 253 50.240 29.080 61.610 0.00 0.00 C

ATOM 4038 O TRP 253 50.770 29.680 60.650 0.00 0.00 O

ATOM 4039 N LEU 254 49.470 28.030 61.400 0.00 0.00 N

ATOM 4040 H LEU 254 49.090 27.600 62.240 0.00 0.00 H

ATOM 4041 CA LEU 254 49.060 27.500 60.080 0.00 0.00 C

ATOM 4042 HA LEU 254 49.970 27.340 59.510 0.00 0.00 H

ATOM 4043 CB LEU 254 48.510 26.010 60.210 0.00 0.00 C

ATOM 4044 HB1 LEU 254 47.880 26.020 61.100 0.00 0.00 H

ATOM 4045 HB2 LEU 254 49.360 25.380 60.490 0.00 0.00 H

ATOM 4046 CG LEU 254 47.890 25.300 58.920 0.00 0.00 C

ATOM 4047 HG LEU 254 47.030 25.910 58.630 0.00 0.00 H

ATOM 4048 CD1 LEU 254 48.970 25.300 57.770 0.00 0.00 C

ATOM 4049 HD11 LEU 254 48.320 25.140 56.910 0.00 0.00 H

ATOM 4050 HD12 LEU 254 49.390 26.300 57.680 0.00 0.00 H

ATOM 4051 HD13 LEU 254 49.760 24.570 57.940 0.00 0.00 H

ATOM 4052 CD2 LEU 254 47.200 23.960 59.090 0.00 0.00 C

ATOM 4053 HD21 LEU 254 47.810 23.180 59.550 0.00 0.00 H

ATOM 4054 HD22 LEU 254 46.330 24.130 59.740 0.00 0.00 H

ATOM 4055 HD23 LEU 254 46.740 23.710 58.140 0.00 0.00 H

ATOM 4056 C LEU 254 48.200 28.490 59.270 0.00 0.00 C

ATOM 4057 O LEU 254 48.550 28.920 58.110 0.00 0.00 O

ATOM 4058 N GLY 255 47.280 29.150 60.060 0.00 0.00 N

ATOM 4059 H GLY 255 47.160 28.820 61.010 0.00 0.00 H

ATOM 4060 CA GLY 255 46.410 30.250 59.660 0.00 0.00 C

ATOM 4061 HA1 GLY 255 45.850 30.570 60.540 0.00 0.00 H

ATOM 4062 HA2 GLY 255 45.870 30.010 58.740 0.00 0.00 H

ATOM 4063 C GLY 255 47.270 31.470 59.210 0.00 0.00 C

ATOM 4064 O GLY 255 47.200 31.980 58.100 0.00 0.00 O

ATOM 4065 N TYR 256 48.280 31.720 60.060 0.00 0.00 N

ATOM 4066 H TYR 256 48.360 31.220 60.930 0.00 0.00 H

ATOM 4067 CA TYR 256 49.180 32.850 59.760 0.00 0.00 C

ATOM 4068 HA TYR 256 48.560 33.630 59.320 0.00 0.00 H

ATOM 4069 CB TYR 256 49.920 33.320 60.990 0.00 0.00 C

ATOM 4070 HB1 TYR 256 50.650 34.080 60.690 0.00 0.00 H

ATOM 4071 HB2 TYR 256 50.430 32.440 61.380 0.00 0.00 H

ATOM 4072 CG TYR 256 49.170 34.020 62.110 0.00 0.00 C

ATOM 4073 CD1 TYR 256 48.300 35.110 61.760 0.00 0.00 C

ATOM 4074 HD1 TYR 256 48.280 35.410 60.730 0.00 0.00 H

ATOM 4075 CE1 TYR 256 47.590 35.900 62.710 0.00 0.00 C

ATOM 4076 HE1 TYR 256 46.940 36.700 62.410 0.00 0.00 H

ATOM 4077 CZ TYR 256 47.720 35.430 64.050 0.00 0.00 C

ATOM 4078 OH TYR 256 47.060 36.070 64.950 0.00 0.00 O

ATOM 4079 HH TYR 256 46.510 36.780 64.610 0.00 0.00 H

ATOM 4080 CE2 TYR 256 48.610 34.370 64.410 0.00 0.00 C

ATOM 4081 HE2 TYR 256 48.640 34.200 65.480 0.00 0.00 H

ATOM 4082 CD2 TYR 256 49.260 33.630 63.450 0.00 0.00 C

ATOM 4083 HD2 TYR 256 49.850 32.780 63.770 0.00 0.00 H

ATOM 4084 C TYR 256 50.180 32.520 58.560 0.00 0.00 C

ATOM 4085 O TYR 256 50.820 33.440 58.020 0.00 0.00 O

ATOM 4086 N SER 257 50.520 31.260 58.410 0.00 0.00 N

ATOM 4087 H SER 257 49.960 30.550 58.880 0.00 0.00 H

ATOM 4088 CA SER 257 51.430 30.780 57.310 0.00 0.00 C

ATOM 4089 HA SER 257 52.370 31.330 57.330 0.00 0.00 H

ATOM 4090 CB SER 257 51.810 29.260 57.490 0.00 0.00 C

ATOM 4091 HB1 SER 257 52.120 29.080 58.520 0.00 0.00 H

ATOM 4092 HB2 SER 257 52.720 29.020 56.930 0.00 0.00 H

ATOM 4093 OG SER 257 50.900 28.350 57.040 0.00 0.00 O

ATOM 4094 HG SER 257 50.010 28.560 57.350 0.00 0.00 H

ATOM 4095 C SER 257 50.780 30.990 55.950 0.00 0.00 C

ATOM 4096 O SER 257 51.460 31.110 54.930 0.00 0.00 O

ATOM 4097 N ASN 258 49.430 31.140 55.870 0.00 0.00 N

ATOM 4098 H ASN 258 48.900 30.950 56.710 0.00 0.00 H

ATOM 4099 CA ASN 258 48.670 31.600 54.670 0.00 0.00 C

ATOM 4100 HA ASN 258 48.840 30.860 53.890 0.00 0.00 H

ATOM 4101 CB ASN 258 47.150 31.740 55.090 0.00 0.00 C

ATOM 4102 HB1 ASN 258 47.030 32.600 55.760 0.00 0.00 H

ATOM 4103 HB2 ASN 258 46.830 30.880 55.670 0.00 0.00 H

ATOM 4104 CG ASN 258 46.350 31.870 53.780 0.00 0.00 C

ATOM 4105 OD1 ASN 258 46.470 31.050 52.880 0.00 0.00 O

ATOM 4106 ND2 ASN 258 45.560 32.830 53.620 0.00 0.00 N

ATOM 4107 HD21 ASN 258 45.530 33.610 54.260 0.00 0.00 H

ATOM 4108 HD22 ASN 258 45.130 32.880 52.700 0.00 0.00 H

ATOM 4109 C ASN 258 49.130 32.980 54.180 0.00 0.00 C

ATOM 4110 O ASN 258 49.100 33.270 52.980 0.00 0.00 O

ATOM 4111 N SER 259 49.660 33.840 55.090 0.00 0.00 N

ATOM 4112 H SER 259 49.350 33.730 56.050 0.00 0.00 H

ATOM 4113 CA SER 259 50.280 35.110 54.680 0.00 0.00 C

ATOM 4114 HA SER 259 49.590 35.750 54.120 0.00 0.00 H

ATOM 4115 CB SER 259 50.710 35.930 55.940 0.00 0.00 C

ATOM 4116 HB1 SER 259 51.060 36.900 55.600 0.00 0.00 H

ATOM 4117 HB2 SER 259 51.570 35.480 56.440 0.00 0.00 H

ATOM 4118 OG SER 259 49.640 36.000 56.880 0.00 0.00 O

ATOM 4119 HG SER 259 48.880 36.260 56.360 0.00 0.00 H

ATOM 4120 C SER 259 51.510 34.910 53.740 0.00 0.00 C

ATOM 4121 O SER 259 52.090 35.820 53.160 0.00 0.00 O

ATOM 4122 N LEU 260 51.920 33.650 53.460 0.00 0.00 N

ATOM 4123 H LEU 260 51.480 32.900 53.970 0.00 0.00 H

ATOM 4124 CA LEU 260 53.000 33.230 52.510 0.00 0.00 C

ATOM 4125 HA LEU 260 53.390 34.120 52.020 0.00 0.00 H

ATOM 4126 CB LEU 260 54.210 32.710 53.300 0.00 0.00 C

ATOM 4127 HB1 LEU 260 53.900 31.830 53.880 0.00 0.00 H

ATOM 4128 HB2 LEU 260 54.410 33.490 54.030 0.00 0.00 H

ATOM 4129 CG LEU 260 55.440 32.110 52.590 0.00 0.00 C

ATOM 4130 HG LEU 260 55.140 31.110 52.270 0.00 0.00 H

ATOM 4131 CD1 LEU 260 55.970 33.030 51.550 0.00 0.00 C

ATOM 4132 HD11 LEU 260 56.850 32.630 51.050 0.00 0.00 H

ATOM 4133 HD12 LEU 260 55.230 33.320 50.800 0.00 0.00 H

ATOM 4134 HD13 LEU 260 56.340 33.970 51.970 0.00 0.00 H

ATOM 4135 CD2 LEU 260 56.520 31.900 53.570 0.00 0.00 C

ATOM 4136 HD21 LEU 260 56.180 31.300 54.420 0.00 0.00 H

ATOM 4137 HD22 LEU 260 57.410 31.440 53.140 0.00 0.00 H

ATOM 4138 HD23 LEU 260 56.830 32.840 54.020 0.00 0.00 H

ATOM 4139 C LEU 260 52.620 32.170 51.460 0.00 0.00 C

ATOM 4140 O LEU 260 52.990 32.250 50.300 0.00 0.00 O

ATOM 4141 N LEU 261 51.870 31.180 51.910 0.00 0.00 N

ATOM 4142 H LEU 261 51.660 31.190 52.900 0.00 0.00 H

ATOM 4143 CA LEU 261 51.500 29.990 51.190 0.00 0.00 C

ATOM 4144 HA LEU 261 52.420 29.540 50.820 0.00 0.00 H

ATOM 4145 CB LEU 261 50.880 28.970 52.230 0.00 0.00 C

ATOM 4146 HB1 LEU 261 49.940 29.350 52.610 0.00 0.00 H

ATOM 4147 HB2 LEU 261 51.620 28.820 53.020 0.00 0.00 H

ATOM 4148 CG LEU 261 50.780 27.540 51.720 0.00 0.00 C

ATOM 4149 HG LEU 261 50.180 27.660 50.810 0.00 0.00 H

ATOM 4150 CD1 LEU 261 52.180 26.860 51.500 0.00 0.00 C

ATOM 4151 HD11 LEU 261 52.780 26.870 52.410 0.00 0.00 H

ATOM 4152 HD12 LEU 261 52.020 25.810 51.260 0.00 0.00 H

ATOM 4153 HD13 LEU 261 52.670 27.250 50.610 0.00 0.00 H

ATOM 4154 CD2 LEU 261 49.950 26.670 52.680 0.00 0.00 C

ATOM 4155 HD21 LEU 261 48.940 27.060 52.790 0.00 0.00 H

ATOM 4156 HD22 LEU 261 49.890 25.690 52.200 0.00 0.00 H

ATOM 4157 HD23 LEU 261 50.370 26.700 53.680 0.00 0.00 H

ATOM 4158 C LEU 261 50.640 30.340 49.950 0.00 0.00 C

ATOM 4159 O LEU 261 51.110 30.040 48.810 0.00 0.00 O

ATOM 4160 N ASN 262 49.600 31.210 50.090 0.00 0.00 N

ATOM 4161 H ASN 262 49.290 31.580 50.980 0.00 0.00 H

ATOM 4162 CA ASN 262 48.880 31.720 48.940 0.00 0.00 C

ATOM 4163 HA ASN 262 48.720 30.810 48.370 0.00 0.00 H

ATOM 4164 CB ASN 262 47.500 32.300 49.500 0.00 0.00 C

ATOM 4165 HB1 ASN 262 47.390 33.270 49.010 0.00 0.00 H

ATOM 4166 HB2 ASN 262 47.440 32.260 50.580 0.00 0.00 H

ATOM 4167 CG ASN 262 46.300 31.580 49.010 0.00 0.00 C

ATOM 4168 OD1 ASN 262 46.130 31.140 47.890 0.00 0.00 O

ATOM 4169 ND2 ASN 262 45.410 31.260 49.890 0.00 0.00 N

ATOM 4170 HD21 ASN 262 45.480 31.580 50.850 0.00 0.00 H

ATOM 4171 HD22 ASN 262 44.570 30.740 49.670 0.00 0.00 H

ATOM 4172 C ASN 262 49.750 32.620 48.030 0.00 0.00 C

ATOM 4173 O ASN 262 49.680 32.410 46.830 0.00 0.00 O

ATOM 4174 N PRO 263 50.410 33.670 48.530 0.00 0.00 N

ATOM 4175 CD PRO 263 50.170 34.280 49.890 0.00 0.00 C

ATOM 4176 HD1 PRO 263 50.510 33.630 50.690 0.00 0.00 H

ATOM 4177 HD2 PRO 263 49.110 34.540 49.940 0.00 0.00 H

ATOM 4178 CG PRO 263 51.010 35.510 49.910 0.00 0.00 C

ATOM 4179 HG1 PRO 263 51.430 35.710 50.900 0.00 0.00 H

ATOM 4180 HG2 PRO 263 50.330 36.340 49.700 0.00 0.00 H

ATOM 4181 CB PRO 263 52.120 35.340 48.950 0.00 0.00 C

ATOM 4182 HB1 PRO 263 52.940 34.840 49.470 0.00 0.00 H

ATOM 4183 HB2 PRO 263 52.520 36.300 48.620 0.00 0.00 H

ATOM 4184 CA PRO 263 51.450 34.490 47.830 0.00 0.00 C

ATOM 4185 HA PRO 263 50.900 35.110 47.110 0.00 0.00 H

ATOM 4186 C PRO 263 52.410 33.650 46.950 0.00 0.00 C

ATOM 4187 O PRO 263 52.600 33.890 45.780 0.00 0.00 O

ATOM 4188 N VAL 264 52.900 32.490 47.430 0.00 0.00 N

ATOM 4189 H VAL 264 52.950 32.440 48.440 0.00 0.00 H

ATOM 4190 CA VAL 264 53.590 31.380 46.720 0.00 0.00 C

ATOM 4191 HA VAL 264 54.360 31.930 46.190 0.00 0.00 H

ATOM 4192 CB VAL 264 54.400 30.390 47.650 0.00 0.00 C

ATOM 4193 HB VAL 264 53.690 30.130 48.440 0.00 0.00 H

ATOM 4194 CG1 VAL 264 54.790 29.100 46.920 0.00 0.00 C

ATOM 4195 HG11 VAL 264 55.210 28.440 47.680 0.00 0.00 H

ATOM 4196 HG12 VAL 264 53.920 28.670 46.420 0.00 0.00 H

ATOM 4197 HG13 VAL 264 55.510 29.230 46.110 0.00 0.00 H

ATOM 4198 CG2 VAL 264 55.720 31.120 48.210 0.00 0.00 C

ATOM 4199 HG21 VAL 264 56.360 30.430 48.750 0.00 0.00 H

ATOM 4200 HG22 VAL 264 56.240 31.650 47.410 0.00 0.00 H

ATOM 4201 HG23 VAL 264 55.340 31.890 48.880 0.00 0.00 H

ATOM 4202 C VAL 264 52.740 30.710 45.650 0.00 0.00 C

ATOM 4203 O VAL 264 53.060 30.590 44.450 0.00 0.00 O

ATOM 4204 N ILE 265 51.530 30.320 46.100 0.00 0.00 N

ATOM 4205 H ILE 265 51.300 30.440 47.070 0.00 0.00 H

ATOM 4206 CA ILE 265 50.720 29.390 45.340 0.00 0.00 C

ATOM 4207 HA ILE 265 51.400 28.670 44.890 0.00 0.00 H

ATOM 4208 CB ILE 265 49.710 28.560 46.130 0.00 0.00 C

ATOM 4209 HB ILE 265 50.190 28.480 47.100 0.00 0.00 H

ATOM 4210 CG2 ILE 265 48.260 29.070 46.350 0.00 0.00 C

ATOM 4211 HG21 ILE 265 48.190 30.160 46.270 0.00 0.00 H

ATOM 4212 HG22 ILE 265 47.560 28.580 45.670 0.00 0.00 H

ATOM 4213 HG23 ILE 265 47.860 28.840 47.340 0.00 0.00 H

ATOM 4214 CG1 ILE 265 49.760 27.130 45.510 0.00 0.00 C

ATOM 4215 HG11 ILE 265 50.720 26.840 45.090 0.00 0.00 H

ATOM 4216 HG12 ILE 265 49.080 27.100 44.670 0.00 0.00 H

ATOM 4217 CD ILE 265 49.390 25.980 46.390 0.00 0.00 C

ATOM 4218 HD1 ILE 265 50.140 25.830 47.170 0.00 0.00 H

ATOM 4219 HD2 ILE 265 48.440 26.160 46.890 0.00 0.00 H

ATOM 4220 HD3 ILE 265 49.340 25.060 45.800 0.00 0.00 H

ATOM 4221 C ILE 265 50.100 30.080 44.120 0.00 0.00 C

ATOM 4222 O ILE 265 49.820 29.450 43.120 0.00 0.00 O

ATOM 4223 N TYR 266 49.900 31.460 44.110 0.00 0.00 N

ATOM 4224 H TYR 266 50.010 31.950 44.990 0.00 0.00 H

ATOM 4225 CA TYR 266 49.590 32.300 42.960 0.00 0.00 C

ATOM 4226 HA TYR 266 48.550 32.170 42.650 0.00 0.00 H

ATOM 4227 CB TYR 266 49.700 33.790 43.280 0.00 0.00 C

ATOM 4228 HB1 TYR 266 49.520 34.270 42.320 0.00 0.00 H

ATOM 4229 HB2 TYR 266 50.710 34.040 43.600 0.00 0.00 H

ATOM 4230 CG TYR 266 48.700 34.350 44.390 0.00 0.00 C

ATOM 4231 CD1 TYR 266 48.980 35.520 45.100 0.00 0.00 C

ATOM 4232 HD1 TYR 266 49.860 36.030 44.730 0.00 0.00 H

ATOM 4233 CE1 TYR 266 48.250 35.990 46.220 0.00 0.00 C

ATOM 4234 HE1 TYR 266 48.470 36.840 46.860 0.00 0.00 H

ATOM 4235 CZ TYR 266 47.060 35.290 46.470 0.00 0.00 C

ATOM 4236 OH TYR 266 46.230 35.650 47.490 0.00 0.00 O

ATOM 4237 HH TYR 266 46.580 36.360 48.030 0.00 0.00 H

ATOM 4238 CE2 TYR 266 46.690 34.120 45.720 0.00 0.00 C

ATOM 4239 HE2 TYR 266 45.790 33.600 46.010 0.00 0.00 H

ATOM 4240 CD2 TYR 266 47.570 33.610 44.790 0.00 0.00 C

ATOM 4241 HD2 TYR 266 47.420 32.580 44.500 0.00 0.00 H

ATOM 4242 C TYR 266 50.440 31.980 41.770 0.00 0.00 C

ATOM 4243 O TYR 266 49.900 32.030 40.700 0.00 0.00 O

ATOM 4244 N ALA 267 51.680 31.620 41.940 0.00 0.00 N

ATOM 4245 H ALA 267 52.160 31.690 42.820 0.00 0.00 H

ATOM 4246 CA ALA 267 52.520 31.180 40.790 0.00 0.00 C

ATOM 4247 HA ALA 267 52.490 31.960 40.030 0.00 0.00 H

ATOM 4248 CB ALA 267 53.950 31.050 41.270 0.00 0.00 C

ATOM 4249 HB1 ALA 267 53.950 30.250 42.020 0.00 0.00 H

ATOM 4250 HB2 ALA 267 54.580 30.770 40.430 0.00 0.00 H

ATOM 4251 HB3 ALA 267 54.250 32.050 41.600 0.00 0.00 H

ATOM 4252 C ALA 267 52.160 29.890 40.100 0.00 0.00 C

ATOM 4253 O ALA 267 52.480 29.700 38.940 0.00 0.00 O

ATOM 4254 N TYR 268 51.450 28.990 40.810 0.00 0.00 N

ATOM 4255 H TYR 268 51.000 29.230 41.680 0.00 0.00 H

ATOM 4256 CA TYR 268 50.900 27.810 40.220 0.00 0.00 C

ATOM 4257 HA TYR 268 51.500 27.540 39.340 0.00 0.00 H

ATOM 4258 CB TYR 268 50.970 26.780 41.290 0.00 0.00 C

ATOM 4259 HB1 TYR 268 50.540 27.170 42.210 0.00 0.00 H

ATOM 4260 HB2 TYR 268 52.030 26.610 41.460 0.00 0.00 H

ATOM 4261 CG TYR 268 50.390 25.490 41.010 0.00 0.00 C

ATOM 4262 CD1 TYR 268 49.500 24.820 41.990 0.00 0.00 C

ATOM 4263 HD1 TYR 268 49.170 25.470 42.780 0.00 0.00 H

ATOM 4264 CE1 TYR 268 48.900 23.600 41.710 0.00 0.00 C

ATOM 4265 HE1 TYR 268 48.330 23.130 42.490 0.00 0.00 H

ATOM 4266 CZ TYR 268 49.240 22.870 40.550 0.00 0.00 C

ATOM 4267 OH TYR 268 48.840 21.620 40.370 0.00 0.00 O

ATOM 4268 HH TYR 268 49.260 21.260 39.580 0.00 0.00 H

ATOM 4269 CE2 TYR 268 50.170 23.500 39.630 0.00 0.00 C

ATOM 4270 HE2 TYR 268 50.480 22.990 38.730 0.00 0.00 H

ATOM 4271 CD2 TYR 268 50.700 24.770 39.860 0.00 0.00 C

ATOM 4272 HD2 TYR 268 51.320 25.260 39.120 0.00 0.00 H

ATOM 4273 C TYR 268 49.430 28.020 39.810 0.00 0.00 C

ATOM 4274 O TYR 268 49.030 27.480 38.830 0.00 0.00 O

ATOM 4275 N PHE 269 48.660 28.890 40.530 0.00 0.00 N

ATOM 4276 H PHE 269 49.110 29.310 41.320 0.00 0.00 H

ATOM 4277 CA PHE 269 47.340 29.380 40.170 0.00 0.00 C

ATOM 4278 HA PHE 269 46.710 28.550 39.850 0.00 0.00 H

ATOM 4279 CB PHE 269 46.680 30.010 41.330 0.00 0.00 C

ATOM 4280 HB1 PHE 269 47.340 30.750 41.760 0.00 0.00 H

ATOM 4281 HB2 PHE 269 46.490 29.280 42.120 0.00 0.00 H

ATOM 4282 CG PHE 269 45.360 30.700 40.990 0.00 0.00 C

ATOM 4283 CD1 PHE 269 45.230 32.150 40.820 0.00 0.00 C

ATOM 4284 HD1 PHE 269 46.060 32.820 40.990 0.00 0.00 H

ATOM 4285 CE1 PHE 269 43.940 32.670 40.800 0.00 0.00 C

ATOM 4286 HE1 PHE 269 43.870 33.750 40.690 0.00 0.00 H

ATOM 4287 CZ PHE 269 42.830 31.880 40.630 0.00 0.00 C

ATOM 4288 HZ PHE 269 41.880 32.360 40.440 0.00 0.00 H

ATOM 4289 CE2 PHE 269 42.910 30.470 40.690 0.00 0.00 C

ATOM 4290 HE2 PHE 269 42.030 29.840 40.580 0.00 0.00 H

ATOM 4291 CD2 PHE 269 44.200 29.940 40.880 0.00 0.00 C

ATOM 4292 HD2 PHE 269 44.260 28.860 40.950 0.00 0.00 H

ATOM 4293 C PHE 269 47.360 30.320 38.950 0.00 0.00 C

ATOM 4294 O PHE 269 46.300 30.350 38.330 0.00 0.00 O

ATOM 4295 N ASN 270 48.430 30.980 38.570 0.00 0.00 N

ATOM 4296 H ASN 270 49.220 30.920 39.200 0.00 0.00 H

ATOM 4297 CA ASN 270 48.400 32.110 37.560 0.00 0.00 C

ATOM 4298 HA ASN 270 47.670 31.800 36.810 0.00 0.00 H

ATOM 4299 CB ASN 270 47.850 33.290 38.310 0.00 0.00 C

ATOM 4300 HB1 ASN 270 48.540 33.750 39.020 0.00 0.00 H

ATOM 4301 HB2 ASN 270 46.960 33.000 38.870 0.00 0.00 H

ATOM 4302 CG ASN 270 47.490 34.390 37.280 0.00 0.00 C

ATOM 4303 OD1 ASN 270 48.270 35.180 36.760 0.00 0.00 O

ATOM 4304 ND2 ASN 270 46.200 34.410 36.910 0.00 0.00 N

ATOM 4305 HD21 ASN 270 45.480 33.830 37.320 0.00 0.00 H

ATOM 4306 HD22 ASN 270 45.900 35.140 36.280 0.00 0.00 H

ATOM 4307 C ASN 270 49.680 32.410 36.820 0.00 0.00 C

ATOM 4308 O ASN 270 50.690 32.830 37.360 0.00 0.00 O

ATOM 4309 N LYS 271 49.580 32.330 35.490 0.00 0.00 N

ATOM 4310 H LYS 271 48.670 32.180 35.060 0.00 0.00 H

ATOM 4311 CA LYS 271 50.690 32.540 34.530 0.00 0.00 C

ATOM 4312 HA LYS 271 51.560 32.100 35.030 0.00 0.00 H

ATOM 4313 CB LYS 271 50.460 31.790 33.190 0.00 0.00 C

ATOM 4314 HB1 LYS 271 51.410 31.790 32.650 0.00 0.00 H

ATOM 4315 HB2 LYS 271 49.730 32.370 32.630 0.00 0.00 H

ATOM 4316 CG LYS 271 49.940 30.330 33.190 0.00 0.00 C

ATOM 4317 HG1 LYS 271 48.890 30.340 33.470 0.00 0.00 H

ATOM 4318 HG2 LYS 271 50.530 29.790 33.930 0.00 0.00 H

ATOM 4319 CD LYS 271 50.080 29.550 31.860 0.00 0.00 C

ATOM 4320 HD1 LYS 271 51.100 29.680 31.480 0.00 0.00 H

ATOM 4321 HD2 LYS 271 49.350 29.960 31.160 0.00 0.00 H

ATOM 4322 CE LYS 271 49.800 28.030 32.180 0.00 0.00 C

ATOM 4323 HE1 LYS 271 50.580 27.740 32.890 0.00 0.00 H

ATOM 4324 HE2 LYS 271 50.050 27.500 31.260 0.00 0.00 H

ATOM 4325 NZ LYS 271 48.410 27.850 32.730 0.00 0.00 N1+

ATOM 4326 HZ1 LYS 271 47.740 28.440 32.260 0.00 0.00 H

ATOM 4327 HZ2 LYS 271 48.090 26.900 32.590 0.00 0.00 H

ATOM 4328 HZ3 LYS 271 48.370 28.000 33.730 0.00 0.00 H

ATOM 4329 C LYS 271 51.060 34.030 34.240 0.00 0.00 C

ATOM 4330 O LYS 271 52.120 34.240 33.610 0.00 0.00 O

ATOM 4331 N ASP 272 50.270 35.010 34.740 0.00 0.00 N

ATOM 4332 H ASP 272 49.420 34.790 35.240 0.00 0.00 H

ATOM 4333 CA ASP 272 50.650 36.460 34.780 0.00 0.00 C

ATOM 4334 HA ASP 272 51.380 36.770 34.040 0.00 0.00 H

ATOM 4335 CB ASP 272 49.480 37.410 34.530 0.00 0.00 C

ATOM 4336 HB1 ASP 272 49.920 38.390 34.660 0.00 0.00 H

ATOM 4337 HB2 ASP 272 48.680 37.170 35.230 0.00 0.00 H

ATOM 4338 CG ASP 272 48.970 37.430 33.060 0.00 0.00 C

ATOM 4339 OD1 ASP 272 49.810 37.660 32.180 0.00 0.00 O

ATOM 4340 OD2 ASP 272 47.800 37.190 32.830 0.00 0.00 O1-

ATOM 4341 C ASP 272 51.310 36.790 36.130 0.00 0.00 C

ATOM 4342 O ASP 272 52.240 37.570 36.180 0.00 0.00 O

ATOM 4343 N PHE 273 50.940 36.170 37.300 0.00 0.00 N

ATOM 4344 H PHE 273 50.080 35.650 37.390 0.00 0.00 H

ATOM 4345 CA PHE 273 51.720 36.270 38.580 0.00 0.00 C

ATOM 4346 HA PHE 273 51.980 37.300 38.830 0.00 0.00 H

ATOM 4347 CB PHE 273 50.900 35.720 39.730 0.00 0.00 C

ATOM 4348 HB1 PHE 273 51.580 35.310 40.480 0.00 0.00 H

ATOM 4349 HB2 PHE 273 50.270 34.870 39.500 0.00 0.00 H

ATOM 4350 CG PHE 273 50.020 36.690 40.430 0.00 0.00 C

ATOM 4351 CD1 PHE 273 48.680 36.470 40.630 0.00 0.00 C

ATOM 4352 HD1 PHE 273 48.230 35.600 40.160 0.00 0.00 H

ATOM 4353 CE1 PHE 273 47.880 37.480 41.200 0.00 0.00 C

ATOM 4354 HE1 PHE 273 46.820 37.420 41.390 0.00 0.00 H

ATOM 4355 CZ PHE 273 48.420 38.630 41.710 0.00 0.00 C

ATOM 4356 HZ PHE 273 47.810 39.360 42.220 0.00 0.00 H

ATOM 4357 CE2 PHE 273 49.830 38.840 41.590 0.00 0.00 C

ATOM 4358 HE2 PHE 273 50.350 39.700 41.980 0.00 0.00 H

ATOM 4359 CD2 PHE 273 50.590 37.870 40.950 0.00 0.00 C

ATOM 4360 HD2 PHE 273 51.670 37.880 40.970 0.00 0.00 H

ATOM 4361 C PHE 273 53.030 35.560 38.440 0.00 0.00 C

ATOM 4362 O PHE 273 53.970 36.090 38.940 0.00 0.00 O

ATOM 4363 N GLN 274 53.080 34.440 37.740 0.00 0.00 N

ATOM 4364 H GLN 274 52.180 34.030 37.530 0.00 0.00 H

ATOM 4365 CA GLN 274 54.280 33.750 37.380 0.00 0.00 C

ATOM 4366 HA GLN 274 54.890 33.640 38.280 0.00 0.00 H

ATOM 4367 CB GLN 274 53.950 32.510 36.620 0.00 0.00 C

ATOM 4368 HB1 GLN 274 53.830 32.820 35.590 0.00 0.00 H

ATOM 4369 HB2 GLN 274 53.140 32.010 37.150 0.00 0.00 H

ATOM 4370 CG GLN 274 55.060 31.450 36.580 0.00 0.00 C

ATOM 4371 HG1 GLN 274 55.300 31.110 37.580 0.00 0.00 H

ATOM 4372 HG2 GLN 274 55.960 31.890 36.160 0.00 0.00 H

ATOM 4373 CD GLN 274 54.760 30.180 35.760 0.00 0.00 C

ATOM 4374 OE1 GLN 274 53.770 30.000 35.090 0.00 0.00 O

ATOM 4375 NE2 GLN 274 55.650 29.210 35.730 0.00 0.00 N

ATOM 4376 HE21 GLN 274 56.470 29.200 36.320 0.00 0.00 H

ATOM 4377 HE22 GLN 274 55.250 28.400 35.260 0.00 0.00 H

ATOM 4378 C GLN 274 55.250 34.650 36.620 0.00 0.00 C

ATOM 4379 O GLN 274 56.330 34.920 37.180 0.00 0.00 O

ATOM 4380 N ASN 275 54.810 35.310 35.570 0.00 0.00 N

ATOM 4381 H ASN 275 53.830 35.220 35.360 0.00 0.00 H

ATOM 4382 CA ASN 275 55.620 36.160 34.750 0.00 0.00 C

ATOM 4383 HA ASN 275 56.480 35.620 34.330 0.00 0.00 H

ATOM 4384 CB ASN 275 54.820 36.650 33.550 0.00 0.00 C

ATOM 4385 HB1 ASN 275 54.190 37.460 33.930 0.00 0.00 H

ATOM 4386 HB2 ASN 275 54.150 35.920 33.100 0.00 0.00 H

ATOM 4387 CG ASN 275 55.660 37.220 32.360 0.00 0.00 C

ATOM 4388 OD1 ASN 275 56.900 37.290 32.480 0.00 0.00 O

ATOM 4389 ND2 ASN 275 55.070 37.670 31.280 0.00 0.00 N

ATOM 4390 HD21 ASN 275 54.060 37.590 31.300 0.00 0.00 H

ATOM 4391 HD22 ASN 275 55.650 37.950 30.510 0.00 0.00 H

ATOM 4392 C ASN 275 56.130 37.420 35.480 0.00 0.00 C

ATOM 4393 O ASN 275 57.280 37.820 35.470 0.00 0.00 O

ATOM 4394 N ALA 276 55.220 37.970 36.310 0.00 0.00 N

ATOM 4395 H ALA 276 54.310 37.550 36.230 0.00 0.00 H

ATOM 4396 CA ALA 276 55.490 39.120 37.130 0.00 0.00 C

ATOM 4397 HA ALA 276 55.980 39.900 36.550 0.00 0.00 H

ATOM 4398 CB ALA 276 54.050 39.530 37.650 0.00 0.00 C

ATOM 4399 HB1 ALA 276 54.120 40.470 38.210 0.00 0.00 H

ATOM 4400 HB2 ALA 276 53.340 39.630 36.840 0.00 0.00 H

ATOM 4401 HB3 ALA 276 53.730 38.730 38.320 0.00 0.00 H

ATOM 4402 C ALA 276 56.470 38.770 38.290 0.00 0.00 C

ATOM 4403 O ALA 276 57.380 39.540 38.460 0.00 0.00 O

ATOM 4404 N PHE 277 56.310 37.700 39.060 0.00 0.00 N

ATOM 4405 H PHE 277 55.740 36.960 38.670 0.00 0.00 H

ATOM 4406 CA PHE 277 57.220 37.260 40.120 0.00 0.00 C

ATOM 4407 HA PHE 277 57.330 38.060 40.860 0.00 0.00 H

ATOM 4408 CB PHE 277 56.710 35.990 40.740 0.00 0.00 C

ATOM 4409 HB1 PHE 277 57.450 35.500 41.370 0.00 0.00 H

ATOM 4410 HB2 PHE 277 56.410 35.270 39.970 0.00 0.00 H

ATOM 4411 CG PHE 277 55.480 36.180 41.620 0.00 0.00 C

ATOM 4412 CD1 PHE 277 55.040 35.000 42.280 0.00 0.00 C

ATOM 4413 HD1 PHE 277 55.480 34.050 42.020 0.00 0.00 H

ATOM 4414 CE1 PHE 277 54.130 35.050 43.320 0.00 0.00 C

ATOM 4415 HE1 PHE 277 53.980 34.150 43.900 0.00 0.00 H

ATOM 4416 CZ PHE 277 53.570 36.260 43.650 0.00 0.00 C

ATOM 4417 HZ PHE 277 52.840 36.180 44.450 0.00 0.00 H

ATOM 4418 CE2 PHE 277 53.990 37.460 43.080 0.00 0.00 C

ATOM 4419 HE2 PHE 277 53.650 38.410 43.470 0.00 0.00 H

ATOM 4420 CD2 PHE 277 54.940 37.390 42.050 0.00 0.00 C

ATOM 4421 HD2 PHE 277 55.280 38.360 41.720 0.00 0.00 H

ATOM 4422 C PHE 277 58.600 36.890 39.530 0.00 0.00 C

ATOM 4423 O PHE 277 59.620 37.150 40.150 0.00 0.00 O

ATOM 4424 N LYS 278 58.740 36.280 38.320 0.00 0.00 N

ATOM 4425 H LYS 278 57.860 35.950 37.970 0.00 0.00 H

ATOM 4426 CA LYS 278 59.940 36.020 37.570 0.00 0.00 C

ATOM 4427 HA LYS 278 60.630 35.400 38.140 0.00 0.00 H

ATOM 4428 CB LYS 278 59.520 35.140 36.380 0.00 0.00 C

ATOM 4429 HB1 LYS 278 58.910 35.710 35.670 0.00 0.00 H

ATOM 4430 HB2 LYS 278 59.020 34.230 36.720 0.00 0.00 H

ATOM 4431 CG LYS 278 60.800 34.620 35.650 0.00 0.00 C

ATOM 4432 HG1 LYS 278 61.450 35.480 35.490 0.00 0.00 H

ATOM 4433 HG2 LYS 278 60.510 34.320 34.640 0.00 0.00 H

ATOM 4434 CD LYS 278 61.550 33.610 36.550 0.00 0.00 C

ATOM 4435 HD1 LYS 278 61.100 32.620 36.650 0.00 0.00 H

ATOM 4436 HD2 LYS 278 61.840 33.970 37.540 0.00 0.00 H

ATOM 4437 CE LYS 278 62.840 33.420 35.850 0.00 0.00 C

ATOM 4438 HE1 LYS 278 63.240 34.400 35.590 0.00 0.00 H

ATOM 4439 HE2 LYS 278 62.690 32.920 34.890 0.00 0.00 H

ATOM 4440 NZ LYS 278 63.890 32.740 36.710 0.00 0.00 N1+

ATOM 4441 HZ1 LYS 278 64.700 32.560 36.140 0.00 0.00 H

ATOM 4442 HZ2 LYS 278 64.020 33.290 37.540 0.00 0.00 H

ATOM 4443 HZ3 LYS 278 63.500 31.910 37.140 0.00 0.00 H

ATOM 4444 C LYS 278 60.690 37.300 37.180 0.00 0.00 C

ATOM 4445 O LYS 278 61.950 37.410 37.460 0.00 0.00 O

ATOM 4446 N LYS 279 60.010 38.320 36.710 0.00 0.00 N

ATOM 4447 H LYS 279 59.040 38.200 36.440 0.00 0.00 H

ATOM 4448 CA LYS 279 60.570 39.640 36.390 0.00 0.00 C

ATOM 4449 HA LYS 279 61.540 39.540 35.880 0.00 0.00 H

ATOM 4450 CB LYS 279 59.690 40.430 35.420 0.00 0.00 C

ATOM 4451 HB1 LYS 279 60.160 41.410 35.330 0.00 0.00 H

ATOM 4452 HB2 LYS 279 58.730 40.490 35.920 0.00 0.00 H

ATOM 4453 CG LYS 279 59.700 39.720 34.050 0.00 0.00 C

ATOM 4454 HG1 LYS 279 59.390 38.680 34.130 0.00 0.00 H

ATOM 4455 HG2 LYS 279 60.640 39.710 33.500 0.00 0.00 H

ATOM 4456 CD LYS 279 58.600 40.250 33.150 0.00 0.00 C

ATOM 4457 HD1 LYS 279 58.840 39.820 32.180 0.00 0.00 H

ATOM 4458 HD2 LYS 279 58.740 41.320 32.970 0.00 0.00 H

ATOM 4459 CE LYS 279 57.110 40.130 33.540 0.00 0.00 C

ATOM 4460 HE1 LYS 279 56.980 40.750 34.440 0.00 0.00 H

ATOM 4461 HE2 LYS 279 56.910 39.160 34.000 0.00 0.00 H

ATOM 4462 NZ LYS 279 56.130 40.530 32.570 0.00 0.00 N1+

ATOM 4463 HZ1 LYS 279 56.150 41.530 32.440 0.00 0.00 H

ATOM 4464 HZ2 LYS 279 56.290 40.140 31.650 0.00 0.00 H

ATOM 4465 HZ3 LYS 279 55.200 40.260 32.860 0.00 0.00 H

ATOM 4466 C LYS 279 61.000 40.480 37.630 0.00 0.00 C

ATOM 4467 O LYS 279 62.030 41.120 37.640 0.00 0.00 O

ATOM 4468 N ILE 280 60.180 40.280 38.720 0.00 0.00 N

ATOM 4469 H ILE 280 59.280 39.830 38.670 0.00 0.00 H

ATOM 4470 CA ILE 280 60.590 40.960 40.070 0.00 0.00 C

ATOM 4471 HA ILE 280 60.750 42.030 39.930 0.00 0.00 H

ATOM 4472 CB ILE 280 59.520 40.710 41.140 0.00 0.00 C

ATOM 4473 HB ILE 280 59.140 39.700 40.960 0.00 0.00 H

ATOM 4474 CG2 ILE 280 59.850 40.880 42.620 0.00 0.00 C

ATOM 4475 HG21 ILE 280 60.390 41.820 42.740 0.00 0.00 H

ATOM 4476 HG22 ILE 280 58.920 41.050 43.160 0.00 0.00 H

ATOM 4477 HG23 ILE 280 60.500 40.060 42.920 0.00 0.00 H

ATOM 4478 CG1 ILE 280 58.400 41.680 40.790 0.00 0.00 C

ATOM 4479 HG11 ILE 280 58.170 41.700 39.730 0.00 0.00 H

ATOM 4480 HG12 ILE 280 58.750 42.650 41.160 0.00 0.00 H

ATOM 4481 CD ILE 280 57.040 41.500 41.450 0.00 0.00 C

ATOM 4482 HD1 ILE 280 56.290 42.030 40.860 0.00 0.00 H

ATOM 4483 HD2 ILE 280 56.790 40.430 41.480 0.00 0.00 H

ATOM 4484 HD3 ILE 280 56.970 41.700 42.520 0.00 0.00 H

ATOM 4485 C ILE 280 61.980 40.470 40.650 0.00 0.00 C

ATOM 4486 O ILE 280 62.900 41.230 40.920 0.00 0.00 O

ATOM 4487 N ILE 281 62.190 39.120 40.630 0.00 0.00 N

ATOM 4488 H ILE 281 61.390 38.510 40.470 0.00 0.00 H

ATOM 4489 CA ILE 281 63.360 38.590 41.410 0.00 0.00 C

ATOM 4490 HA ILE 281 63.600 39.140 42.320 0.00 0.00 H

ATOM 4491 CB ILE 281 63.090 37.180 41.980 0.00 0.00 C

ATOM 4492 HB ILE 281 63.860 36.770 42.620 0.00 0.00 H

ATOM 4493 CG2 ILE 281 61.850 37.210 42.890 0.00 0.00 C

ATOM 4494 HG21 ILE 281 60.990 37.530 42.310 0.00 0.00 H

ATOM 4495 HG22 ILE 281 61.850 36.250 43.400 0.00 0.00 H

ATOM 4496 HG23 ILE 281 62.010 38.090 43.510 0.00 0.00 H

ATOM 4497 CG1 ILE 281 63.020 36.110 40.910 0.00 0.00 C

ATOM 4498 HG11 ILE 281 64.070 35.950 40.660 0.00 0.00 H

ATOM 4499 HG12 ILE 281 62.550 36.550 40.030 0.00 0.00 H

ATOM 4500 CD ILE 281 62.310 34.820 41.190 0.00 0.00 C

ATOM 4501 HD1 ILE 281 61.240 34.980 41.350 0.00 0.00 H

ATOM 4502 HD2 ILE 281 62.390 34.160 40.330 0.00 0.00 H

ATOM 4503 HD3 ILE 281 62.760 34.360 42.070 0.00 0.00 H

ATOM 4504 C ILE 281 64.710 38.520 40.620 0.00 0.00 C

ATOM 4505 O ILE 281 65.770 38.170 41.220 0.00 0.00 O

ATOM 4506 N LYS 282 64.680 38.750 39.270 0.00 0.00 N

ATOM 4507 H LYS 282 63.790 38.920 38.810 0.00 0.00 H

ATOM 4508 CA LYS 282 65.840 38.580 38.390 0.00 0.00 C

ATOM 4509 HA LYS 282 66.150 37.540 38.420 0.00 0.00 H

ATOM 4510 CB LYS 282 65.500 39.010 36.890 0.00 0.00 C

ATOM 4511 HB1 LYS 282 65.620 40.090 36.850 0.00 0.00 H

ATOM 4512 HB2 LYS 282 64.440 38.830 36.750 0.00 0.00 H

ATOM 4513 CG LYS 282 66.340 38.170 35.850 0.00 0.00 C

ATOM 4514 HG1 LYS 282 66.410 37.150 36.220 0.00 0.00 H

ATOM 4515 HG2 LYS 282 67.340 38.600 35.810 0.00 0.00 H

ATOM 4516 CD LYS 282 65.760 38.350 34.470 0.00 0.00 C

ATOM 4517 HD1 LYS 282 66.090 39.290 34.040 0.00 0.00 H

ATOM 4518 HD2 LYS 282 64.680 38.380 34.610 0.00 0.00 H

ATOM 4519 CE LYS 282 66.190 37.160 33.610 0.00 0.00 C

ATOM 4520 HE1 LYS 282 65.500 37.000 32.780 0.00 0.00 H

ATOM 4521 HE2 LYS 282 66.090 36.220 34.160 0.00 0.00 H

ATOM 4522 NZ LYS 282 67.570 37.320 33.060 0.00 0.00 N1+

ATOM 4523 HZ1 LYS 282 68.250 37.370 33.810 0.00 0.00 H

ATOM 4524 HZ2 LYS 282 67.870 36.490 32.570 0.00 0.00 H

ATOM 4525 HZ3 LYS 282 67.630 38.140 32.480 0.00 0.00 H

ATOM 4526 C LYS 282 67.180 39.120 38.760 0.00 0.00 C

ATOM 4527 O LYS 282 68.240 38.390 38.680 0.00 0.00 O

ATOM 4528 N CYS 283 67.180 40.320 39.230 0.00 0.00 N

ATOM 4529 H CYS 283 66.300 40.820 39.290 0.00 0.00 H

ATOM 4530 CA CYS 283 68.380 41.020 39.580 0.00 0.00 C

ATOM 4531 HA CYS 283 69.150 40.580 38.940 0.00 0.00 H

ATOM 4532 CB CYS 283 68.300 42.510 39.340 0.00 0.00 C

ATOM 4533 HB1 CYS 283 69.200 42.930 39.800 0.00 0.00 H

ATOM 4534 HB2 CYS 283 67.500 42.880 39.980 0.00 0.00 H

ATOM 4535 SG CYS 283 68.120 42.970 37.510 0.00 0.00 S

ATOM 4536 HG CYS 283 68.070 44.310 37.540 0.00 0.00 H

ATOM 4537 C CYS 283 68.820 40.610 40.990 0.00 0.00 C

ATOM 4538 O CYS 283 69.930 40.890 41.410 0.00 0.00 O

TER

ATOM 4539 N NME 283 68.030 39.840 41.810 0.00 0.00 N

ATOM 4540 H NME 283 67.060 39.700 41.580 0.00 0.00 H

ATOM 4541 CH3 NME 283 68.370 39.340 43.100 0.00 0.00 C

ATOM 4542 HH31 NME 283 68.760 40.160 43.700 0.00 0.00 H

ATOM 4543 HH32 NME 283 67.440 38.910 43.470 0.00 0.00 H

ATOM 4544 HH33 NME 283 69.070 38.510 43.040 0.00 0.00 H

ATOM 4545 O1 ERG 284 46.990 33.130 70.790 0.00 0.00 O

ATOM 4546 O2 ERG 284 48.250 31.180 70.500 0.00 0.00 O

ATOM 4547 O3 ERG 284 47.680 29.670 74.860 0.00 0.00 O

ATOM 4548 O4 ERG 284 44.040 31.610 71.980 0.00 0.00 O

ATOM 4549 O ERG 284 44.890 32.360 68.680 0.00 0.00 O

ATOM 4550 N1 ERG 284 48.530 31.290 73.580 0.00 0.00 N

ATOM 4551 N2 ERG 284 46.320 31.250 71.870 0.00 0.00 N

ATOM 4552 N3 ERG 284 45.010 34.040 70.070 0.00 0.00 N

ATOM 4553 N4 ERG 284 38.100 36.780 65.000 0.00 0.00 N

ATOM 4554 C1 ERG 284 48.450 32.360 72.590 0.00 0.00 C

ATOM 4555 C2 ERG 284 47.520 31.970 71.420 0.00 0.00 C

ATOM 4556 C3 ERG 284 49.890 32.680 72.310 0.00 0.00 C

ATOM 4557 C4 ERG 284 46.420 30.190 72.890 0.00 0.00 C

ATOM 4558 C5 ERG 284 49.870 31.230 74.190 0.00 0.00 C

ATOM 4559 C6 ERG 284 50.640 32.410 73.650 0.00 0.00 C

ATOM 4560 C7 ERG 284 45.640 33.410 71.240 0.00 0.00 C

ATOM 4561 C8 ERG 284 47.600 30.360 73.850 0.00 0.00 C

ATOM 4562 C9 ERG 284 45.230 31.980 71.700 0.00 0.00 C

ATOM 4563 C10 ERG 284 46.470 28.790 72.230 0.00 0.00 C

ATOM 4564 C11 ERG 284 45.730 34.360 72.530 0.00 0.00 C

ATOM 4565 C12 ERG 284 44.170 34.610 67.940 0.00 0.00 C

ATOM 4566 C13 ERG 284 42.320 35.140 65.650 0.00 0.00 C

ATOM 4567 C14 ERG 284 44.700 33.540 68.860 0.00 0.00 C

ATOM 4568 C15 ERG 284 45.190 28.350 71.570 0.00 0.00 C

ATOM 4569 C16 ERG 284 44.510 34.250 66.510 0.00 0.00 C

ATOM 4570 C17 ERG 284 41.790 35.220 67.140 0.00 0.00 C

ATOM 4571 C18 ERG 284 41.720 36.290 64.810 0.00 0.00 C

ATOM 4572 C19 ERG 284 42.650 34.890 68.080 0.00 0.00 C

ATOM 4573 C20 ERG 284 40.300 35.410 67.310 0.00 0.00 C

ATOM 4574 C21 ERG 284 40.210 36.220 64.970 0.00 0.00 C

ATOM 4575 C22 ERG 284 39.610 35.810 66.220 0.00 0.00 C

ATOM 4576 C23 ERG 284 44.140 27.790 72.370 0.00 0.00 C

ATOM 4577 C24 ERG 284 44.930 28.710 70.240 0.00 0.00 C

ATOM 4578 C25 ERG 284 38.360 36.210 66.190 0.00 0.00 C

ATOM 4579 C26 ERG 284 39.200 36.800 64.220 0.00 0.00 C

ATOM 4580 C27 ERG 284 39.460 35.140 68.500 0.00 0.00 C

ATOM 4581 C28 ERG 284 37.570 36.180 67.330 0.00 0.00 C

ATOM 4582 C29 ERG 284 42.910 27.590 71.790 0.00 0.00 C

ATOM 4583 C30 ERG 284 43.730 28.390 69.590 0.00 0.00 C

ATOM 4584 C31 ERG 284 38.130 35.580 68.410 0.00 0.00 C

ATOM 4585 C32 ERG 284 42.720 27.910 70.380 0.00 0.00 C

ATOM 4586 H1 ERG 284 47.940 33.180 73.120 0.00 0.00 H

ATOM 4587 H2 ERG 284 49.980 33.720 71.990 0.00 0.00 H

ATOM 4588 H3 ERG 284 50.430 32.000 71.650 0.00 0.00 H

ATOM 4589 H4 ERG 284 45.630 30.320 73.640 0.00 0.00 H

ATOM 4590 H5 ERG 284 50.290 30.280 73.840 0.00 0.00 H

ATOM 4591 H6 ERG 284 49.830 31.210 75.280 0.00 0.00 H

ATOM 4592 H7 ERG 284 51.710 32.220 73.620 0.00 0.00 H

ATOM 4593 H8 ERG 284 50.520 33.280 74.290 0.00 0.00 H

ATOM 4594 H9 ERG 284 47.280 28.830 71.500 0.00 0.00 H

ATOM 4595 H10 ERG 284 46.750 28.090 73.010 0.00 0.00 H

ATOM 4596 H11 ERG 284 48.610 31.770 69.810 0.00 0.00 H

ATOM 4597 H12 ERG 284 44.750 35.010 70.130 0.00 0.00 H

ATOM 4598 H13 ERG 284 44.810 34.910 72.700 0.00 0.00 H

ATOM 4599 H14 ERG 284 45.820 33.660 73.360 0.00 0.00 H

ATOM 4600 H15 ERG 284 46.560 35.060 72.410 0.00 0.00 H

ATOM 4601 H16 ERG 284 44.600 35.520 68.370 0.00 0.00 H

ATOM 4602 H17 ERG 284 41.850 34.260 65.190 0.00 0.00 H

ATOM 4603 H18 ERG 284 45.590 34.350 66.370 0.00 0.00 H

ATOM 4604 H19 ERG 284 44.300 33.180 66.440 0.00 0.00 H

ATOM 4605 H20 ERG 284 41.950 36.210 63.740 0.00 0.00 H

ATOM 4606 H21 ERG 284 42.120 37.230 65.190 0.00 0.00 H

ATOM 4607 H22 ERG 284 42.250 34.780 69.090 0.00 0.00 H

ATOM 4608 H23 ERG 284 44.360 27.600 73.420 0.00 0.00 H

ATOM 4609 H24 ERG 284 45.660 29.290 69.680 0.00 0.00 H

ATOM 4610 H25 ERG 284 39.300 37.390 63.320 0.00 0.00 H

ATOM 4611 H26 ERG 284 39.920 34.780 69.410 0.00 0.00 H

ATOM 4612 H27 ERG 284 37.180 37.050 64.690 0.00 0.00 H

ATOM 4613 H28 ERG 284 36.520 36.440 67.380 0.00 0.00 H

ATOM 4614 H29 ERG 284 42.230 26.960 72.370 0.00 0.00 H

ATOM 4615 H30 ERG 284 43.440 28.740 68.600 0.00 0.00 H

ATOM 4616 H31 ERG 284 37.500 35.380 69.280 0.00 0.00 H

ATOM 4617 H32 ERG 284 41.750 27.780 69.920 0.00 0.00 H

ATOM 4618 N ERG 284 43.810 35.060 65.470 0.00 0.00 N

ATOM 4619 H33 ERG 284 44.190 36.000 65.650 0.00 0.00 H

ATOM 4620 C ERG 284 44.220 34.540 64.160 0.00 0.00 C

ATOM 4621 H34 ERG 284 45.300 34.400 64.030 0.00 0.00 H

ATOM 4622 H35 ERG 284 44.000 35.310 63.430 0.00 0.00 H

ATOM 4623 H ERG 284 43.650 33.630 63.970 0.00 0.00 H

END

**Plumed-5ht1a.dat**

RESTART

WHOLEMOLECULES ENTITY0=9,23,37,44,63,74,86,102,116,132,143,164,181,197,216,230,241,260,279,298,305,319,338,357,377,388,398,414,433,440,454,464,475,491,507,517,527,546,556,575,590,614,625,644,661,675,691,701,715,736,755,774,781,792,811,821,837,851,863,882,899,915,926,942,961,977,1004,1010,1027,1037,1047,1066,1087,1104,1120,1139,1153,1175,1199,1213,1232,1239,1256,1272,1286,1296,1308,1327,1347,1366,1376,1395,1407,1423,1442,1453,1464,1478,1489,1500,1519,1538,1555,1574,1585,1595,1614,1624,1643,1655,1679,1700,1724,1734,1753,1767,1787,1793,1812,1824,1845,1861,1875,1897,1921,1943,1949,1973,1997,2007,2017,2027,2046,2065,2076,2095,2109,2133,2152,2171,2178,2198,2217,2236,2247,2274,2288,2294,2311,2330,2337,2361,2385,2407,2413,2428,2440,2464,2475,2495,2501,2513,2523,2533,2547,2566,2577,2599,2611,2628,2635,2656,2670,2689,2710,2721,2735,2755,2762,2772,2792,2813,2840,2846,2865,2884,2903,2920,2939,2955,2974,2995,3002,3026,3045,3065,3089,3099,3109,3133,3153,3177,3208,3227,3237,3261,3276,3300,3322,3336,3352,3374,3388,3407,3414,3433,3452,3469,3476,3490,3510,3529,3548,3559,3583,3610,3616,3636,3656,3675,3691,3701,3720,3736,3763,3769,3789,3800,3815,3826,3837,3848,3865,3890,3896,3910,3929,3948,3955,3965,3984,4003,4017,4041,4060,4067,4088,4099,4113,4124,4143,4162,4184,4190,4206,4225,4246,4256,4277,4297,4311,4333,4345,4365,4382,4396,4406,4426,4448,4470,4489,4508,4530

c1: COM ATOMS=4577,4578,4580,4582,4583,4584,4586,4587,4589

c2: COM ATOMS=4565,4568,4571,4574,4575,4596

h1: COM ATOMS=1272,1286,1296,1308

h2: COM ATOMS=1407,1423,1442,1453

a1: COM ATOMS=4545,4550,4547,4552,4554,4549

a2: COM ATOMS=4582,4583,4584,4586,4587,4589

ser98: COM ATOMS=1476,1477,1478,1479,1480,1481,1482,1483,1484,1485,1486

d1: DISTANCE ATOMS=ser98,4596

tor: TORSION ATOMS=c1,c2,h2,h1

ang: ANGLE ATOMS=a1,c2,a2

lig: COM ATOMS=4565,4568,4571,4574,4575,4596

fps: FUNNEL_PS LIGAND=lig REFERENCE=../START.PDB ANCHOR=2533 POINTS=4.426,3.91,5.5,4.46,3.2,8.101

rmsd: RMSD REFERENCE=../START.PDB TYPE=OPTIMAL

FUNNEL ARG=fps.lp,fps.ld ZCC=4.77 ALPHA=0.62 RCYL=0.3 MINS=-2.0 MAXS=12.0 KAPPA=35100 NBINS=2000 NBINZ=2000 SAFETY=1.0 SLOPE=0.8 FILE=../BIAS LABEL=funnel WALKERS_MPI

METAD ARG=d1,tor,ang SIGMA=0.3,0.4,0.4 HEIGHT=0.15 PACE=500 TEMP=309.75 BIASFACTOR=150 CALC_RCT RCT_USTRIDE=10 LABEL=metad GRID_MIN=0.0,-pi,0 GRID_MAX=10.0,pi,pi GRID_RFILE=../grid_r.dat GRID_WFILE=../grid_w.dat GRID_WSTRIDE=250000 WALKERS_MPI FILE=../HILLS

LOWER_WALLS ARG=fps.lp AT=0.6 KAPPA=500000 EXP=2 OFFSET=0 LABEL=lwall

UPPER_WALLS ARG=rmsd AT=0.1 KAPPA=500000 EXP=2 OFFSET=0 LABEL=uwall-rmsd

UPPER_WALLS ARG=fps.lp AT=7.8 KAPPA=500000 EXP=2 OFFSET=0 LABEL=uwall

PRINT STRIDE=500 ARG=* FILE=COLVAR

**Plumed-5ht2a.dat**

RESTART

WHOLEMOLECULES ENTITY0=7,8,9,10,11,12,13,14,15,16,17,18,19,20,21,22,23,24,25,26,27,28,29,30,31,32,33,34,35,36,37,38,39,40,41,42,43,44,45,46,47,48,49,50,51,52,53,54,55,56,57,58,59,60,61,62,63,64,65,66,67,68,69,70,71,72,73,74,75,76,77,78,79,80,81,82,83,84,85,86,87,88,89,90,91,92,93,94,95,96,97,98,99,100,101,102,103,104,105,106,107,108,109,110,111,112,113,114,115,116,117,118,119,120,121,122,123,124,125,126,127,128,129,130,131,132,133,134,135,136,137,138,139,140,141,142,143,144,145,146,147,148,149,150,151,152,153,154,155,156,157,158,159,160,161,162,163,164,165,166,167,168,169,170,171,172,173,174,175,176,177,178,179,180,181,182,183,184,185,186,187,188,189,190,191,192,193,194,195,196,197,198,199,200,201,202,203,204,205,206,207,208,209,210,211,212,213,214,215,216,217,218,219,220,221,222,223,224,225,226,227,228,229,230,231,232,233,234,235,236,237,238,239,240,241,242,243,244,245,246,247,248,249,250,251,252,253,254,255,256,257,258,259,260,261,262,263,264,265,266,267,268,269,270,271,272,273,274,275,276,277,278,279,280,281,282,283,284,285,286,287,288,289,290,291,292,293,294,295,296,297,298,299,300,301,302,303,304,305,306,307,308,309,310,311,312,313,314,315,316,317,318,319,320,321,322,323,324,325,326,327,328,329,330,331,332,333,334,335,336,337,338,339,340,341,342,343,344,345,346,347,348,349,350,351,352,353,354,355,356,357,358,359,360,361,362,363,364,365,366,367,368,369,370,371,372,373,374,375,376,377,378,379,380,381,382,383,384,385,386,387,388,389,390,391,392,393,394,395,396,397,398,399,400,401,402,403,404,405,406,407,408,409,410,411,412,413,414,415,416,417,418,419,420,421,422,423,424,425,426,427,428,429,430,431,432,433,434,435,436,437,438,439,440,441,442,443,444,445,446,447,448,449,450,451,452,453,454,455,456,457,458,459,460,461,462,463,464,465,466,467,468,469,470,471,472,473,474,475,476,477,478,479,480,481,482,483,484,485,486,487,488,489,490,491,492,493,494,495,496,497,498,499,500,501,502,503,504,505,506,507,508,509,510,511,512,513,514,515,516,517,518,519,520,521,522,523,524,525,526,527,528,529,530,531,532,533,534,535,536,537,538,539,540,541,542,543,544,545,546,547,548,549,550,551,552,553,554,555,556,557,558,559,560,561,562,563,564,565,566,567,568,569,570,571,572,573,574,575,576,577,578,579,580,581,582,583,584,585,586,587,588,589,590,591,592,593,594,595,596,597,598,599,600,601,602,603,604,605,606,607,608,609,610,611,612,613,614,615,616,617,618,619,620,621,622,623,624,625,626,627,628,629,630,631,632,633,634,635,636,637,638,639,640,641,642,643,644,645,646,647,648,649,650,651,652,653,654,655,656,657,658,659,660,661,662,663,664,665,666,667,668,669,670,671,672,673,674,675,676,677,678,679,680,681,682,683,684,685,686,687,688,689,690,691,692,693,694,695,696,697,698,699,700,701,702,703,704,705,706,707,708,709,710,711,712,713,714,715,716,717,718,719,720,721,722,723,724,725,726,727,728,729,730,731,732,733,734,735,736,737,738,739,740,741,742,743,744,745,746,747,748,749,750,751,752,753,754,755,756,757,758,759,760,761,762,763,764,765,766,767,768,769,770,771,772,773,774,775,776,777,778,779,780,781,782,783,784,785,786,787,788,789,790,791,792,793,794,795,796,797,798,799,800,801,802,803,804,805,806,807,808,809,810,811,812,813,814,815,816,817,818,819,820,821,822,823,824,825,826,827,828,829,830,831,832,833,834,835,836,837,838,839,840,841,842,843,844,845,846,847,848,849,850,851,852,853,854,855,856,857,858,859,860,861,862,863,864,865,866,867,868,869,870,871,872,873,874,875,876,877,878,879,880,881,882,883,884,885,886,887,888,889,890,891,892,893,894,895,896,897,898,899,900,901,902,903,904,905,906,907,908,909,910,911,912,913,914,915,916,917,918,919,920,921,922,923,924,925,926,927,928,929,930,931,932,933,934,935,936,937,938,939,940,941,942,943,944,945,946,947,948,949,950,951,952,953,954,955,956,957,958,959,960,961,962,963,964,965,966,967,968,969,970,971,972,973,974,975,976,977,978,979,980,981,982,983,984,985,986,987,988,989,990,991,992,993,994,995,996,997,998,999,1000,1001,1002,1003,1004,1005,1006,1007,1008,1009,1010,1011,1012,1013,1014,1015,1016,1017,1018,1019,1020,1021,1022,1023,1024,1025,1026,1027,1028,1029,1030,1031,1032,1033,1034,1035,1036,1037,1038,1039,1040,1041,1042,1043,1044,1045,1046,1047,1048,1049,1050,1051,1052,1053,1054,1055,1056,1057,1058,1059,1060,1061,1062,1063,1064,1065,1066,1067,1068,1069,1070,1071,1072,1073,1074,1075,1076,1077,1078,1079,1080,1081,1082,1083,1084,1085,1086,1087,1088,1089,1090,1091,1092,1093,1094,1095,1096,1097,1098,1099,1100,1101,1102,1103,1104,1105,1106,1107,1108,1109,1110,1111,1112,1113,1114,1115,1116,1117,1118,1119,1120,1121,1122,1123,1124,1125,1126,1127,1128,1129,1130,1131,1132,1133,1134,1135,1136,1137,1138,1139,1140,1141,1142,1143,1144,1145,1146,1147,1148,1149,1150,1151,1152,1153,1154,1155,1156,1157,1158,1159,1160,1161,1162,1163,1164,1165,1166,1167,1168,1169,1170,1171,1172,1173,1174,1175,1176,1177,1178,1179,1180,1181,1182,1183,1184,1185,1186,1187,1188,1189,1190,1191,1192,1193,1194,1195,1196,1197,1198,1199,1200,1201,1202,1203,1204,1205,1206,1207,1208,1209,1210,1211,1212,1213,1214,1215,1216,1217,1218,1219,1220,1221,1222,1223,1224,1225,1226,1227,1228,1229,1230,1231,1232,1233,1234,1235,1236,1237,1238,1239,1240,1241,1242,1243,1244,1245,1246,1247,1248,1249,1250,1251,1252,1253,1254,1255,1256,1257,1258,1259,1260,1261,1262,1263,1264,1265,1266,1267,1268,1269,1270,1271,1272,1273,1274,1275,1276,1277,1278,1279,1280,1281,1282,1283,1284,1285,1286,1287,1288,1289,1290,1291,1292,1293,1294,1295,1296,1297,1298,1299,1300,1301,1302,1303,1304,1305,1306,1307,1308,1309,1310,1311,1312,1313,1314,1315,1316,1317,1318,1319,1320,1321,1322,1323,1324,1325,1326,1327,1328,1329,1330,1331,1332,1333,1334,1335,1336,1337,1338,1339,1340,1341,1342,1343,1344,1345,1346,1347,1348,1349,1350,1351,1352,1353,1354,1355,1356,1357,1358,1359,1360,1361,1362,1363,1364,1365,1366,1367,1368,1369,1370,1371,1372,1373,1374,1375,1376,1377,1378,1379,1380,1381,1382,1383,1384,1385,1386,1387,1388,1389,1390,1391,1392,1393,1394,1395,1396,1397,1398,1399,1400,1401,1402,1403,1404,1405,1406,1407,1408,1409,1410,1411,1412,1413,1414,1415,1416,1417,1418,1419,1420,1421,1422,1423,1424,1425,1426,1427,1428,1429,1430,1431,1432,1433,1434,1435,1436,1437,1438,1439,1440,1441,1442,1443,1444,1445,1446,1447,1448,1449,1450,1451,1452,1453,1454,1455,1456,1457,1458,1459,1460,1461,1462,1463,1464,1465,1466,1467,1468,1469,1470,1471,1472,1473,1474,1475,1476,1477,1478,1479,1480,1481,1482,1483,1484,1485,1486,1487,1488,1489,1490,1491,1492,1493,1494,1495,1496,1497,1498,1499,1500,1501,1502,1503,1504,1505,1506,1507,1508,1509,1510,1511,1512,1513,1514,1515,1516,1517,1518,1519,1520,1521,1522,1523,1524,1525,1526,1527,1528,1529,1530,1531,1532,1533,1534,1535,1536,1537,1538,1539,1540,1541,1542,1543,1544,1545,1546,1547,1548,1549,1550,1551,1552,1553,1554,1555,1556,1557,1558,1559,1560,1561,1562,1563,1564,1565,1566,1567,1568,1569,1570,1571,1572,1573,1574,1575,1576,1577,1578,1579,1580,1581,1582,1583,1584,1585,1586,1587,1588,1589,1590,1591,1592,1593,1594,1595,1596,1597,1598,1599,1600,1601,1602,1603,1604,1605,1606,1607,1608,1609,1610,1611,1612,1613,1614,1615,1616,1617,1618,1619,1620,1621,1622,1623,1624,1625,1626,1627,1628,1629,1630,1631,1632,1633,1634,1635,1636,1637,1638,1639,1640,1641,1642,1643,1644,1645,1646,1647,1648,1649,1650,1651,1652,1653,1654,1655,1656,1657,1658,1659,1660,1661,1662,1663,1664,1665,1666,1667,1668,1669,1670,1671,1672,1673,1674,1675,1676,1677,1678,1679,1680,1681,1682,1683,1684,1685,1686,1687,1688,1689,1690,1691,1692,1693,1694,1695,1696,1697,1698,1699,1700,1701,1702,1703,1704,1705,1706,1707,1708,1709,1710,1711,1712,1713,1714,1715,1716,1717,1718,1719,1720,1721,1722,1723,1724,1725,1726,1727,1728,1729,1730,1731,1732,1733,1734,1735,1736,1737,1738,1739,1740,1741,1742,1743,1744,1745,1746,1747,1748,1749,1750,1751,1752,1753,1754,1755,1756,1757,1758,1759,1760,1761,1762,1763,1764,1765,1766,1767,1768,1769,1770,1771,1772,1773,1774,1775,1776,1777,1778,1779,1780,1781,1782,1783,1784,1785,1786,1787,1788,1789,1790,1791,1792,1793,1794,1795,1796,1797,1798,1799,1800,1801,1802,1803,1804,1805,1806,1807,1808,1809,1810,1811,1812,1813,1814,1815,1816,1817,1818,1819,1820,1821,1822,1823,1824,1825,1826,1827,1828,1829,1830,1831,1832,1833,1834,1835,1836,1837,1838,1839,1840,1841,1842,1843,1844,1845,1846,1847,1848,1849,1850,1851,1852,1853,1854,1855,1856,1857,1858,1859,1860,1861,1862,1863,1864,1865,1866,1867,1868,1869,1870,1871,1872,1873,1874,1875,1876,1877,1878,1879,1880,1881,1882,1883,1884,1885,1886,1887,1888,1889,1890,1891,1892,1893,1894,1895,1896,1897,1898,1899,1900,1901,1902,1903,1904,1905,1906,1907,1908,1909,1910,1911,1912,1913,1914,1915,1916,1917,1918,1919,1920,1921,1922,1923,1924,1925,1926,1927,1928,1929,1930,1931,1932,1933,1934,1935,1936,1937,1938,1939,1940,1941,1942,1943,1944,1945,1946,1947,1948,1949,1950,1951,1952,1953,1954,1955,1956,1957,1958,1959,1960,1961,1962,1963,1964,1965,1966,1967,1968,1969,1970,1971,1972,1973,1974,1975,1976,1977,1978,1979,1980,1981,1982,1983,1984,1985,1986,1987,1988,1989,1990,1991,1992,1993,1994,1995,1996,1997,1998,1999,2000,2001,2002,2003,2004,2005,2006,2007,2008,2009,2010,2011,2012,2013,2014,2015,2016,2017,2018,2019,2020,2021,2022,2023,2024,2025,2026,2027,2028,2029,2030,2031,2032,2033,2034,2035,2036,2037,2038,2039,2040,2041,2042,2043,2044,2045,2046,2047,2048,2049,2050,2051,2052,2053,2054,2055,2056,2057,2058,2059,2060,2061,2062,2063,2064,2065,2066,2067,2068,2069,2070,2071,2072,2073,2074,2075,2076,2077,2078,2079,2080,2081,2082,2083,2084,2085,2086,2087,2088,2089,2090,2091,2092,2093,2094,2095,2096,2097,2098,2099,2100,2101,2102,2103,2104,2105,2106,2107,2108,2109,2110,2111,2112,2113,2114,2115,2116,2117,2118,2119,2120,2121,2122,2123,2124,2125,2126,2127,2128,2129,2130,2131,2132,2133,2134,2135,2136,2137,2138,2139,2140,2141,2142,2143,2144,2145,2146,2147,2148,2149,2150,2151,2152,2153,2154,2155,2156,2157,2158,2159,2160,2161,2162,2163,2164,2165,2166,2167,2168,2169,2170,2171,2172,2173,2174,2175,2176,2177,2178,2179,2180,2181,2182,2183,2184,2185,2186,2187,2188,2189,2190,2191,2192,2193,2194,2195,2196,2197,2198,2199,2200,2201,2202,2203,2204,2205,2206,2207,2208,2209,2210,2211,2212,2213,2214,2215,2216,2217,2218,2219,2220,2221,2222,2223,2224,2225,2226,2227,2228,2229,2230,2231,2232,2233,2234,2235,2236,2237,2238,2239,2240,2241,2242,2243,2244,2245,2246,2247,2248,2249,2250,2251,2252,2253,2254,2255,2256,2257,2258,2259,2260,2261,2262,2263,2264,2265,2266,2267,2268,2269,2270,2271,2272,2273,2274,2275,2276,2277,2278,2279,2280,2281,2282,2283,2284,2285,2286,2287,2288,2289,2290,2291,2292,2293,2294,2295,2296,2297,2298,2299,2300,2301,2302,2303,2304,2305,2306,2307,2308,2309,2310,2311,2312,2313,2314,2315,2316,2317,2318,2319,2320,2321,2322,2323,2324,2325,2326,2327,2328,2329,2330,2331,2332,2333,2334,2335,2336,2337,2338,2339,2340,2341,2342,2343,2344,2345,2346,2347,2348,2349,2350,2351,2352,2353,2354,2355,2356,2357,2358,2359,2360,2361,2362,2363,2364,2365,2366,2367,2368,2369,2370,2371,2372,2373,2374,2375,2376,2377,2378,2379,2380,2381,2382,2383,2384,2385,2386,2387,2388,2389,2390,2391,2392,2393,2394,2395,2396,2397,2398,2399,2400,2401,2402,2403,2404,2405,2406,2407,2408,2409,2410,2411,2412,2413,2414,2415,2416,2417,2418,2419,2420,2421,2422,2423,2424,2425,2426,2427,2428,2429,2430,2431,2432,2433,2434,2435,2436,2437,2438,2439,2440,2441,2442,2443,2444,2445,2446,2447,2448,2449,2450,2451,2452,2453,2454,2455,2456,2457,2458,2459,2460,2461,2462,2463,2464,2465,2466,2467,2468,2469,2470,2471,2472,2473,2474,2475,2476,2477,2478,2479,2480,2481,2482,2483,2484,2485,2486,2487,2488,2489,2490,2491,2492,2493,2494,2495,2496,2497,2498,2499,2500,2501,2502,2503,2504,2505,2506,2507,2508,2509,2510,2511,2512,2513,2514,2515,2516,2517,2518,2519,2520,2521,2522,2523,2524,2525,2526,2527,2528,2529,2530,2531,2532,2533,2534,2535,2536,2537,2538,2539,2540,2541,2542,2543,2544,2545,2546,2547,2548,2549,2550,2551,2552,2553,2554,2555,2556,2557,2558,2559,2560,2561,2562,2563,2564,2565,2566,2567,2568,2569,2570,2571,2572,2573,2574,2575,2576,2577,2578,2579,2580,2581,2582,2583,2584,2585,2586,2587,2588,2589,2590,2591,2592,2593,2594,2595,2596,2597,2598,2599,2600,2601,2602,2603,2604,2605,2606,2607,2608,2609,2610,2611,2612,2613,2614,2615,2616,2617,2618,2619,2620,2621,2622,2623,2624,2625,2626,2627,2628,2629,2630,2631,2632,2633,2634,2635,2636,2637,2638,2639,2640,2641,2642,2643,2644,2645,2646,2647,2648,2649,2650,2651,2652,2653,2654,2655,2656,2657,2658,2659,2660,2661,2662,2663,2664,2665,2666,2667,2668,2669,2670,2671,2672,2673,2674,2675,2676,2677,2678,2679,2680,2681,2682,2683,2684,2685,2686,2687,2688,2689,2690,2691,2692,2693,2694,2695,2696,2697,2698,2699,2700,2701,2702,2703,2704,2705,2706,2707,2708,2709,2710,2711,2712,2713,2714,2715,2716,2717,2718,2719,2720,2721,2722,2723,2724,2725,2726,2727,2728,2729,2730,2731,2732,2733,2734,2735,2736,2737,2738,2739,2740,2741,2742,2743,2744,2745,2746,2747,2748,2749,2750,2751,2752,2753,2754,2755,2756,2757,2758,2759,2760,2761,2762,2763,2764,2765,2766,2767,2768,2769,2770,2771,2772,2773,2774,2775,2776,2777,2778,2779,2780,2781,2782,2783,2784,2785,2786,2787,2788,2789,2790,2791,2792,2793,2794,2795,2796,2797,2798,2799,2800,2801,2802,2803,2804,2805,2806,2807,2808,2809,2810,2811,2812,2813,2814,2815,2816,2817,2818,2819,2820,2821,2822,2823,2824,2825,2826,2827,2828,2829,2830,2831,2832,2833,2834,2835,2836,2837,2838,2839,2840,2841,2842,2843,2844,2845,2846,2847,2848,2849,2850,2851,2852,2853,2854,2855,2856,2857,2858,2859,2860,2861,2862,2863,2864,2865,2866,2867,2868,2869,2870,2871,2872,2873,2874,2875,2876,2877,2878,2879,2880,2881,2882,2883,2884,2885,2886,2887,2888,2889,2890,2891,2892,2893,2894,2895,2896,2897,2898,2899,2900,2901,2902,2903,2904,2905,2906,2907,2908,2909,2910,2911,2912,2913,2914,2915,2916,2917,2918,2919,2920,2921,2922,2923,2924,2925,2926,2927,2928,2929,2930,2931,2932,2933,2934,2935,2936,2937,2938,2939,2940,2941,2942,2943,2944,2945,2946,2947,2948,2949,2950,2951,2952,2953,2954,2955,2956,2957,2958,2959,2960,2961,2962,2963,2964,2965,2966,2967,2968,2969,2970,2971,2972,2973,2974,2975,2976,2977,2978,2979,2980,2981,2982,2983,2984,2985,2986,2987,2988,2989,2990,2991,2992,2993,2994,2995,2996,2997,2998,2999,3000,3001,3002,3003,3004,3005,3006,3007,3008,3009,3010,3011,3012,3013,3014,3015,3016,3017,3018,3019,3020,3021,3022,3023,3024,3025,3026,3027,3028,3029,3030,3031,3032,3033,3034,3035,3036,3037,3038,3039,3040,3041,3042,3043,3044,3045,3046,3047,3048,3049,3050,3051,3052,3053,3054,3055,3056,3057,3058,3059,3060,3061,3062,3063,3064,3065,3066,3067,3068,3069,3070,3071,3072,3073,3074,3075,3076,3077,3078,3079,3080,3081,3082,3083,3084,3085,3086,3087,3088,3089,3090,3091,3092,3093,3094,3095,3096,3097,3098,3099,3100,3101,3102,3103,3104,3105,3106,3107,3108,3109,3110,3111,3112,3113,3114,3115,3116,3117,3118,3119,3120,3121,3122,3123,3124,3125,3126,3127,3128,3129,3130,3131,3132,3133,3134,3135,3136,3137,3138,3139,3140,3141,3142,3143,3144,3145,3146,3147,3148,3149,3150,3151,3152,3153,3154,3155,3156,3157,3158,3159,3160,3161,3162,3163,3164,3165,3166,3167,3168,3169,3170,3171,3172,3173,3174,3175,3176,3177,3178,3179,3180,3181,3182,3183,3184,3185,3186,3187,3188,3189,3190,3203,3204,3205,3206,3207,3208,3209,3210,3211,3212,3213,3214,3215,3216,3217,3218,3219,3220,3221,3222,3223,3224,3225,3226,3227,3228,3229,3230,3231,3232,3233,3234,3235,3236,3237,3238,3239,3240,3241,3242,3243,3244,3245,3246,3247,3248,3249,3250,3251,3252,3253,3254,3255,3256,3257,3258,3259,3260,3261,3262,3263,3264,3265,3266,3267,3268,3269,3270,3271,3272,3273,3274,3275,3276,3277,3278,3279,3280,3281,3282,3283,3284,3285,3286,3287,3288,3289,3290,3291,3292,3293,3294,3295,3296,3297,3298,3299,3300,3301,3302,3303,3304,3305,3306,3307,3308,3309,3310,3311,3312,3313,3314,3315,3316,3317,3318,3319,3320,3321,3322,3323,3324,3325,3326,3327,3328,3329,3330,3331,3332,3333,3334,3335,3336,3337,3338,3339,3340,3341,3342,3343,3344,3345,3346,3347,3348,3349,3350,3351,3352,3353,3354,3355,3356,3357,3358,3359,3360,3361,3362,3363,3364,3365,3366,3367,3368,3369,3370,3371,3372,3373,3374,3375,3376,3377,3378,3379,3380,3381,3382,3383,3384,3385,3386,3387,3388,3389,3390,3391,3392,3393,3394,3395,3396,3397,3398,3399,3400,3401,3402,3403,3404,3405,3406,3407,3408,3409,3410,3411,3412,3413,3414,3415,3416,3417,3418,3419,3420,3421,3422,3423,3424,3425,3426,3427,3428,3429,3430,3431,3432,3433,3434,3435,3436,3437,3438,3439,3440,3441,3442,3443,3444,3445,3446,3447,3448,3449,3450,3451,3452,3453,3454,3455,3456,3457,3458,3459,3460,3461,3462,3463,3464,3465,3466,3467,3468,3469,3470,3471,3472,3473,3474,3475,3476,3477,3478,3479,3480,3481,3482,3483,3484,3485,3486,3487,3488,3489,3490,3491,3492,3493,3494,3495,3496,3497,3498,3499,3500,3501,3502,3503,3504,3505,3506,3507,3508,3509,3510,3511,3512,3513,3514,3515,3516,3517,3518,3519,3520,3521,3522,3523,3524,3525,3526,3527,3528,3529,3530,3531,3532,3533,3534,3535,3536,3537,3538,3539,3540,3541,3542,3543,3544,3545,3546,3547,3548,3549,3550,3551,3552,3553,3554,3555,3556,3557,3558,3559,3560,3561,3562,3563,3564,3565,3566,3567,3568,3569,3570,3571,3572,3573,3574,3575,3576,3577,3578,3579,3580,3581,3582,3583,3584,3585,3586,3587,3588,3589,3590,3591,3592,3593,3594,3595,3596,3597,3598,3599,3600,3601,3602,3603,3604,3605,3606,3607,3608,3609,3610,3611,3612,3613,3614,3615,3616,3617,3618,3619,3620,3621,3622,3623,3624,3625,3626,3627,3628,3629,3630,3631,3632,3633,3634,3635,3636,3637,3638,3639,3640,3641,3642,3643,3644,3645,3646,3647,3648,3649,3650,3651,3652,3653,3654,3655,3656,3657,3658,3659,3660,3661,3662,3663,3664,3665,3666,3667,3668,3669,3670,3671,3672,3673,3674,3675,3676,3677,3678,3679,3680,3681,3682,3683,3684,3685,3686,3687,3688,3689,3690,3691,3692,3693,3694,3695,3696,3697,3698,3699,3700,3701,3702,3703,3704,3705,3706,3707,3708,3709,3710,3711,3712,3713,3714,3715,3716,3717,3718,3719,3720,3721,3722,3723,3724,3725,3726,3727,3728,3729,3730,3731,3732,3733,3734,3735,3736,3737,3738,3739,3740,3741,3742,3743,3744,3745,3746,3747,3748,3749,3750,3751,3752,3753,3754,3755,3756,3757,3758,3759,3760,3761,3762,3763,3764,3765,3766,3767,3768,3769,3770,3771,3772,3773,3774,3775,3776,3777,3778,3779,3780,3781,3782,3783,3784,3785,3786,3787,3788,3789,3790,3791,3792,3793,3794,3795,3796,3797,3798,3799,3800,3801,3802,3803,3804,3805,3806,3807,3808,3809,3810,3811,3812,3813,3814,3815,3816,3817,3818,3819,3820,3821,3822,3823,3824,3825,3826,3827,3828,3829,3830,3831,3832,3833,3834,3835,3836,3837,3838,3839,3840,3841,3842,3843,3844,3845,3846,3847,3848,3849,3850,3851,3852,3853,3854,3855,3856,3857,3858,3859,3860,3861,3862,3863,3864,3865,3866,3867,3868,3869,3870,3871,3872,3873,3874,3875,3876,3877,3878,3879,3880,3881,3882,3883,3884,3885,3886,3887,3888,3889,3890,3891,3892,3893,3894,3895,3896,3897,3898,3899,3900,3901,3902,3903,3904,3905,3906,3907,3908,3909,3910,3911,3912,3913,3914,3915,3916,3917,3918,3919,3920,3921,3922,3923,3924,3925,3926,3927,3928,3929,3930,3931,3932,3933,3934,3935,3936,3937,3938,3939,3940,3941,3942,3943,3944,3945,3946,3947,3948,3949,3950,3951,3952,3953,3954,3955,3956,3957,3958,3959,3960,3961,3962,3963,3964,3965,3966,3967,3968,3969,3970,3971,3972,3973,3974,3975,3976,3977,3978,3979,3980,3981,3982,3983,3984,3985,3986,3987,3988,3989,3990,3991,3992,3993,3994,3995,3996,3997,3998,3999,4000,4001,4002,4003,4004,4005,4006,4007,4008,4009,4010,4011,4012,4013,4014,4015,4016,4017,4018,4019,4020,4021,4022,4023,4024,4025,4026,4027,4028,4029,4030,4031,4032,4033,4034,4035,4036,4037,4038,4039,4040,4041,4042,4043,4044,4045,4046,4047,4048,4049,4050,4051,4052,4053,4054,4055,4056,4057,4058,4059,4060,4061,4062,4063,4064,4065,4066,4067,4068,4069,4070,4071,4072,4073,4074,4075,4076,4077,4078,4079,4080,4081,4082,4083,4084,4085,4086,4087,4088,4089,4090,4091,4092,4093,4094,4095,4096,4097,4098,4099,4100,4101,4102,4103,4104,4105,4106,4107,4108,4109,4110,4111,4112,4113,4114,4115,4116,4117,4118,4119,4120,4121,4122,4123,4124,4125,4126,4127,4128,4129,4130,4131,4132,4133,4134,4135,4136,4137,4138,4139,4140,4141,4142,4143,4144,4145,4146,4147,4148,4149,4150,4151,4152,4153,4154,4155,4156,4157,4158,4159,4160,4161,4162,4163,4164,4165,4166,4167,4168,4169,4170,4171,4172,4173,4174,4175,4176,4177,4178,4179,4180,4181,4182,4183,4184,4185,4186,4187,4188,4189,4190,4191,4192,4193,4194,4195,4196,4197,4198,4199,4200,4201,4202,4203,4204,4205,4206,4207,4208,4209,4210,4211,4212,4213,4214,4215,4216,4217,4218,4219,4220,4221,4222,4223,4224,4225,4226,4227,4228,4229,4230,4231,4232,4233,4234,4235,4236,4237,4238,4239,4240,4241,4242,4243,4244,4245,4246,4247,4248,4249,4250,4251,4252,4253,4254,4255,4256,4257,4258,4259,4260,4261,4262,4263,4264,4265,4266,4267,4268,4269,4270,4271,4272,4273,4274,4275,4276,4277,4278,4279,4280,4281,4282,4283,4284,4285,4286,4287,4288,4289,4290,4291,4292,4293,4294,4295,4296,4297,4298,4299,4300,4301,4302,4303,4304,4305,4306,4307,4308,4309,4310,4311,4312,4313,4314,4315,4316,4317,4318,4319,4320,4321,4322,4323,4324,4325,4326,4327,4328,4329,4330,4331,4332,4333,4334,4335,4336,4337,4338,4339,4340,4341,4342,4343,4344,4345,4346,4347,4348,4349,4350,4351,4352,4353,4354,4355,4356,4357,4358,4359,4360,4361,4362,4363,4364,4365,4366,4367,4368,4369,4370,4371,4372,4373,4374,4375,4376,4377,4378,4379,4380,4381,4382,4383,4384,4385,4386,4387,4388,4389,4390,4391,4392,4393,4394,4395,4396,4397,4398,4399,4400,4401,4402,4403,4404,4405,4406,4407,4408,4409,4410,4411,4412,4413,4414,4415,4416,4417,4418,4419,4420,4421,4422,4423,4424,4425,4426,4427,4428,4429,4430,4431,4432,4433,4434,4435,4436,4437,4438,4439,4440,4441,4442,4443,4444,4445,4446,4447,4448,4449,4450,4451,4452,4453,4454,4455,4456,4457,4458,4459,4460,4461,4462,4463,4464,4465,4466,4467,4468,4469,4470,4471,4472,4473,4474,4475,4476,4477,4478,4479,4480,4481,4482,4483,4484,4485,4486,4487,4488,4489,4490,4491,4492,4493,4494,4495,4496,4497,4498,4499,4500,4501,4502,4503,4504,4505,4506,4507,4508,4509,4510,4511,4512,4513,4514,4515,4516,4517,4518,4519,4520,4521,4522,4523,4524,4525,4526,4527,4528,4529,4530,4531,4532,4533,4534,4535,4536,4537,4538,4539,4540,4541,4542,4543,4544,4545,4546,4547,4548,4549,4550,4551,4552,4553,4554,4555,4556,4557,4558,4559,4560,4561,4562,4563,4564,4565,4566,4567,4568,4569,4570,4571,4572,4573,4574,4575,4576,4577,4578,4579,4580,4581,4582,4583,4584,4585,4586,4587,4588,4589,4590,4591,4592,4593,4594,4595,4596,4597,4598,4599,4600,4601,4602,4603,4604,4605,4606,4607,4608,4609,4610

c1: COM ATOMS=4637,4638,4639,4640,4641,4642,4643,4644,4645,4646,4647,4648,4668,4669

c2: COM ATOMS=4649,4650,4652,4654,4655,4656,4658,4659,4661

h1: COM ATOMS=1278,1297,1307,1317

h2: COM ATOMS=1428,1444,1463,1483

a1: COM ATOMS=4617,4619,4621,4622,4624,4626

a2: COM ATOMS=4654,4655,4656,4658,4659,4661

ser162: COM ATOMS=1516,1517,1518,1519,1520,1521,1522,1523,1524,1525

d1: DISTANCE ATOMS=ser162,4668

tor: TORSION ATOMS=c1,c2,h2,h1

ang: ANGLE ATOMS=a1,c1,a2

lig: COM ATOMS=4637,4640,4641,4642,4643,4644,4645,4646,4647,4668

fps: FUNNEL_PS LIGAND=lig REFERENCE=../REFERENCE.PDB ANCHOR=1157 POINTS=3.9,3.8,5.5,3.9,3.8,8.0

rmsd: RMSD REFERENCE=../RMSD.PDB TYPE=OPTIMAL

FUNNEL ARG=fps.lp,fps.ld ZCC=5.0 ALPHA=0.57 RCYL=0.1 MINS=0.0 MAXS=10.0 KAPPA=35100 NBINS=2000 NBINZ=2000 SAFETY=1.0 SLOPE=1.0 FILE=../BIAS LABEL=funnel WALKERS_MPI

METAD ARG=d1,tor,ang SIGMA=0.3,0.4,0.4 HEIGHT=0.3 PACE=500 TEMP=309.75 BIASFACTOR=120 CALC_RCT RCT_USTRIDE=10 GRID_MIN=-2.0,-pi,0 GRID_MAX=10.0,pi,pi LABEL=metad WALKERS_MPI FILE=../HILLS GRID_WFILE=../grid_w.dat GRID_RFILE=../grid_r.dat GRID_WSTRIDE=250000

LOWER_WALLS ARG=fps.lp AT=0.8 KAPPA=500000 EXP=2 OFFSET=0 LABEL=lwall

UPPER_WALLS ARG=rmsd AT=0.05 KAPPA=500000 EXP=2 OFFSET=0 LABEL=uwall-rmsd

UPPER_WALLS ARG=fps.lp AT=7.5 KAPPA=500000 EXP=2 OFFSET=0 LABEL=uwall

UPPER_WALLS ARG=d1 AT=6.5 KAPPA=5000000 EXP=2 OFFSET=0 LABEL=distwall

LOWER_WALLS ARG=d1 AT=0.8 KAPPA=5000000 EXP=2 OFFSET=0 LABEL=ldistwall

PRINT STRIDE=500 ARG=* FILE=COLVAR

**fes-5ht1a-3d.dat**

#! FIELDS d1 tor projection

#! SET min_d1 -0.830261

#! SET max_d1 9.15632

#! SET nbins_d1 96

#! SET periodic_d1 false

#! SET min_tor -pi

#! SET max_tor pi

#! SET nbins_tor 45

#! SET periodic_tor true

-0.830261000 -3.141592654 1346.633563187

-0.725139095 -3.141592654 1346.010337132

-0.620017189 -3.141592654 1343.713776334

-0.514895284 -3.141592654 1335.938621214

-0.409773379 -3.141592654 1315.154741838

-0.304651474 -3.141592654 1269.412644647

-0.199529568 -3.141592654 1184.490578984

-0.094407663 -3.141592654 1049.719150969

0.010714242 -3.141592654 866.206483391

0.115836147 -3.141592654 653.825038004

0.220958053 -3.141592654 448.820762684

0.326079958 -3.141592654 289.748225707

0.431201863 -3.141592654 192.557902513

0.536323768 -3.141592654 122.096956201

0.641445674 -3.141592654 95.537845020

0.746567579 -3.141592654 93.992109604

0.851689484 -3.141592654 103.049719257

0.956811389 -3.141592654 115.949662373

1.061933295 -3.141592654 129.910239537

1.167055200 -3.141592654 143.236125906

1.272177105 -3.141592654 151.580944827

1.377299011 -3.141592654 130.193318287

1.482420916 -3.141592654 106.795162139

1.587542821 -3.141592654 91.523092834

1.692664726 -3.141592654 81.842163653

1.797786632 -3.141592654 75.352326607

1.902908537 -3.141592654 70.727637752

2.008030442 -3.141592654 68.520916493

2.113152347 -3.141592654 69.364125094

2.218274253 -3.141592654 73.048994060

2.323396158 -3.141592654 78.201513888

2.428518063 -3.141592654 83.366723788

2.533639968 -3.141592654 87.898336559

2.638761874 -3.141592654 92.463886148

2.743883779 -3.141592654 98.771013034

2.849005684 -3.141592654 108.402286729

2.954127589 -3.141592654 121.781551940

3.059249495 -3.141592654 137.135946021

3.164371400 -3.141592654 151.127697819

3.269493305 -3.141592654 160.582043077

3.374615211 -3.141592654 164.107030017

3.479737116 -3.141592654 163.249208423

3.584859021 -3.141592654 161.146312227

3.689980926 -3.141592654 159.884698813

3.795102832 -3.141592654 160.790691961

3.900224737 -3.141592654 163.716800632

4.005346642 -3.141592654 167.551424645

4.110468547 -3.141592654 170.765202262

4.215590453 -3.141592654 171.906156386

4.320712358 -3.141592654 170.375158685

4.425834263 -3.141592654 166.894232954

4.530956168 -3.141592654 163.216128561

4.636078074 -3.141592654 161.015237172

4.741199979 -3.141592654 161.022254375

4.846321884 -3.141592654 162.923666476

4.951443789 -3.141592654 165.846099247

5.056565695 -3.141592654 168.972895898

5.161687600 -3.141592654 171.823187022

5.266809505 -3.141592654 174.326761551

5.371931411 -3.141592654 176.622623243

5.477053316 -3.141592654 178.825937764

5.582175221 -3.141592654 180.822804240

5.687297126 -3.141592654 182.432814594

5.792419032 -3.141592654 183.851886266

5.897540937 -3.141592654 186.054683340

6.002662842 -3.141592654 190.383230809

6.107784747 -3.141592654 197.416132507

6.212906653 -3.141592654 206.269994296

6.318028558 -3.141592654 215.026012413

6.423150463 -3.141592654 222.224350147

6.528272368 -3.141592654 227.948358219

6.633394274 -3.141592654 233.295120824

6.738516179 -3.141592654 238.891354157

6.843638084 -3.141592654 242.324675126

6.948759989 -3.141592654 242.934955901

7.053881895 -3.141592654 242.320624465

7.159003800 -3.141592654 242.700547349

7.264125705 -3.141592654 247.269942168

7.369247611 -3.141592654 257.681672849

7.474369516 -3.141592654 270.585871325

7.579491421 -3.141592654 277.439828783

7.684613326 -3.141592654 270.662486654

7.789735232 -3.141592654 253.949585654

7.894857137 -3.141592654 247.879025002

7.999979042 -3.141592654 283.013245597

8.105100947 -3.141592654 382.198082477

8.210222853 -3.141592654 544.577932401

8.315344758 -3.141592654 743.595274935

8.420466663 -3.141592654 940.843725538

8.525588568 -3.141592654 1104.325547535

8.630710474 -3.141592654 1219.535171553

8.735832379 -3.141592654 1288.592329259

8.840954284 -3.141592654 1323.606007512

8.946076189 -3.141592654 1339.048002543

9.051198095 -3.141592654 1344.645371362

9.156320000 -3.141592654 1346.282501310

-0.830261000 -3.001966313 1346.635988206

-0.725139095 -3.001966313 1346.020070662

-0.620017189 -3.001966313 1343.744531646

-0.514895284 -3.001966313 1336.003277188

-0.409773379 -3.001966313 1315.133504724

-0.304651474 -3.001966313 1269.102897875

-0.199529568 -3.001966313 1183.850871663

-0.094407663 -3.001966313 1048.654307633

0.010714242 -3.001966313 864.662301840

0.115836147 -3.001966313 651.859281703

0.220958053 -3.001966313 446.628570907

0.326079958 -3.001966313 287.663391412

0.431201863 -3.001966313 197.102626022

0.536323768 -3.001966313 130.504088597

0.641445674 -3.001966313 96.426632596

0.746567579 -3.001966313 88.789725481

0.851689484 -3.001966313 94.486925644

0.956811389 -3.001966313 106.951714732

1.061933295 -3.001966313 122.696323055

1.167055200 -3.001966313 139.076526840

1.272177105 -3.001966313 149.774224948

1.377299011 -3.001966313 121.035654729

1.482420916 -3.001966313 96.256221673

1.587542821 -3.001966313 80.046586301

1.692664726 -3.001966313 70.357201352

1.797786632 -3.001966313 64.751582420

1.902908537 -3.001966313 61.371619072

2.008030442 -3.001966313 60.369656498

2.113152347 -3.001966313 62.429797307

2.218274253 -3.001966313 67.621548460

2.323396158 -3.001966313 74.632043178

2.428518063 -3.001966313 81.640954856

2.533639968 -3.001966313 87.447602308

2.638761874 -3.001966313 92.452690384

2.743883779 -3.001966313 98.507264052

2.849005684 -3.001966313 107.632688543

2.954127589 -3.001966313 120.592231939

3.059249495 -3.001966313 135.600870006

3.164371400 -3.001966313 149.057280149

3.269493305 -3.001966313 157.758286816

3.374615211 -3.001966313 160.636362448

3.479737116 -3.001966313 159.391828246

3.584859021 -3.001966313 157.019376881

3.689980926 -3.001966313 155.846323145

3.795102832 -3.001966313 156.922226976

3.900224737 -3.001966313 160.008617846

4.005346642 -3.001966313 164.168697808

4.110468547 -3.001966313 168.057663295

4.215590453 -3.001966313 170.207933337

4.320712358 -3.001966313 169.774090339

4.425834263 -3.001966313 167.146332193

4.530956168 -3.001966313 163.882664732

4.636078074 -3.001966313 161.700273696

4.741199979 -3.001966313 161.515481821

4.846321884 -3.001966313 163.204932567

4.951443789 -3.001966313 165.990373949

5.056565695 -3.001966313 169.060563345

5.161687600 -3.001966313 171.903654461

5.266809505 -3.001966313 174.420199453

5.371931411 -3.001966313 176.759055456

5.477053316 -3.001966313 179.065881984

5.582175221 -3.001966313 181.216013931

5.687297126 -3.001966313 182.961395494

5.792419032 -3.001966313 184.481250734

5.897540937 -3.001966313 186.802536952

6.002662842 -3.001966313 191.283086463

6.107784747 -3.001966313 198.487190180

6.212906653 -3.001966313 207.502010673

6.318028558 -3.001966313 216.346192592

6.423150463 -3.001966313 223.520929839

6.528272368 -3.001966313 229.217515642

6.633394274 -3.001966313 234.591982200

6.738516179 -3.001966313 240.004168496

6.843638084 -3.001966313 242.955334023

6.948759989 -3.001966313 243.315606925

7.053881895 -3.001966313 242.492310635

7.159003800 -3.001966313 242.696556220

7.264125705 -3.001966313 247.156082048

7.369247611 -3.001966313 257.531238686

7.474369516 -3.001966313 270.405414771

7.579491421 -3.001966313 277.087655062

7.684613326 -3.001966313 269.863151435

7.789735232 -3.001966313 252.436084713

7.894857137 -3.001966313 245.599603049

7.999979042 -3.001966313 280.221341350

8.105100947 -3.001966313 379.352276054

8.210222853 -3.001966313 542.119813741

8.315344758 -3.001966313 741.779871643

8.420466663 -3.001966313 939.695072701

8.525588568 -3.001966313 1103.700069629

8.630710474 -3.001966313 1219.245878378

8.735832379 -3.001966313 1288.488340128

8.840954284 -3.001966313 1323.575279647

8.946076189 -3.001966313 1339.039707880

9.051198095 -3.001966313 1344.643205033

9.156320000 -3.001966313 1346.283229268

-0.830261000 -2.862339973 1346.639078135

-0.725139095 -2.862339973 1346.032003163

-0.620017189 -2.862339973 1343.780447548

-0.514895284 -2.862339973 1336.060181453

-0.409773379 -2.862339973 1315.073231249

-0.304651474 -2.862339973 1268.803549213

-0.199529568 -2.862339973 1183.247632873

-0.094407663 -2.862339973 1047.611818353

0.010714242 -2.862339973 863.090282561

0.115836147 -2.862339973 649.774628449

0.220958053 -2.862339973 444.215257863

0.326079958 -2.862339973 285.287855447

0.431201863 -2.862339973 195.163197910

0.536323768 -2.862339973 148.393985962

0.641445674 -2.862339973 105.377361046

0.746567579 -2.862339973 89.897606913

0.851689484 -2.862339973 90.421585234

0.956811389 -2.862339973 100.665539962

1.061933295 -2.862339973 116.665118116

1.167055200 -2.862339973 134.848838074

1.272177105 -2.862339973 145.513593644

1.377299011 -2.862339973 113.825675919

1.482420916 -2.862339973 88.360445393

1.587542821 -2.862339973 71.740466872

1.692664726 -2.862339973 62.486805915

1.797786632 -2.862339973 58.152069384

1.902908537 -2.862339973 56.288009103

2.008030442 -2.862339973 56.488286279

2.113152347 -2.862339973 59.390074491

2.218274253 -2.862339973 65.437555521

2.323396158 -2.862339973 73.681109725

2.428518063 -2.862339973 82.259702010

2.533639968 -2.862339973 89.550691166

2.638761874 -2.862339973 95.533895268

2.743883779 -2.862339973 101.858171163

2.849005684 -2.862339973 110.664406211

2.954127589 -2.862339973 122.913695930

3.059249495 -2.862339973 136.890399911

3.164371400 -2.862339973 148.947478957

3.269493305 -2.862339973 156.016495638

3.374615211 -2.862339973 157.350705852

3.479737116 -2.862339973 154.937958237

3.584859021 -2.862339973 151.885695201

3.689980926 -2.862339973 150.474315826

3.795102832 -2.862339973 151.586584002

3.900224737 -2.862339973 154.943304921

4.005346642 -2.862339973 159.732285407

4.110468547 -2.862339973 164.706234421

4.215590453 -2.862339973 168.299601318

4.320712358 -2.862339973 169.355372322

4.425834263 -2.862339973 167.900562757

4.530956168 -2.862339973 165.284782040

4.636078074 -2.862339973 163.269679152

4.741199979 -2.862339973 162.969044803

4.846321884 -2.862339973 164.460410333

4.951443789 -2.862339973 167.062994981

5.056565695 -2.862339973 169.974611330

5.161687600 -2.862339973 172.665386667

5.266809505 -2.862339973 175.030547336

5.371931411 -2.862339973 177.250987177

5.477053316 -2.862339973 179.508108697

5.582175221 -2.862339973 181.682591051

5.687297126 -2.862339973 183.499052131

5.792419032 -2.862339973 185.122117148

5.897540937 -2.862339973 187.588112665

6.002662842 -2.862339973 192.236564581

6.107784747 -2.862339973 199.587947933

6.212906653 -2.862339973 208.671291183

6.318028558 -2.862339973 217.437587879

6.423150463 -2.862339973 224.450992521

6.528272368 -2.862339973 230.101244388

6.633394274 -2.862339973 235.569635125

6.738516179 -2.862339973 240.968166007

6.843638084 -2.862339973 243.677627015

6.948759989 -2.862339973 243.871813190

7.053881895 -2.862339973 242.884251329

7.159003800 -2.862339973 242.939808590

7.264125705 -2.862339973 247.296535145

7.369247611 -2.862339973 257.623660859

7.474369516 -2.862339973 270.456545198

7.579491421 -2.862339973 276.988941331

7.684613326 -2.862339973 269.396356744

7.789735232 -2.862339973 251.380818442

7.894857137 -2.862339973 243.901773362

7.999979042 -2.862339973 278.070634442

8.105100947 -2.862339973 377.114746578

8.210222853 -2.862339973 540.159177744

8.315344758 -2.862339973 740.316667098

8.420466663 -2.862339973 938.761673340

8.525588568 -2.862339973 1103.188309248

8.630710474 -2.862339973 1219.008071198

8.735832379 -2.862339973 1288.404205571

8.840954284 -2.862339973 1323.552947847

8.946076189 -2.862339973 1339.034511537

9.051198095 -2.862339973 1344.641894833

9.156320000 -2.862339973 1346.284084711

-0.830261000 -2.722713633 1346.642597941

-0.725139095 -2.722713633 1346.045166679

-0.620017189 -2.722713633 1343.817861568

-0.514895284 -2.722713633 1336.101523651

-0.409773379 -2.722713633 1314.984121341

-0.304651474 -2.722713633 1268.513480501

-0.199529568 -2.722713633 1182.655068411

-0.094407663 -2.722713633 1046.556693396

0.010714242 -2.722713633 861.453031797

0.115836147 -2.722713633 647.541453299

0.220958053 -2.722713633 441.572903628

0.326079958 -2.722713633 282.658600060

0.431201863 -2.722713633 193.058745733

0.536323768 -2.722713633 166.775025471

0.641445674 -2.722713633 121.653988849

0.746567579 -2.722713633 97.675847674

0.851689484 -2.722713633 91.835392494

0.956811389 -2.722713633 98.346050422

1.061933295 -2.722713633 113.088977217

1.167055200 -2.722713633 131.507336021

1.272177105 -2.722713633 142.522687093

1.377299011 -2.722713633 110.539425881

1.482420916 -2.722713633 84.910423705

1.587542821 -2.722713633 68.391735310

1.692664726 -2.722713633 59.882535603

1.797786632 -2.722713633 56.866055277

1.902908537 -2.722713633 56.342990337

2.008030442 -2.722713633 57.353810750

2.113152347 -2.722713633 60.480063717

2.218274253 -2.722713633 66.593552955

2.323396158 -2.722713633 75.273732179

2.428518063 -2.722713633 84.839256006

2.533639968 -2.722713633 93.387102202

2.638761874 -2.722713633 100.432793687

2.743883779 -2.722713633 107.222048569

2.849005684 -2.722713633 115.786169041

2.954127589 -2.722713633 127.141069819

3.059249495 -2.722713633 139.677642567

3.164371400 -2.722713633 149.871120786

3.269493305 -2.722713633 154.881103721

3.374615211 -2.722713633 154.281057757

3.479737116 -2.722713633 150.326286070

3.584859021 -2.722713633 146.277656513

3.689980926 -2.722713633 144.418056084

3.795102832 -2.722713633 145.536329376

3.900224737 -2.722713633 149.309545366

4.005346642 -2.722713633 154.956835486

4.110468547 -2.722713633 161.219372333

4.215590453 -2.722713633 166.383395821

4.320712358 -2.722713633 169.000663294

4.425834263 -2.722713633 168.787491910

4.530956168 -2.722713633 166.915130039

4.636078074 -2.722713633 165.182603158

4.741199979 -2.722713633 164.873637160

4.846321884 -2.722713633 166.238348922

4.951443789 -2.722713633 168.676232876

5.056565695 -2.722713633 171.397727218

5.161687600 -2.722713633 173.870626808

5.266809505 -2.722713633 176.005809665

5.371931411 -2.722713633 178.026938537

5.477053316 -2.722713633 180.149384394

5.582175221 -2.722713633 182.264909354

5.687297126 -2.722713633 184.093244950

5.792419032 -2.722713633 185.795266907

5.897540937 -2.722713633 188.400951408

6.002662842 -2.722713633 193.211386665

6.107784747 -2.722713633 200.673504241

6.212906653 -2.722713633 209.707521816

6.318028558 -2.722713633 218.162582372

6.423150463 -2.722713633 224.840816948

6.528272368 -2.722713633 230.430460000

6.633394274 -2.722713633 236.072899626

6.738516179 -2.722713633 241.649804523

6.843638084 -2.722713633 244.314418138

6.948759989 -2.722713633 244.446268816

7.053881895 -2.722713633 243.380398252

7.159003800 -2.722713633 243.351745516

7.264125705 -2.722713633 247.642190672

7.369247611 -2.722713633 257.934258646

7.474369516 -2.722713633 270.737637117

7.579491421 -2.722713633 277.166156777

7.684613326 -2.722713633 269.311372828

7.789735232 -2.722713633 250.859861297

7.894857137 -2.722713633 242.879561733

7.999979042 -2.722713633 276.657513654

8.105100947 -2.722713633 375.567794254

8.210222853 -2.722713633 538.754276145

8.315344758 -2.722713633 739.238149153

8.420466663 -2.722713633 938.055914396

8.525588568 -2.722713633 1102.791535922

8.630710474 -2.722713633 1218.818746825

8.735832379 -2.722713633 1288.337042106

8.840954284 -2.722713633 1323.537319789

8.946076189 -2.722713633 1339.031726213

9.051198095 -2.722713633 1344.641226432

9.156320000 -2.722713633 1346.284936756

-0.830261000 -2.583087293 1346.645951676

-0.725139095 -2.583087293 1346.057377405

-0.620017189 -2.583087293 1343.849725445

-0.514895284 -2.583087293 1336.115845945

-0.409773379 -2.583087293 1314.856530691

-0.304651474 -2.583087293 1268.179017072

-0.199529568 -2.583087293 1181.976315066

-0.094407663 -2.583087293 1045.353084572

0.010714242 -2.583087293 859.585528543

0.115836147 -2.583087293 644.990197696

0.220958053 -2.583087293 438.556312899

0.326079958 -2.583087293 279.667397926

0.431201863 -2.583087293 190.762551899

0.536323768 -2.583087293 166.057894736

0.641445674 -2.583087293 141.666028344

0.746567579 -2.583087293 110.693791238

0.851689484 -2.583087293 98.680861794

0.956811389 -2.583087293 100.864118448

1.061933295 -2.583087293 113.142339351

1.167055200 -2.583087293 130.172503292

1.272177105 -2.583087293 142.769566486

1.377299011 -2.583087293 111.496165267

1.482420916 -2.583087293 86.159087720

1.587542821 -2.583087293 70.258534745

1.692664726 -2.583087293 62.745591202

1.797786632 -2.583087293 60.923062947

1.902908537 -2.583087293 61.333829051

2.008030442 -2.583087293 62.568549276

2.113152347 -2.583087293 65.184059202

2.218274253 -2.583087293 70.484516706

2.323396158 -2.583087293 78.654430216

2.428518063 -2.583087293 88.373780331

2.533639968 -2.583087293 97.648529846

2.638761874 -2.583087293 105.577757578

2.743883779 -2.583087293 112.873943749

2.849005684 -2.583087293 121.238994469

2.954127589 -2.583087293 131.590923240

3.059249495 -2.583087293 142.466259025

3.164371400 -2.583087293 150.633210496

3.269493305 -2.583087293 153.573912162

3.374615211 -2.583087293 151.160816064

3.479737116 -2.583087293 145.823146545

3.584859021 -2.583087293 140.910905213

3.689980926 -2.583087293 138.717855484

3.795102832 -2.583087293 139.971971304

3.900224737 -2.583087293 144.288770066

4.005346642 -2.583087293 150.832419691

4.110468547 -2.583087293 158.263741574

4.215590453 -2.583087293 164.739464525

4.320712358 -2.583087293 168.620461202

4.425834263 -2.583087293 169.429434899

4.530956168 -2.583087293 168.216932382

4.636078074 -2.583087293 166.801382243

4.741199979 -2.583087293 166.579386544

4.846321884 -2.583087293 167.919200188

4.951443789 -2.583087293 170.272131450

5.056565695 -2.583087293 172.861759430

5.161687600 -2.583087293 175.161287450

5.266809505 -2.583087293 177.102939690

5.371931411 -2.583087293 178.947052544

5.477053316 -2.583087293 180.933251102

5.582175221 -2.583087293 182.963723412

5.687297126 -2.583087293 184.765495217

5.792419032 -2.583087293 186.510043973

5.897540937 -2.583087293 189.220744596

6.002662842 -2.583087293 194.150363390

6.107784747 -2.583087293 201.643838843

6.212906653 -2.583087293 210.420404858

6.318028558 -2.583087293 218.226615918

6.423150463 -2.583087293 224.441503267

6.528272368 -2.583087293 230.014345754

6.633394274 -2.583087293 235.933980164

6.738516179 -2.583087293 241.898325071

6.843638084 -2.583087293 244.709725906

6.948759989 -2.583087293 244.903997783

7.053881895 -2.583087293 243.876024275

7.159003800 -2.583087293 243.851969251

7.264125705 -2.583087293 248.128832612

7.369247611 -2.583087293 258.411201424

7.474369516 -2.583087293 271.209236418

7.579491421 -2.583087293 277.592307510

7.684613326 -2.583087293 269.593003852

7.789735232 -2.583087293 250.870193610

7.894857137 -2.583087293 242.542824290

7.999979042 -2.583087293 276.004232540

8.105100947 -2.583087293 374.741696245

8.210222853 -2.583087293 537.937123320

8.315344758 -2.583087293 738.571809889

8.420466663 -2.583087293 937.597539572

8.525588568 -2.583087293 1102.521968361

8.630710474 -2.583087293 1218.684118238

8.735832379 -2.583087293 1288.289146281

8.840954284 -2.583087293 1323.528619578

8.946076189 -2.583087293 1339.031310493

9.051198095 -2.583087293 1344.641199870

9.156320000 -2.583087293 1346.285736958

-0.830261000 -2.443460953 1346.648609967

-0.725139095 -2.443460953 1346.066833924

-0.620017189 -2.443460953 1343.871164888

-0.514895284 -2.443460953 1336.098961413

-0.409773379 -2.443460953 1314.677903393

-0.304651474 -2.443460953 1267.746440053

-0.199529568 -2.443460953 1181.121443832

-0.094407663 -2.443460953 1043.877560162

0.010714242 -2.443460953 857.342971429

0.115836147 -2.443460953 641.983769165

0.220958053 -2.443460953 435.058439586

0.326079958 -2.443460953 276.218642251

0.431201863 -2.443460953 188.103103040

0.536323768 -2.443460953 165.143454114

0.641445674 -2.443460953 162.371632086

0.746567579 -2.443460953 127.111344197

0.851689484 -2.443460953 110.447065969

0.956811389 -2.443460953 108.527801114

1.061933295 -2.443460953 117.485830793

1.167055200 -2.443460953 131.752139143

1.272177105 -2.443460953 144.260190667

1.377299011 -2.443460953 115.909955552

1.482420916 -2.443460953 91.247069916

1.587542821 -2.443460953 76.409045227

1.692664726 -2.443460953 70.102837999

1.797786632 -2.443460953 69.344166804

1.902908537 -2.443460953 70.314868307

2.008030442 -2.443460953 71.263491337

2.113152347 -2.443460953 72.719372947

2.218274253 -2.443460953 76.362357541

2.323396158 -2.443460953 83.009290636

2.428518063 -2.443460953 91.900307734

2.533639968 -2.443460953 101.219426434

2.638761874 -2.443460953 109.778132169

2.743883779 -2.443460953 117.654044302

2.849005684 -2.443460953 125.960887236

2.954127589 -2.443460953 135.328799443

3.059249495 -2.443460953 144.467407240

3.164371400 -2.443460953 150.618686764

3.269493305 -2.443460953 151.726762735

3.374615211 -2.443460953 147.962149810

3.479737116 -2.443460953 141.798091706

3.584859021 -2.443460953 136.526382627

3.689980926 -2.443460953 134.378438617

3.795102832 -2.443460953 136.003397358

3.900224737 -2.443460953 140.927844899

4.005346642 -2.443460953 148.206114830

4.110468547 -2.443460953 156.407211129

4.215590453 -2.443460953 163.634137246

4.320712358 -2.443460953 168.201910472

4.425834263 -2.443460953 169.586439025

4.530956168 -2.443460953 168.788078486

4.636078074 -2.443460953 167.624473253

4.741199979 -2.443460953 167.539154448

4.846321884 -2.443460953 168.955917090

4.951443789 -2.443460953 171.346128223

5.056565695 -2.443460953 173.939039129

5.161687600 -2.443460953 176.204810900

5.266809505 -2.443460953 178.083064055

5.371931411 -2.443460953 179.851385932

5.477053316 -2.443460953 181.761283814

5.582175221 -2.443460953 183.725673028

5.687297126 -2.443460953 185.486779154

5.792419032 -2.443460953 187.234860676

5.897540937 -2.443460953 189.987263123

6.002662842 -2.443460953 194.943211159

6.107784747 -2.443460953 202.301490210

6.212906653 -2.443460953 210.419227908

6.318028558 -2.443460953 217.241981924

6.423150463 -2.443460953 223.052095063

6.528272368 -2.443460953 228.724603927

6.633394274 -2.443460953 235.026047669

6.738516179 -2.443460953 241.579775784

6.843638084 -2.443460953 244.744955159

6.948759989 -2.443460953 245.135042492

7.053881895 -2.443460953 244.269724544

7.159003800 -2.443460953 244.343913364

7.264125705 -2.443460953 248.662096812

7.369247611 -2.443460953 258.962881827

7.474369516 -2.443460953 271.784037636

7.579491421 -2.443460953 278.182796130

7.684613326 -2.443460953 270.154673029

7.789735232 -2.443460953 251.321950160

7.894857137 -2.443460953 242.804977306

7.999979042 -2.443460953 276.039379048

8.105100947 -2.443460953 374.588767617

8.210222853 -2.443460953 537.684948523

8.315344758 -2.443460953 738.313815515

8.420466663 -2.443460953 937.392982798

8.525588568 -2.443460953 1102.388785088

8.630710474 -2.443460953 1218.611573386

8.735832379 -2.443460953 1288.264478984

8.840954284 -2.443460953 1323.527964899

8.946076189 -2.443460953 1339.033584505

9.051198095 -2.443460953 1344.641946489

9.156320000 -2.443460953 1346.286511591

-0.830261000 -2.303834613 1346.650294658

-0.725139095 -2.303834613 1346.072681844

-0.620017189 -2.303834613 1343.880739038

-0.514895284 -2.303834613 1336.054430826

-0.409773379 -2.303834613 1314.451210715

-0.304651474 -2.303834613 1267.212613990

-0.199529568 -2.303834613 1180.092987469

-0.094407663 -2.303834613 1042.148521116

0.010714242 -2.303834613 854.772673708

0.115836147 -2.303834613 638.608945061

0.220958053 -2.303834613 431.185548431

0.326079958 -2.303834613 272.380696114

0.431201863 -2.303834613 184.990517314

0.536323768 -2.303834613 163.704889711

0.641445674 -2.303834613 179.653694736

0.746567579 -2.303834613 145.840053293

0.851689484 -2.303834613 126.164921732

0.956811389 -2.303834613 120.669568472

1.061933295 -2.303834613 125.867210780

1.167055200 -2.303834613 136.503481278

1.272177105 -2.303834613 146.317175411

1.377299011 -2.303834613 122.329128177

1.482420916 -2.303834613 98.645840817

1.587542821 -2.303834613 85.191886908

1.692664726 -2.303834613 80.285129947

1.797786632 -2.303834613 80.588164852

1.902908537 -2.303834613 81.982046267

2.008030442 -2.303834613 82.425908134

2.113152347 -2.303834613 82.356001740

2.218274253 -2.303834613 83.705901181

2.323396158 -2.303834613 87.906266380

2.428518063 -2.303834613 94.959961588

2.533639968 -2.303834613 103.555415324

2.638761874 -2.303834613 112.424917672

2.743883779 -2.303834613 120.974125927

2.849005684 -2.303834613 129.484309995

2.954127589 -2.303834613 138.082686735

3.059249495 -2.303834613 145.624841274

3.164371400 -2.303834613 149.952207915

3.269493305 -2.303834613 149.600308957

3.374615211 -2.303834613 145.052836683

3.479737116 -2.303834613 138.715533281

3.584859021 -2.303834613 133.668858688

3.689980926 -2.303834613 131.984232915

3.795102832 -2.303834613 134.193685474

3.900224737 -2.303834613 139.709830450

4.005346642 -2.303834613 147.452820955

4.110468547 -2.303834613 155.915771219

4.215590453 -2.303834613 163.233612851

4.320712358 -2.303834613 167.819065663

4.425834263 -2.303834613 169.240422573

4.530956168 -2.303834613 168.521901822

4.636078074 -2.303834613 167.469789111

4.741199979 -2.303834613 167.519324929

4.846321884 -2.303834613 169.094360312

4.951443789 -2.303834613 171.657357191

5.056565695 -2.303834613 174.425702772

5.161687600 -2.303834613 176.841746472

5.266809505 -2.303834613 178.824268968

5.371931411 -2.303834613 180.645288339

5.477053316 -2.303834613 182.561212996

5.582175221 -2.303834613 184.500448565

5.687297126 -2.303834613 186.222728977

5.792419032 -2.303834613 187.932116298

5.897540937 -2.303834613 190.633512492

6.002662842 -2.303834613 195.461500586

6.107784747 -2.303834613 202.356968569

6.212906653 -2.303834613 209.243963001

6.318028558 -2.303834613 215.090358672

6.423150463 -2.303834613 220.712890438

6.528272368 -2.303834613 226.595500041

6.633394274 -2.303834613 233.331993935

6.738516179 -2.303834613 240.625339178

6.843638084 -2.303834613 244.383018428

6.948759989 -2.303834613 245.091108493

7.053881895 -2.303834613 244.492254348

7.159003800 -2.303834613 244.736163200

7.264125705 -2.303834613 249.131426282

7.369247611 -2.303834613 259.464635797

7.474369516 -2.303834613 272.327540350

7.579491421 -2.303834613 278.793665259

7.684613326 -2.303834613 270.839744116

7.789735232 -2.303834613 252.045646350

7.894857137 -2.303834613 243.493519414

7.999979042 -2.303834613 276.606105932

8.105100947 -2.303834613 374.985093985

8.210222853 -2.303834613 537.914727633

8.315344758 -2.303834613 738.418827502

8.420466663 -2.303834613 937.424131557

8.525588568 -2.303834613 1102.388698473

8.630710474 -2.303834613 1218.603317940

8.735832379 -2.303834613 1288.265399780

8.840954284 -2.303834613 1323.536235043

8.946076189 -2.303834613 1339.038860233

9.051198095 -2.303834613 1344.643605208

9.156320000 -2.303834613 1346.287326579

-0.830261000 -2.164208272 1346.650965925

-0.725139095 -2.164208272 1346.074852024

-0.620017189 -2.164208272 1343.879350279

-0.514895284 -2.164208272 1335.990823541

-0.409773379 -2.164208272 1314.202372038

-0.304651474 -2.164208272 1266.641716469

-0.199529568 -2.164208272 1179.016135294

-0.094407663 -2.164208272 1040.372858417

0.010714242 -2.164208272 852.173367279

0.115836147 -2.164208272 635.230245504

0.220958053 -2.164208272 427.290946040

0.326079958 -2.164208272 268.400723640

0.431201863 -2.164208272 181.477153068

0.536323768 -2.164208272 161.414782928

0.641445674 -2.164208272 180.427381467

0.746567579 -2.164208272 165.683777037

0.851689484 -2.164208272 144.289139222

0.956811389 -2.164208272 135.817317280

1.061933295 -2.164208272 137.241677945

1.167055200 -2.164208272 143.965818548

1.272177105 -2.164208272 150.046606286

1.377299011 -2.164208272 128.470718236

1.482420916 -2.164208272 106.132176185

1.587542821 -2.164208272 94.395999708

1.692664726 -2.164208272 91.213400014

1.797786632 -2.164208272 92.855899568

1.902908537 -2.164208272 94.901403314

2.008030442 -2.164208272 95.013448787

2.113152347 -2.164208272 93.439847325

2.218274253 -2.164208272 92.216963400

2.323396158 -2.164208272 93.327314444

2.428518063 -2.164208272 97.681741248

2.533639968 -2.164208272 104.773381190

2.638761874 -2.164208272 113.513605088

2.743883779 -2.164208272 122.723046919

2.849005684 -2.164208272 131.732320170

2.954127589 -2.164208272 139.982191749

3.059249495 -2.164208272 146.363912724

3.164371400 -2.164208272 149.306284460

3.269493305 -2.164208272 147.958111558

3.374615211 -2.164208272 143.109887281

3.479737116 -2.164208272 137.053552411

3.584859021 -2.164208272 132.591272003

3.689980926 -2.164208272 131.592916958

3.795102832 -2.164208272 134.462579155

3.900224737 -2.164208272 140.489696199

4.005346642 -2.164208272 148.442202697

4.110468547 -2.164208272 156.731744526

4.215590453 -2.164208272 163.580830340

4.320712358 -2.164208272 167.606686788

4.425834263 -2.164208272 168.574778344

4.530956168 -2.164208272 167.608075505

4.636078074 -2.164208272 166.505034711

4.741199979 -2.164208272 166.656119497

4.846321884 -2.164208272 168.443274709

4.951443789 -2.164208272 171.296954574

5.056565695 -2.164208272 174.398378988

5.161687600 -2.164208272 177.129341116

5.266809505 -2.164208272 179.356347082

5.371931411 -2.164208272 181.329321006

5.477053316 -2.164208272 183.312414651

5.582175221 -2.164208272 185.258687607

5.687297126 -2.164208272 186.941743430

5.792419032 -2.164208272 188.561520213

5.897540937 -2.164208272 191.093172446

6.002662842 -2.164208272 195.565240876

6.107784747 -2.164208272 201.471366191

6.212906653 -2.164208272 206.871478462

6.318028558 -2.164208272 212.105358224

6.423150463 -2.164208272 217.704628289

6.528272368 -2.164208272 223.818570662

6.633394274 -2.164208272 230.952945861

6.738516179 -2.164208272 239.034567605

6.843638084 -2.164208272 243.670912867

6.948759989 -2.164208272 244.793049435

7.053881895 -2.164208272 244.523198833

7.159003800 -2.164208272 244.970500970

7.264125705 -2.164208272 249.447760626

7.369247611 -2.164208272 259.802228116

7.474369516 -2.164208272 272.703060429

7.579491421 -2.164208272 279.265472435

7.684613326 -2.164208272 271.464155167

7.789735232 -2.164208272 252.833919860

7.894857137 -2.164208272 244.388785245

7.999979042 -2.164208272 277.493761182

8.105100947 -2.164208272 375.751798640

8.210222853 -2.164208272 538.493678881

8.315344758 -2.164208272 738.801969278

8.420466663 -2.164208272 937.645404453

8.525588568 -2.164208272 1102.501625879

8.630710474 -2.164208272 1218.652737090

8.735832379 -2.164208272 1288.290489958

8.840954284 -2.164208272 1323.553163093

8.946076189 -2.164208272 1339.047108649

9.051198095 -2.164208272 1344.646211258

9.156320000 -2.164208272 1346.288244839

-0.830261000 -2.024581932 1346.650681350

-0.725139095 -2.024581932 1346.073523487

-0.620017189 -2.024581932 1343.868233753

-0.514895284 -2.024581932 1335.916367055

-0.409773379 -2.024581932 1313.964522025

-0.304651474 -2.024581932 1266.119730910

-0.199529568 -2.024581932 1178.050698387

-0.094407663 -2.024581932 1038.799535038

0.010714242 -2.024581932 849.876164947

0.115836147 -2.024581932 632.201975182

0.220958053 -2.024581932 423.649419292

0.326079958 -2.024581932 264.371096883

0.431201863 -2.024581932 177.404862713

0.536323768 -2.024581932 157.861885159

0.641445674 -2.024581932 178.342580023

0.746567579 -2.024581932 185.389505036

0.851689484 -2.024581932 163.148847052

0.956811389 -2.024581932 152.235548145

1.061933295 -2.024581932 150.156622483

1.167055200 -2.024581932 153.121667519

1.272177105 -2.024581932 152.884664216

1.377299011 -2.024581932 131.333667744

1.482420916 -2.024581932 110.821787216

1.587542821 -2.024581932 101.317212327

1.692664726 -2.024581932 100.463519141

1.797786632 -2.024581932 104.092681911

1.902908537 -2.024581932 107.418439847

2.008030442 -2.024581932 107.774896169

2.113152347 -2.024581932 105.155481038

2.218274253 -2.024581932 101.581622290

2.323396158 -2.024581932 99.490911981

2.428518063 -2.024581932 100.715088379

2.533639968 -2.024581932 105.706105401

2.638761874 -2.024581932 113.779781295

2.743883779 -2.024581932 123.391679619

2.849005684 -2.024581932 133.021555760

2.954127589 -2.024581932 141.364449564

3.059249495 -2.024581932 147.191521890

3.164371400 -2.024581932 149.358834579

3.269493305 -2.024581932 147.507634088

3.374615211 -2.024581932 142.674284495

3.479737116 -2.024581932 137.051149483

3.584859021 -2.024581932 133.205179470

3.689980926 -2.024581932 132.851126890

3.795102832 -2.024581932 136.305977372

3.900224737 -2.024581932 142.743479069

4.005346642 -2.024581932 150.744262595

4.110468547 -2.024581932 158.592638412

4.215590453 -2.024581932 164.635722991

4.320712358 -2.024581932 167.730493759

4.425834263 -2.024581932 167.904998896

4.530956168 -2.024581932 166.451123437

4.636078074 -2.024581932 165.168775374

4.741199979 -2.024581932 165.381884324

4.846321884 -2.024581932 167.402347191

4.951443789 -2.024581932 170.615185958

5.056565695 -2.024581932 174.145058524

5.161687600 -2.024581932 177.286626007

5.266809505 -2.024581932 179.829989979

5.371931411 -2.024581932 181.994387886

5.477053316 -2.024581932 184.059047952

5.582175221 -2.024581932 186.010827547

5.687297126 -2.024581932 187.631097716

5.792419032 -2.024581932 189.093265935

5.897540937 -2.024581932 191.312976123

6.002662842 -2.024581932 195.116768180

6.107784747 -2.024581932 199.586365632

6.212906653 -2.024581932 203.866573832

6.318028558 -2.024581932 208.793794863

6.423150463 -2.024581932 214.396437448

6.528272368 -2.024581932 220.662331157

6.633394274 -2.024581932 228.071190210

6.738516179 -2.024581932 236.868024624

6.843638084 -2.024581932 242.739723630

6.948759989 -2.024581932 244.338062867

7.053881895 -2.024581932 244.406952750

7.159003800 -2.024581932 245.054308059

7.264125705 -2.024581932 249.596305146

7.369247611 -2.024581932 259.944733239

7.474369516 -2.024581932 272.859799485

7.579491421 -2.024581932 279.518510595

7.684613326 -2.024581932 271.910566974

7.789735232 -2.024581932 253.528659582

7.894857137 -2.024581932 245.300037855

7.999979042 -2.024581932 278.499883858

8.105100947 -2.024581932 376.701327319

8.210222853 -2.024581932 539.270489089

8.315344758 -2.024581932 739.357344124

8.420466663 -2.024581932 937.993005061

8.525588568 -2.024581932 1102.694416620

8.630710474 -2.024581932 1218.745397337

8.735832379 -2.024581932 1288.334457285

8.840954284 -2.024581932 1323.577046290

8.946076189 -2.024581932 1339.057821973

9.051198095 -2.024581932 1344.649652709

9.156320000 -2.024581932 1346.289293841

-0.830261000 -1.884955592 1346.649501035

-0.725139095 -1.884955592 1346.068797746

-0.620017189 -1.884955592 1343.847568911

-0.514895284 -1.884955592 1335.832659162

-0.409773379 -1.884955592 1313.750313949

-0.304651474 -1.884955592 1265.682119814

-0.199529568 -1.884955592 1177.255040247

-0.094407663 -1.884955592 1037.501760410

0.010714242 -1.884955592 847.944291452

0.115836147 -1.884955592 629.525475072

0.220958053 -1.884955592 420.140644802

0.326079958 -1.884955592 260.004130403

0.431201863 -1.884955592 172.283630074

0.536323768 -1.884955592 152.368739653

0.641445674 -1.884955592 173.315561609

0.746567579 -1.884955592 201.053049638

0.851689484 -1.884955592 181.450574699

0.956811389 -1.884955592 168.435141833

1.061933295 -1.884955592 163.177221081

1.167055200 -1.884955592 162.526497450

1.272177105 -1.884955592 151.042461674

1.377299011 -1.884955592 128.427663109

1.482420916 -1.884955592 110.143532306

1.587542821 -1.884955592 103.544358645

1.692664726 -1.884955592 105.847286646

1.797786632 -1.884955592 112.365456889

1.902908537 -1.884955592 117.863681819

2.008030442 -1.884955592 119.323231429

2.113152347 -1.884955592 116.491002578

2.218274253 -1.884955592 111.336163160

2.323396158 -1.884955592 106.638413371

2.428518063 -1.884955592 104.998920208

2.533639968 -1.884955592 107.748545286

2.638761874 -1.884955592 114.691829040

2.743883779 -1.884955592 124.189461709

2.849005684 -1.884955592 134.180522131

2.954127589 -1.884955592 142.759545163

3.059249495 -1.884955592 148.488252006

3.164371400 -1.884955592 150.423907381

3.269493305 -1.884955592 148.484705954

3.374615211 -1.884955592 143.838036876

3.479737116 -1.884955592 138.602103402

3.584859021 -1.884955592 135.204089221

3.689980926 -1.884955592 135.299127316

3.795102832 -1.884955592 139.187765919

3.900224737 -1.884955592 145.941717168

4.005346642 -1.884955592 153.893986470

4.110468547 -1.884955592 161.173383626

4.215590453 -1.884955592 166.321291196

4.320712358 -1.884955592 168.332867025

4.425834263 -1.884955592 167.557606986

4.530956168 -1.884955592 165.508150839

4.636078074 -1.884955592 163.988146429

4.741199979 -1.884955592 164.238326917

4.846321884 -1.884955592 166.482425061

4.951443789 -1.884955592 170.057716479

5.056565695 -1.884955592 174.026067982

5.161687600 -1.884955592 177.586413227

5.266809505 -1.884955592 180.441876446

5.371931411 -1.884955592 182.775396843

5.477053316 -1.884955592 184.882041709

5.582175221 -1.884955592 186.786129993

5.687297126 -1.884955592 188.274370598

5.792419032 -1.884955592 189.477597202

5.897540937 -1.884955592 191.209787944

6.002662842 -1.884955592 194.020144972

6.107784747 -1.884955592 197.142760791

6.212906653 -1.884955592 200.822069299

6.318028558 -1.884955592 205.571150399

6.423150463 -1.884955592 211.114931025

6.528272368 -1.884955592 217.389652081

6.633394274 -1.884955592 224.896507576

6.738516179 -1.884955592 234.209542726

6.843638084 -1.884955592 241.715317703

6.948759989 -1.884955592 243.847919815

7.053881895 -1.884955592 244.214243187

7.159003800 -1.884955592 245.035756057

7.264125705 -1.884955592 249.626086315

7.369247611 -1.884955592 259.949577648

7.474369516 -1.884955592 272.853372200

7.579491421 -1.884955592 279.586004107

7.684613326 -1.884955592 272.168380556

7.789735232 -1.884955592 254.061609313

7.894857137 -1.884955592 246.102435388

7.999979042 -1.884955592 279.461469040

8.105100947 -1.884955592 377.662050815

8.210222853 -1.884955592 540.093297735

8.315344758 -1.884955592 739.970069708

8.420466663 -1.884955592 938.392210370

8.525588568 -1.884955592 1102.924901545

8.630710474 -1.884955592 1218.861131952

8.735832379 -1.884955592 1288.389487414

8.840954284 -1.884955592 1323.605357090

8.946076189 -1.884955592 1339.070200742

9.051198095 -1.884955592 1344.653714219

9.156320000 -1.884955592 1346.290455721

-0.830261000 -1.745329252 1346.647438127

-0.725139095 -1.745329252 1346.060552714

-0.620017189 -1.745329252 1343.815724178

-0.514895284 -1.745329252 1335.730339846

-0.409773379 -1.745329252 1313.533890811

-0.304651474 -1.745329252 1265.272975000

-0.199529568 -1.745329252 1176.515059856

-0.094407663 -1.745329252 1036.274417681

0.010714242 -1.745329252 846.058603190

0.115836147 -1.745329252 626.773026836

0.220958053 -1.745329252 416.275659827

0.326079958 -1.745329252 254.821652977

0.431201863 -1.745329252 165.671776310

0.536323768 -1.745329252 144.493084930

0.641445674 -1.745329252 164.931303342

0.746567579 -1.745329252 194.254645926

0.851689484 -1.745329252 198.465614204

0.956811389 -1.745329252 183.485740354

1.061933295 -1.745329252 175.218479726

1.167055200 -1.745329252 164.777140182

1.272177105 -1.745329252 144.450020184

1.377299011 -1.745329252 118.899033199

1.482420916 -1.745329252 103.357255991

1.587542821 -1.745329252 100.291091204

1.692664726 -1.745329252 106.455259361

1.797786632 -1.745329252 116.631097922

1.902908537 -1.745329252 125.104248532

2.008030442 -1.745329252 128.521947375

2.113152347 -1.745329252 126.461947279

2.218274253 -1.745329252 120.875856185

2.323396158 -1.745329252 114.774487066

2.428518063 -1.745329252 111.250312926

2.533639968 -1.745329252 112.205976516

2.638761874 -1.745329252 117.818305155

2.743883779 -1.745329252 126.543341031

2.849005684 -1.745329252 136.183426828

2.954127589 -1.745329252 144.592070143

3.059249495 -1.745329252 150.225835245

3.164371400 -1.745329252 152.186350748

3.269493305 -1.745329252 150.439309779

3.374615211 -1.745329252 146.130514582

3.479737116 -1.745329252 141.280088310

3.584859021 -1.745329252 138.228132536

3.689980926 -1.745329252 138.631844932

3.795102832 -1.745329252 142.826835599

3.900224737 -1.745329252 149.788330988

4.005346642 -1.745329252 157.515520073

4.110468547 -1.745329252 164.184970097

4.215590453 -1.745329252 168.559852005

4.320712358 -1.745329252 169.503981455

4.425834263 -1.745329252 167.785045719

4.530956168 -1.745329252 165.154537433

4.636078074 -1.745329252 163.407411804

4.741199979 -1.745329252 163.684042895

4.846321884 -1.745329252 166.109282751

4.951443789 -1.745329252 169.979863183

5.056565695 -1.745329252 174.306469778

5.161687600 -1.745329252 178.206112515

5.266809505 -1.745329252 181.302000022

5.371931411 -1.745329252 183.735211353

5.477053316 -1.745329252 185.801790734

5.582175221 -1.745329252 187.556556944

5.687297126 -1.745329252 188.795043091

5.792419032 -1.745329252 189.601921383

5.897540937 -1.745329252 190.665696079

6.002662842 -1.745329252 192.412088338

6.107784747 -1.745329252 194.705560297

6.212906653 -1.745329252 198.119041414

6.318028558 -1.745329252 202.727891855

6.423150463 -1.745329252 208.122493705

6.528272368 -1.745329252 214.244947664

6.633394274 -1.745329252 221.657307775

6.738516179 -1.745329252 231.202422836

6.843638084 -1.745329252 240.596285159

6.948759989 -1.745329252 243.447258860

7.053881895 -1.745329252 244.040885027

7.159003800 -1.745329252 245.008179021

7.264125705 -1.745329252 249.650562465

7.369247611 -1.745329252 259.958201885

7.474369516 -1.745329252 272.841948487

7.579491421 -1.745329252 279.617280338

7.684613326 -1.745329252 272.347119390

7.789735232 -1.745329252 254.477286067

7.894857137 -1.745329252 246.766541106

7.999979042 -1.745329252 280.287144361

8.105100947 -1.745329252 378.508825845

8.210222853 -1.745329252 540.835242841

8.315344758 -1.745329252 740.535146255

8.420466663 -1.745329252 938.769662800

8.525588568 -1.745329252 1103.148999525

8.630710474 -1.745329252 1218.977578357

8.735832379 -1.745329252 1288.446657123

8.840954284 -1.745329252 1323.635037552

8.946076189 -1.745329252 1339.083188663

9.051198095 -1.745329252 1344.658079776

9.156320000 -1.745329252 1346.291660383

-0.830261000 -1.605702912 1346.644484000

-0.725139095 -1.605702912 1346.048595008

-0.620017189 -1.605702912 1343.770119831

-0.514895284 -1.605702912 1335.593296449

-0.409773379 -1.605702912 1313.263799601

-0.304651474 -1.605702912 1264.778756634

-0.199529568 -1.605702912 1175.616104210

-0.094407663 -1.605702912 1034.765800762

0.010714242 -1.605702912 843.731370029

0.115836147 -1.605702912 623.400100139

0.220958053 -1.605702912 411.614706556

0.326079958 -1.605702912 248.674814592

0.431201863 -1.605702912 157.792903764

0.536323768 -1.605702912 134.757542437

0.641445674 -1.605702912 153.779941745

0.746567579 -1.605702912 182.883295947

0.851689484 -1.605702912 198.674309617

0.956811389 -1.605702912 193.793156787

1.061933295 -1.605702912 177.922008106

1.167055200 -1.605702912 156.769648082

1.272177105 -1.605702912 128.123467330

1.377299011 -1.605702912 104.384200338

1.482420916 -1.605702912 92.242444925

1.587542821 -1.605702912 93.116106108

1.692664726 -1.605702912 103.408733607

1.797786632 -1.605702912 117.505269905

1.902908537 -1.605702912 129.301354418

2.008030442 -1.605702912 135.150635571

2.113152347 -1.605702912 134.634488996

2.218274253 -1.605702912 129.764329023

2.323396158 -1.605702912 123.629452392

2.428518063 -1.605702912 119.524366803

2.533639968 -1.605702912 119.498683477

2.638761874 -1.605702912 123.829418737

2.743883779 -1.605702912 131.114793294

2.849005684 -1.605702912 139.371133098

2.954127589 -1.605702912 146.704337699

3.059249495 -1.605702912 151.787697331

3.164371400 -1.605702912 153.742536039

3.269493305 -1.605702912 152.406493172

3.374615211 -1.605702912 148.731511683

3.479737116 -1.605702912 144.528853591

3.584859021 -1.605702912 141.997051997

3.689980926 -1.605702912 142.766911625

3.795102832 -1.605702912 147.208687145

3.900224737 -1.605702912 154.172278366

4.005346642 -1.605702912 161.299874682

4.110468547 -1.605702912 167.417553735

4.215590453 -1.605702912 171.245427466

4.320712358 -1.605702912 171.244922980

4.425834263 -1.605702912 168.700474824

4.530956168 -1.605702912 165.586326663

4.636078074 -1.605702912 163.664886257

4.741199979 -1.605702912 163.961403253

4.846321884 -1.605702912 166.497966907

4.951443789 -1.605702912 170.541338572

5.056565695 -1.605702912 175.072731584

5.161687600 -1.605702912 179.155904387

5.266809505 -1.605702912 182.360649668

5.371931411 -1.605702912 184.787675772

5.477053316 -1.605702912 186.704847033

5.582175221 -1.605702912 188.178709453

5.687297126 -1.605702912 189.024053158

5.792419032 -1.605702912 189.305525234

5.897540937 -1.605702912 189.661090507

6.002662842 -1.605702912 190.665816993

6.107784747 -1.605702912 192.628717148

6.212906653 -1.605702912 195.954487255

6.318028558 -1.605702912 200.436765135

6.423150463 -1.605702912 205.607050844

6.528272368 -1.605702912 211.438621344

6.633394274 -1.605702912 218.582029178

6.738516179 -1.605702912 228.083455895

6.843638084 -1.605702912 239.144531983

6.948759989 -1.605702912 243.246373451

7.053881895 -1.605702912 243.999870334

7.159003800 -1.605702912 245.090526387

7.264125705 -1.605702912 249.803890404

7.369247611 -1.605702912 260.127153028

7.474369516 -1.605702912 273.002940841

7.579491421 -1.605702912 279.796472356

7.684613326 -1.605702912 272.612003771

7.789735232 -1.605702912 254.893907218

7.894857137 -1.605702912 247.345699057

7.999979042 -1.605702912 280.965986441

8.105100947 -1.605702912 379.184460903

8.210222853 -1.605702912 541.419316566

8.315344758 -1.605702912 740.979163074

8.420466663 -1.605702912 939.068977541

8.525588568 -1.605702912 1103.330287641

8.630710474 -1.605702912 1219.075056964

8.735832379 -1.605702912 1288.497782264

8.840954284 -1.605702912 1323.662995809

8.946076189 -1.605702912 1339.095595907

9.051198095 -1.605702912 1344.662360755

9.156320000 -1.605702912 1346.292795450

-0.830261000 -1.466076572 1346.640707993

-0.725139095 -1.466076572 1346.033127097

-0.620017189 -1.466076572 1343.709906014

-0.514895284 -1.466076572 1335.411603633

-0.409773379 -1.466076572 1312.901837692

-0.304651474 -1.466076572 1264.116759908

-0.199529568 -1.466076572 1174.414833108

-0.094407663 -1.466076572 1032.763827469

0.010714242 -1.466076572 840.713465944

0.115836147 -1.466076572 619.234492132

0.220958053 -1.466076572 406.267314988

0.326079958 -1.466076572 242.167216354

0.431201863 -1.466076572 149.854284255

0.536323768 -1.466076572 124.858703264

0.641445674 -1.466076572 141.588025804

0.746567579 -1.466076572 169.573258900

0.851689484 -1.466076572 185.596152091

0.956811389 -1.466076572 182.122540473

1.061933295 -1.466076572 164.010608571

1.167055200 -1.466076572 136.030257199

1.272177105 -1.466076572 108.084516663

1.377299011 -1.466076572 87.787062928

1.482420916 -1.466076572 79.750566375

1.587542821 -1.466076572 84.840313064

1.692664726 -1.466076572 99.164304113

1.797786632 -1.466076572 116.943086723

1.902908537 -1.466076572 131.885051636

2.008030442 -1.466076572 139.497461436

2.113152347 -1.466076572 139.274879742

2.218274253 -1.466076572 136.961602259

2.323396158 -1.466076572 132.597099024

2.428518063 -1.466076572 129.049545542

2.533639968 -1.466076572 128.779701986

2.638761874 -1.466076572 131.934606666

2.743883779 -1.466076572 137.199673853

2.849005684 -1.466076572 143.061518000

2.954127589 -1.466076572 148.398587880

3.059249495 -1.466076572 152.372395078

3.164371400 -1.466076572 154.196449225

3.269493305 -1.466076572 153.534097850

3.374615211 -1.466076572 150.980756967

3.479737116 -1.466076572 147.961663200

3.584859021 -1.466076572 146.387632731

3.689980926 -1.466076572 147.755435040

3.795102832 -1.466076572 152.414898441

3.900224737 -1.466076572 159.016582487

4.005346642 -1.466076572 165.142088890

4.110468547 -1.466076572 170.753108451

4.215590453 -1.466076572 174.216707819

4.320712358 -1.466076572 173.459248420

4.425834263 -1.466076572 170.257051774

4.530956168 -1.466076572 166.781513838

4.636078074 -1.466076572 164.740988527

4.741199979 -1.466076572 165.041565398

4.846321884 -1.466076572 167.605976840

4.951443789 -1.466076572 171.676616546

5.056565695 -1.466076572 176.221858702

5.161687600 -1.466076572 180.279753960

5.266809505 -1.466076572 183.407477604

5.371931411 -1.466076572 185.687960033

5.477053316 -1.466076572 187.335805799

5.582175221 -1.466076572 188.408349211

5.687297126 -1.466076572 188.767074994

5.792419032 -1.466076572 188.529294353

5.897540937 -1.466076572 188.397392363

6.002662842 -1.466076572 189.112722930

6.107784747 -1.466076572 191.053517220

6.212906653 -1.466076572 194.388641247

6.318028558 -1.466076572 198.763770739

6.423150463 -1.466076572 203.675859960

6.528272368 -1.466076572 209.127753262

6.633394274 -1.466076572 215.866009221

6.738516179 -1.466076572 225.105472084

6.843638084 -1.466076572 237.096697845

6.948759989 -1.466076572 243.326218668

7.053881895 -1.466076572 244.216087477

7.159003800 -1.466076572 245.416645591

7.264125705 -1.466076572 250.218074816

7.369247611 -1.466076572 260.587957831

7.474369516 -1.466076572 273.475495921

7.579491421 -1.466076572 280.273833863

7.684613326 -1.466076572 273.115117713

7.789735232 -1.466076572 255.445743000

7.894857137 -1.466076572 247.936973508

7.999979042 -1.466076572 281.548620481

8.105100947 -1.466076572 379.697232799

8.210222853 -1.466076572 541.825427040

8.315344758 -1.466076572 741.270378069

8.420466663 -1.466076572 939.259630625

8.525588568 -1.466076572 1103.446073414

8.630710474 -1.466076572 1219.139855500

8.735832379 -1.466076572 1288.536824281

8.840954284 -1.466076572 1323.686756644

8.946076189 -1.466076572 1339.106367796

9.051198095 -1.466076572 1344.666181021

9.156320000 -1.466076572 1346.293738640

-0.830261000 -1.326450232 1346.636324022

-0.725139095 -1.326450232 1346.015067217

-0.620017189 -1.326450232 1343.638054325

-0.514895284 -1.326450232 1335.191548101

-0.409773379 -1.326450232 1312.451484342

-0.304651474 -1.326450232 1263.291924363

-0.199529568 -1.326450232 1172.938523478

-0.094407663 -1.326450232 1030.340910543

0.010714242 -1.326450232 837.158186324

0.115836147 -1.326450232 614.583324074

0.220958053 -1.326450232 400.830097925

0.326079958 -1.326450232 236.296603505

0.431201863 -1.326450232 143.138703800

0.536323768 -1.326450232 115.510617017

0.641445674 -1.326450232 130.004680030

0.746567579 -1.326450232 156.140311730

0.851689484 -1.326450232 169.757362921

0.956811389 -1.326450232 162.693791052

1.061933295 -1.326450232 139.930008229

1.167055200 -1.326450232 111.443386490

1.272177105 -1.326450232 86.216858440

1.377299011 -1.326450232 70.467737374

1.482420916 -1.326450232 67.538848322

1.587542821 -1.326450232 77.369520781

1.692664726 -1.326450232 95.731411914

1.797786632 -1.326450232 116.874066277

1.902908537 -1.326450232 134.552350447

2.008030442 -1.326450232 140.837005591

2.113152347 -1.326450232 137.204399162

2.218274253 -1.326450232 135.378349553

2.323396158 -1.326450232 135.599440032

2.428518063 -1.326450232 136.324487175

2.533639968 -1.326450232 137.299706547

2.638761874 -1.326450232 139.554413097

2.743883779 -1.326450232 142.666619221

2.849005684 -1.326450232 146.029850235

2.954127589 -1.326450232 149.114168110

3.059249495 -1.326450232 151.642250838

3.164371400 -1.326450232 153.298584074

3.269493305 -1.326450232 153.619139350

3.374615211 -1.326450232 152.713506813

3.479737116 -1.326450232 151.452643123

3.584859021 -1.326450232 151.302527248

3.689980926 -1.326450232 153.502945707

3.795102832 -1.326450232 158.301203566

3.900224737 -1.326450232 164.085398598

4.005346642 -1.326450232 168.978321894

4.110468547 -1.326450232 173.967560181

4.215590453 -1.326450232 177.188315945

4.320712358 -1.326450232 175.934521300

4.425834263 -1.326450232 172.250849998

4.530956168 -1.326450232 168.522103811

4.636078074 -1.326450232 166.398999871

4.741199979 -1.326450232 166.678910903

4.846321884 -1.326450232 169.194390275

4.951443789 -1.326450232 173.159548972

5.056565695 -1.326450232 177.533418193

5.161687600 -1.326450232 181.348461322

5.266809505 -1.326450232 184.191466025

5.371931411 -1.326450232 186.169745290

5.477053316 -1.326450232 187.453149847

5.582175221 -1.326450232 188.087537183

5.687297126 -1.326450232 188.011988469

5.792419032 -1.326450232 187.441520666

5.897540937 -1.326450232 187.148592802

6.002662842 -1.326450232 187.912865137

6.107784747 -1.326450232 189.995777082

6.212906653 -1.326450232 193.386798649

6.318028558 -1.326450232 197.675743491

6.423150463 -1.326450232 202.333461172

6.528272368 -1.326450232 207.371282150

6.633394274 -1.326450232 213.614898024

6.738516179 -1.326450232 222.426878210

6.843638084 -1.326450232 234.596864269

6.948759989 -1.326450232 243.676818549

7.053881895 -1.326450232 244.790526132

7.159003800 -1.326450232 246.096822223

7.264125705 -1.326450232 250.990066897

7.369247611 -1.326450232 261.418185468

7.474369516 -1.326450232 274.327617660

7.579491421 -1.326450232 281.122523698

7.684613326 -1.326450232 273.942448817

7.789735232 -1.326450232 256.227287047

7.894857137 -1.326450232 248.631024217

7.999979042 -1.326450232 282.109124506

8.105100947 -1.326450232 380.097118630

8.210222853 -1.326450232 542.078984603

8.315344758 -1.326450232 741.415417880

8.420466663 -1.326450232 939.337625824

8.525588568 -1.326450232 1103.489096786

8.630710474 -1.326450232 1219.165518034

8.735832379 -1.326450232 1288.560186023

8.840954284 -1.326450232 1323.704584311

8.946076189 -1.326450232 1339.114693750

9.051198095 -1.326450232 1344.669233521

9.156320000 -1.326450232 1346.294383922

-0.830261000 -1.186823891 1346.631682306

-0.725139095 -1.186823891 1345.996006208

-0.620017189 -1.186823891 1343.561384869

-0.514895284 -1.186823891 1334.955539086

-0.409773379 -1.186823891 1311.956607059

-0.304651474 -1.186823891 1262.380867601

-0.199529568 -1.186823891 1171.332601051

-0.094407663 -1.186823891 1027.738785480

0.010714242 -1.186823891 833.408918391

0.115836147 -1.186823891 609.882348410

0.220958053 -1.186823891 395.814759155

0.326079958 -1.186823891 231.439317670

0.431201863 -1.186823891 135.682204946

0.536323768 -1.186823891 105.674309440

0.641445674 -1.186823891 119.223184781

0.746567579 -1.186823891 142.412481104

0.851689484 -1.186823891 151.003796734

0.956811389 -1.186823891 138.637129855

1.061933295 -1.186823891 112.499293271

1.167055200 -1.186823891 83.939168935

1.272177105 -1.186823891 62.064552488

1.377299011 -1.186823891 51.935571756

1.482420916 -1.186823891 55.211482173

1.587542821 -1.186823891 70.635239439

1.692664726 -1.186823891 93.476355411

1.797786632 -1.186823891 117.984645435

1.902908537 -1.186823891 138.117238778

2.008030442 -1.186823891 141.653758816

2.113152347 -1.186823891 136.112135608

2.218274253 -1.186823891 133.147149763

2.323396158 -1.186823891 133.145307529

2.428518063 -1.186823891 135.233255658

2.533639968 -1.186823891 138.342111993

2.638761874 -1.186823891 141.827980913

2.743883779 -1.186823891 145.284075976

2.849005684 -1.186823891 147.800385897

2.954127589 -1.186823891 148.816615763

3.059249495 -1.186823891 149.885830849

3.164371400 -1.186823891 151.496397614

3.269493305 -1.186823891 153.026210598

3.374615211 -1.186823891 154.067248160

3.479737116 -1.186823891 154.875466118

3.584859021 -1.186823891 156.391028819

3.689980926 -1.186823891 159.517132017

3.795102832 -1.186823891 164.303807463

3.900224737 -1.186823891 168.855836226

4.005346642 -1.186823891 172.422357888

4.110468547 -1.186823891 176.618181182

4.215590453 -1.186823891 179.707850307

4.320712358 -1.186823891 178.345709072

4.425834263 -1.186823891 174.364238387

4.530956168 -1.186823891 170.476769924

4.636078074 -1.186823891 168.302312171

4.741199979 -1.186823891 168.546338082

4.846321884 -1.186823891 170.960649658

4.951443789 -1.186823891 174.721052006

5.056565695 -1.186823891 178.776355832

5.161687600 -1.186823891 182.179997625

5.266809505 -1.186823891 184.584284693

5.371931411 -1.186823891 186.151876515

5.477053316 -1.186823891 187.043265743

5.582175221 -1.186823891 187.307224600

5.687297126 -1.186823891 186.954552880

5.792419032 -1.186823891 186.274330367

5.897540937 -1.186823891 186.069174987

6.002662842 -1.186823891 187.083759486

6.107784747 -1.186823891 189.386293518

6.212906653 -1.186823891 192.832446798

6.318028558 -1.186823891 197.040636575

6.423150463 -1.186823891 201.467664564

6.528272368 -1.186823891 206.104133735

6.633394274 -1.186823891 211.817722520

6.738516179 -1.186823891 220.088948970

6.843638084 -1.186823891 231.987936683

6.948759989 -1.186823891 243.989409168

7.053881895 -1.186823891 245.731248607

7.159003800 -1.186823891 247.151498186

7.264125705 -1.186823891 252.132567110

7.369247611 -1.186823891 262.608139697

7.474369516 -1.186823891 275.524009811

7.579491421 -1.186823891 282.290085098

7.684613326 -1.186823891 275.039483418

7.789735232 -1.186823891 257.195750672

7.894857137 -1.186823891 249.403438200

7.999979042 -1.186823891 282.640285286

8.105100947 -1.186823891 380.387845887

8.210222853 -1.186823891 542.186421057

8.315344758 -1.186823891 741.417914590

8.420466663 -1.186823891 939.302447747

8.525588568 -1.186823891 1103.456381337

8.630710474 -1.186823891 1219.148387965

8.735832379 -1.186823891 1288.565551155

8.840954284 -1.186823891 1323.715564672

8.946076189 -1.186823891 1339.120185386

9.051198095 -1.186823891 1344.671365453

9.156320000 -1.186823891 1346.294675453

-0.830261000 -1.047197551 1346.627156012

-0.725139095 -1.047197551 1345.977659107

-0.620017189 -1.047197551 1343.487864466

-0.514895284 -1.047197551 1334.730983782

-0.409773379 -1.047197551 1311.475028820

-0.304651474 -1.047197551 1261.475407623

-0.199529568 -1.047197551 1169.738062823

-0.094407663 -1.047197551 1025.152984192

0.010714242 -1.047197551 829.683420850

0.115836147 -1.047197551 605.294556911

0.220958053 -1.047197551 391.166224873

0.326079958 -1.047197551 226.915307647

0.431201863 -1.047197551 128.618167848

0.536323768 -1.047197551 97.783723521

0.641445674 -1.047197551 109.188185311

0.746567579 -1.047197551 127.981214929

0.851689484 -1.047197551 130.770775132

0.956811389 -1.047197551 112.893676193

1.061933295 -1.047197551 83.304585526

1.167055200 -1.047197551 54.690288863

1.272177105 -1.047197551 36.327034958

1.377299011 -1.047197551 32.105181160

1.482420916 -1.047197551 42.053808149

1.587542821 -1.047197551 63.746619865

1.692664726 -1.047197551 91.645419349

1.797786632 -1.047197551 119.704969414

1.902908537 -1.047197551 142.166273359

2.008030442 -1.047197551 144.262022949

2.113152347 -1.047197551 137.736624213

2.218274253 -1.047197551 133.731025113

2.323396158 -1.047197551 132.927671032

2.428518063 -1.047197551 134.688536675

2.533639968 -1.047197551 138.033960526

2.638761874 -1.047197551 142.218765230

2.743883779 -1.047197551 146.600624198

2.849005684 -1.047197551 148.973136358

2.954127589 -1.047197551 148.112187705

3.059249495 -1.047197551 147.988779540

3.164371400 -1.047197551 149.608563378

3.269493305 -1.047197551 152.237588064

3.374615211 -1.047197551 155.067823175

3.479737116 -1.047197551 157.823103659

3.584859021 -1.047197551 160.937160140

3.689980926 -1.047197551 164.943625752

3.795102832 -1.047197551 169.555384122

3.900224737 -1.047197551 172.614641925

4.005346642 -1.047197551 174.890793196

4.110468547 -1.047197551 178.214994466

4.215590453 -1.047197551 181.199521674

4.320712358 -1.047197551 180.261448350

4.425834263 -1.047197551 176.237243424

4.530956168 -1.047197551 172.316830150

4.636078074 -1.047197551 170.159472786

4.741199979 -1.047197551 170.388962559

4.846321884 -1.047197551 172.681509960

4.951443789 -1.047197551 176.169731070

5.056565695 -1.047197551 179.803796115

5.161687600 -1.047197551 182.701893525

5.266809505 -1.047197551 184.610917092

5.371931411 -1.047197551 185.747584362

5.477053316 -1.047197551 186.284238677

5.582175221 -1.047197551 186.285791638

5.687297126 -1.047197551 185.813889983

5.792419032 -1.047197551 185.194964074

5.897540937 -1.047197551 185.226760595

6.002662842 -1.047197551 186.581852224

6.107784747 -1.047197551 189.098833279

6.212906653 -1.047197551 192.544839607

6.318028558 -1.047197551 196.648511632

6.423150463 -1.047197551 200.868037942

6.528272368 -1.047197551 205.149624339

6.633394274 -1.047197551 210.359002770

6.738516179 -1.047197551 218.050223311

6.843638084 -1.047197551 229.472833516

6.948759989 -1.047197551 243.468947302

7.053881895 -1.047197551 246.940594021

7.159003800 -1.047197551 248.500432152

7.264125705 -1.047197551 253.571486567

7.369247611 -1.047197551 264.068287273

7.474369516 -1.047197551 276.937277203

7.579491421 -1.047197551 283.603395788

7.684613326 -1.047197551 276.202112952

7.789735232 -1.047197551 258.146815021

7.894857137 -1.047197551 250.080777793

7.999979042 -1.047197551 283.018379820

8.105100947 -1.047197551 380.497915794

8.210222853 -1.047197551 542.116480365

8.315344758 -1.047197551 741.269722834

8.420466663 -1.047197551 939.155227511

8.525588568 -1.047197551 1103.350660203

8.630710474 -1.047197551 1219.089873726

8.735832379 -1.047197551 1288.553281613

8.840954284 -1.047197551 1323.720030755

8.946076189 -1.047197551 1339.122989981

9.051198095 -1.047197551 1344.672616705

9.156320000 -1.047197551 1346.294624739

-0.830261000 -0.907571211 1346.623086788

-0.725139095 -0.907571211 1345.961490457

-0.620017189 -0.907571211 1343.424176032

-0.514895284 -0.907571211 1334.539438442

-0.409773379 -0.907571211 1311.052730391

-0.304651474 -0.907571211 1260.646027125

-0.199529568 -0.907571211 1168.240475209

-0.094407663 -0.907571211 1022.668285684

0.010714242 -0.907571211 826.020882295

0.115836147 -0.907571211 600.702299673

0.220958053 -0.907571211 386.433747779

0.326079958 -0.907571211 223.524357027

0.431201863 -0.907571211 125.765594266

0.536323768 -0.907571211 92.839144130

0.641445674 -0.907571211 100.503215562

0.746567579 -0.907571211 114.396494823

0.851689484 -0.907571211 112.008637099

0.956811389 -0.907571211 89.684779589

1.061933295 -0.907571211 57.466356157

1.167055200 -0.907571211 28.897197883

1.272177105 -0.907571211 13.365439806

1.377299011 -0.907571211 14.028873136

1.482420916 -0.907571211 29.899609124

1.587542821 -0.907571211 57.457780816

1.692664726 -0.907571211 90.157218638

1.797786632 -0.907571211 121.415354592

1.902908537 -0.907571211 145.825376872

2.008030442 -0.907571211 148.664773731

2.113152347 -0.907571211 141.935657343

2.218274253 -0.907571211 137.236509835

2.323396158 -0.907571211 135.463263406

2.428518063 -0.907571211 136.319750503

2.533639968 -0.907571211 139.147917088

2.638761874 -0.907571211 143.328628494

2.743883779 -0.907571211 148.108681585

2.849005684 -0.907571211 150.347119529

2.954127589 -0.907571211 148.016123158

3.059249495 -0.907571211 146.843415891

3.164371400 -0.907571211 148.175104135

3.269493305 -0.907571211 151.341630026

3.374615211 -0.907571211 155.358231040

3.479737116 -0.907571211 159.588340543

3.584859021 -0.907571211 164.034526152

3.689980926 -0.907571211 168.851133703

3.795102832 -0.907571211 173.249173438

3.900224737 -0.907571211 174.883983920

4.005346642 -0.907571211 176.062385515

4.110468547 -0.907571211 178.572741202

4.215590453 -0.907571211 181.335945321

4.320712358 -0.907571211 181.222312391

4.425834263 -0.907571211 177.566358519

4.530956168 -0.907571211 173.826521795

4.636078074 -0.907571211 171.829418914

4.741199979 -0.907571211 172.117666049

4.846321884 -0.907571211 174.292860409

4.951443789 -0.907571211 177.455301809

5.056565695 -0.907571211 180.590972688

5.161687600 -0.907571211 182.938708542

5.266809505 -0.907571211 184.360746045

5.371931411 -0.907571211 185.106298577

5.477053316 -0.907571211 185.360942586

5.582175221 -0.907571211 185.219531285

5.687297126 -0.907571211 184.780999275

5.792419032 -0.907571211 184.366448487

5.897540937 -0.907571211 184.713893217

6.002662842 -0.907571211 186.377765097

6.107784747 -0.907571211 188.986333076

6.212906653 -0.907571211 192.326988427

6.318028558 -0.907571211 196.280286988

6.423150463 -0.907571211 200.303952676

6.528272368 -0.907571211 204.291176478

6.633394274 -0.907571211 209.073246617

6.738516179 -0.907571211 216.229590626

6.843638084 -0.907571211 227.130053103

6.948759989 -0.907571211 241.736387522

7.053881895 -0.907571211 248.288117351

7.159003800 -0.907571211 250.029496203

7.264125705 -0.907571211 255.192419790

7.369247611 -0.907571211 265.661283999

7.474369516 -0.907571211 278.375768360

7.579491421 -0.907571211 284.799408701

7.684613326 -0.907571211 277.110333741

7.789735232 -0.907571211 258.746711746

7.894857137 -0.907571211 250.366955975

7.999979042 -0.907571211 283.022493030

8.105100947 -0.907571211 380.293504952

8.210222853 -0.907571211 541.808723590

8.315344758 -0.907571211 740.957043680

8.420466663 -0.907571211 938.903487029

8.525588568 -0.907571211 1103.183964648

8.630710474 -0.907571211 1218.999009283

8.735832379 -0.907571211 1288.527492582

8.840954284 -0.907571211 1323.719535915

8.946076189 -0.907571211 1339.123667754

9.051198095 -0.907571211 1344.673169260

9.156320000 -0.907571211 1346.294304535

-0.830261000 -0.767944871 1346.619821126

-0.725139095 -0.767944871 1345.948758524

-0.620017189 -0.767944871 1343.375496004

-0.514895284 -0.767944871 1334.395306556

-0.409773379 -0.767944871 1310.719119308

-0.304651474 -0.767944871 1259.942669039

-0.199529568 -0.767944871 1166.903295143

-0.094407663 -0.767944871 1020.350112892

0.010714242 -0.767944871 822.458648775

0.115836147 -0.767944871 596.013268695

0.220958053 -0.767944871 381.245925330

0.326079958 -0.767944871 219.601496821

0.431201863 -0.767944871 126.377236468

0.536323768 -0.767944871 90.792189206

0.641445674 -0.767944871 94.580231933

0.746567579 -0.767944871 104.800574056

0.851689484 -0.767944871 99.530875106

0.956811389 -0.767944871 75.250588905

1.061933295 -0.767944871 42.077945784

1.167055200 -0.767944871 13.800772425

1.272177105 -0.767944871 0.000000000

1.377299011 -0.767944871 3.736134149

1.482420916 -0.767944871 23.454483283

1.587542821 -0.767944871 54.754812599

1.692664726 -0.767944871 90.397446974

1.797786632 -0.767944871 123.373531022

1.902908537 -0.767944871 148.650131879

2.008030442 -0.767944871 153.933452404

2.113152347 -0.767944871 147.660075856

2.218274253 -0.767944871 142.720132444

2.323396158 -0.767944871 140.065918808

2.428518063 -0.767944871 139.751888728

2.533639968 -0.767944871 141.596774525

2.638761874 -0.767944871 145.282340784

2.743883779 -0.767944871 150.059393657

2.849005684 -0.767944871 152.534464867

2.954127589 -0.767944871 149.093153184

3.059249495 -0.767944871 146.639644205

3.164371400 -0.767944871 147.062130311

3.269493305 -0.767944871 149.963761573

3.374615211 -0.767944871 154.401346488

3.479737116 -0.767944871 159.533546789

3.584859021 -0.767944871 165.004070021

3.689980926 -0.767944871 170.603656949

3.795102832 -0.767944871 175.026587119

3.900224737 -0.767944871 175.683835451

4.005346642 -0.767944871 176.049758431

4.110468547 -0.767944871 177.901815914

4.215590453 -0.767944871 180.359540514

4.320712358 -0.767944871 180.993982482

4.425834263 -0.767944871 178.166390026

4.530956168 -0.767944871 174.918926131

4.636078074 -0.767944871 173.293156710

4.741199979 -0.767944871 173.750198123

4.846321884 -0.767944871 175.822405286

4.951443789 -0.767944871 178.609850229

5.056565695 -0.767944871 181.191306259

5.161687600 -0.767944871 182.980781575

5.266809505 -0.767944871 183.961167592

5.371931411 -0.767944871 184.391492903

5.477053316 -0.767944871 184.467420572

5.582175221 -0.767944871 184.319961670

5.687297126 -0.767944871 184.062733843

5.792419032 -0.767944871 183.960146762

5.897540937 -0.767944871 184.624456066

6.002662842 -0.767944871 186.427148801

6.107784747 -0.767944871 188.885246086

6.212906653 -0.767944871 192.000500410

6.318028558 -0.767944871 195.757330079

6.423150463 -0.767944871 199.586603162

6.528272368 -0.767944871 203.337575517

6.633394274 -0.767944871 207.797512354

6.738516179 -0.767944871 214.534806978

6.843638084 -0.767944871 224.985345282

6.948759989 -0.767944871 239.498053872

7.053881895 -0.767944871 249.626006177

7.159003800 -0.767944871 251.637200888

7.264125705 -0.767944871 256.864450652

7.369247611 -0.767944871 267.210794700

7.474369516 -0.767944871 279.598155473

7.579491421 -0.767944871 285.565090986

7.684613326 -0.767944871 277.399815564

7.789735232 -0.767944871 258.626293817

7.894857137 -0.767944871 249.943240622

7.999979042 -0.767944871 282.424886824

8.105100947 -0.767944871 379.648830533

8.210222853 -0.767944871 541.221080122

8.315344758 -0.767944871 740.488620146

8.420466663 -0.767944871 938.575734464

8.525588568 -0.767944871 1102.984262030

8.630710474 -0.767944871 1218.895072834

8.735832379 -0.767944871 1288.496645943

8.840954284 -0.767944871 1323.716602054

8.946076189 -0.767944871 1339.123016277

9.051198095 -0.767944871 1344.673278705

9.156320000 -0.767944871 1346.293832779

-0.830261000 -0.628318531 1346.617720151

-0.725139095 -0.628318531 1345.940619319

-0.620017189 -0.628318531 1343.346476725

-0.514895284 -0.628318531 1334.311901747

-0.409773379 -0.628318531 1310.502241326

-0.304651474 -0.628318531 1259.421340667

-0.199529568 -0.628318531 1165.822506874

-0.094407663 -0.628318531 1018.348486836

0.010714242 -0.628318531 819.200281800

0.115836147 -0.628318531 591.430181228

0.220958053 -0.628318531 375.708616719

0.326079958 -0.628318531 214.228135005

0.431201863 -0.628318531 127.472275018

0.536323768 -0.628318531 91.482690541

0.641445674 -0.628318531 92.800753117

0.746567579 -0.628318531 101.901004716

0.851689484 -0.628318531 96.954842656

0.956811389 -0.628318531 73.689901361

1.061933295 -0.628318531 41.582536468

1.167055200 -0.628318531 14.257933562

1.272177105 -0.628318531 1.424237978

1.377299011 -0.628318531 6.213503114

1.482420916 -0.628318531 26.858559972

1.587542821 -0.628318531 58.636193838

1.692664726 -0.628318531 94.188233079

1.797786632 -0.628318531 126.523604837

1.902908537 -0.628318531 150.963617783

2.008030442 -0.628318531 158.609484945

2.113152347 -0.628318531 153.194436053

2.218274253 -0.628318531 148.400376010

2.323396158 -0.628318531 145.179592291

2.428518063 -0.628318531 143.838313891

2.533639968 -0.628318531 144.672054961

2.638761874 -0.628318531 147.722298466

2.743883779 -0.628318531 152.305994530

2.849005684 -0.628318531 155.293827606

2.954127589 -0.628318531 150.830483692

3.059249495 -0.628318531 146.697886115

3.164371400 -0.628318531 145.635166445

3.269493305 -0.628318531 147.650108407

3.374615211 -0.628318531 151.946370466

3.479737116 -0.628318531 157.545067851

3.584859021 -0.628318531 163.773651695

3.689980926 -0.628318531 170.107023153

3.795102832 -0.628318531 174.982898175

3.900224737 -0.628318531 175.318855377

4.005346642 -0.628318531 175.184308190

4.110468547 -0.628318531 176.581704192

4.215590453 -0.628318531 178.782443881

4.320712358 -0.628318531 179.857323331

4.425834263 -0.628318531 178.028765952

4.530956168 -0.628318531 175.570022187

4.636078074 -0.628318531 174.515823337

4.741199979 -0.628318531 175.233742751

4.846321884 -0.628318531 177.206370926

4.951443789 -0.628318531 179.584305550

5.056565695 -0.628318531 181.604812873

5.161687600 -0.628318531 182.888209430

5.266809505 -0.628318531 183.519896844

5.371931411 -0.628318531 183.753092292

5.477053316 -0.628318531 183.785190419

5.582175221 -0.628318531 183.766116196

5.687297126 -0.628318531 183.792682434

5.792419032 -0.628318531 184.035781801

5.897540937 -0.628318531 184.926290399

6.002662842 -0.628318531 186.596912765

6.107784747 -0.628318531 188.668945927

6.212906653 -0.628318531 191.499487365

6.318028558 -0.628318531 195.043352138

6.423150463 -0.628318531 198.682226059

6.528272368 -0.628318531 202.241553601

6.633394274 -0.628318531 206.477390045

6.738516179 -0.628318531 212.935191448

6.843638084 -0.628318531 223.073444960

6.948759989 -0.628318531 237.373336625

7.053881895 -0.628318531 250.693897873

7.159003800 -0.628318531 253.255905313

7.264125705 -0.628318531 258.462152568

7.369247611 -0.628318531 268.533076861

7.474369516 -0.628318531 280.371515216

7.579491421 -0.628318531 285.635434931

7.684613326 -0.628318531 276.799064268

7.789735232 -0.628318531 257.540218636

7.894857137 -0.628318531 248.623878289

7.999979042 -0.628318531 281.120002425

8.105100947 -0.628318531 378.535801032

8.210222853 -0.628318531 540.380447193

8.315344758 -0.628318531 739.917012713

8.420466663 -0.628318531 938.225666824

8.525588568 -0.628318531 1102.792763806

8.630710474 -0.628318531 1218.803776978

8.735832379 -0.628318531 1288.471541850

8.840954284 -0.628318531 1323.714085283

8.946076189 -0.628318531 1339.121878391

9.051198095 -0.628318531 1344.673200405

9.156320000 -0.628318531 1346.293338083

-0.830261000 -0.488692191 1346.616981682

-0.725139095 -0.488692191 1345.937602597

-0.620017189 -0.488692191 1343.339844578

-0.514895284 -0.488692191 1334.300056751

-0.409773379 -0.488692191 1310.428394028

-0.304651474 -0.488692191 1259.138450465

-0.199529568 -0.488692191 1165.110164469

-0.094407663 -0.488692191 1016.867986565

0.010714242 -0.488692191 816.569968209

0.115836147 -0.488692191 587.385985572

0.220958053 -0.488692191 370.282230245

0.326079958 -0.488692191 208.028178417

0.431201863 -0.488692191 124.032439543

0.536323768 -0.488692191 94.469214739

0.641445674 -0.488692191 95.446309582

0.746567579 -0.488692191 105.883656458

0.851689484 -0.488692191 103.764331480

0.956811389 -0.488692191 83.618771877

1.061933295 -0.488692191 53.943995547

1.167055200 -0.488692191 28.015052986

1.272177105 -0.488692191 15.692790825

1.377299011 -0.488692191 20.191107841

1.482420916 -0.488692191 39.613738353

1.587542821 -0.488692191 69.194359030

1.692664726 -0.488692191 101.956312290

1.797786632 -0.488692191 131.470174780

1.902908537 -0.488692191 153.422694877

2.008030442 -0.488692191 161.369443027

2.113152347 -0.488692191 156.848467332

2.218274253 -0.488692191 152.405233080

2.323396158 -0.488692191 149.069850901

2.428518063 -0.488692191 147.215349938

2.533639968 -0.488692191 147.462345670

2.638761874 -0.488692191 150.145021665

2.743883779 -0.488692191 154.603083089

2.849005684 -0.488692191 157.727586282

2.954127589 -0.488692191 151.995376908

3.059249495 -0.488692191 146.051897681

3.164371400 -0.488692191 143.394016985

3.269493305 -0.488692191 144.421771916

3.374615211 -0.488692191 148.442978852

3.479737116 -0.488692191 154.313355926

3.584859021 -0.488692191 161.067767811

3.689980926 -0.488692191 167.941883454

3.795102832 -0.488692191 173.451716562

3.900224737 -0.488692191 174.114264039

4.005346642 -0.488692191 173.786995611

4.110468547 -0.488692191 174.940512352

4.215590453 -0.488692191 177.002943607

4.320712358 -0.488692191 178.317656388

4.425834263 -0.488692191 177.324795460

4.530956168 -0.488692191 175.753453156

4.636078074 -0.488692191 175.348811116

4.741199979 -0.488692191 176.346164003

4.846321884 -0.488692191 178.209990455

4.951443789 -0.488692191 180.194225827

5.056565695 -0.488692191 181.733062276

5.161687600 -0.488692191 182.636004229

5.266809505 -0.488692191 183.062003691

5.371931411 -0.488692191 183.256831402

5.477053316 -0.488692191 183.399652728

5.582175221 -0.488692191 183.616776958

5.687297126 -0.488692191 183.958040390

5.792419032 -0.488692191 184.486839578

5.897540937 -0.488692191 185.419261927

6.002662842 -0.488692191 186.694701428

6.107784747 -0.488692191 188.319427394

6.212906653 -0.488692191 190.904770125

6.318028558 -0.488692191 194.266242860

6.423150463 -0.488692191 197.739328414

6.528272368 -0.488692191 201.141301554

6.633394274 -0.488692191 205.220093325

6.738516179 -0.488692191 211.512086690

6.843638084 -0.488692191 221.478858289

6.948759989 -0.488692191 235.636425704

7.053881895 -0.488692191 251.101806013

7.159003800 -0.488692191 254.825557927

7.264125705 -0.488692191 259.864224358

7.369247611 -0.488692191 269.465774632

7.474369516 -0.488692191 280.533663523

7.579491421 -0.488692191 284.888649052

7.684613326 -0.488692191 275.250514842

7.789735232 -0.488692191 255.500145222

7.894857137 -0.488692191 246.482491313

7.999979042 -0.488692191 279.225589284

8.105100947 -0.488692191 377.090280772

8.210222853 -0.488692191 539.415911068

8.315344758 -0.488692191 739.348057652

8.420466663 -0.488692191 937.929005714

8.525588568 -0.488692191 1102.656951962

8.630710474 -0.488692191 1218.751132004

8.735832379 -0.488692191 1288.462484847

8.840954284 -0.488692191 1323.714712134

8.946076189 -0.488692191 1339.121130305

9.051198095 -0.488692191 1344.673187810

9.156320000 -0.488692191 1346.292935540

-0.830261000 -0.349065850 1346.617347119

-0.725139095 -0.349065850 1345.938638412

-0.620017189 -0.349065850 1343.352539390

-0.514895284 -0.349065850 1334.356437920

-0.409773379 -0.349065850 1310.498287248

-0.304651474 -0.349065850 1259.109794568

-0.199529568 -0.349065850 1164.829205627

-0.094407663 -0.349065850 1016.068134916

0.010714242 -0.349065850 814.874888098

0.115836147 -0.349065850 584.369009901

0.220958053 -0.349065850 365.625831977

0.326079958 -0.349065850 201.821093386

0.431201863 -0.349065850 117.128073611

0.536323768 -0.349065850 98.208088174

0.641445674 -0.349065850 101.657132337

0.746567579 -0.349065850 114.463555122

0.851689484 -0.349065850 115.616387655

0.956811389 -0.349065850 98.492395795

1.061933295 -0.349065850 70.821161215

1.167055200 -0.349065850 45.932392930

1.272177105 -0.349065850 34.073959820

1.377299011 -0.349065850 38.387349708

1.482420916 -0.349065850 56.410364488

1.587542821 -0.349065850 83.070625645

1.692664726 -0.349065850 111.999779660

1.797786632 -0.349065850 137.674224850

1.902908537 -0.349065850 156.234931869

2.008030442 -0.349065850 162.090327691

2.113152347 -0.349065850 157.867144518

2.218274253 -0.349065850 153.811021082

2.323396158 -0.349065850 150.765404515

2.428518063 -0.349065850 149.035792422

2.533639968 -0.349065850 149.360330452

2.638761874 -0.349065850 152.198431971

2.743883779 -0.349065850 156.788741868

2.849005684 -0.349065850 158.953164240

2.954127589 -0.349065850 151.548324304

3.059249495 -0.349065850 144.206648140

3.164371400 -0.349065850 140.437428374

3.269493305 -0.349065850 140.908273189

3.374615211 -0.349065850 144.893032028

3.479737116 -0.349065850 151.013574459

3.584859021 -0.349065850 158.048935492

3.689980926 -0.349065850 165.112335057

3.795102832 -0.349065850 170.979567673

3.900224737 -0.349065850 172.341238204

4.005346642 -0.349065850 172.090820483

4.110468547 -0.349065850 173.181463060

4.215590453 -0.349065850 175.222956945

4.320712358 -0.349065850 176.676516270

4.425834263 -0.349065850 176.236296276

4.530956168 -0.349065850 175.404787944

4.636078074 -0.349065850 175.551134148

4.741199979 -0.349065850 176.761229550

4.846321884 -0.349065850 178.517531777

4.951443789 -0.349065850 180.203551397

5.056565695 -0.349065850 181.427151779

5.161687600 -0.349065850 182.122523172

5.266809505 -0.349065850 182.497712328

5.371931411 -0.349065850 182.806612934

5.477053316 -0.349065850 183.198325037

5.582175221 -0.349065850 183.726621455

5.687297126 -0.349065850 184.358154715

5.792419032 -0.349065850 185.043386157

5.897540937 -0.349065850 185.810610043

6.002662842 -0.349065850 186.622615800

6.107784747 -0.349065850 187.944403131

6.212906653 -0.349065850 190.395823017

6.318028558 -0.349065850 193.655385865

6.423150463 -0.349065850 197.025439538

6.528272368 -0.349065850 200.313565754

6.633394274 -0.349065850 204.276189175

6.738516179 -0.349065850 210.467392701

6.843638084 -0.349065850 220.357846917

6.948759989 -0.349065850 234.458372886

7.053881895 -0.349065850 250.919905353

7.159003800 -0.349065850 256.260564180

7.264125705 -0.349065850 260.966731316

7.369247611 -0.349065850 269.926564536

7.474369516 -0.349065850 280.082891632

7.579491421 -0.349065850 283.448786525

7.684613326 -0.349065850 273.012353445

7.789735232 -0.349065850 252.868393279

7.894857137 -0.349065850 243.931405741

7.999979042 -0.349065850 277.141978490

8.105100947 -0.349065850 375.650332599

8.210222853 -0.349065850 538.579201879

8.315344758 -0.349065850 738.948567124

8.420466663 -0.349065850 937.784056392

8.525588568 -0.349065850 1102.628388890

8.630710474 -0.349065850 1218.761019503

8.735832379 -0.349065850 1288.478332381

8.840954284 -0.349065850 1323.721300217

8.946076189 -0.349065850 1339.121842128

9.051198095 -0.349065850 1344.673531235

9.156320000 -0.349065850 1346.292707315

-0.830261000 -0.209439510 1346.617979742

-0.725139095 -0.209439510 1345.940668322

-0.620017189 -0.209439510 1343.373520383

-0.514895284 -0.209439510 1334.454940827

-0.409773379 -0.209439510 1310.672830372

-0.304651474 -0.209439510 1259.293948252

-0.199529568 -0.209439510 1164.969026315

-0.094407663 -0.209439510 1016.024828603

0.010714242 -0.209439510 814.352305619

0.115836147 -0.209439510 582.870067026

0.220958053 -0.209439510 362.577977895

0.326079958 -0.209439510 196.846189181

0.431201863 -0.209439510 110.197247537

0.536323768 -0.209439510 95.552324350

0.641445674 -0.209439510 109.781183880

0.746567579 -0.209439510 124.479912318

0.851689484 -0.209439510 127.309767244

0.956811389 -0.209439510 111.155112970

1.061933295 -0.209439510 83.721239309

1.167055200 -0.209439510 58.991233534

1.272177105 -0.209439510 47.742086058

1.377299011 -0.209439510 52.826944741

1.482420916 -0.209439510 70.747357359

1.587542821 -0.209439510 95.580624268

1.692664726 -0.209439510 121.382901345

1.797786632 -0.209439510 143.573443367

1.902908537 -0.209439510 158.844192945

2.008030442 -0.209439510 161.361313369

2.113152347 -0.209439510 156.801939137

2.218274253 -0.209439510 153.074389398

2.323396158 -0.209439510 150.505276375

2.428518063 -0.209439510 149.348582132

2.533639968 -0.209439510 150.331094116

2.638761874 -0.209439510 153.828822484

2.743883779 -0.209439510 158.789294001

2.849005684 -0.209439510 158.503514615

2.954127589 -0.209439510 149.707475194

3.059249495 -0.209439510 141.762895070

3.164371400 -0.209439510 137.691852846

3.269493305 -0.209439510 138.226177864

3.374615211 -0.209439510 142.478764499

3.479737116 -0.209439510 148.810343121

3.584859021 -0.209439510 155.817132171

3.689980926 -0.209439510 162.627334150

3.795102832 -0.209439510 168.342181113

3.900224737 -0.209439510 170.296517289

4.005346642 -0.209439510 170.285916267

4.110468547 -0.209439510 171.432389713

4.215590453 -0.209439510 173.523269564

4.320712358 -0.209439510 175.003375669

4.425834263 -0.209439510 174.807377037

4.530956168 -0.209439510 174.423963695

4.636078074 -0.209439510 174.902809349

4.741199979 -0.209439510 176.216842513

4.846321884 -0.209439510 177.897984552

4.951443789 -0.209439510 179.448640509

5.056565695 -0.209439510 180.581124750

5.161687600 -0.209439510 181.263052737

5.266809505 -0.209439510 181.715600997

5.371931411 -0.209439510 182.218955008

5.477053316 -0.209439510 182.915342412

5.582175221 -0.209439510 183.775109797

5.687297126 -0.209439510 184.647740130

5.792419032 -0.209439510 185.372714144

5.897540937 -0.209439510 185.914178451

6.002662842 -0.209439510 186.454374957

6.107784747 -0.209439510 187.699611507

6.212906653 -0.209439510 190.157288805

6.318028558 -0.209439510 193.434086462

6.423150463 -0.209439510 196.806948266

6.528272368 -0.209439510 200.056247505

6.633394274 -0.209439510 203.945091908

6.738516179 -0.209439510 210.064924208

6.843638084 -0.209439510 219.914339995

6.948759989 -0.209439510 233.993694401

7.053881895 -0.209439510 250.790753517

7.159003800 -0.209439510 257.447197607

7.264125705 -0.209439510 261.711743218

7.369247611 -0.209439510 269.965689348

7.474369516 -0.209439510 279.228893818

7.579491421 -0.209439510 281.710806286

7.684613326 -0.209439510 270.647754461

7.789735232 -0.209439510 250.316937690

7.894857137 -0.209439510 241.663495329

7.999979042 -0.209439510 275.492803243

8.105100947 -0.209439510 374.709169705

8.210222853 -0.209439510 538.214903482

8.315344758 -0.209439510 738.931409040

8.420466663 -0.209439510 937.906514071

8.525588568 -0.209439510 1102.762194364

8.630710474 -0.209439510 1218.856126004

8.735832379 -0.209439510 1288.527214102

8.840954284 -0.209439510 1323.737015983

8.946076189 -0.209439510 1339.125295265

9.051198095 -0.209439510 1344.674560221

9.156320000 -0.209439510 1346.292698166

-0.830261000 -0.069813170 1346.617760010

-0.725139095 -0.069813170 1345.939759797

-0.620017189 -0.069813170 1343.387492191

-0.514895284 -0.069813170 1334.555471231

-0.409773379 -0.069813170 1310.896931549

-0.304651474 -0.069813170 1259.636773299

-0.199529568 -0.069813170 1165.505330082

-0.094407663 -0.069813170 1016.801289950

0.010714242 -0.069813170 815.246484528

0.115836147 -0.069813170 583.457960120

0.220958053 -0.069813170 362.171102486

0.326079958 -0.069813170 194.660485097

0.431201863 -0.069813170 105.583708817

0.536323768 -0.069813170 88.419194211

0.641445674 -0.069813170 110.262433925

0.746567579 -0.069813170 132.320386149

0.851689484 -0.069813170 135.961798239

0.956811389 -0.069813170 118.585591042

1.061933295 -0.069813170 89.617862954

1.167055200 -0.069813170 64.187271939

1.272177105 -0.069813170 53.579167153

1.377299011 -0.069813170 60.139041101

1.482420916 -0.069813170 79.128686867

1.587542821 -0.069813170 103.530379420

1.692664726 -0.069813170 127.538206537

1.797786632 -0.069813170 147.360471171

1.902908537 -0.069813170 160.218834163

2.008030442 -0.069813170 160.036427558

2.113152347 -0.069813170 154.969093150

2.218274253 -0.069813170 151.365373117

2.323396158 -0.069813170 149.240051206

2.428518063 -0.069813170 148.813162051

2.533639968 -0.069813170 150.719419182

2.638761874 -0.069813170 155.121437100

2.743883779 -0.069813170 160.199342074

2.849005684 -0.069813170 156.771675234

2.954127589 -0.069813170 147.830092924

3.059249495 -0.069813170 140.170564528

3.164371400 -0.069813170 136.492353773

3.269493305 -0.069813170 137.469092804

3.374615211 -0.069813170 142.033664922

3.479737116 -0.069813170 148.354316230

3.584859021 -0.069813170 154.953369281

3.689980926 -0.069813170 161.084935054

3.795102832 -0.069813170 166.201021976

3.900224737 -0.069813170 168.362451281

4.005346642 -0.069813170 168.573838751

4.110468547 -0.069813170 169.797717904

4.215590453 -0.069813170 171.926080369

4.320712358 -0.069813170 173.243381397

4.425834263 -0.069813170 172.981755981

4.530956168 -0.069813170 172.732358576

4.636078074 -0.069813170 173.307626068

4.741199979 -0.069813170 174.624873081

4.846321884 -0.069813170 176.279390645

4.951443789 -0.069813170 177.864892585

5.056565695 -0.069813170 179.141341930

5.161687600 -0.069813170 180.026571694

5.266809505 -0.069813170 180.680458233

5.371931411 -0.069813170 181.394966611

5.477053316 -0.069813170 182.343445306

5.582175221 -0.069813170 183.469021530

5.687297126 -0.069813170 184.533358346

5.792419032 -0.069813170 185.301342968

5.897540937 -0.069813170 185.779713613

6.002662842 -0.069813170 186.333812003

6.107784747 -0.069813170 187.699312051

6.212906653 -0.069813170 190.296277302

6.318028558 -0.069813170 193.720936455

6.423150463 -0.069813170 197.230627023

6.528272368 -0.069813170 200.555955287

6.633394274 -0.069813170 204.444380923

6.738516179 -0.069813170 210.521272091

6.843638084 -0.069813170 220.325705369

6.948759989 -0.069813170 234.361167579

7.053881895 -0.069813170 251.183900654

7.159003800 -0.069813170 258.290414853

7.264125705 -0.069813170 262.120606361

7.369247611 -0.069813170 269.772647219

7.474369516 -0.069813170 278.353102746

7.579491421 -0.069813170 280.243371487

7.684613326 -0.069813170 268.875316756

7.789735232 -0.069813170 248.645659017

7.894857137 -0.069813170 240.468113458

7.999979042 -0.069813170 274.969640798

8.105100947 -0.069813170 374.806704445

8.210222853 -0.069813170 538.699391333

8.315344758 -0.069813170 739.530289654

8.420466663 -0.069813170 938.424985827

8.525588568 -0.069813170 1103.120863826

8.630710474 -0.069813170 1219.062906655

8.735832379 -0.069813170 1288.618783372

8.840954284 -0.069813170 1323.765397813

8.946076189 -0.069813170 1339.132745666

9.051198095 -0.069813170 1344.676569045

9.156320000 -0.069813170 1346.292911861

-0.830261000 0.069813170 1346.615768038

-0.725139095 0.069813170 1345.932838175

-0.620017189 0.069813170 1343.381720854

-0.514895284 0.069813170 1334.622031787

-0.409773379 0.069813170 1311.133489627

-0.304651474 0.069813170 1260.122038271

-0.199529568 0.069813170 1166.462140223

-0.094407663 0.069813170 1018.509230102

0.010714242 0.069813170 817.846848890

0.115836147 0.069813170 586.725981058

0.220958053 0.069813170 365.377236732

0.326079958 0.069813170 196.615130922

0.431201863 0.069813170 104.998180041

0.536323768 0.069813170 84.403072569

0.641445674 0.069813170 102.660156833

0.746567579 0.069813170 124.148685632

0.851689484 0.069813170 132.143566453

0.956811389 0.069813170 122.147624683

1.061933295 0.069813170 91.232122826

1.167055200 0.069813170 64.866190044

1.272177105 0.069813170 54.680652800

1.377299011 0.069813170 62.415775657

1.482420916 0.069813170 82.282199315

1.587542821 0.069813170 106.471666368

1.692664726 0.069813170 129.362312288

1.797786632 0.069813170 147.810608838

1.902908537 0.069813170 159.517531823

2.008030442 0.069813170 159.239198911

2.113152347 0.069813170 153.659937498

2.218274253 0.069813170 149.833543361

2.323396158 0.069813170 147.884567764

2.428518063 0.069813170 148.047107087

2.533639968 0.069813170 150.797473364

2.638761874 0.069813170 155.970928416

2.743883779 0.069813170 160.089305784

2.849005684 0.069813170 155.451428624

2.954127589 0.069813170 147.539205700

3.059249495 0.069813170 140.827637293

3.164371400 0.069813170 137.834446939

3.269493305 0.069813170 139.183917770

3.374615211 0.069813170 143.734942926

3.479737116 0.069813170 149.621795263

3.584859021 0.069813170 155.419166576

3.689980926 0.069813170 160.561887617

3.795102832 0.069813170 164.829215014

3.900224737 0.069813170 166.864159911

4.005346642 0.069813170 167.131597357

4.110468547 0.069813170 168.350660987

4.215590453 0.069813170 170.418499651

4.320712358 0.069813170 171.339998710

4.425834263 0.069813170 170.769527865

4.530956168 0.069813170 170.407280757

4.636078074 0.069813170 170.903020469

4.741199979 0.069813170 172.164912459

4.846321884 0.069813170 173.838529042

4.951443789 0.069813170 175.566860044

5.056565695 0.069813170 177.133696837

5.161687600 0.069813170 178.400398503

5.266809505 0.069813170 179.403722338

5.371931411 0.069813170 180.376979296

5.477053316 0.069813170 181.518773426

5.582175221 0.069813170 182.815246935

5.687297126 0.069813170 184.040146736

5.792419032 0.069813170 184.951043524

5.897540937 0.069813170 185.590154855

6.002662842 0.069813170 186.375271100

6.107784747 0.069813170 187.995467101

6.212906653 0.069813170 190.835165800

6.318028558 0.069813170 194.519803388

6.423150463 0.069813170 198.296600025

6.528272368 0.069813170 201.830860302

6.633394274 0.069813170 205.822439436

6.738516179 0.069813170 211.904519476

6.843638084 0.069813170 221.651619361

6.948759989 0.069813170 235.585620140

7.053881895 0.069813170 252.210635711

7.159003800 0.069813170 258.752331713

7.264125705 0.069813170 262.279994442

7.369247611 0.069813170 269.585137763

7.474369516 0.069813170 277.835799796

7.579491421 0.069813170 279.541137185

7.684613326 0.069813170 268.265163201

7.789735232 0.069813170 248.452107550

7.894857137 0.069813170 240.913242732

7.999979042 0.069813170 276.061208798

8.105100947 0.069813170 376.324473027

8.210222853 0.069813170 540.302974840

8.315344758 0.069813170 740.918325725

8.420466663 0.069813170 939.439213221

8.525588568 0.069813170 1103.755963279

8.630710474 0.069813170 1219.404878537

8.735832379 0.069813170 1288.761328069

8.840954284 0.069813170 1323.808664709

8.946076189 0.069813170 1339.144719038

9.051198095 0.069813170 1344.679636908

9.156320000 0.069813170 1346.293300371

-0.830261000 0.209439510 1346.611579902

-0.725139095 0.209439510 1345.918619379

-0.620017189 0.209439510 1343.350317561

-0.514895284 0.209439510 1334.634098422

-0.409773379 0.209439510 1311.369711733

-0.304651474 0.209439510 1260.771274033

-0.199529568 0.209439510 1167.895277137

-0.094407663 0.209439510 1021.253425925

0.010714242 0.209439510 822.345153482

0.115836147 0.209439510 592.983621587

0.220958053 0.209439510 372.614465547

0.326079958 0.209439510 203.212746554

0.431201863 0.209439510 108.979263632

0.536323768 0.209439510 84.368414219

0.641445674 0.209439510 98.498316917

0.746567579 0.209439510 117.544826659

0.851689484 0.209439510 124.553790012

0.956811389 0.209439510 121.610875847

1.061933295 0.209439510 93.260915345

1.167055200 0.209439510 66.785640853

1.272177105 0.209439510 56.803277462

1.377299011 0.209439510 64.447001886

1.482420916 0.209439510 83.481584074

1.587542821 0.209439510 106.124239720

1.692664726 0.209439510 127.391973105

1.797786632 0.209439510 144.785907407

1.902908537 0.209439510 156.248166849

2.008030442 0.209439510 158.711123169

2.113152347 0.209439510 153.193387501

2.218274253 0.209439510 148.778277175

2.323396158 0.209439510 146.665051270

2.428518063 0.209439510 147.149451822

2.533639968 0.209439510 150.472059646

2.638761874 0.209439510 155.994712235

2.743883779 0.209439510 159.324085542

2.849005684 0.209439510 155.801156157

2.954127589 0.209439510 149.560466449

3.059249495 0.209439510 144.105666322

3.164371400 0.209439510 141.745614656

3.269493305 0.209439510 143.105578724

3.374615211 0.209439510 147.130504704

3.479737116 0.209439510 152.110649051

3.584859021 0.209439510 156.794901250

3.689980926 0.209439510 160.806135482

3.795102832 0.209439510 164.171266430

3.900224737 0.209439510 165.891941475

4.005346642 0.209439510 165.994207433

4.110468547 0.209439510 167.067521984

4.215590453 0.209439510 168.958838044

4.320712358 0.209439510 169.331833049

4.425834263 0.209439510 168.319915972

4.530956168 0.209439510 167.692009196

4.636078074 0.209439510 168.016013123

4.741199979 0.209439510 169.216320171

4.846321884 0.209439510 170.958375320

4.951443789 0.209439510 172.880226062

5.056565695 0.209439510 174.756408868

5.161687600 0.209439510 176.434683372

5.266809505 0.209439510 177.880260946

5.371931411 0.209439510 179.224580107

5.477053316 0.209439510 180.613575105

5.582175221 0.209439510 182.069846637

5.687297126 0.209439510 183.458169883

5.792419032 0.209439510 184.594935186

5.897540937 0.209439510 185.528345425

6.002662842 0.209439510 186.650458326

6.107784747 0.209439510 188.591758141

6.212906653 0.209439510 191.731264260

6.318028558 0.209439510 195.744246569

6.423150463 0.209439510 199.879546496

6.528272368 0.209439510 203.734057349

6.633394274 0.209439510 207.933855722

6.738516179 0.209439510 214.085666801

6.843638084 0.209439510 223.779358105

6.948759989 0.209439510 237.560091694

7.053881895 0.209439510 253.715578728

7.159003800 0.209439510 258.862974069

7.264125705 0.209439510 262.290385990

7.369247611 0.209439510 269.561020704

7.474369516 0.209439510 277.864936619

7.579491421 0.209439510 279.790002377

7.684613326 0.209439510 268.979487803

7.789735232 0.209439510 249.868125974

7.894857137 0.209439510 243.101613330

7.999979042 0.209439510 278.845117355

8.105100947 0.209439510 379.320329696

8.210222853 0.209439510 543.068580668

8.315344758 0.209439510 743.126820055

8.420466663 0.209439510 940.970822587

8.525588568 0.209439510 1104.681269158

8.630710474 0.209439510 1219.889406080

8.735832379 0.209439510 1288.955996272

8.840954284 0.209439510 1323.865716365

8.946076189 0.209439510 1339.160418687

9.051198095 0.209439510 1344.683497038

9.156320000 0.209439510 1346.293758561

-0.830261000 0.349065850 1346.605210573

-0.725139095 0.349065850 1345.897165160

-0.620017189 0.349065850 1343.292895840

-0.514895284 0.349065850 1334.583099522

-0.409773379 0.349065850 1311.589662961

-0.304651474 0.349065850 1261.592565453

-0.199529568 0.349065850 1169.803124648

-0.094407663 0.349065850 1024.977797135

0.010714242 0.349065850 828.578299960

0.115836147 0.349065850 601.901340649

0.220958053 0.349065850 383.357251833

0.326079958 0.349065850 213.761419429

0.431201863 0.349065850 116.720734188

0.536323768 0.349065850 87.368134878

0.641445674 0.349065850 96.874818744

0.746567579 0.349065850 113.074794499

0.851689484 0.349065850 119.501335069

0.956811389 0.349065850 117.407989298

1.061933295 0.349065850 98.550318189

1.167055200 0.349065850 73.804223020

1.272177105 0.349065850 64.250133312

1.377299011 0.349065850 70.408017248

1.482420916 0.349065850 86.345307658

1.587542821 0.349065850 105.308035560

1.692664726 0.349065850 123.603707242

1.797786632 0.349065850 139.508853279

1.902908537 0.349065850 150.921674803

2.008030442 0.349065850 155.754000842

2.113152347 0.349065850 152.324865478

2.218274253 0.349065850 147.419001896

2.323396158 0.349065850 145.126690387

2.428518063 0.349065850 145.792224717

2.533639968 0.349065850 149.453030984

2.638761874 0.349065850 155.116320303

2.743883779 0.349065850 158.952451709

2.849005684 0.349065850 157.451140330

2.954127589 0.349065850 153.148059797

3.059249495 0.349065850 149.049698240

3.164371400 0.349065850 147.201023806

3.269493305 0.349065850 148.245721564

3.374615211 0.349065850 151.338705197

3.479737116 0.349065850 155.085215269

3.584859021 0.349065850 158.513804934

3.689980926 0.349065850 161.427245787

3.795102832 0.349065850 163.987416437

3.900224737 0.349065850 165.293638497

4.005346642 0.349065850 165.001944433

4.110468547 0.349065850 165.813573523

4.215590453 0.349065850 167.521943595

4.320712358 0.349065850 167.345680322

4.425834263 0.349065850 165.823670318

4.530956168 0.349065850 164.839391980

4.636078074 0.349065850 164.955097006

4.741199979 0.349065850 166.119684002

4.846321884 0.349065850 167.989538742

4.951443789 0.349065850 170.152663973

5.056565695 0.349065850 172.326237331

5.161687600 0.349065850 174.340989466

5.266809505 0.349065850 176.173635967

5.371931411 0.349065850 177.941486627

5.477053316 0.349065850 179.714402865

5.582175221 0.349065850 181.442926438

5.687297126 0.349065850 183.047596328

5.792419032 0.349065850 184.444801666

5.897540937 0.349065850 185.704612197

6.002662842 0.349065850 187.181319839

6.107784747 0.349065850 189.453116837

6.212906653 0.349065850 192.903961051

6.318028558 0.349065850 197.262473630

6.423150463 0.349065850 201.786600877

6.528272368 0.349065850 206.012447901

6.633394274 0.349065850 210.483520525

6.738516179 0.349065850 216.756447348

6.843638084 0.349065850 226.414873440

6.948759989 0.349065850 240.021238811

7.053881895 0.349065850 255.260066752

7.159003800 0.349065850 258.705750186

7.264125705 0.349065850 262.208282058

7.369247611 0.349065850 269.692927159

7.474369516 0.349065850 278.342911459

7.579491421 0.349065850 280.784754593

7.684613326 0.349065850 270.706326085

7.789735232 0.349065850 252.502637576

7.894857137 0.349065850 246.614430339

7.999979042 0.349065850 282.927345646

8.105100947 0.349065850 383.463181300

8.210222853 0.349065850 546.746896809

8.315344758 0.349065850 745.988345065

8.420466663 0.349065850 942.919811192

8.525588568 0.349065850 1105.843876327

8.630710474 0.349065850 1220.491008179

8.735832379 0.349065850 1289.189945234

8.840954284 0.349065850 1323.930731697

8.946076189 0.349065850 1339.177559858

9.051198095 0.349065850 1344.687527266

9.156320000 0.349065850 1346.294130674

-0.830261000 0.488692191 1346.596922597

-0.725139095 0.488692191 1345.869035508

-0.620017189 0.488692191 1343.210822520

-0.514895284 0.488692191 1334.462132471

-0.409773379 0.488692191 1311.740430021

-0.304651474 0.488692191 1262.525534226

-0.199529568 0.488692191 1172.063107544

-0.094407663 0.488692191 1029.390043130

0.010714242 0.488692191 835.968627828

0.115836147 0.488692191 612.539444099

0.220958053 0.488692191 396.321602130

0.326079958 0.488692191 226.761477410

0.431201863 0.488692191 126.712349045

0.536323768 0.488692191 91.960950989

0.641445674 0.488692191 96.186545991

0.746567579 0.488692191 108.896623998

0.851689484 0.488692191 114.498044300

0.956811389 0.488692191 113.199948408

1.061933295 0.488692191 106.433921117

1.167055200 0.488692191 85.965295238

1.272177105 0.488692191 77.775344255

1.377299011 0.488692191 81.948831515

1.482420916 0.488692191 93.377837433

1.587542821 0.488692191 107.031640338

1.692664726 0.488692191 120.992747284

1.797786632 0.488692191 134.486231999

1.902908537 0.488692191 145.320566461

2.008030442 0.488692191 150.960336224

2.113152347 0.488692191 149.377984514

2.218274253 0.488692191 144.586467410

2.323396158 0.488692191 142.713014837

2.428518063 0.488692191 143.634370404

2.533639968 0.488692191 147.522905053

2.638761874 0.488692191 153.295950381

2.743883779 0.488692191 158.022754679

2.849005684 0.488692191 158.734367831

2.954127589 0.488692191 156.508009344

3.059249495 0.488692191 153.894835738

3.164371400 0.488692191 152.617871645

3.269493305 0.488692191 153.292606909

3.374615211 0.488692191 155.331726933

3.479737116 0.488692191 157.766438074

3.584859021 0.488692191 159.997140474

3.689980926 0.488692191 162.008125180

3.795102832 0.488692191 163.978788461

3.900224737 0.488692191 164.759840532

4.005346642 0.488692191 163.818523153

4.110468547 0.488692191 164.352965171

4.215590453 0.488692191 166.084386477

4.320712358 0.488692191 165.508172546

4.425834263 0.488692191 163.414264041

4.530956168 0.488692191 161.999315968

4.636078074 0.488692191 161.886263610

4.741199979 0.488692191 163.052650230

4.846321884 0.488692191 165.125388401

4.951443789 0.488692191 167.610367277

5.056565695 0.488692191 170.122701898

5.161687600 0.488692191 172.432505625

5.266809505 0.488692191 174.534198747

5.371931411 0.488692191 176.622287385

5.477053316 0.488692191 178.815063163

5.582175221 0.488692191 180.966141106

5.687297126 0.488692191 182.896775559

5.792419032 0.488692191 184.575444528

5.897540937 0.488692191 186.135173862

6.002662842 0.488692191 187.928162795

6.107784747 0.488692191 190.499397384

6.212906653 0.488692191 194.239045742
[truncated: 203,230 more chars]
